# 1-Hydroxyalkylphosphonium Salts—Synthesis and Properties

**DOI:** 10.3390/molecules29010018

**Published:** 2023-12-19

**Authors:** Jakub Adamek, Anna Kuźnik, Agnieszka Październiok-Holewa, Mirosława Grymel, Dominika Kozicka, Dominika Mierzwa, Karol Erfurt

**Affiliations:** 1 Department of Organic Chemistry, Bioorganic Chemistry and Biotechnology, Silesian University of Technology, B. Krzywoustego 4, 44-100 Gliwice, Poland; anna.kuznik@polsl.pl (A.K.); agnieszka.pazdzierniok@polsl.pl (A.P.-H.); miroslawa.grymel@polsl.pl (M.G.); dominika.kozicka@polsl.pl (D.K.); dominikamierzwaa@gmail.com (D.M.); 2 Biotechnology Center, Silesian University of Technology, B. Krzywoustego 8, 44-100 Gliwice, Poland; 3 Department of Chemical Organic Technology and Petrochemistry, Faculty of Chemistry, Silesian University of Technology, B. Krzywoustego 4, 44-100 Gliwice, Poland; karol.erfurt@polsl.pl

**Keywords:** phosphonium salts, synthesis, stability, alkylation, alkylating agent

## Abstract

An efficient and convenient method for the synthesis of 1-hydroxyalkylphosphonium salts is described. Reactions were carried out at room temperature, in a short time, and without chromatography for product isolation. The properties of the obtained phosphonium salts were examined and discussed. In this paper, primary attention was paid to the stability of phosphonium salts, depending on the structure of the aldehydes used as substrates in their preparation. Other conditions such as the type of solvent, temperature, and molar ratio of the substrates were also investigated. Finally, the high reactivity of 1-hydroxyalkylphosphonium salts was demonstrated in reactions with amide-type substrates and (hetero)aromatic compounds. The developed step-by-step procedure (with the isolation of 1-hydroxyphosphonium salts) was compared to the one-pot protocol (in situ formation of such phosphonium salts).

## 1. Introduction

Chemical compounds containing a phosphonium moiety play an increasingly important role in organic synthesis. They are already known not only for their use in the Wittig reaction as ylide precursors [1,2,3], but also as convenient building blocks used in many other reactions, especially various types of couplings [4,5,6,7,8]. Moreover, they are successfully used as solvents and catalysts (PILs—phosphonium ionic liquids are good examples here) [9,10,11,12,13]. Due to some specific properties, the presence of a phosphonium group can also influence the biological properties of the whole molecule (e.g., (TPP^+^)-based mitochondria-targeted compounds) [14]. 

**Scheme 1:**
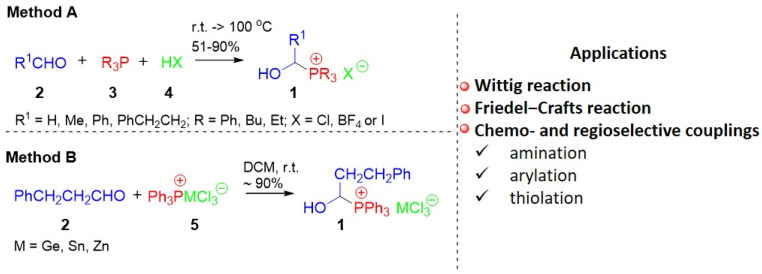
1-hydroxyalkylphosphonium salts **1**: synthesis and applications.

One of the relatively poorly known classes of phosphonium salts is 1-hydroxyalkylphosphonium salts **1**. Although such phosphonium salts were already described in the 1960s [15,16,17], their properties (apart from spectroscopic ones [18,19]) have not been studied in detail thus far. However, there have been some references to the synthesis of these compounds based on the three-component reaction of an aldehyde with phosphine in the presence of HX (aqueous or ethereal HBF_4_ solution, concentrated hydrochloric acid, etc., Scheme 1/Method A) [15,16,17,18,20,21,22] or the two-component reaction in the presence of complex triphenylphosphonium salts **5** (Scheme 1/Method B) [23]. Each time, they refer to individual examples—most often the synthesis of hydroxymethylphosphonium salts (R^1^ = H, from paraformaldehyde or formalin). In many cases, phosphonium salts **1** were further processed without isolation and purification [6,24,25]. They perfectly fulfill the role of building blocks in Wittig reactions (e.g., the Anders-Gassner variant) [20,26,27], Friedel–Crafts reactions, or other couplings characterized by high chemo- and regioselectivity [6,24,25].

Therefore, we present our research related to the synthesis and isolation of 1-hydroxyphosphonium salts as well as their properties, with particular emphasis on their stability, and reactivity with selected reagents.

## 2. Results and Discussion

Our journey to discover the properties of 1-hydroxyalkylphosphonium salts began with the synthesis of a wide range of such salts by slightly modifying the methods already described in the literature [6,24]. To this end, we conducted two-component couplings of an aldehyde (aliphatic, aromatic, simple, and more complex) with triarylphosphonium salts in a molar ratio of 1:1 (see Table 1). We decided to use previously synthesized [24] or commercially available triarylphosphonium salts Ar_3_P∙HX (HP^+^Ar_3_ X^−^) in the reaction, rather than generating them in situ from the appropriate phosphines and acids. 

In general, the reactions were carried out at room temperature, but in some cases (see paraformaldehyde) raising the temperature was necessary. The type of solvent affects the course of the reaction. Acetonitrile, chloroform, and dichloromethane (DCM) turned out to be the most effective. However, we also noticed that the use of THF leads to a complex reaction mixture (Table 1, entries 1–3). We demonstrated that the described procedure can be applied to a variety of phosphonium salts Ar_3_P∙HX (HP^+^Ar_3_ X^−^). The anion (X^−^) does not play a significant role, while the triarylphosphonium group is crucial here. So far, we have not been able to obtain phosphonium salt derivatives of triarylphosphines substituted with electron-withdrawing substituents (e.g., P(3-C_6_H_4_Cl)_3_). Triphenylphosphonium salts and derivatives of triarylphosphines substituted with electron-donating substituents (PPh_3_ or P(4-C_6_H_4_OMe)_3_) can be synthesized without any problems (compare entries 5–7, Table 1). The reaction in which Ph_3_P·HBF_4_ was generated in situ using PPh_3_ and HBF_4_ (tetrafluoroboric acid diethyl ether complex) was also performed, but it turned out to be less efficient (92 vs. 86%—Table 1, entry 1).

The structure of the aldehyde appears to be most important to the success of the reaction. The synthesis of pure products from aliphatic aldehydes does not cause any significant problems (see Table 2). Even the use of hydrated chloroacetaldehyde allowed the expected product to be obtained with a high yield (Table 2, entry 8). The reactions can also be scaled up and conducted at a gram-scale. We demonstrated this for the reaction of paraformaldehyde with Ph_3_P·HBr (Table 2, entry 5), obtaining 2.6 g of product **1h** with a yield of 87%. 

**Scheme 2:**
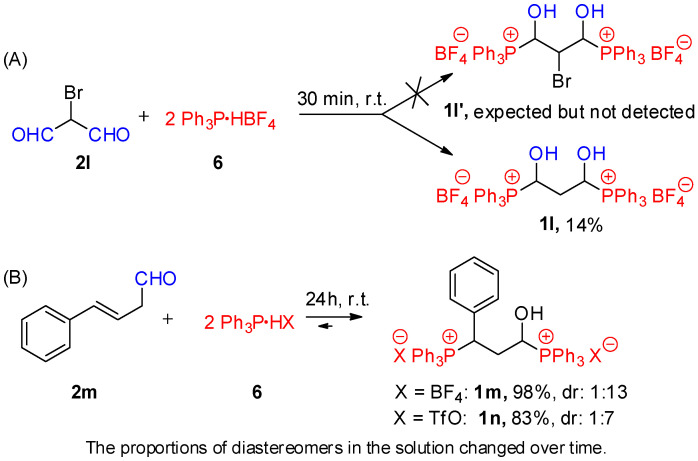
Hydroxyalkylphosphonium salts from bromomalonaldehyde (**A**) or (*E*)-cinnamaldehyde (**B**): conditions and results.

Interestingly, we also managed to obtain hydroxyalkylphosphonium salts from bis-aldehyde systems (e.g., bromomalonaldehyde; see compound **1l**) or unsaturated aldehydes (e.g., *(E)*-cinnamaldehyde; see compounds **1m** and **1n**). In both cases, we had to use an excess of triphenylphosphonium salt (the molar ratio of substrates **2**:**6** was 1:2); otherwise, complex reaction mixtures were obtained. Furthermore, in the reaction of bromomalonoaldehyde, we isolated only compound **1l**, most likely due to the reductive dehalogenation that occurred (Scheme 2).

**Figure 1:**
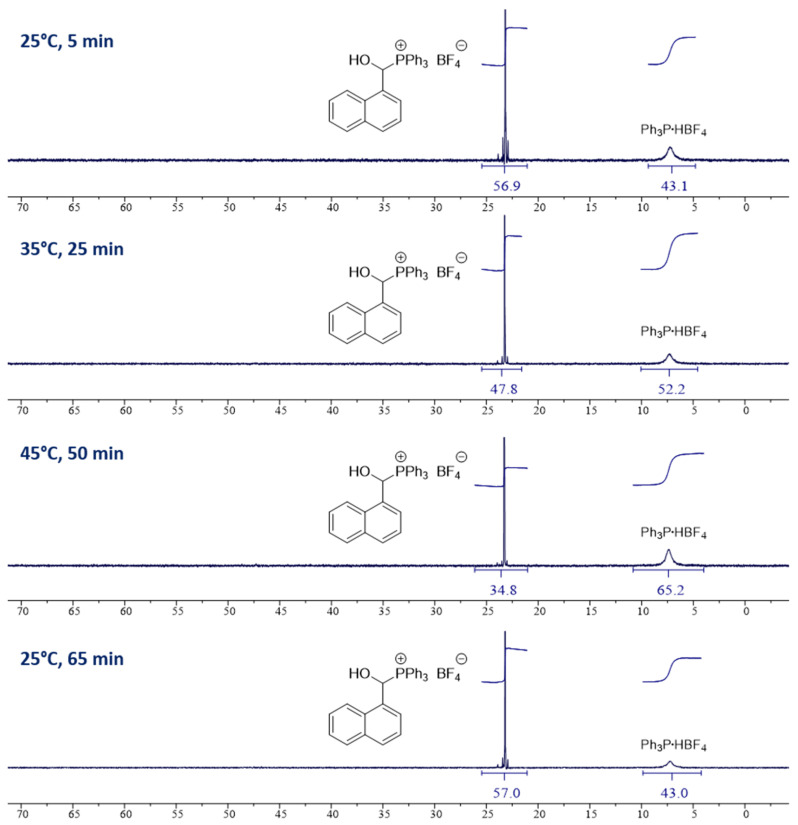
^31^P{^1^H} NMR spectra (162 MHz/CD_3_CN) of reaction mixtures (1-naphthaldehyde with triphenylphosphonium tetrafluoroborate) recorded at 25 °C, 35 °C, 45 °C, and back to 25 °C.

On the other hand, in the case of bulky aldehydes (R^1^ = *i*-Pr) and especially aromatic aldehydes, we observed the incomplete conversion of substrates (see Figure 1, Table 3 and Table 4). We assumed that equilibrium was reached and decided to investigate this phenomenon closely (see also Table S1, Supporting Information). Reactions between 1-naphthaldehyde and triarylphosphonium salts Ar_3_P∙HX (HP^+^Ar_3_ X^−^) were chosen as model reactions (see Figure 1 and Table 3). As we checked (^1^H NMR and ^31^P{^1^H} NMR), the reaction initially proceeds very quickly (until about 5 min), after which the composition of the reaction mixture does not change at a given temperature (Table 3, entries 1–3). 

Next, we performed some temperature tests using NMR (VT techniques) and determined the composition of the reaction mixture after increasing the temperature every 10 degrees (from 25 °C to 45 °C and back to 25 °C, see Figure 1 and Table 3). Regardless of the type of phosphonium salt used (anion X or phosphonium moiety Ar_3_P), increasing the temperature shifts the equilibrium towards the substrates (see entries 4–15 and Figure 1). Coming back to the initial temperature (25 °C) returns it to the starting composition of the reaction mixture (e.g., compare entries 4–6, Table 3). The equilibrium state itself is slightly affected by the type of solvent used (see CD_3_CN vs. CDCl_3_, entries 4–6 and 13–15) and the counteranion of the phosphonium salt **6** (compare BF_4_ vs. TfO, entries 4–9). We also observed that the use of excess aldehyde **2o** (2:1, 3:1, and 5:1; entries 16–19, Table 3) moves the reaction equilibrium toward product **1**.

The presence of equilibria was confirmed for the reactions of structurally diverse aldehydes, mostly aromatic but sometimes also aliphatic (e.g., isobutyraldehyde, cyclohexanecarboaldehyde; see Table 4). Generally, in CD_3_CN the equilibrium is clearly shifted toward the 1-hydroxyalkylphosphonium salts. In CDCl_3_, it looks less favorable, most likely due to the acidic nature of this solvent. Furthermore, the (hetero)aromatic systems react less willingly, giving reaction mixtures with lower contents of 1-hydroxyalkylphosphonium salts **1**.

Attempts to isolate pure 1-aryl-1-hydroxyalkylphosphonium salts, e.g., by crystallization from the CH_2_Cl_2_/Et_2_O or CD_3_CN/Et_2_O, failed (the crystallization leads to the precipitation of Ar_3_P∙HX rather than to 1-aryl-1-hydroxyalkylphosphonium salts). 

Masarwa et al. reported similar observations for many aromatic aldehydes. They were also unable to isolate 1-hydroxyalkylphosphonium salts either through crystallization or flash chromatography, but they did not provide any explanation for this problem [6].

**Scheme 3:**
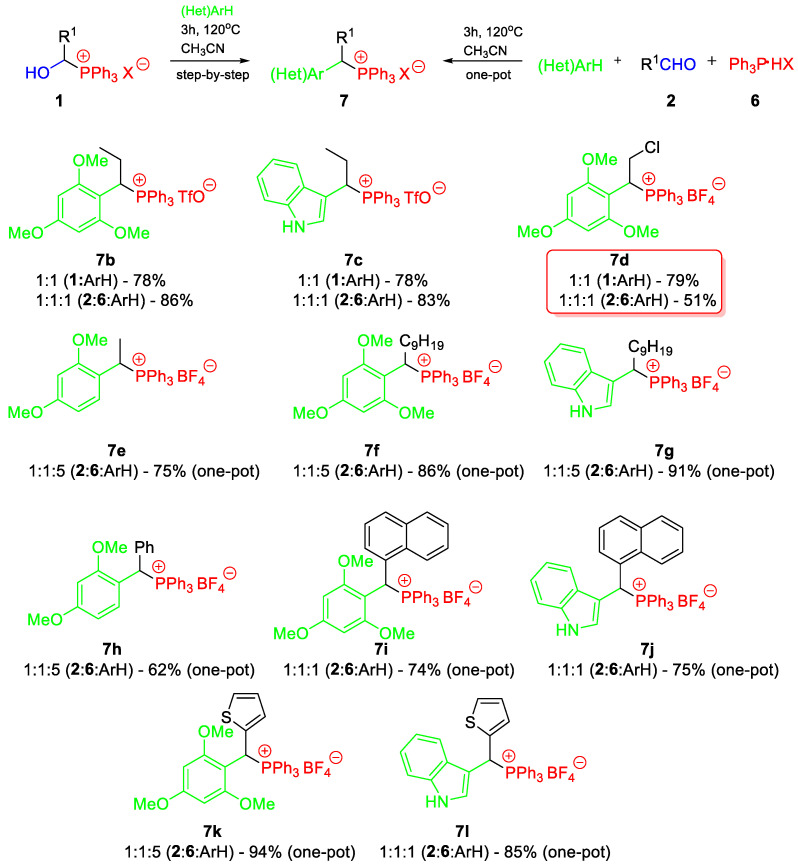
Conditions and results for the reaction of 1-hydroxyalkylphosphonium salts **1** with (hetero)aromatic compounds (step-by-step vs. one-pot protocol).

The high reactivity of the 1-hydroxyalkylphosphonium salts generated in situ (one-pot method) toward aromatic compounds or heteronucleophiles is well documented [6,24]. Therefore, we decided to examine the reactivity of the isolated 1-hydroxyalkylphosphonium salts (step-by-step procedure); however, some in-house results for one-pot reactions are also presented for comparison (see Table 5, Scheme 3, and Table S2, Supporting Information). To this end, we used the reaction with (hetero)aromatic systems or amide-type substrates (amides/carbamates/lactams). 

Both transformations required an elevated temperature of 120 °C and 50 °C, respectively. 

In the case of the less activated aromatic systems (e.g., 1,3-dimethoxybenzene), their excess (1:2 or 1:5) had a positive effect on the reaction yields but made the work-up procedure more difficult (sometimes one crystallization was not enough). Our research showed that the one-pot methodology (without the isolation of 1-hydroxyalkylphosphonium salts) is slightly more efficient (Table 5, entries 1, 4, and 5).

For more reactive substrates (1,3,5-trimethoxybenzene and indole), the stoichiometric ratio (1:1) is optimal, as it ensures high yields and an easier purification of the crude product (see Scheme 3). In special cases, e.g., when aqueous solutions of aldehydes are used (see chloroacetaldehyde, Scheme 3/compound **7d**), the step-by-step procedure gives much better results. Water does not affect the formation of 1-hydroxyalkylphosphonium salts, but it hinders reactions with arenes. All in all, both methods allowed the synthesis of 1-arylalkylphosphonium salts **7a**–**g** (R^1^ = alkyl) at good to very good yields, and such compounds had not been synthesized in this way before. The only option for the synthesis of compounds **7h**–**l** is the one-pot protocol because the isolation of the expected 1-hydroxyalkylphosphonium salt is not possible.

In comparison to the one-pot procedure proposed by Masarwa et al. (PPh_3_:TfOH:**2**:(Het)ArH = 1.1:1.2:1:1, CH_3_CN, 45–80 °C, 15–24 h) [6], we used a different ratio of substrates (the lack of excess acid is crucial here; its excess can catalyze the reaction). Therefore, we had to increase the reaction temperature, but its duration was shortened, and the product purification became easier (simple crystallization, without chromatography). Furthermore, the use of Ar_3_P·HX seems to be safer and more convenient (solid and less aggressive reagents).

**Scheme 4:**
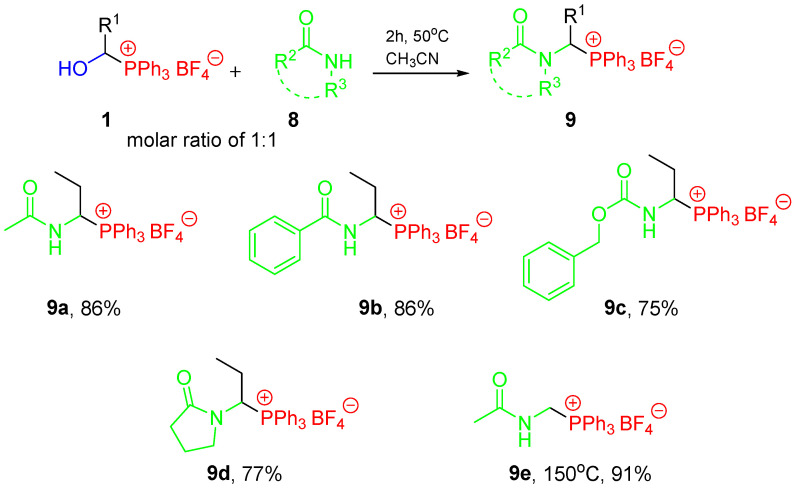
Conditions and results for the reaction of 1-hydroxyalkylphosphonium salts **1** with amide-type substrates.

For reactions with amide-type substrates, we used analogous conditions to those in the case of the one-pot procedure we recently described [24]. Transformations occurred similarly, with good yields. It is easy to notice that hydroxymethylphosphonium salts **1** (R^1^ = H) are less reactive compared to phosphonium salts with a longer alkyl chain (e.g., R^1^ = Et), which also is in agreement with the observations made previously (Scheme 4, see also Table S2, Supporting Information) [24].

**Scheme 5:**
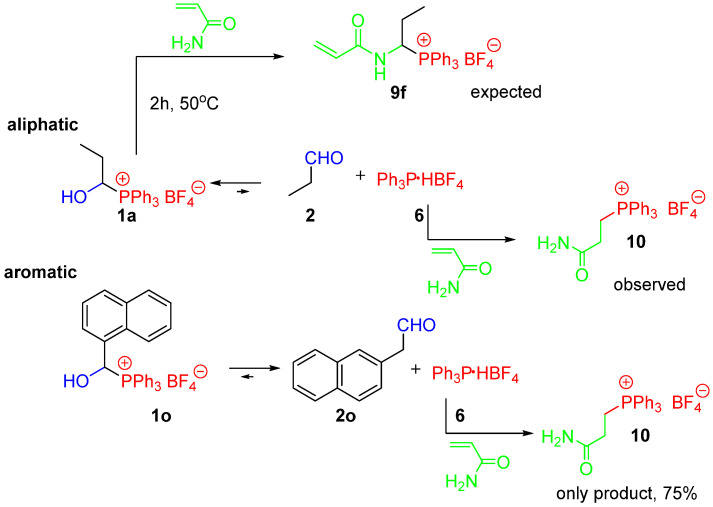
The reaction of 1-hydroxyalkylphosphonium salts **1a** and **1o** with acrylamide—possible pathways.

Interestingly, the conducted research provided further evidence for the presence of equilibrium in the examined systems. The reactions of phosphonium salt **1a** with acrylamide did not lead to the expected 1-(*N*-acylamino)alkylphosphonium salt **9f**, but rather a complex mixture in which we identified the phosphonium salt **10** as one of the main products (about 30%). In turn, the phosphonium salt **1o** (generated in situ) reacts under the same conditions to give **10** as the only reaction product. This can be explained by the course of the reaction proposed in Scheme 5. The addition to the double bond appears to occur more readily, causing the equilibrium to be shifted, limiting the formation of the desired product **9f**. The 1-aryl-1-hydroxyalkylphosphonium salts (e.g., **1o**) are much less stable, which means that there is more Ph_3_P·HBF_4_ in the system and thus the addition to the double bond is even more privileged.

## 3. Materials and Methods

### 3.1. General Methods

Melting points were determined in capillaries and were uncorrected. The ^1^H- and ^13^C-NMR spectra were recorded at operating frequencies of 400 and 100 MHz, respectively, using TMS (tetramethylsilane) as the internal resonance shift standard. The ^31^P-NMR spectra were recorded at an operating frequency of 161.9 MHz, with respect to H_3_PO_4_ at zero ppm. All chemical shifts (δ) are reported in ppm and coupling constants (J) in Hz. Infrared (IR) spectra were measured on a Fourier transform (FT)-IR spectrophotometer (Attenuated Total Reflectance—ATR method). High-resolution mass spectrometry (HR-MS) analyses were performed using a Waters Xevo G2 Q-TOF mass spectrometer equipped with an ESI source operating in positive ion mode. The accurate mass and composition of the molecular ion adducts were calculated using the MassLynx 4.1 software incorporated within the instrument. Solvents (ACS grade) were stored over molecular sieves prior to use. All other commercially available reagents were used as received, without further purification or modifications.

### 3.2. Synthesis of 1-Hydroxyalkylphosphonium Salts

To an aldehyde (1.0 mmol) and a solvent (1 cm^3^) placed in a glass vial sealed with a screw-cap, triarylphosphonium salt HP^+^Ar_3_ X^−^ (1 mmol) was added. The reaction was carried out under the conditions given in Table 1 and 2. Then, 1-hydroxyalkylphosphonium salts **1** were precipitated using Et_2_O. The crude 1-hydroxyalkylphosphonium salts **1** can also be obtained only via the evaporation of volatile components.

### 3.3. Synthesis of 1-Hydroxyalkylphosphonium Salts on a Gram-Scale

The reaction was carried out in a 25 cm^3^ round-bottom flask equipped with a reflux condenser. Triphenylphosphonium bromide (2.74 g, 8 mmol) was added to a solution of paraformaldehyde (0.24 g, 8 mmol) in CH_3_CN (8 cm^3^). Then, the obtained mixture was stirred vigorously and heated at 50 °C for 2 h using an oil bath. After cooling to room temperature, the product was precipitated with diethyl ether (10 cm^3^), separated via vacuum filtration, washed on a Büchner funnel with CH_3_CN/Et_2_O (10 cm^3^, 1:3 [*v*/*v*]), and dried to afford pure hydroxymethyltriphenylphosphonium bromide **1h** at an 87% yield.

### 3.4. Synthesis of 1-Hydroxyalkylphosphonium Salts in the Presence of PPh_3_ and HBF_4_·Et_2_O

To a solution of propionaldehyde (67 µL, 58.1 mg, 1.0 mmol) in CH_3_CN (1 cm^3^) placed in a glass vial sealed with a screw-cap, PPh_3_ (262.2 mg, 1.0 mmol) and HBF_4_·Et_2_O (136 µL, 161.9 mg, 1.0 mmol) were added. The reaction was carried out under the conditions given in Table 1. Then, 1-hydroxyalkylphosphonium salt **1a** was precipitated using Et_2_O.

*1-hydroxypropyltriphenylphosphonium tetrafluoroborate* (**1a**). White crystals (379.6 mg, 93% yield), mp 125–127 °C. ^1^H NMR (400 MHz, CDCl_3_) δ 7.83–7.62 (m, 15H, 3×Ph), 5.64 (dd, *J* = 10.2, 3.0 Hz, 1H, C_α_H), 5.31 (br s, 1H, OH), 1.91–1.80 (m, 2H, CH_2_), and 1.20 (td, *J* = 7.2, 0.9 Hz, 3H, CH_3_); ^13^C{^1^H} NMR (100 MHz, CDCl_3_) δ aromatic carbons: 135.2, 134.3 (d, *J* = 8.5 Hz), 130.5 (d, *J* = 12.0 Hz), 117.2 (d, *J* = 81.1 Hz), 69.9 (d, *J* = 60.9 Hz, C_α_H), 25.7 (d, *J* = 4.9 Hz, CH_2_), and 10.3 (d, *J* = 14.4 Hz, CH_3_); ^31^P{^1^H} NMR (161.9 MHz, CDCl_3_) δ 21.5 ppm; IR (ATR) 3407, 1438, 1110, 1088, 1066, 996, and 976 cm^−1^. HRMS (TOF-ESI) calcd. for C_21_H_22_OP^+^ [M^+^] 321.1408, found 321.1422.
*1-hydroxypropyltriphenylphosphonium bromide* (**1b**) [24]. White crystals (353.1 mg, 88% yield), mp 150–152 °C (lit.: mp 157–159 °C [24]). ^1^H NMR (400 MHz, CDCl_3_) δ 7.95–7.71 (m, 9H, Ph), 7.70–7.58 (m, 6H, Ph), 5.96–5.88 (m, 1H, C_α_H), 1.91–1.77 (m, 2H, CH_2_), and 1.24 (td, *J* = 7.2, 1.1 Hz, 3H, CH_3_); ^13^C{^1^H} NMR (100 MHz, CDCl_3_) δ aromatic carbons: 134.9 (d, *J* = 3.0 Hz), 134.4 (d, *J* = 9.0 Hz), 130.3 (d, *J* = 12.1 Hz), 117.7 (d, *J* = 80.6 Hz), 68.4 (d, *J* = 60.4 Hz, C_α_H), 25.7 (d, *J* = 6.2 Hz, CH_2_), and 10.7 (d, *J* = 14.5 Hz, CH_3_); ^31^P{^1^H} NMR (161.9 MHz, CDCl_3_) δ 20.9 ppm; IR (ATR) 3072, 1438, 1111, 754 cm^−1^.
*1-hydroxypropyltriphenylphosphonium triflate* (**1c**). White crystals (371.6 mg, 79% yield), mp 132–134 °C. ^1^H NMR (400 MHz, CDCl_3_) δ 7.84–7.70 (m, 9H, Ph), 7.70–7.62 (m, 6H, Ph), 5.76–5.67 (m, 1H, C_α_H), 1.91–1.76 (m, 2H, CH_2_), and 1.21 (td, *J* = 7.4, 1.2 Hz, 3H, CH_3_); ^13^C{^1^H} NMR (100 MHz, CDCl_3_) δ aromatic carbons: 135.1 (d, *J* = 3.0 Hz), 134.4 (d, *J* = 8.9 Hz), 130.5 (d, *J* = 12.0 Hz), 120.7 (q, *J* = 319.6 Hz, CF_3_), 117.5 (d, *J* = 80.7 Hz), 69.7 (d, *J* = 60.4 Hz, C_α_H), 25.8 (d, *J* = 5.4 Hz, CH_2_), and 10.4 (d, *J* = 14.7 Hz, CH_3_); ^31^P{^1^H} NMR (161,9 MHz, CDCl_3_) δ 21.0 ppm; IR (ATR) 3266, 1438, 1292, 1244, 1224, 1155, 1109, and 1028 cm^−1^. HRMS (TOF-ESI) calcd. For C_21_H_22_OP^+^ [M^+^] 321.1408, found 321.1407.
*1-hydroxypropyltris(4-methoxyphenyl)phosphonium tetrafluoroborate* (**1d**). Resin (328.8 mg, 66% yield). ^1^H NMR (400 MHz, CDCl_3_) δ 7.70–7.53 (m, 6H, aromatic), 7.20–7.07 (m, 6H, aromatic), 5.42 (dd, *J* = 10.5, 3.0 Hz, 1H, C_α_H), 3.90 (s, 9H, 3xOCH_3_), 1.87–1.68 (m, 2H, CH_2_), and 1.17 (td, *J* = 7.2, 0.4 Hz, 3H, CH_3_); ^13^C{^1^H} NMR (100 MHz, CDCl_3_) δ aromatic carbons: 164.7 (d, *J* = 3.0 Hz), 136.1 (d, *J* = 10.5 Hz), 116.1 (d, *J* = 13.2 Hz), 108.0 (d, *J* = 89.2 Hz), 70.1 (d, *J* = 65.2 Hz, C_α_H), 55.9 (OCH_3_), 25.4 (CH_2_), and 10.3 (d, *J* = 14.3 Hz, CH_3_); ^31^P{^1^H} NMR (161.9 MHz, CDCl_3_) δ 19.9 ppm; IR (ATR) 3442, 1593, 1503, 1263, 1110, 1055, and 1016 cm^−1^. HRMS (TOF-ESI) calcd. for C_24_H_28_O_4_P^+^ [M^+^] 411.1725, found 411.1721.
*hydroxymethyltriphenylphosphonium tetrafluoroborate* (**1f**) [16,17]. White crystals (361.1 mg, 95% yield), mp 132–134 °C C (lit.: mp 128–130 °C [16], 130–131 °C [17]). ^1^H NMR (400 MHz, CDCl_3_) δ 7.85–7.64 (m, 15H, 3xPh), 5.47 (s, 2H, CH_2_); ^13^C{^1^H} NMR (100 MHz, CDCl_3_) δ aromatic carbons: 135.4 (d, *J* = 3.0 Hz), 134.1 (d, *J* = 9.5 Hz), 130.5 (d, *J* = 12.3 Hz), 117.3 (d, *J* = 83.9 Hz), and 57.5 (d, *J* = 65.2 Hz, CH_2_); ^31^P{^1^H} NMR (161.9 MHz, CDCl_3_) δ 17.1 ppm; IR (ATR) 3073, 1439, 1076, 1025, and 998 cm^−1^. HRMS (TOF-ESI) calcd. for C_19_H_18_OP^+^ [M^+^] 293.1095, found 293.1085.
*hydroxymethyltriphenylphosphonium triflate* (**1g**) [28]. White crystals (411.4 mg, 93% yield), mp 134–135 °C. ^1^H NMR (400 MHz, CDCl_3_) δ 7.89–7.78 (m, 3H, Ph), 7.75–7.63 (m, 12H, Ph), 5.41 (s, 2H, CH_2_); ^13^C{^1^H} NMR (100 MHz, CDCl_3_) δ aromatic carbons: 135.5 (d, *J* = 3.0 Hz), 134.0 (d, *J* = 9.5 Hz), 130.6 (d, *J* = 12.4 Hz), 120.6 (q, *J* = 319.6 Hz, CF_3_), 116.7 (d, *J* = 84.0 Hz), and 58.1 (d, *J* = 65.1 Hz, CH_2_); ^31^P{^1^H} NMR (161.9 MHz, CDCl_3_) δ 17.2 ppm; IR (ATR) 3313, 1438, 1281, 1249, 1228, 1158, 1113, and 1027 cm^−1^. HRMS (TOF-ESI) calcd. for C_19_H_18_OP^+^ [M^+^] 293.1095, found 293.1096.
*hydroxymethyltriphenylphosphonium bromide* (**1h**) [29]. White crystals (354.5 mg, 95% yield), mp 191–193 °C (lit.: mp 203 °C [29]). ^1^H NMR (400 MHz, CDCl_3_) δ 7.85–7.61 (m, 15H, 3xPh), 5.47 (s, 2H, CH_2_); ^13^C{^1^H} NMR (100 MHz, CDCl_3_) δ aromatic carbons: 135.4 (d, *J* = 3.0 Hz), 134.1 (d, *J* = 9.5 Hz), 130.5 (d, *J* = 12.3 Hz), 117.3 (d, *J* = 83.6 Hz), and 57.6 (d, *J* = 65.1 Hz, CH_2_); ^31^P{^1^H} NMR (161.9 MHz, CDCl_3_) δ 17.1 ppm; IR (ATR) 3087, 1435, 1115, and 1051 cm^−1^. HRMS (TOF-ESI) calcd. For C_19_H_18_OP^+^ [M^+^] 293.1095, found 293.1096.
*1-hydroxyethyltriphenylphosphonium tetrafluoroborate* (**1i**). White crystals (390.3 mg, 99% yield), mp 114–116 °C. ^1^H NMR (400 MHz, CDCl_3_) δ 7.90–7.55 (m, 15H, 3xPh), 5.96–5.86 (m, 1H, C_α_H), 5.29 (dd, *J* = 13.1, 6.8 Hz, 1H, OH), and 1.70 (dd, *J* = 18.2, 6.8 Hz, 3H, CH_3_); ^13^C{^1^H} NMR (150 MHz, CDCl_3_) δ aromatic carbons: 135.3 (br s), 134.2 (d, *J* = 8.9 Hz), 130.5 (d, *J* = 12.1 Hz), 116.8 (d, *J* = 82.1 Hz), 65.4 (d, *J* = 65.1 Hz, C_α_H), and 18.5 (br s, CH_3_); ^31^P{^1^H} NMR (161.9 MHz, CDCl_3_) δ 17.5 ppm; IR (ATR) 3451, 1488, 1439, 1108, 1060, 996, and 976 cm^−1^. HRMS (TOF-ESI) calcd. for C_20_H_20_OP^+^ [M^+^] 307.1252, found 307.1251.
*2-chloro-1-hydroxyethyltriphenylphosphonium tetrafluoroborate* (**1j**). White crystals (369.0 mg, 89% yield), mp 111.5–113.5 °C. ^1^H NMR (400 MHz, CDCl_3_) δ 7.90–7.74 (m, 9H, Ph), 7.70–7.59 (m, 6H, Ph), 6.11 (br s, 1H, OH), 5.73 (dd, *J* = 13.2, 6.2 Hz, 1H, C_α_H), 4.03 (ddd, *J* = 19.9, 12.8, 4.3 Hz, 1H, C*H*H), and 3.88 (ddd, *J* = 12.9, 8.2, 5.0 Hz, 1H, CH*H*); ^13^C{^1^H} NMR (100 MHz, CDCl_3_) δ aromatic carbons: 135.6 (d, *J* = 3.1 Hz), 134.3 (d, *J* = 9.5 Hz), 130.6 (d, *J* = 12.5 Hz), 116.5 (d, *J* = 82.7 Hz), 69.8 (d, *J* = 67.3 Hz, C_α_H), and 44.4 (d, *J* = 7.3 Hz, CH_2_); ^31^P{^1^H} NMR (161.9 MHz, CDCl_3_) δ 20.8 ppm; IR (ATR) 3337, 1586, 1483, 1436, 1061, 1023, 995, and 973 cm^−1^. HRMS (TOF-ESI) calcd. for C_20_H_19_ClOP^+^ [M^+^] 341.0862, found 341.0861.
*1-hydroxydecyltriphenylphosphonium tetrafluoroborate* (**1k**). Resin (501.3 mg, 99% yield). ^1^H NMR (400 MHz, CDCl_3_) δ 7.87–7.60 (m, 15H, 3xPh), 5.73–5.66 (m, 1H, C_α_H), 5.29 (br s, 1H, OH), aliphatics (8xCH_2_): 1.90–1.66 (m, 3H), 1.66–1.46 (m, 1H), 1.35–1.11 (m, 12H), and 0.85 (t, *J* = 6.9 Hz, 3H, CH_3_); ^13^C{^1^H} NMR (100 MHz, CDCl_3_) δ aromatic carbons: 135.2 (d, *J* = 3.0 Hz), 134.3 (d, *J* = 8.9 Hz), 130.5 (d, *J* = 12.0 Hz), 117.2 (d, *J* = 80.9 Hz), 68.9 (d, *J* = 60.1 Hz, C_α_H), 32.3 (d, *J* = 4.3 Hz, CH_2_), 31.9 (CH_2_), 29.5 (CH_2_), 29.4 (CH_2_), 29.3 (CH_2_), 29.2 (CH_2_), 25.7 (d, *J* = 13.4 Hz, CH_2_), 22.7 (br s, CH_2_), and 14.2 (CH_3_); ^31^P{^1^H} NMR (161.9 MHz, CDCl_3_) δ 21.4 ppm; IR (ATR) 2923, 2854, 1439, 1109, 1056, and 996 cm^−1^. HRMS (TOF-ESI) calcd. for C_28_H_36_OP^+^ [M^+^] 419.2504, found 419.2499.
*1,3-dihydroxypropane-1,3-bis(triphenylphosphonium) bis(tetrafluoroborate)* (**1l**). White crystals (108.1 mg, 14% yield), mp 150–151 °C. ^1^H NMR (400 MHz, CDCl_3_) δ 7.90–7.50 (m, 30H, 6xPh), 6.64 (br s, 2H, OH), 5.94 (br s, 2H, C_α_H), and 2.18–2.05 (m, 2H, CH_2_); ^13^C{^1^H} NMR (100 MHz, CDCl_3_) δ aromatic carbons: 135.4 (br s), 134.7–134.3 (m), 130.9–130.5 (m), 116.1 (d, *J* = 84.2 Hz), 62.7 (dd, *J* = 73.2, 13.8 Hz, C_α_H), and 33.5 (t, *J* = 8.7 Hz, CH_2_); ^31^P{^1^H} NMR (161.9 MHz, CDCl_3_) δ 22.7 ppm; IR (ATR) 2989, 1586, 1483, 1437, 1109, 1045, and 995 cm^−1^. HRMS (TOF-ESI) calcd. for C_39_H_36_O_2_P_2_^2+^ [M^2+^] 299.1090, found 299.1087.
*1-hydroxy-3-phenylpropane-1,3-bis(triphenylphosphonium) bis(tetrafluoroborate)* (**1m**). White crystals (815.8 mg, 98% yield), mp 205–207 °C. ^1^H NMR (400 MHz, CDCl_3_) δ 7.83–7.71 (m, 6H, Ph), 7.69–7.54 (m, 18H, Ph), 7.47–7.39 (m, 6H, Ph), 7.39–7.30 (m, 1H, Ph), 7.27–7.21 (m, 2H, Ph), 7.06–7.00 (m, 2H, Ph), 6.16 (t, *J* = 7.1 Hz, 1H, OH), 5.24–5.16 (m, 1H, CH-Ph or C_α_H), 5.04 (dd, *J* = 17.1, 10.8 Hz, 1H, CH-Ph or C_α_H), 2.81–2.69 (m, 1H, C*H*H), and 2.59–2.50 (m, 1H, CH*H*); ^13^C{^1^H} NMR (100 MHz, CDCl_3_) δ aromatic carbons: 135.6 (d, *J* = 3.0 Hz), 135.5 (d, *J* = 3.1 Hz), 134.4 (d, *J* = 9.4 Hz), 134.1 (d, *J* = 9.3 Hz), 131.3 (d, *J* = 5.8 Hz), 130.7 (d, *J* = 12.4 Hz), 130.6 (d, *J* = 12.5 Hz), 130.1 (d, *J* = 3.0 Hz), 129.9 (d, *J* = 2.2 Hz), 129.4 (d, *J* = 5.1 Hz), 116.1 (d, *J* = 83.9 Hz), 115.4 (d, *J* = 82.6 Hz), 66.5 (dd, *J* = 66.2, 14.8 Hz, C_α_H), 40.2 (dd, *J* = 47.7, 15.6 Hz, CH-Ph), and 35.2 (br d, *J* = 10.2 Hz, CH_2_); ^31^P{^1^H} NMR (161.9 MHz, CDCl_3_) δ 26.9 (d, *J* = 9.1 Hz), 21.8 (d, *J* = 9.1 Hz) ppm; IR (ATR) 3410, 1439, 1108, 1050, and 996 cm^−1^. HRMS (TOF-ESI) calcd. for C_39_H_36_O_2_P_2_^2+^ [M^2+^] 329.1277, found 329.1265.
*1-hydroxy-3-phenylpropane-1,3-bis(triphenylphosphonium) bis(triflate)* (**1n**). White crystals (108.1 mg, 14% yield), mp 190–192 °C. ^1^H NMR (400 MHz, CDCl_3_) δ 7.84–7.70 (m, 6H, Ph), 7.69–7.56 (m, 18H, Ph), 7.49–7.41 (m, 6H, Ph), 7.39–7.33 (m, 1H, Ph), 7.27–7.22 (m, 2H, Ph), 7.05–6.99 (m, 2H, Ph), 5.18–5.02 (m, 2H, CH-Ph and C_α_H), 2.76–2.64 (m, 1H, C*H*H), 2.59–and 2.46 (m, 1H, CH*H*); ^13^C{^1^H} NMR (100 MHz, CDCl_3_) δ aromatic carbons: 135.6 (d, *J* = 3.0 Hz), 135.4 (d, *J* = 3.1 Hz), 134.6 (d, *J* = 9.4 Hz), 134.3 (d, *J* = 9.3 Hz), 131.4 (d, *J* = 5.8 Hz), 130.7 (d, *J* = 12.4 Hz), 130.7 (d, *J* = 12.4 Hz), 130.2 (d, *J* = 3.0 Hz), 129.9 (d, *J* = 2.3 Hz), 129.5 (d, *J* = 5.1 Hz), 120.6 (q, *J* = 320.2 Hz, CF_3_), 116.3 (d, *J* = 83.9 Hz), 115.8 (d, *J* = 82.5 Hz), 66.3 (dd, *J* = 66.8, 14.8 Hz, C_α_H), 40.0 (dd, *J* = 47.7, 15.7 Hz, CH-Ph), and 35.2 (dd, *J* = 9.8, 1.0 Hz, CH_2_); ^31^P{^1^H} NMR (161.9 MHz, CDCl_3_) δ 26.4 (d, *J* = 8.7 Hz), 21.4 (d, *J* = 8.7 Hz) ppm; IR (ATR) 3062, 1439, 1262, 1107, 1025, and 997 cm^−1^. HRMS (TOF-ESI) calcd. For C_39_H_36_O_2_P_2_^2+^ [M^2+^] 329.1277, found 329.1270.

### 3.5. NMR Experiments (Studies of Equilibria)

A mixture of an aldehyde (0.1 mmol), a triarylphosphonium salt Ar_3_P∙HX (0.1 mmol), and a deuterated solvent (0.65 cm^3^) was placed in an NMR tube. The reaction was carried out under the conditions given in Table 3 and Table 4. Changes in substrate and/or product concentrations were monitored via ^1^H NMR and confirmed via ^31^P{^1^H} NMR spectroscopy (for ^31^P{^1^H} NMR: seqfil = s2pul, sw = 249 ppm, at = 0.813 s, np = 65,536, pw = 3.356 µs, d1 = 1 s, and offset = 16,377.3 Hz).

### 3.6. Reactions of 1-Hydroxyalkylphosphonium Salts with (hetero)arenes (Step-by-Step)

To a solution of 1-hydroxyalkylphosphonium salt (1.0 mmol) in CH_3_CN (1 cm^3^) placed in a glass vial sealed with a screw-cap, a (hetero)aromatic compound (5, 2, or 1 mmol) was added. The reaction was carried out under the conditions given in Scheme 3. Then, the 1-arylalkylphosphonium salts **7** were precipitated using Et_2_O.

### 3.7. Reactions of Aldehydes with (hetero)arenes in the Presence of Ar_3_P∙HX (One-Pot)

To a solution of aldehydes (1.0 mmol) in CH_3_CN (1.0 cm^3^) placed in a glass vial sealed with a screw-cap, the Ar_3_P∙HX (1.0 mmol) and a (hetero)aromatic compound (5.0 mmol or 1.0 mmol) were added. The reaction was carried out under the conditions given in Table 5 and Scheme 3. Then, the 1-arylalkylphosphonium salts **7** were precipitated using Et_2_O.

### 3.8. Reactions of Aldehydes with (hetero)arenes in the Presence of PPh_3_ and HBF_4_·Et_2_O (One-Pot)

To a solution of propionaldehyde (67 µL, 58.1 mg, 1.0 mmol) in CH_3_CN (1.0 cm^3^) placed in a glass vial sealed with a screw-cap, PPh_3_ (262.2 mg, 1.0 mmol), HBF_4_·Et_2_O (136 µL, 161.9 mg, 1.0 mmol), and 1,3-dimethoxybenzene (648.6 µL, 690.8 mg, 5 mmol) were added. The reaction was carried out under the conditions given in Table 5. Then, the 1-arylalkylphosphonium salt **7a** was precipitated using Et_2_O.

*1-(2,4-dimethoxyphenyl)propyltriphenylphosphonium tetrafluoroborate* (**7a**). White crystals (433.2 mg, 82%), mp 174–176 °C. ^1^H NMR (400 MHz, CDCl_3_) δ 7.88–7.79 (m, 3H, Ph), 7.73–7.63 (m, 6H, Ph), 7.51–7.40 (m, 6H, Ph), 6.58 (dd, *J* = 9.3, 2.4 Hz, 1H, aromatic), 6.44–6.33 (m, 2H, aromatic), 4.90 (ddd, *J* = 15.1, 12.2, 2.6 Hz, 1H, C_α_H), 3.82 (s, 3H, OCH_3_), 3.43 (s, 3H, OCH_3_), 2.35–2.20 (m, 1H, C*H*H), 2.19–2.08 (m, 1H, CH*H*), and 0.95 (td, *J* = 7.1, 1.2 Hz, 3H, CH_3_) ppm; ^13^C{^1^H} NMR (100 MHz, CDCl_3_) δ aromatic carbons: 161.9 (d, *J* = 3.0 Hz), 159.4 (d, *J* = 5.9 Hz), 135.4 (d, *J* = 3.0 Hz), 134.3 (d, *J* = 9.0 Hz), 130.5 (d, *J* = 12.1 Hz), 117.7 (d, *J* = 82.4 Hz), 110.5 (d, *J* = 5.2 Hz), 105.8 (d, *J* = 2.6 Hz), 98.9 (d, *J* = 2.4 Hz), 55.7 (OCH_3_), 55.5 (OCH_3_), 36.7 (d, *J* = 41.6 Hz, C_α_H), 24.2 (CH_2_), and 12.5 (d, *J* = 15.3 Hz, CH_3_) ppm; ^31^P{^1^H} NMR (161.9 MHz, CDCl_3_) δ 23.2 ppm; IR (ATR) 2971, 1608, 1585, 1508, 1438, 1107, 1050, 1027, and 997 cm^−1^. HRMS (TOF-ESI) calcd. for C_29_H_30_O_2_P^+^ [M^+^] 441.1983 found 441.1982.
*1-(2,4,6-trimethoxyphenyl)propyltriphenylphosphonium triflate* (**7b**). Resin (533.7 mg, 86%). ^1^H NMR (400 MHz, CDCl_3_) 7.88–7.75 (m, 3H, Ph), 7.71–7.59 (m, 6H, Ph), 7.44–7.31 (m, 6H, Ph), 6.03 (s, 2H, aromatic), 5.00 (ddd, *J* = 17.5, 11.9, 3.2 Hz, 1H, C_α_H), 3.84 (s, 3H, OCH_3_), 3.46 (s, 3H, OCH_3_), 3.15 (s, 3H, OCH_3_), 2.77–2.62 (m, 1H, C*H*H), 2.13–2.00 (m, 1H, CH*H*), and 0.91 (td, *J* = 7.2, 1.1 Hz, 3H, CH_3_) ppm; ^13^C{^1^H} NMR (100 MHz, CDCl_3_) δ aromatic carbons: 162.7 (d, *J* = 2.8 Hz), 160.0 (d, *J* = 7.5 Hz), 135.0 (d, *J* = 3.0 Hz), 134.0 (d, *J* = 8.9 Hz), 130.1 (d, *J* = 12.0 Hz), 121.0 (q, *J* = 320.7 Hz, CF_3_), 118.8 (d, *J* = 82.4 Hz), 98.6 (d, *J* = 5.3 Hz), 91.0, 56.1 (OCH_3_), 55.7 (OCH_3_), 54.7 (OCH_3_), 36.4 (d, *J* = 45.5 Hz, C_α_H), and 21.6 (CH_2_), 13.0 (d, *J* = 15.1 Hz, CH_3_) ppm; ^31^P{^1^H} NMR (161.9 MHz, CDCl_3_) δ 21.7 ppm; IR (ATR) 2940, 1609, 1586, 1438, 1266, 1222, 1141, 1119, 1103, and 1030 cm^−1^. HRMS (TOF-ESI) calcd. for C_30_H_32_O_3_P^+^ [M^+^] 471.2089 found 471.2093.
*1-(H-indol-3-yl)propyltriphenylphosphonium triflate* (**7c**). White crystals (472.8 mg, 83%), mp 191–193 °C. ^1^H NMR (400 MHz, CDCl_3_) 10.22 (s, 1H, NH), 7.77–7.69 (m, 3H, Ph), 7.61–7.53 (m, 6H, Ph), 7.51–7.43 (m, 7H, aromatic), 7.07–6.99 (m, 1H, aromatic), 6.97–6.91 (m, 1H, aromatic), 6.81 (br t, *J* = 7.5 Hz, 1H, aromatic), 6.65 (br t, *J* = 3.0 Hz, 1H, aromatic), 4.73 (ddd, *J* = 14.4, 12.2, 2.4 Hz, 1H, C_α_H), 2.40–2.21 (m, 1H, C*H*H), 2.15–2.01 (m, 1H, CH*H*), and 0.91 (td, *J* = 7.1, 1.3 Hz, 3H, CH_3_) ppm; ^13^C{^1^H} NMR (100 MHz, CDCl_3_) δ aromatic carbons: 136.2, 135.2 (d, *J* = 2.0 Hz), 134.3 (d, *J* = 8.9 Hz), 130.3 (d, *J* = 12.0 Hz), 127.4 (d, *J* = 4.6 Hz), 126.3 (d, *J* = 7.8 Hz), 122.4, 121.0 (q, *J* = 318.8 Hz, CF_3_), 119.9, 117.8 (d, *J* = 80.9 Hz), 117.7, 112.9, 103.0 (d, *J* = 5.8 Hz), 36.8 (d, *J* = 45.2 Hz, C_α_H), 25.6 (CH_2_), and 12.4 (d, *J* = 14.9 Hz, CH_3_) ppm; ^31^P{^1^H} NMR (161.9 MHz, CDCl_3_) δ 21.9 ppm; IR (ATR) 3329, 1438, 1277, 1266, 1248, 1225, 1158, 1109, and 1030 cm^−1^. HRMS (TOF-ESI) calcd. for C_29_H_27_NP^+^ [M^+^] 420.1881 found 420.1882.
*2-chloro-1-(2,4,6-trimethoxyphenyl)ethyltriphenylphosphonium tetrafluoroborate* (**7d**). White crystals (457.3 mg, 79%), mp 159–160 °C. ^1^H NMR (400 MHz, CDCl_3_) 7.86–7.79 (m, 3H, Ph), 7.72–7.64 (m, 6H, Ph), 7.49–7.41 (m, 6H, Ph), 6.03 (s, 2H, aromatic), 5.50 (ddd, *J* = 16.4, 7.8, 7.1 Hz, 1H, C_α_H), 4.45–4.27 (m, 2H, CH_2_Cl), 3.83 (s, 3H, OCH_3_), and 3.42 (s, 6H, OCH_3_) ppm; ^13^C{^1^H} NMR (100 MHz, CDCl_3_) δ aromatic carbons: 163.5 (d, *J* = 2.7 Hz), 159.5 (br s), 135.4 (d, *J* = 3.1 Hz), 134.2 (d, *J* = 9.4 Hz), 130.3 (d, *J* = 12.4 Hz), 117.8 (d, *J* = 83.1 Hz), 98.3 (d, *J* = 5.1 Hz), 91.2, 55.9 (OCH_3_), 55.5 (OCH_3_), 41.7 (d, *J* = 5.0 Hz, CH_2_Cl), and 38.5 (d, *J* = 45.6 Hz, C_α_H) ppm; ^31^P{^1^H} NMR (161.9 MHz, CDCl_3_) δ 22.1 ppm; IR (ATR) 3354, 2843, 1608, 1590, 1438, 1340, 1209, 1157, 1142, 1118, 1103, 1032, and 997 cm^−1^. HRMS (TOF-ESI) calcd. for C_29_H_29_ClO_3_P^+^ [M^+^] 491.1543 found 491.1546.
*1-(2,4-dimethoxyphenyl)ethyltriphenylphosphonium tetrafluoroborate* (**7e**) [30]. Resin (385.7 mg, 75%). ^1^H NMR (400 MHz, CDCl_3_) δ 7.93–7.80 (m, 3H, Ph), 7.73–7.60 (m, 6H, Ph), 7.53–7.42 (m, 6H, Ph), 6.69–6.58 (m, 1H, aromatic), 6.42–6.32 (m, 2H, aromatic), 5.27–5.14 (m, 1H, C_α_H), 3.81 (s, 3H, OCH_3_), 3.41 (s, 3H, OCH_3_), and 1.82 (dd, *J* = 18.5, 7.4 Hz, 3H, CH_3_) ppm; ^13^C{^1^H} NMR (100 MHz, CDCl_3_) δ aromatic carbons: 161.8 (d, *J* = 3.0 Hz), 158.1 (d, *J* = 5.6 Hz), 135.2 (d, *J* = 3.0 Hz), 134.2 (d, *J* = 8.9 Hz), 130.3 (d, *J* = 12.1 Hz), 130.1 (d, *J* = 5.0 Hz), 117.3 (d, *J* = 82.4 Hz), 112.6 (d, *J* = 5.4 Hz), 105.5 (d, *J* = 2.6 Hz), 98.7 (d, *J* = 2.5 Hz), 55.6 (OCH_3_), 55.2 (OCH_3_), 29.7 (d, *J* = 45.7 Hz, C_α_H), and 16.5 (CH_3_) ppm; δ ^31^P{^1^H} NMR (161.9 MHz, CDCl_3_) δ 24.2 ppm; IR (ATR) 2936, 1608, 1506, 1486, 1438, 1301, 1209, 1107, 1049, 1023, and 996 cm^−1^. The spectra reported here are in agreement with previously published data [30].
*1-(2,4,6-trimethoxyphenyl)decyltriphenylphosphonium tetrafluoroborate* (**7f**). White crystals (564.6 mg, 86%), mp 110–112 °C. ^1^H NMR (400 MHz, CDCl_3_) 7.88–7.76 (m, 3H, Ph), 7.70–7.60 (m, 6H, Ph), 7.44–7.31 (m, 6H, Ph), 6.06 (s, 1H, aromatic), 6.03 (s, 1H, aromatic), 5.08 (ddd, *J* = 17.7, 12.0, 3.0 Hz, 1H, C_α_H), 3.85 (s, 3H, OCH_3_), 3.45 (s, 3H, OCH_3_), 3.16 (s, 3H, OCH_3_), and aliphatics (8xCH_2_): 2.81–2.64 (m, 1H), 1.99–1.81 (m, 1H), 1.32–1.09 (m, 14H), and 0.85 (t, *J* = 7.0 Hz, 3H, CH_3_) ppm; ^13^C{^1^H} NMR (100 MHz, CDCl_3_) δ aromatic carbons: 162.7 (d, *J* = 2.9 Hz), 160.1 (d, *J* = 3.6 Hz), 159.8 (d, *J* = 6.5 Hz), 135.0 (d, *J* = 3.0 Hz), 134.1 (d, *J* = 8.9 Hz), 130.2 (d, *J* = 12.1 Hz), 118.8 (d, *J* = 82.3 Hz), 98.8 (d, *J* = 5.4 Hz), 91.1 (br s), 56.1 (OCH_3_), 55.8 (OCH_3_), 54.7 (OCH_3_), 34.8 (d, *J* = 45.3 Hz, C_α_H), 31.9 (CH_2_), 29.5 (CH_2_), 29.3 (CH_2_), 29.3 (CH_2_), 28.8 (CH_2_), 28.1 (d, *J* = 13.6 Hz), 27.7 (CH_2_), 22.7 (CH_2_), and 14.2 (CH_3_) ppm; ^31^P{^1^H} NMR (161.9 MHz, CDCl_3_) δ 21.7 ppm; IR (ATR) 2925, 2853, 1607, 1589, 1456, 1437, 1206, 1147, 1127, 1101, 1046, 1033, and 996 cm^−1^. HRMS (TOF-ESI) calcd. for C_37_H_46_O_3_P^+^ [M^+^] 569.3185 found 569.3185.
*1-(H-indol-3-yl)decyltriphenylphosphonium tetrafluoroborate* (**7g**). Creamy crystals (551.0 mg, 91%), mp 195–197 °C. ^1^H NMR (400 MHz, CDCl_3_) 9.70 (s, 1H, NH), 7.82–7.70 (m, 3H, Ph), 7.65–7.53 (m, 6H, Ph), 7.52–7.40 (m, 7H, aromatic), 7.12–7.01 (m, 1H, aromatic), 6.91–6.78 (m, 2H, aromatic), 6.77–6.68 (m, 1H, aromatic), 4.81–4.66 (m, 1H, C_α_H), and aliphatics (8xCH_2_): 2.27–2.01 (m, 2H), 1.42–0.98 (m, 14H), and 0.82 (t, *J* = 7.1 Hz, 3H, CH_3_) ppm; ^13^C{^1^H} NMR (100 MHz, CDCl_3_) δ aromatic carbons: 136.2, 135.3 (d, *J* = 2.9 Hz), 134.3 (d, *J* = 8.9 Hz), 130.4 (d, *J* = 12.0 Hz), 127.2 (d, *J* = 4.4 Hz), 126.6 (d, *J* = 6.9 Hz), 122.6, 120.1, 117.9 (d, *J* = 82.0 Hz), 117.5, 113.0, 103.4 (d, *J* = 5.9 Hz), 35.4 (d, *J* = 44.8 Hz, C_α_H), 32.0 (br s, CH_2_), 31.9 (CH_2_), 29.5 (CH_2_), 29.3 (CH_2_), 29.2 (CH_2_), 29.1 (CH_2_), 27.6 (d, *J* = 13.6 Hz), 22.7 (CH_2_), and 14.2 (CH_3_) ppm; ^31^P{^1^H} NMR (161.9 MHz, CDCl_3_) δ 21.9 ppm; IR (ATR) 3357, 2927, 2854, 1484, 1459, 1436, 1340, 1106, 1055, 1020, 995, 744, 720, and 691 cm^−1^. HRMS (TOF-ESI) calcd. for C_36_H_41_NP^+^ [M^+^] 518.2977 found 518.2982.
*1-(2,4-dimethoxyphenyl)phenylmethyltriphenylphosphonium tetrafluoroborate* (**7h**) [30]. White crystals (357.4 mg, 62%), mp 209–211 °C (lit.: mp 199.5–200.5 °C [30]). ^1^H NMR (400 MHz, CDCl_3_) δ 7.84–7.75 (m, 3H), 7.67–7.58 (m, 8H), 7.51–7.40 (m, 6H), 7.32–7.19 (m, 1H), 7.14–7.07 (m, 2H), 7.02–6.95 (m, 1H), 6.52 (d, *J* = 18.2 Hz, 1H, C_α_H), 6.44–6.40 (m, 2H), 3.80 (s, 3H, OCH_3_), and 3.62 (s, 3H, OCH_3_) ppm; ^13^C{^1^H} NMR (100 MHz, CDCl_3_) δ aromatic carbons: 161.8 (d, *J* = 2.1 Hz), 157.7 (d, *J* = 6.5 Hz), 135.1 (d, *J* = 3.0 Hz), 134.5 (d, *J* = 9.0 Hz), 132.5 (d, *J* = 3.1 Hz), 131.6 (d, *J* = 6.1 Hz), 130.4 (d, *J* = 6.2 Hz), 130.1 (d, *J* = 12.2 Hz), 129.2 (d, *J* = 1.9 Hz), 129.0 (d, *J* = 2.8 Hz), 118.3 (d, *J* = 82.4 Hz), 112.8 (d, *J* = 3.5 Hz), 105.5 (d, *J* = 1.6 Hz), 99.1 (d, *J* = 1.6 Hz), 55.6 (OCH_3_), 55.5 (OCH_3_), and 41.8 (d, *J* = 45.5 Hz, C_α_H) ppm; ^31^P{^1^H} NMR (161.9 MHz, CDCl_3_) δ 22.0 ppm; IR (ATR) 3058, 1614, 1582, 1505, 1438, 1215, and 1035 cm^−1^. The spectra reported here are in agreement with previously published data [30].
*1-(naphthalen-1-yl)-1-(2,4,6-trimethoxyphenyl)methyltriphenylphosphonium tetrafluoroborate* (**7i**). Resin (485.8 mg, 74%). ^1^H NMR (400 MHz, CDCl_3_) 7.90–7.76 (m, 3H, aromatic), 7.75–7.62 (m, 4H, aromatic), 7.60–7.39 (m, 13H, aromatic), 7.40–7.27 (m, 2H, aromatic), 7.19 (d, *J* = 19.4 Hz, 1H, C_α_H), 6.08 (s, 2H, aromatic), 3.81 (s, 3H, OCH_3_), and 3.44 (s, 6H, OCH_3_) ppm; ^13^C{^1^H} NMR (100 MHz, CDCl_3_) δ aromatic carbons: 162.8 (d, *J* = 1.8 Hz), 158.6 (d, *J* = 5.5 Hz), 134.7 (d, *J* = 3.1 Hz), 134.5 (d, *J* = 8.9 Hz), 134.0, 131.4 (d, *J* = 8.1 Hz), 130.8 (d, *J* = 6.4 Hz), 129.9 (d, *J* = 12.1 Hz), 129.8, 129.3 (d, *J* = 1.9 Hz), 129.3, 127.1, 126.1, 124.8 (d, *J* = 2.1 Hz), 122.7, 120.3 (d, *J* = 82.5 Hz), 102.1 (d, *J* = 3.6 Hz), 91.5 (d, *J* = 1.3 Hz), 55.8 (OCH_3_), 55.4 (OCH_3_), and 37.7 (d, *J* = 49.6 Hz, C_α_H) ppm; ^31^P{^1^H} NMR (161.9 MHz, CDCl_3_) δ 22.4 ppm; IR (ATR) 2940, 1604, 1587, 1458, 1207, 1149, 1097, 1049, 996, and 690 cm^−1^. HRMS (TOF-ESI) calcd. for C_38_H_34_O_3_P^+^ [M^+^] 569.2246 found 569.2252.
*1-(H-indol-3-yl)-1-(naphthalen-1-yl)methyltriphenylphosphonium tetrafluoroborate* (**7j**). Pink crystals (454.1 mg, 75%), mp 115–117 °C. ^1^H NMR (400 MHz, CDCl_3_) 9.81 (s, 1H, NH), 8.07–7.99 (m, 1H, aromatic), 7.87–7.75 (m, 2H, aromatic), 7.73–7.58 (m, 3H, aromatic), 7.54–7.35 (m, 15H, aromatic), 7.33–7.19 (m, 2H, aromatic), 7.07–6.95 (m, 3H, aromatic), 6.96 (d, *J* = 17.2 Hz, 1H, C_α_H), and 6.87–6.73 (m, 1H, aromatic) ppm; ^13^C{^1^H} NMR (100 MHz, CDCl_3_) δ aromatic carbons: 136.1, 135.3 (d, *J* = 3.0 Hz), 134.8 (d, *J* = 8.9 Hz), 134.2, 131.2 (d, *J* = 8.1 Hz), 130.3 (d, *J* = 12.0 Hz), 130.1 (d, *J* = 2.1 Hz), 130.0 (d, *J* = 1.8 Hz), 129.5, 128.5 (d, *J* = 7.1 Hz), 128.4 (d, *J* = 5.5 Hz), 127.8, 126.7, 126.0 (d, *J* = 6.4 Hz), 125.1 (d, *J* = 1.9 Hz), 122.6, 122.2, 120.2, 118.5 (d, *J* = 80.9 Hz), 117.5, 113.0, 104.9 (d, *J* = 4.8 Hz), and 38.4 (d, *J* = 47.1 Hz, C_α_H) ppm; ^31^P{^1^H} NMR (161.9 MHz, CDCl_3_) δ 19.6 ppm; IR (ATR) 3356, 3059, 1461, 1023, 997, 738, 719, and 690 cm^−1^. HRMS (TOF-ESI) calcd. for C_37_H_29_NP^+^ [M^+^] 518.2038 found 518.2036.
*1-(tiophen-2-yl)-1-(2,4,6-trimethoxyphenyl)methyltriphenylphosphonium* (**7k**). White crystals (575.7 mg, 94%), mp 195–197 °C (decomposition). ^1^H NMR (400 MHz, CDCl_3_) 7.83–7.76 (m, 3H, Ph), 7.66–7.58 (m, 6H, Ph), 7.39–7.29 (m, 6H, Ph), 7.24–7.19 (m, 1H, aromatic), 6.90–6.84 (m, 1H, aromatic), 6.84 (d, *J* = 18.7 Hz, 1H, C_α_H), 6.70 (br t, *J* = 3.5 Hz, 1H), 6.13 (s, 2H, aromatic), 3.86 (s, 3H, OCH_3_), and 3.48 (br s, 6H, OCH_3_) ppm; ^13^C{^1^H} NMR (100 MHz, CDCl_3_) δ aromatic carbons: 163.2 (d, *J* = 2.1 Hz), 158.9 (br s), 135.1 (d, *J* = 3.0 Hz), 134.3 (d, *J* = 8.8 Hz), 134.1 (d, *J* = 8.8 Hz), 130.2 (d, *J* = 12.2 Hz), 129.8 (d, *J* = 6.7 Hz), 127.4 (d, *J* = 4.1 Hz), 127.1 (d, *J* = 3.2 Hz), 119.0 (d, *J* = 82.5 Hz), 101.8 (d, *J* = 3.7 Hz), 91.2, 55.9 (OCH_3_), 55.5 (OCH_3_), and 37.8 (d, *J* = 49.2 Hz, C_α_H) ppm; ^31^P{^1^H} NMR (161.9 MHz, CDCl_3_) δ 21.9 ppm; IR (ATR) 3111, 2947, 1608, 1596, 1435, 1421, 1220, 1158, 1100, 1049, and 997 cm^−1^. HRMS (TOF-ESI) calcd. for C_32_H_30_O_3_PS^+^ [M^+^] 525.1653 found 525.1655.
*1-(H-indol-3-yl)-1-(tiophen-2-yl)methyltriphenylphosphonium tetrafluoroborate* (**7l**). Pink crystals (477.2 mg, 85%), mp 185–187 °C. ^1^H NMR (400 MHz, CDCl_3_) 9.87 (s, 1H, NH), 7.81–7.71 (m, 3H, Ph), 7.60–7.51 (m, 7H, aromatic), 7.40–7.30 (m, 6H, Ph), 7.21–7.15 (m, 2H, aromatic), 7.15–7.08 (m, 1H, aromatic), 6.95–6.87 (m, 2H, aromatic), 6.86–6.82 (m, 1H, aromatic), 6.78–6.72 (m, 1H, aromatic), and 6.45 (d, *J* = 16.4 Hz, 1H, C_α_H) ppm; ^13^C{^1^H} NMR (100 MHz, CDCl_3_) δ aromatic carbons: 135.9, 135.6 (d, *J* = 2.9 Hz), 134.8 (d, *J* = 8.9 Hz), 130.4 (d, *J* = 12.1 Hz), 127.8 (d, *J* = 4.0 Hz), 127.7 (d, *J* = 3.2 Hz), 127.5 (d, *J* = 2.9 Hz), 127.4 (d, *J* = 2.7 Hz), 126.4 (d, *J* = 6.4 Hz), 123.1, 120.5, 117.5 (d, *J* = 82.0 Hz), 117.5, 113.2, 104.4 (d, *J* = 0.9 Hz), 104.4 (d, *J* = 1.7 Hz), and 38.4 (d, *J* = 47.5 Hz, C_α_H) ppm; ^31^P{^1^H} NMR (161.9 MHz, CDCl_3_) δ 19.9 ppm; IR (ATR) 3362, 3060, 1437, 1343, 1107, 1055, 996, and 688 cm^−1^. HRMS (TOF-ESI) calcd. for C_31_H_25_NPS^+^ [M^+^] 474.1445 found 474.1437.

### 3.9. Reactions of 1-Hydroxyalkylphosphonium Salts with Amide-Type Substrates

To a solution of 1-hydroxyalkylphosphonium salt (1.0 mmol) in CH_3_CN (1 cm^3^) placed in a glass vial sealed with a screw-cap, an amide-type substrate (1 mmol) was added. The reaction was carried out under the conditions given in Scheme 4. Then, 1-(*N*-acylamino)alkylphosphonium salts **9** were precipitated using Et_2_O.

*1-(N-acetylamino)propyltriphenylphosphonium tetrafluoroborate* (**9a**) [24]. White crystals (386.3 mg, 86%), mp 199–201 °C (lit.: mp 185–186 °C [24]). ^1^H NMR (400 MHz, CDCl_3_) δ 7.87–7.76 (m, 4H, Ph + NH), 7.74–7.66 (m, 12H, Ph), 5.67 (dddd, *J* = 12.0, 9.2, 7.7, 2.7 Hz, 1H, C_α_H), 2.13–1.97 (m, 1H, C*H*H), 1.91 (d, *J* = 1.2 Hz, 3H, CH_3_C=O), 1.86–1.70 (m, 1H, CH*H*), and 1.12 (td, *J* = 7.2, 1.3 Hz, 3H, CH_3_) ppm; ^13^C{^1^H} NMR (100 MHz, CDCl_3_) δ 172.6 (d, *J* = 3.0 Hz, C=O), aromatic carbons: 135.4 (d, *J* = 3.0 Hz), 134.3 (d, *J* = 9.3 Hz), 130.6 (d, *J* = 12.3 Hz), 117.2 (d, *J* = 81.7 Hz), 49.6 (d, *J* = 53.4 Hz, C_α_H), 25.2 (d, *J* = 5.4 Hz, CH_2_), 22.3 (CH_3_C=O), and 11.5 (d, *J* = 14.1 Hz, CH_3_) ppm; ^31^P{^1^H} NMR (161.9 MHz, CDCl_3_) δ 26.3 ppm; IR (ATR) 3330, 1683, 1522, 1440, 1286, 1109, 1062, 1020, and 996 cm^−1^. The spectra reported here are in agreement with previously published data [24].
*1-(N-benzoylamino)propyltriphenylphosphonium tetrafluoroborate* (**9b**) [24]. White crystals (383.5 mg, 75%), mp 197–198 °C (lit.: mp 198–199 °C [24]). ^1^H NMR (400 MHz, CDCl_3_) δ 8.39 (dd, *J* = 8.2, 2.5 Hz, 1H, NH), 7.83–7.73 (m, 8H, Ph), 7.72–7.68 (m, 3H, Ph), 7.68–7.59 (m, 6H, Ph), 7.48–7.42 (m, 1H, Ph), 7.39–7.33 (m, 2H, Ph), 5.81 (dtd, *J* = 11.5, 8.3, 3.0 Hz, 1H, C_α_H), 2.54–2.37 (m, 1H, C*H*H), 1.89–1.77 (m, 1H, CH*H*), and 1.17 (td, *J* = 7.2, 1.2 Hz, 3H, CH_3_) ppm; ^13^C{^1^H} NMR (100 MHz, CDCl_3_) δ 168.5 (d, *J* = 2.3 Hz, C=O), aromatic carbons: 134.9 (d, *J* = 3.0 Hz), 134.4 (d, *J* = 9.4 Hz), 132.3, 131.7, 130.1 (d, *J* = 12.3 Hz), 128.6, 127.4, 117.9 (d, *J* = 81.7 Hz), 51.4 (d, *J* = 51.7 Hz, C_α_H), 24.9 (d, *J* = 5.2 Hz, CH_2_), and 11.7 (d, *J* = 13.9 Hz, CH_3_) ppm; ^31^P{^1^H} NMR (161.9 MHz, CDCl_3_) δ 27.0 ppm; IR (ATR) 3357, 1670, 1508, 1483, 1438, 1111, 1070, 1030, and 996 cm^−1^. The spectra reported here are in agreement with previously published data [24].
*1-(N-benzyloxycarbonylamino)propyltriphenylphosphonium tetrafluoroborate* (**9c**) [24]. White crystals (465.5 mg, 86%), mp 176–177 °C (lit.: mp 161–162 °C [24]). ^1^H NMR (400 MHz, CDCl_3_) δ 7.81–7.71 (m, 3H, Ph), 7.70–7.56 (m, 12H, Ph), 7.33–7.24 (m, 3H, Ph), 7.24–7.17 (m, 2H, Ph), 6.93 (d, *J* = 9.1 Hz, 1H, NH), 5.45–5.33 (m, 1H, C_α_H), 4.98, 4.89 (ABq, *J* = 12.5 Hz, 2H, CH_2_), 2.28–2.14 (m, 1H, C*H*H), 1.83–1.70 (m, 1H, CH*H*), and 1.15 (td, *J* = 7.2, 1.3 Hz, 3H, CH_3_) ppm; ^13^C{^1^H} NMR (100 MHz, CDCl_3_) δ 156.9 (d, *J* = 2.9 Hz, C=O), aromatic carbons: 136.1, 135.3 (d, *J* = 2.9 Hz), 134.3 (d, *J* = 9.3 Hz), 130.5 (d, *J* = 12.3 Hz), 128.6, 128.1, 128.0, 117.1 (d, *J* = 81.2 Hz), 67.4 (CH_2_Ph), 52.7 (d, *J* = 52.9 Hz, C_α_H), 25.0 (d, *J* = 6.2 Hz, CH_2_), and 11.5 (d, *J* = 13.9 Hz, CH_3_) ppm; ^31^P{^1^H} NMR (161.9 MHz, CDCl_3_) δ 25.6 ppm; IR (ATR) 3334, 1712, 1519, 1439, 1227, 1110, 1063, 1035, 1009, and 995 cm^−1^. The spectra reported here are in agreement with previously published data [24].
*1-(2-Oxopyrrolidin-1-yl)propyltriphenylphosphonium tetrafluoroborate* (**9d**) [24]. White crystals (366.0 mg, 77%), mp 189–191 °C (lit.: mp 186–188 °C [24]). ^1^H NMR (400 MHz, CDCl_3_) δ 7.91–7.80 (m, 3H, Ph), 7.79–7.65 (m, 12H, Ph), and 5.72 (ddd, *J* = 12.4, 10.5, 3.1 Hz, 1H, C_α_H), CH_2_ groups: 3.57–3.42 (m, 1H), 3.32–3.17 (m, 1H), 2.48–2.28 (m, 1H), 2.27–2.09 (m, 2H), 1.98–1.80 (m, 3H), and 1.07 (td, *J* = 7.2, 1.3 Hz, 3H, CH_3_) ppm; ^13^C{^1^H} NMR (100 MHz, CDCl_3_) δ 177.0 (d, *J* = 2.3 Hz, C=O), aromatic carbons: 135.6 (d, *J* = 3.1 Hz), 134.3 (d, *J* = 9.7 Hz), 130.8 (d, *J* = 12.4 Hz), 117.1 (d, *J* = 81.4 Hz), 53.3 (d, *J* = 51.4 Hz, C_α_H), 46.9 (CH_2_N), 30.3 (CH_2_), 22.8 (d, *J* = 5.0 Hz, CH_2_), 18.6 (CH_2_), and 11.5 (d, *J* = 14.1 Hz, CH_3_) ppm; ^31^P{^1^H} NMR (161.9 MHz, CDCl_3_) δ 24.7 ppm; IR (ATR) 2880, 1692, 1509, 1438, 1405, 1271, 1109, 1047, 1035, and 997 cm^−1^. The spectra reported here are in agreement with previously published data [24].
*(N-Acetylamino)methyltriphenylphosphonium tetrafluoroborate* (**9e**) [24,31]. White crystals (383.3 mg, 91%), mp 191–193 °C (lit.: mp 191–193 °C [24]). ^1^H NMR (400 MHz, CDCl_3_) δ 9.67 (t, *J* = 6.2 Hz, 1H, NH), 7.86–7.76 (m, 9H, Ph), 7.74–7.63 (m, 6H, Ph), 5.13 (dd, *J* = 6.3, 2.9 Hz, 2H, CH_2_), and 1.89 (d, *J* = 1.4 Hz, 3H, CH_3_) ppm; ^13^C{^1^H} NMR (100 MHz, CDCl_3_) δ 172.2 (d, *J* = 1.4 Hz, C=O), aromatic carbons: 135.3 (d, *J* = 3.1 Hz), 134.2 (d, *J* = 9.8 Hz), 130.3 (d, *J* = 12.6 Hz), 117.5 (d, *J* = 83.9 Hz), 37.6 (d, *J* = 56.8 Hz, CH_2_), and 22.6 (CH_3_) ppm; ^31^P{^1^H} NMR (161.9 MHz, CDCl_3_) δ 20.7 ppm; IR (ATR) 3356, 1671, 1508, 1483, 1437, 1111, 1071, 1030, and 996 cm^−1^. The spectra reported here are in agreement with previously published data [24,31].
*2-Carbamoyletyhyltriphenylphosphonium tetrafluoroborate* (**10**) [24]. White crystals (315.9 mg, 75%), mp 145–147 °C (lit.: mp 146–148 °C [24]). ^1^H NMR (400 MHz, CDCl_3_) δ 7.85–7.64 (m, 15H, Ph), 6.98 (br s, 1H, NH), 5.33 (br s, 1H, NH), 3.49–3.41 (m, 2H, CH_2_), and 2.85–2.78 (m, 2H); ^13^C{^1^H} NMR (100 MHz, CDCl_3_) δ 171.3 (d, *J* = 14.2 Hz), 135.4 (d, *J* = 3.0 Hz), 133.5 (d, *J* = 10.0 Hz), 130.7 (d, *J* = 12.7 Hz), 117.5 (d, *J* = 86.7 Hz), 27.3 (d, *J* = 2.9 Hz), and 19.1 (d, *J* = 56.0 Hz); ^31^P{^1^H} NMR (161_,_9 MHz, CDCl_3_) δ 24.6; IR (ATR) 3422, 3198, 1669, 1441, 1025, and 996 cm^−1^. The spectra reported here are in agreement with previously published data [24].

## 4. Conclusions

An efficient and convenient method for the preparation of 1-hydroxyalkylphosphonium salts was optimized and improved. This synthesis is based on the reaction of aldehydes with triarylphosphonium salts in a stoichiometric ratio under mild conditions: r.t., 30 min, without chromatography. The properties of the compounds obtained were determined on the basis of almost 50 examples, which also helped identify factors affecting their stability in solutions. The selected products were isolated and purified by crystallization from CH_3_CN/Et_2_O or CH_2_Cl_2_/Et_2_O and then reacted with aromatic systems (120 °C, 2 h) or amide-type substrates (amide/carbamate/lactam; 50 °C, 2 h). The two protocols, ‘step-by-step’ and ‘one-pot’, have been described and compared with each other. New products such as 1-arylalkylphosphonium salts and 1-(*N*-acylamino)alkylphosphonium salts were formed with good or very good yields.

This confirms the high reactivity of 1-hydroxyalkylphosphonium salts, which are not necessarily generated in situ.

## Figures and Tables

**Table 1:** Synthesis of 1-hydroxyalkylphosphonium salts **1**—optimization of the reaction conditions.

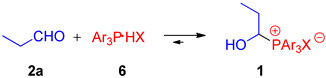							
Entry	Phosphonium Salts 1			Time, min	Temp., °C	Solvent	Yield, %
	No.	Ar	X				
1	**1a**	Ph	BF_4_	30	r.t.	MeCN	99 ^a^/92/85 ^b^
2	**1a**	Ph	BF_4_	30	r.t.	CH_2_Cl_2_	93
3	**1a**	Ph	BF_4_	30	r.t.	THF	^c^
4	**1b**	Ph	Br	30	r.t.	MeCN	88
5	**1c**	Ph	TfO	30	r.t.	MeCN	99 ^a^/79
6	**1d**	3-C_6_H_4_Cl	BF_4_	30	r.t.	MeCN	^d^
7	**1e**	4-C_6_H_4_OMe	BF_4_	30	r.t.	MeCN	99 ^a^/66

^a^ Only after solvent evaporation, no crystallization (crude salt), ^b^ The reaction of aldehyde **2** and PPh_3_ in the presence of HBF_4_ (tetrafluoroboric acid diethyl ether complex, molar ratio of 1:1:1), ^c^ A mixture of products (estimated content of compound **1a** based on ^31^P{^1^H} NMR is 76%), ^d^ A mixture of products (estimated content of compound **1d** based on ^1^H and ^31^P{^1^H} NMR is 31%), crystallization attempts failed.

**Table 2:** Synthesis of 1-hydroxyalkylphosphonium salts **1**—scope of application.

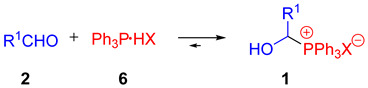							
Entry	Phosphonium Salts 1			Time, min	Temp., °C	Solvent	Yield, %
	No.	R^1^	X				
1	**1f**	H	BF_4_	30	r.t.	CHCl_3_	n.r.
2	**1f**	H	BF_4_	120	50	CHCl_3_	88
3	**1f**	H	BF_4_	120	50	MeCN	95
4	**1g**	H	TfO	120	50	MeCN	93
5	**1h**	H	Br	120	50	MeCN	95/87 ^a^
6	**1i**	Me	BF_4_	30	r.t.	MeCN	49
7	**1i**	Me	BF_4_	30	r.t.	CH_2_Cl_2_	99
8	**1j**	ClCH_2_	BF_4_	30	r.t.	CHCl_3_	89 ^b^
9	**1k**	*n*-C_9_H_19_	BF_4_	30	r.t.	CHCl_3_	99 ^c^
10	**1k**	*n*-C_9_H_19_	BF_4_	30	r.t.	MeCN	^d^

^a^ A gram-scale synthesis (8 mmol), ^b^ A 55% wt solution of chloroacetaldehyde in water was used, ^c^ The salt **1k** was obtained through evaporation of the solvent; crystallization attempts failed, ^d^ Aldehyde does not dissolve in acetonitrile—the reaction does not occur.

**Table 3:** Equilibrium in the reaction of naphthaldehyde **2o** and triarylphosphonium salts **6**.

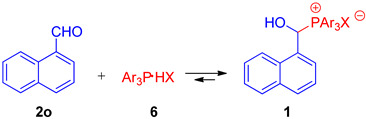								
Entry	No.	Molar Ratio of 2o:6	Ar	X	Time, min	Temp., °C	Solvent	Molar Ratio of 1:2(6) ^a^
1	**1o**	1:1	Ph	BF_4_	5	25	CD_3_CN	57:43
2	**1o**	1:1	Ph	BF_4_	15	25	CD_3_CN	57:43
3	**1o**	1:1	Ph	BF_4_	30	25	CD_3_CN	57:43
4	**1o**	1:1	Ph	BF_4_	5/65	25	CD_3_CN	57:43
5	**1o**	1:1	Ph	BF_4_	25	35	CD_3_CN	48:52
6	**1o**	1:1	Ph	BF_4_	50	45	CD_3_CN	35:65
7	**1p**	1:1	Ph	TfO	5/65	25	CD_3_CN	62:38
8	**1p**	1:1	Ph	TfO	25	35	CD_3_CN	55:45
9	**1p**	1:1	Ph	TfO	50	45	CD_3_CN	44:56
10	**1q**	1:1	4-C_6_H_4_OMe	BF_4_	5/65	25	CD_3_CN	59:41
11	**1q**	1:1	4-C_6_H_4_OMe	BF_4_	25	35	CD_3_CN	48:52
12	**1q**	1:1	4-C_6_H_4_OMe	BF_4_	50	45	CD_3_CN	39:61
13	**1o**	1:1	Ph	BF_4_	5/65	25	CDCl_3_	45:55
14	**1o**	1:1	Ph	BF_4_	25	35	CDCl_3_	35:65
15	**1o**	1:1	Ph	BF_4_	50	45	CDCl_3_	25:75
16	**1o**	1:1	Ph	BF_4_	5/15/30	25	CD_3_CN	57:43
17	**1o**	2:1	Ph	BF_4_	5/15/30	25	CD_3_CN	75:25
18	**1o**	3:1	Ph	BF_4_	5/15/30	25	CD_3_CN	88:12
19	**1o**	5:1	Ph	BF_4_	5/15/30	25	CD_3_CN	94:6

^a^ The composition of the reaction mixture was estimated based on ^1^H and ^31^P{^1^H} NMR (see also Table S1, Supporting Information).

**Table 4:** Equilibrium in the synthesis of 1-hydroxyalkylphosphonium salts **1**.

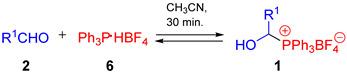					
Entry	Phosphonium Salts 1		Temp., °C	Solvent	Molar Ratio of 1:2(6) ^a^
	No.	R^1^			
1	**1r**	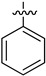	25	CDCl_3_	57:43
				CD_3_CN	82:18
2	**1s**	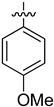	25	CD_3_CN	67:33
3	**1t**	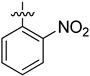	25	CD_3_CN	82:18
4	**1u**	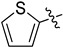	25	CD_3_CN	63:37
5	**1v**	*i*-Pr	25	CD_3_CN	88:12
6	**1v**	*i*-Pr	45	CD_3_CN	72:28
7	**1w**	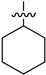	25	CDCl_3_	68:32
8	**1x**	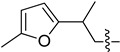	25	CD_3_CN	84:16 ^b^
9	**1y**	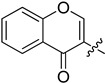	25/50	CD_3_CN	no reaction

^a^ The composition of the reaction mixture was estimated by ^1^H and ^31^P{^1^H} NMR (see also Table S1, Supporting Information), ^b^ A mixture of two diastereomers is formed at the ratio of 1:1.9.

**Table 5:** The reaction of 1-hydroxyalkylphosphonium salts **1** with aromatic compounds—optimization of the conditions (step-by-step vs. one-pot protocol).

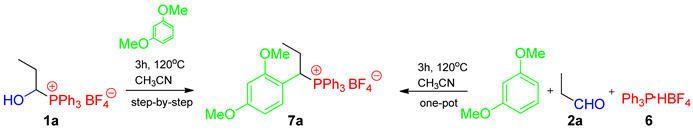				
Entry	Molar Ratio	Procedure	Solvent	Yield, %
1	1:5 (**1a**:ArH)	step-by-step	CD_3_CN	89 ^a^/76 ^b^
2	1:2 (**1a**:ArH)	step-by-step	CD_3_CN	83 ^a^/82 ^b^
3	1:5 (**1a**:ArH)	step-by-step	no solvent	57 ^b^
4	1:1:5 (**2a**:**6**:ArH)	one-pot	CD_3_CN	77 ^b^
5	1:1:1:5 (**2a**:PPh_3_:HBF_4_:ArH) ^c^	one-pot	CD_3_CN	78 ^b^

^a^ The yield was estimated based on ^1^H and ^31^P{^1^H} NMR, ^b^ The isolated yields, ^c^ The reaction of aldehyde **2a,** 1,3-dimethoxybenzene, and PPh_3_ in the presence of HBF_4_ (tetrafluoroboric acid diethyl ether complex).

## Data Availability

The data presented in this study are available on request from the corresponding author.

## Supplementary Materials

The following supporting information can be downloaded at: https://www.mdpi.com/article/10.3390/molecules29010018/s1, Supporting information includes apparatus for the synthesis; ^1^H-, ^13^C-, ^31^P-NMR, IR, and MS spectra of all new compounds; comparison of in-house results with available literature data [24]; Table S1. Characteristic signals (^1^H and ^31^P{^1^H} NMR) for the identification of compounds **1o-x**; Table S2. Comparison of yields in one-pot and step-by-step methodology for the reactions of 1-hydroxyalkylphosphonium salts **1** with the amide-type substrates.
