# Supplementary material for: 1-Hydroxyalkylphosphonium Salts—Synthesis and Properties
Source: Molecules. 2023 Dec 19;29(1):18. doi: 10.3390/molecules29010018 (PMC10780258; doi:10.3390/molecules29010018)
Supplement: Supplementary file 1 [file molecules-29-00018-s001.zip › molecules-2778473-supplementary.pdf]

molecules

Supporting information

1-Hydroxyalkylphosphonium Salts—Synthesis and Properties

Jakub Adamek [1,2,*], Anna Kuźnik [1,2], Agnieszka Październiok-Holewa [1,2], Mirosława Grymel [1,2], Dominika Kozicka [1,2], Dominika Mierzwa [1] and Karol Erfurt [3]

[1] Department of Organic Chemistry, Bioorganic Chemistry and Biotechnology, Silesian University of Technology, B. Krzywoustego 4, 44-100 Gliwice, Poland; anna.kuznik@polsl.pl (A.K.); agnieszka.pazdzierniok@polsl.pl (A.P.-H.); miroslawa.grymel@polsl.pl (M.G.); dominika.kozicka@polsl.pl (D.K.); dominikamierzwaa@gmail.com (D.M.)
[2] Biotechnology Center, Silesian University of Technology, B. Krzywoustego 8, 44-100 Gliwice, Poland
[3] Department of Chemical Organic Technology and Petrochemistry, Faculty of Chemistry, Silesian University of Technology, B. Krzywoustego 4, 44-100 Gliwice, Poland; karol.erfurt@polsl.pl (K.E.)
* Correspondence: jakub.adamek@polsl.pl; Tel.: +48-032-237-1724; Fax: +48-032-237-2094

Supporting information

1. Apparatus for the synthesis……………………………………………………………………S2
2. NMR, IR and MS spectra of compounds **1, 7, 9** ………..…………………….....................S3-S116
3. Characteristic signals (^{1}H and ^{31}P{^{1}H} NMR) for the identification of compounds **1o-x**…….S117
4. Comparison of yields in one-pot and step-by-step methodology for the reactions of 1-hydroxyalkylphosphonium salts **1** with amide-type substrates…………………………..S118

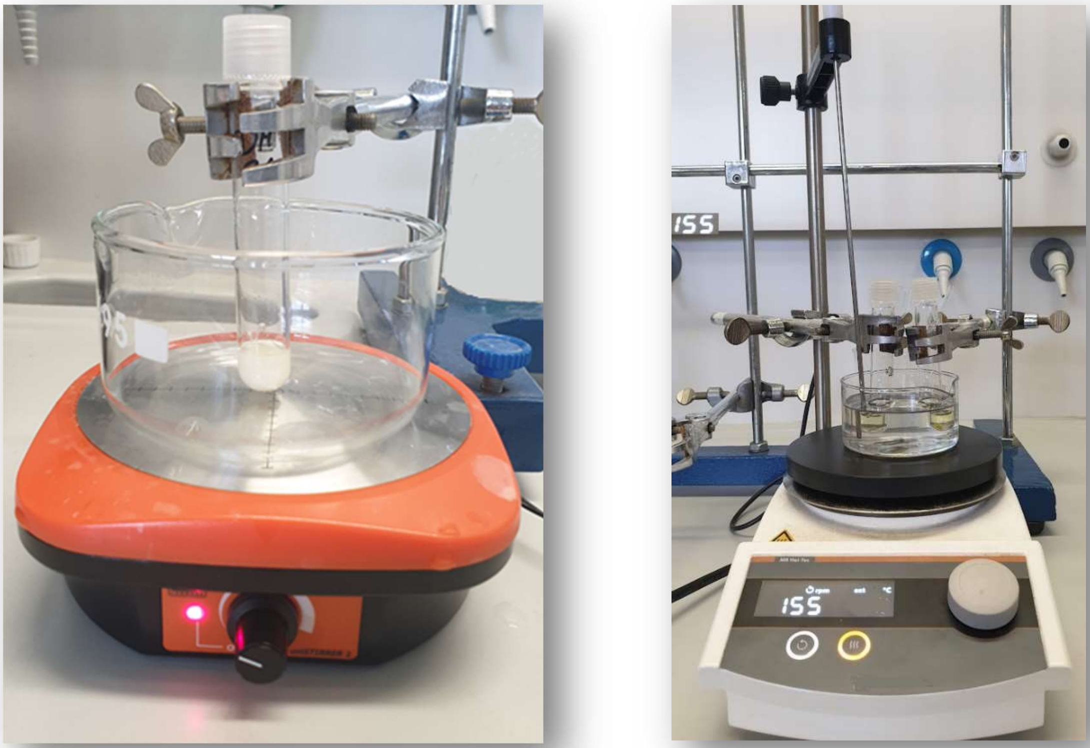

Photo 1. Apparatus for the synthesis of 1-hydroxyalkylphosphonium salts **1** (room temperature or 50°C), 1-arylalkylphosphonium salts **7** (120°C) or 1-(*N*-acylamino)alkylphosphonium salts **9** (50°C or 150°C).

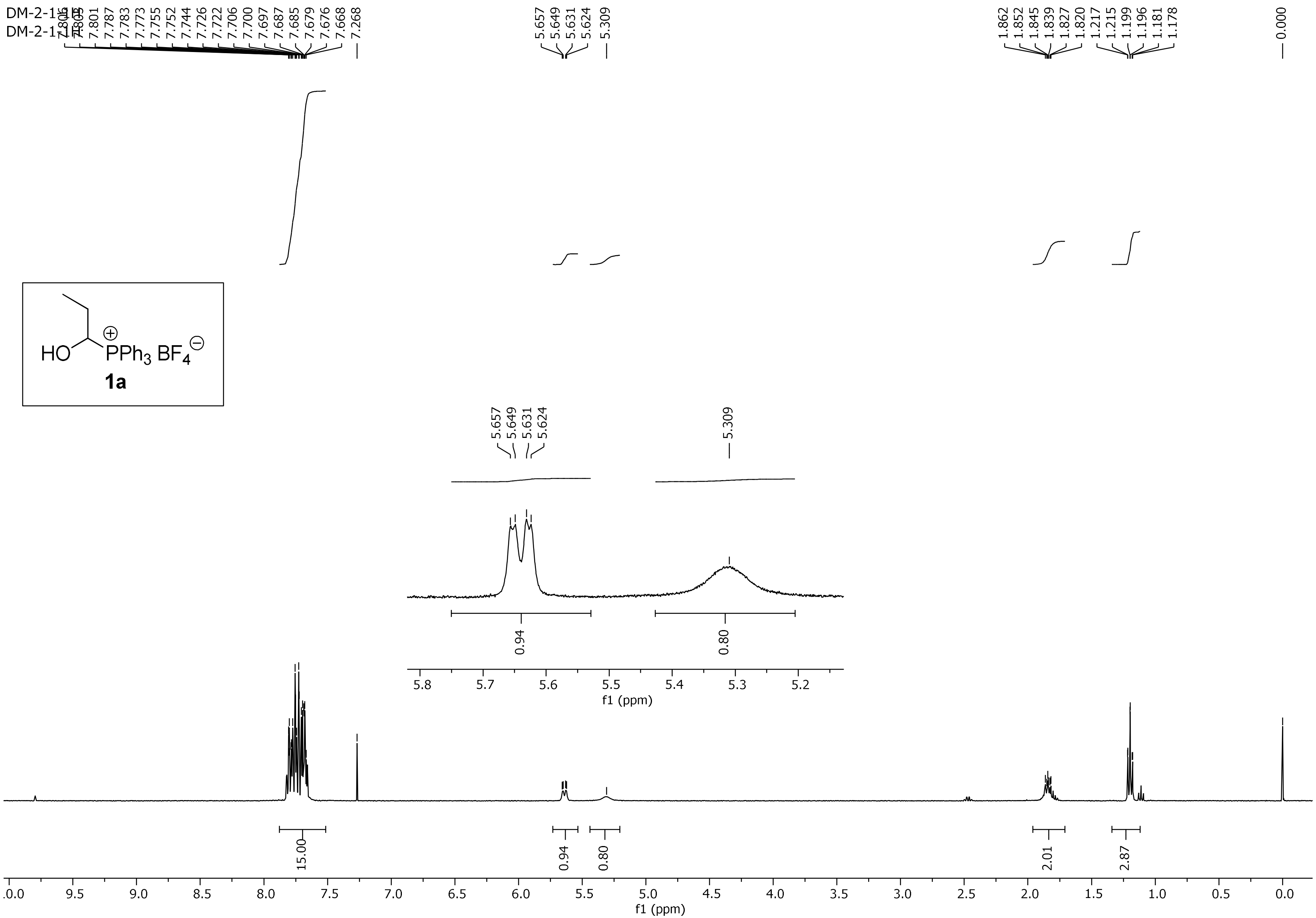

^{1}H NMR spectrum of 1-hydroxypropyltriphenylphosphonium tetrafluoroborate (**1a**); 400 MHz/$CDCl_3$/TMS; δ (ppm).

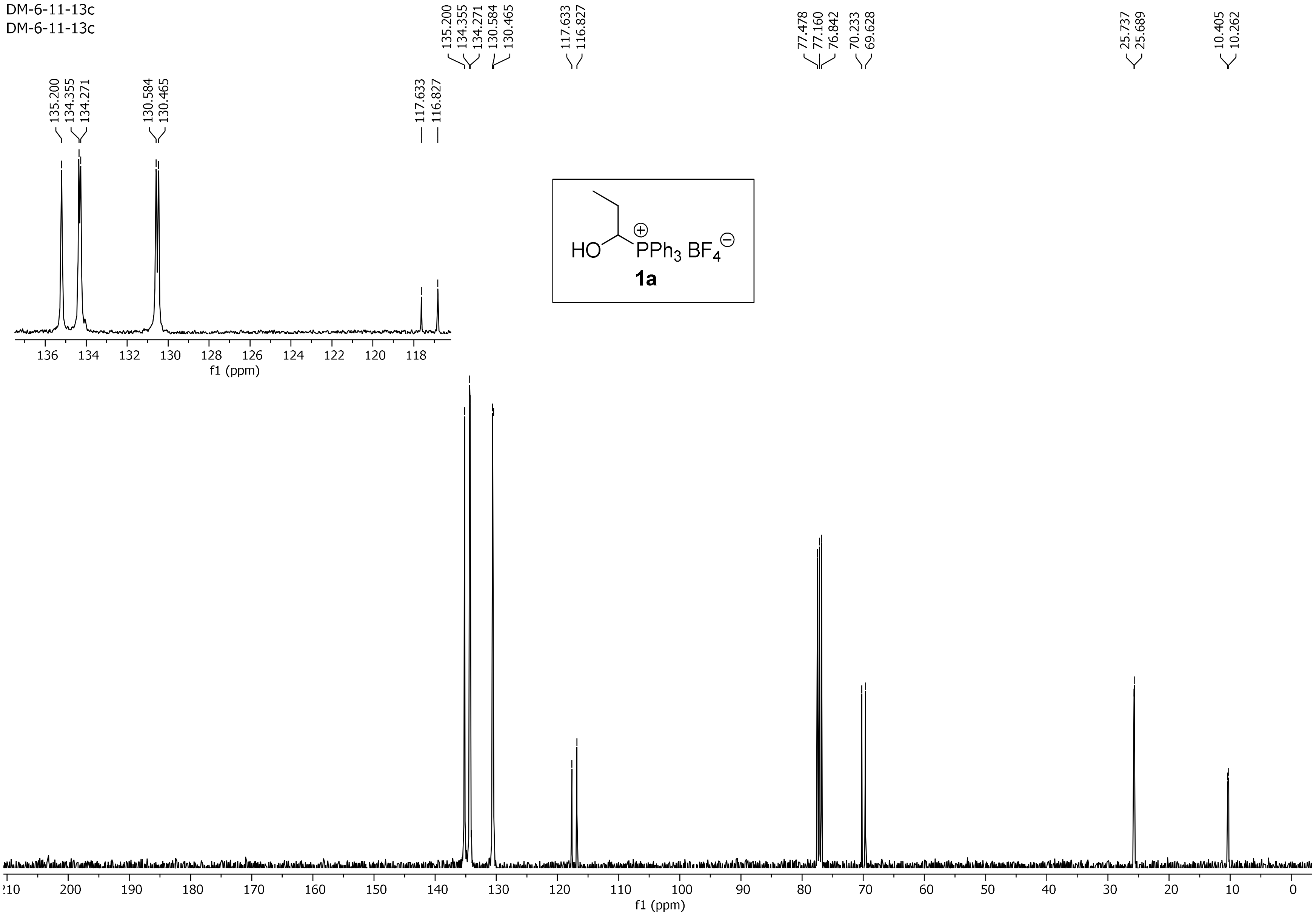

^{13}C NMR spectrum of 1-hydroxypropyltriphenylphosphonium tetrafluoroborate (**1a**); 100 MHz/$CDCl_3$/TMS; δ (ppm).

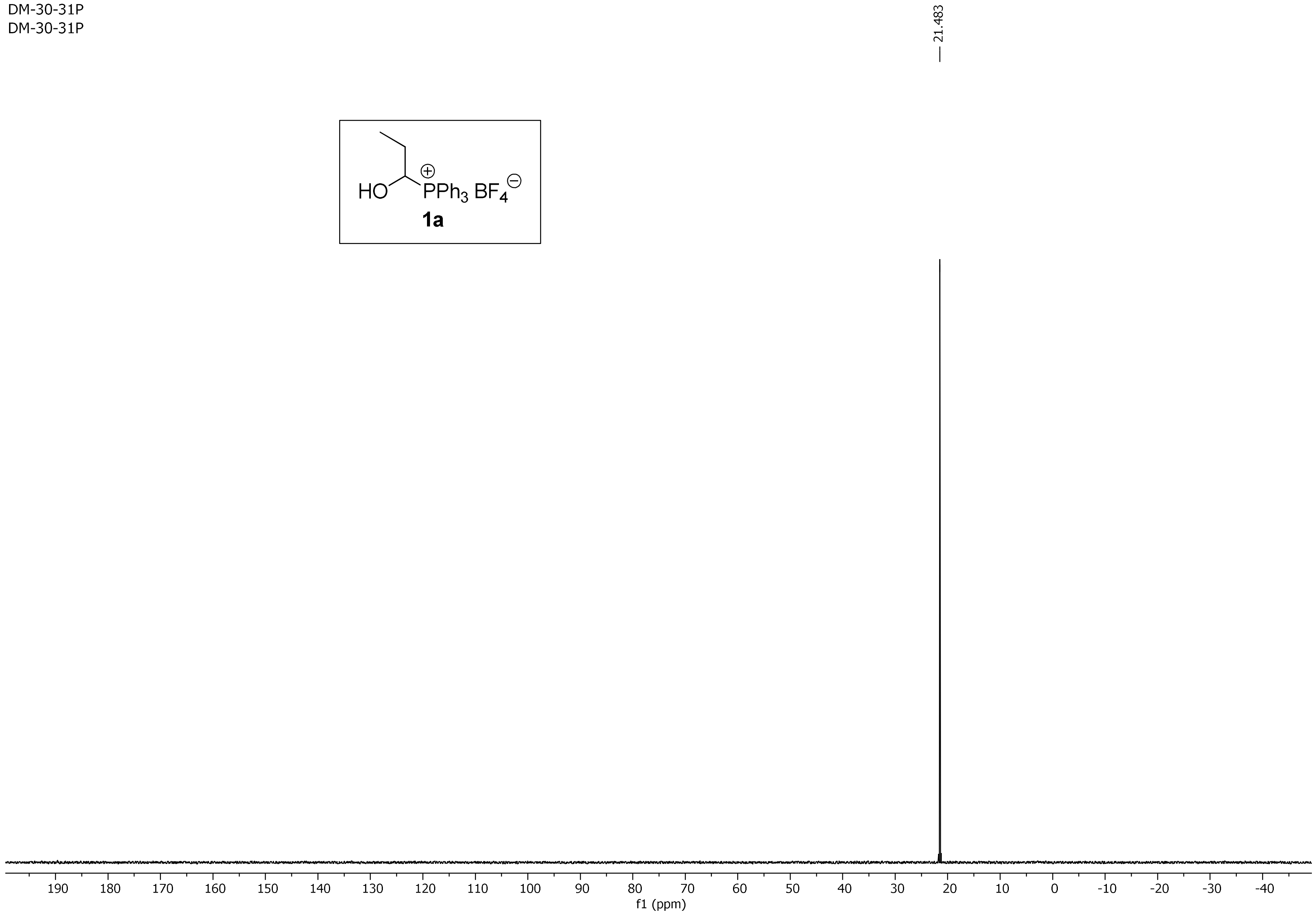

^{31}P NMR spectrum of 1-hydroxypropyltriphenylphosphonium tetrafluoroborate (**1a**); 161.9 MHz/$CDCl_3$; δ (ppm).

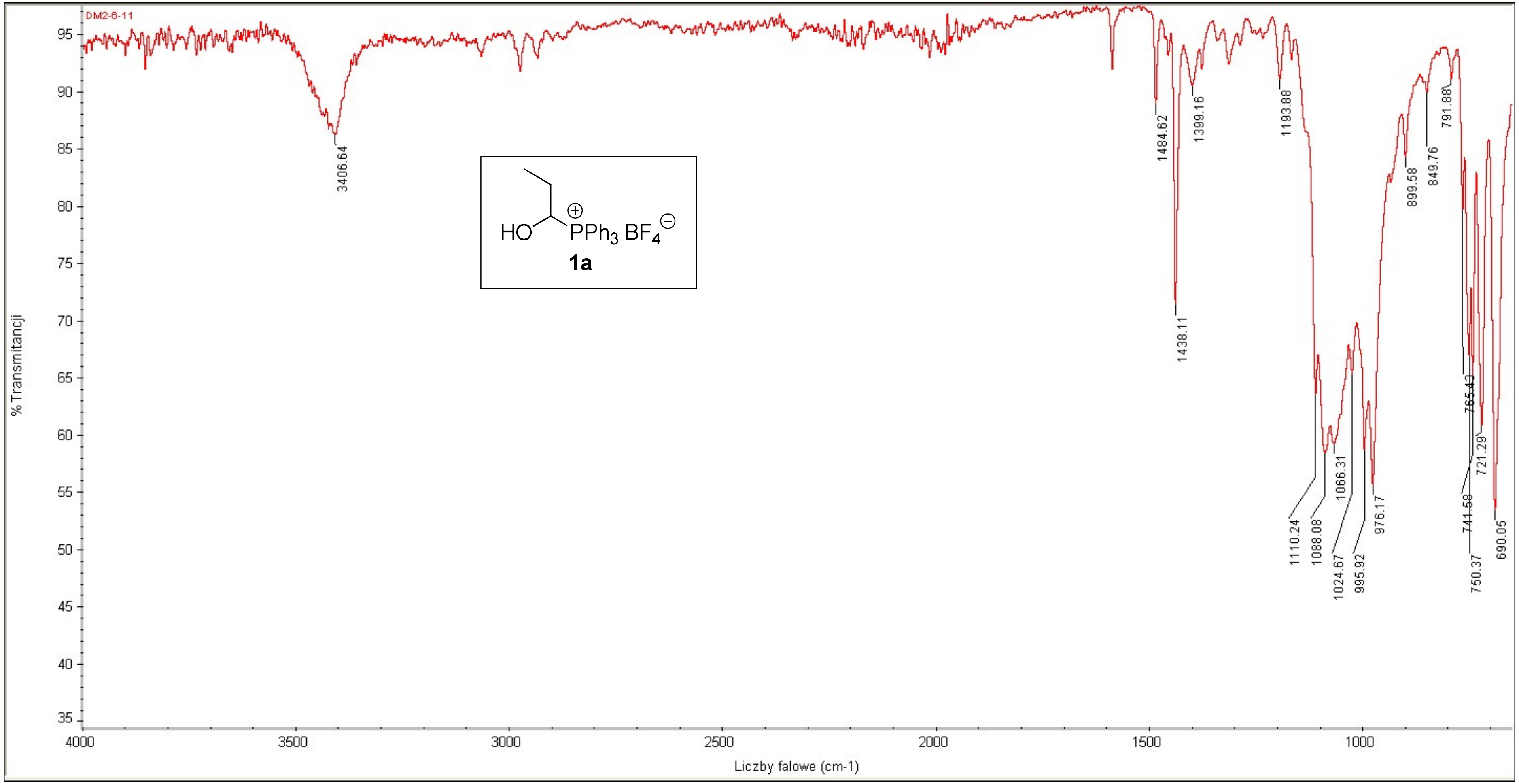

IR spectrum of 1-hydroxypropyltriphenylphosphonium tetrafluoroborate (**1a**); ATR (cm^{-1}).

Tolerance = 40.0 mDa / DBE: min = -10.0, max = 100.0
Element prediction: Off
Number of isotope peaks used for i-FIT = 2
Monoisotopic Mass, Even Electron Ions
5 formula(e) evaluated with 1 results within limits (up to 3 closest results for each mass)
Elements Used:

Mass	RA	Calc. Mass	mDa	PPM	DBE	Formula	i-FIT	i-FIT Norm	Fit Conf %	C	H	O	P
321.1422	100.00	321.1408	1.4	4.4	11.5	C21 H22 O P	425.3	n/a	n/a	21	22	1	1

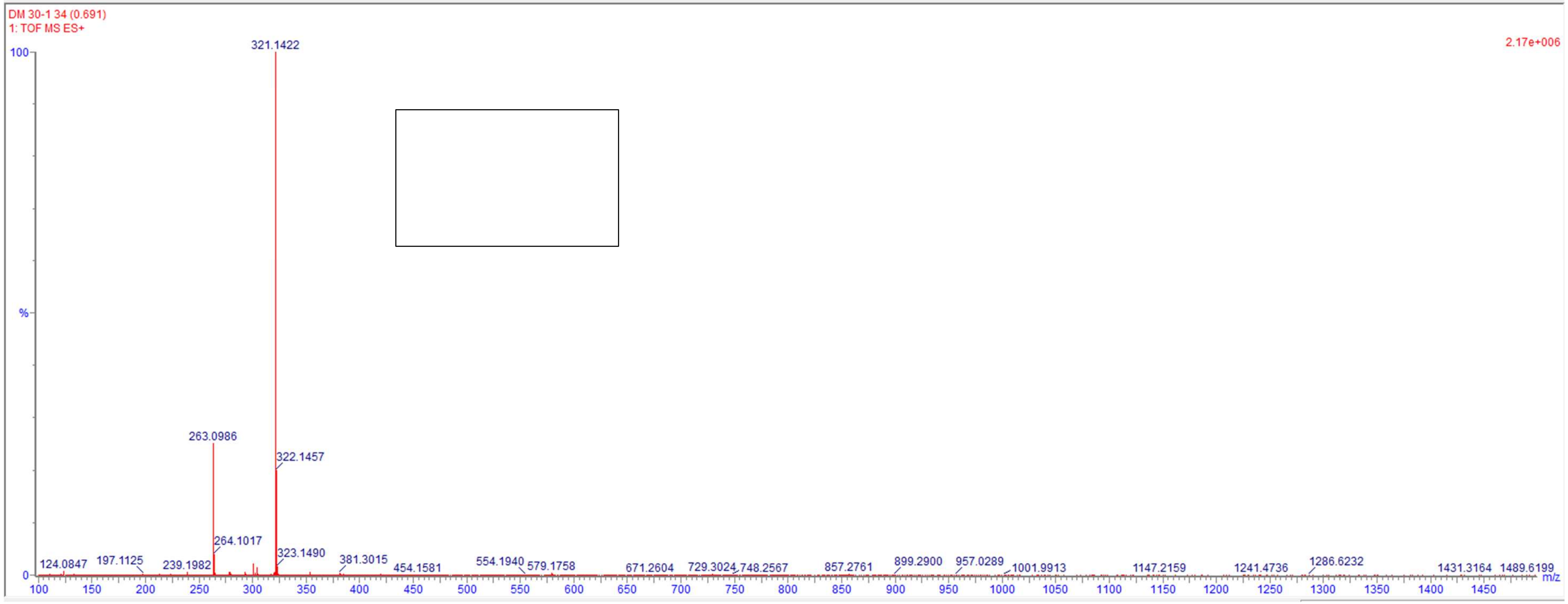

MS spectrum of 1-hydroxypropyltriphenylphosphonium tetrafluoroborate (**1a**).

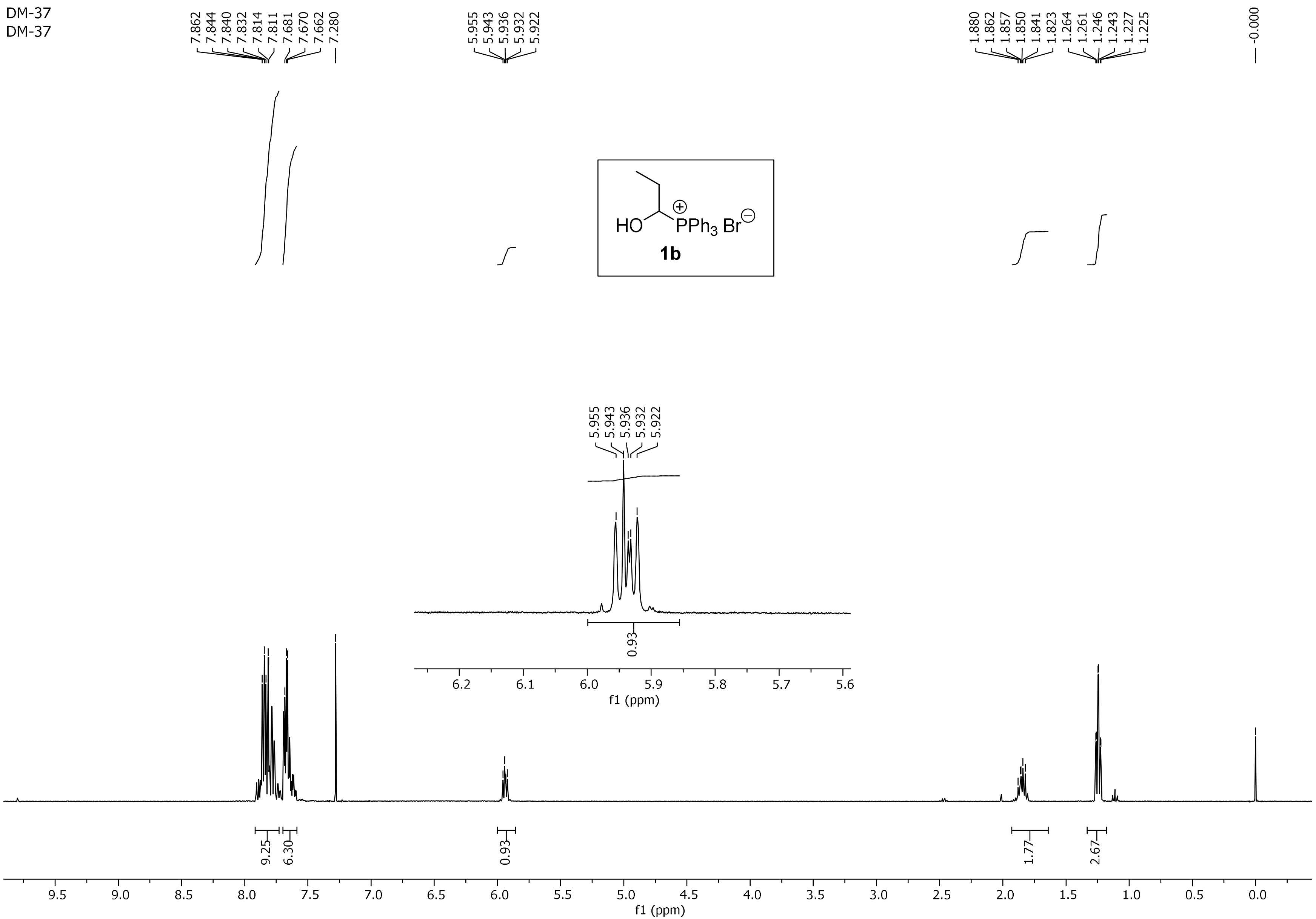

^{1}H NMR spectrum of 1-hydroxypropyltriphenylphosphonium bromide (**1b**); 400 MHz/$CDCl_3$/TMS; δ (ppm).

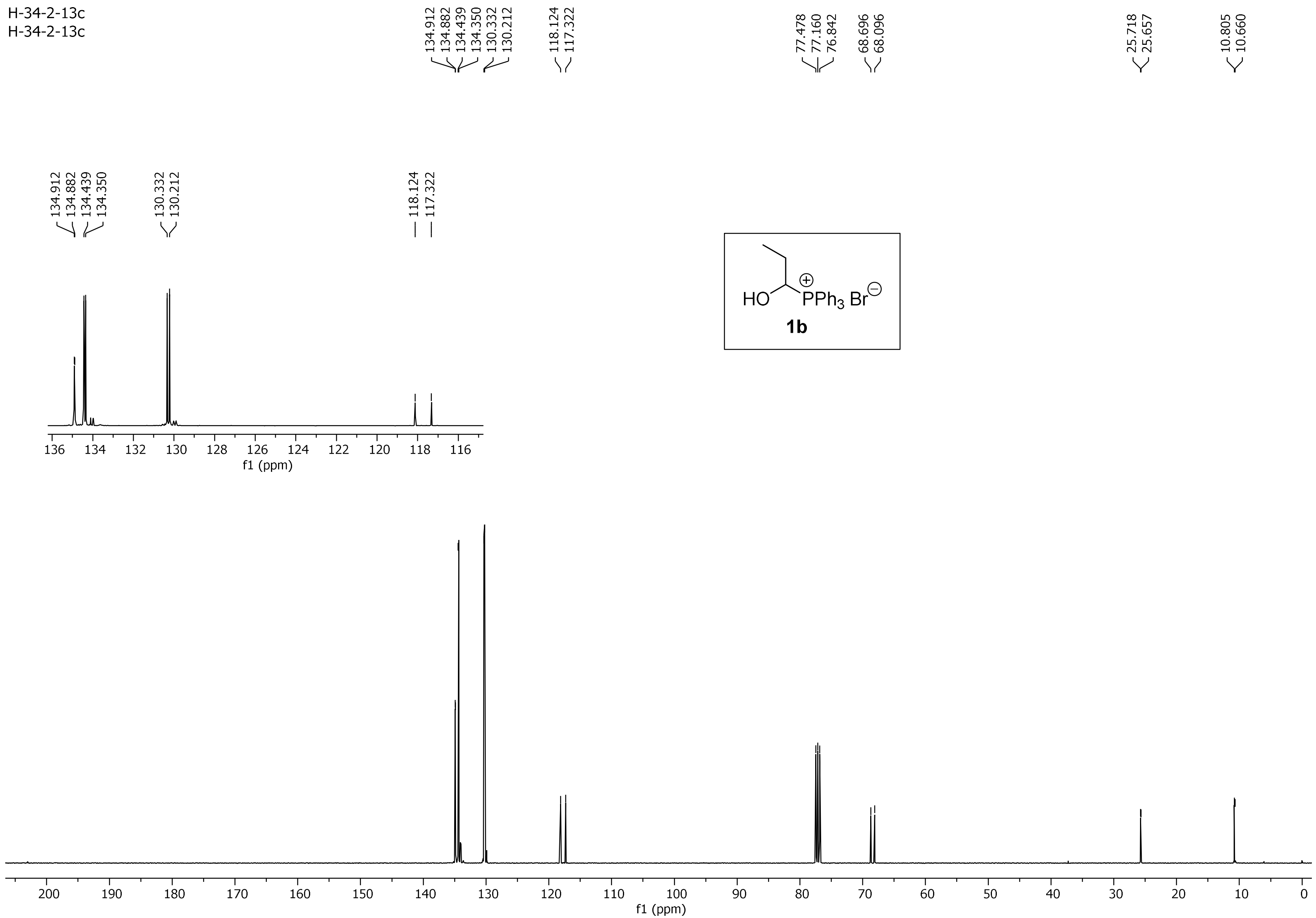

^{13}C NMR spectrum of 1-hydroxypropyltriphenylphosphonium bromide (**1b**); 100 MHz/$CDCl_3$/TMS; *δ* (ppm).

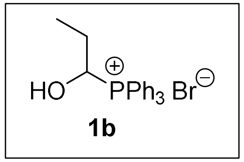

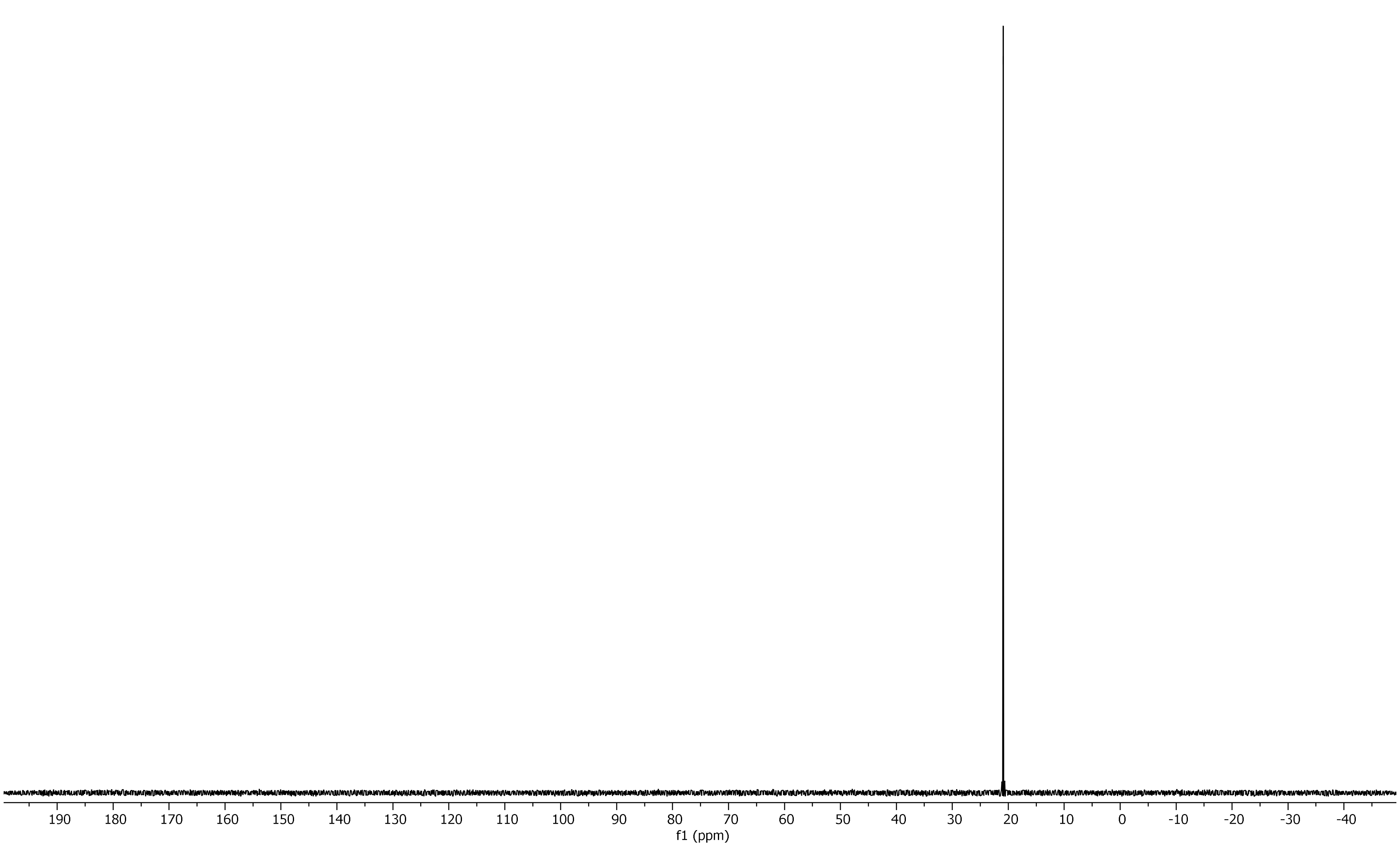

^{31}P NMR spectrum of 1-hydroxypropyltriphenylphosphonium bromide (**1b**); 161.9 MHz/$CDCl_3$; δ (ppm).

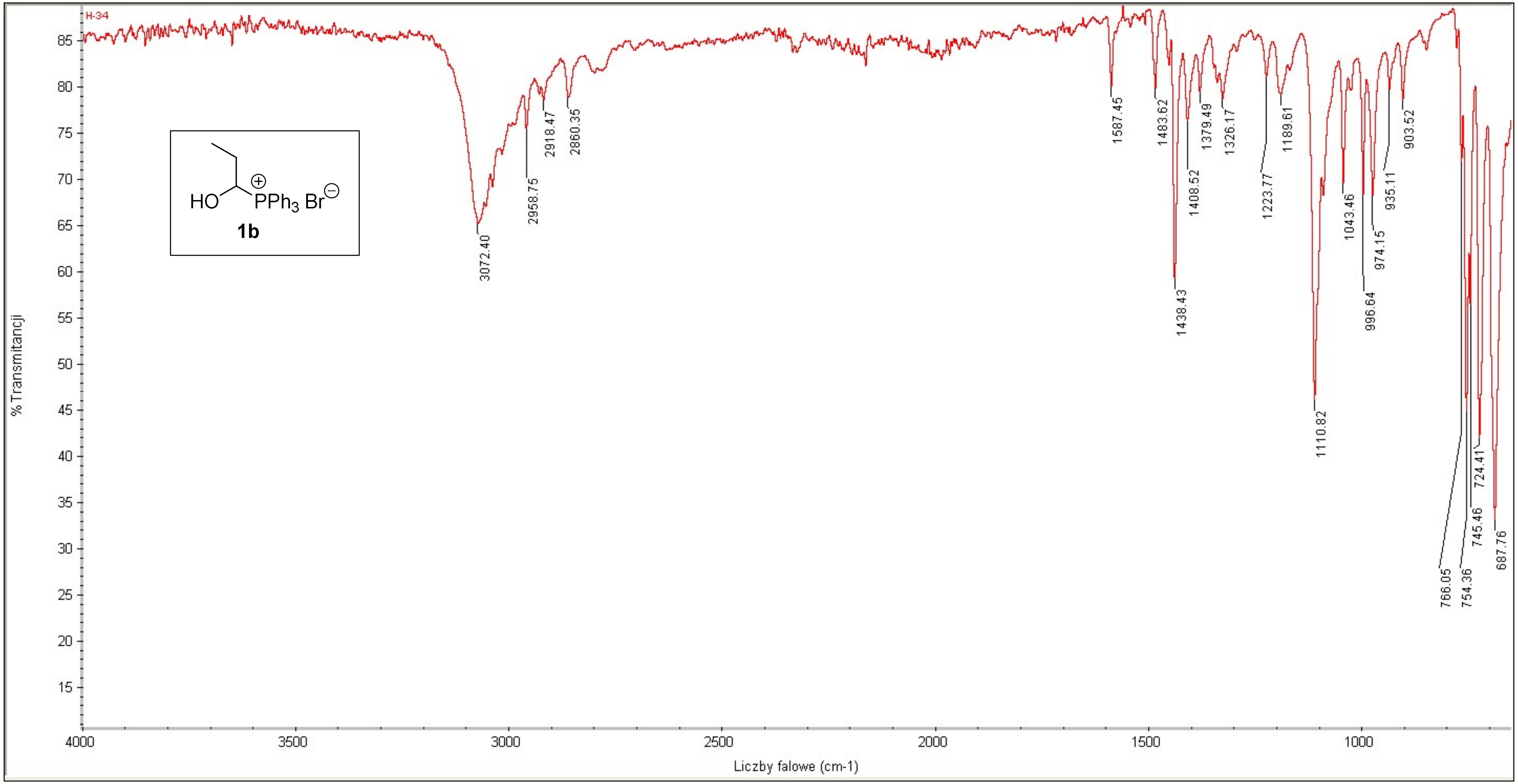

IR spectrum of 1-hydroxypropyltriphenylphosphonium bromide (**1b**); ATR (cm^{-1}).

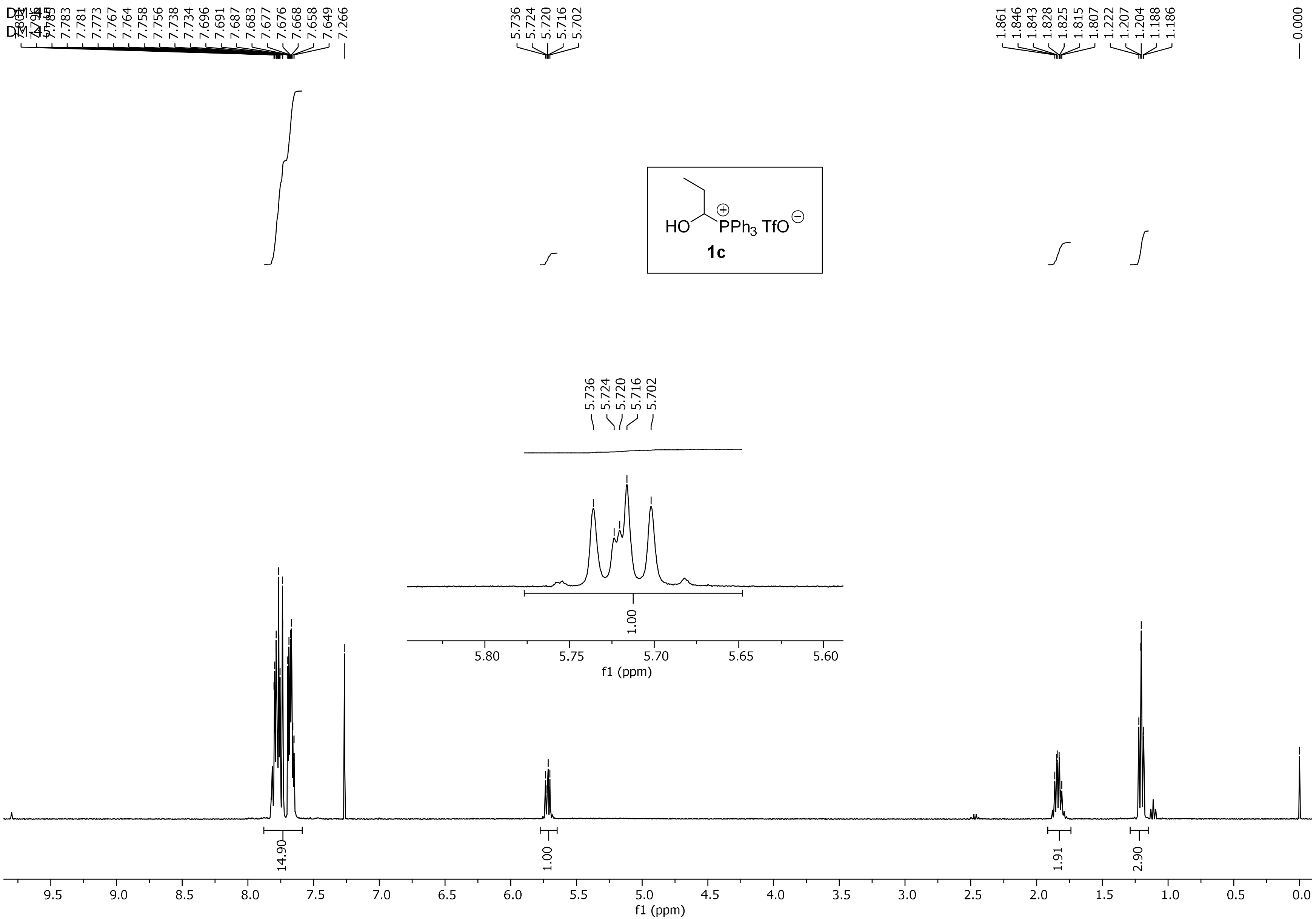

^{1}H NMR spectrum of 1-hydroxypropyltriphenylphosphonium triflate (**1c**); 400 MHz/$CDCl_3$/TMS; δ (ppm).

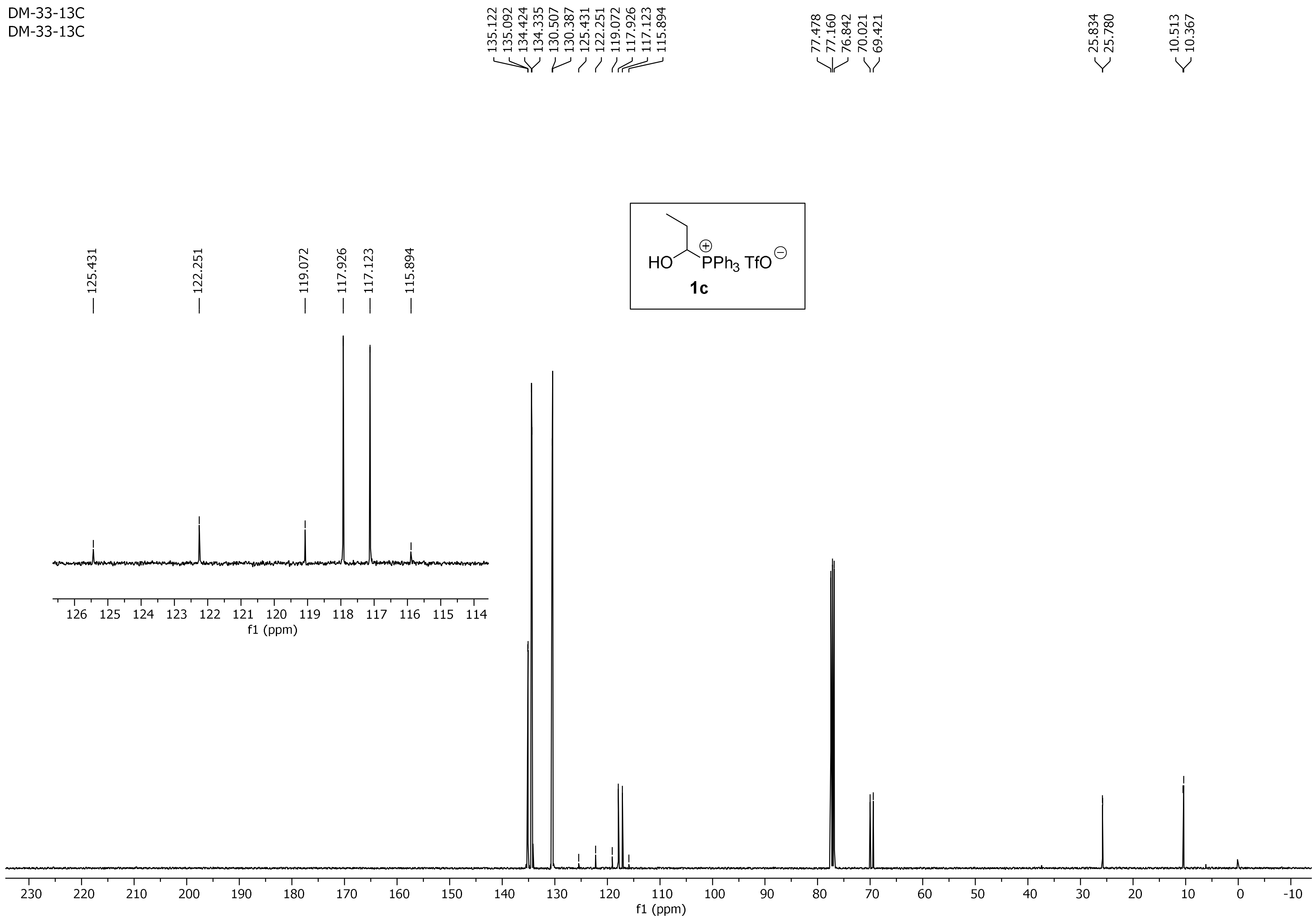

^{13}C NMR spectrum of 1-hydroxypropyltriphenylphosphonium triflate (**1c**); 100 MHz/$CDCl_3$/TMS; δ (ppm).

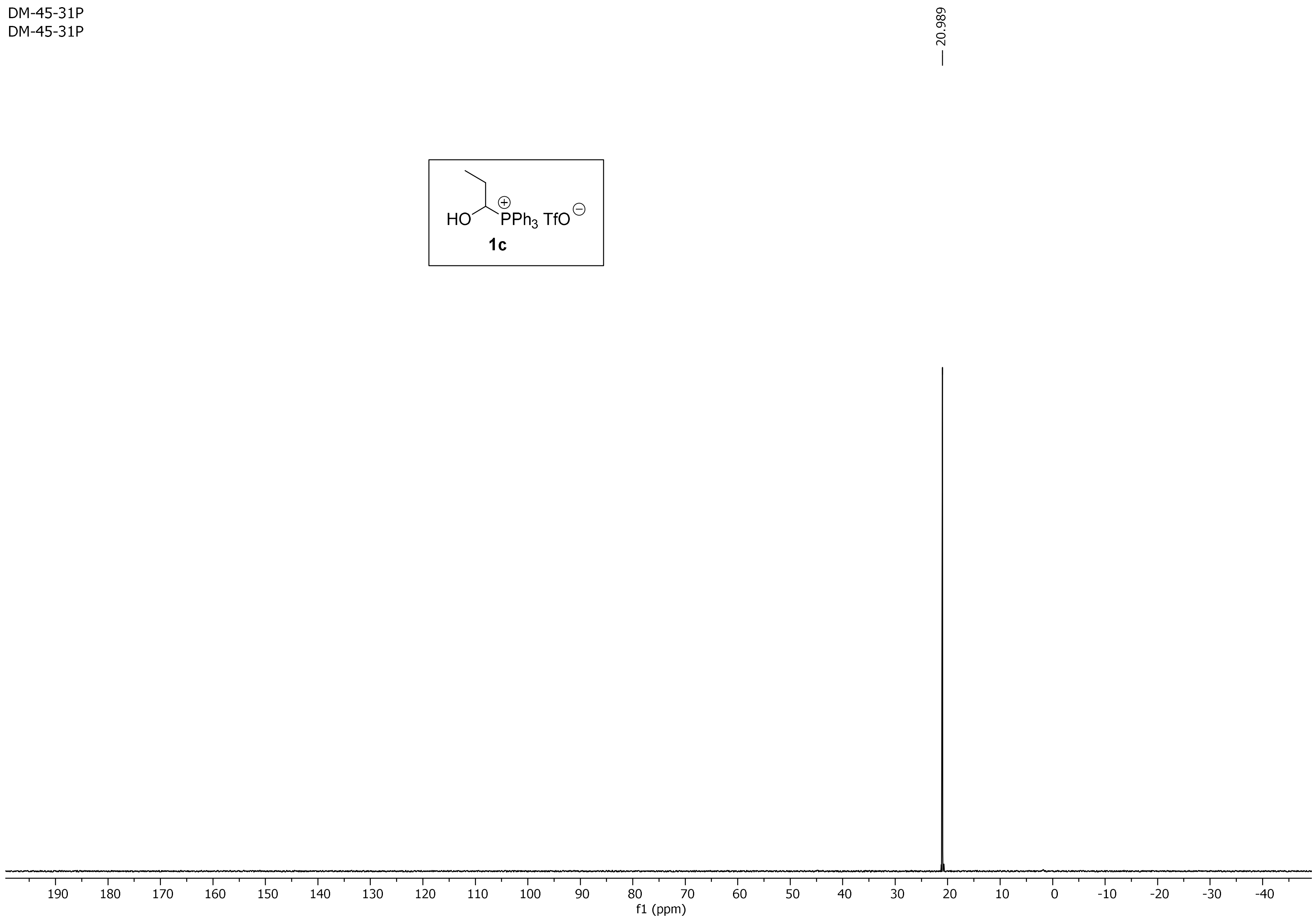

^{31}P NMR spectrum of 1-hydroxypropyltriphenylphosphonium triflate (**1c**); 161.9 MHz/$CDCl_3$; δ (ppm).

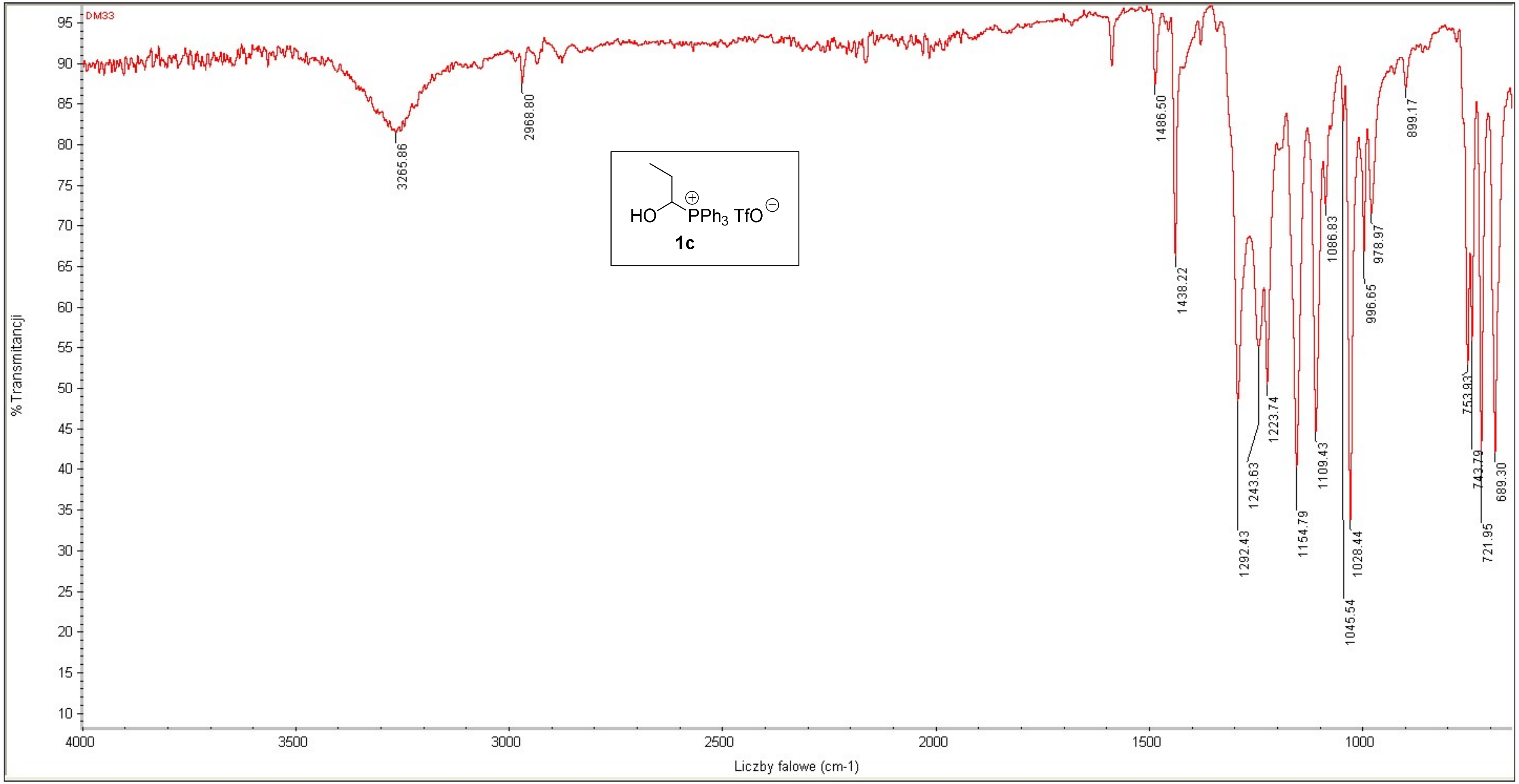

IR spectrum of 1-hydroxypropyltriphenylphosphonium triflate (**1c**); ATR (cm^{-1}).

Tolerance = 40.0 mDa / DBE: min = -10.0, max = 100.0
Element prediction: Off
Number of isotope peaks used for i-FIT = 2
Monoisotopic Mass, Even Electron Ions
5 formula(e) evaluated with 1 results within limits (up to 3 closest results for each mass)
Elements Used:

Mass	RA	Calc. Mass	mDa	PPM	DBE	Formula	i-FIT	i-FIT Norm	Fit Conf %	C	H	O	P
321.1407	100.00	321.1408	-0.1	-0.3	11.5	C21 H22 O P	392.4	n/a	n/a	21	22	1	1

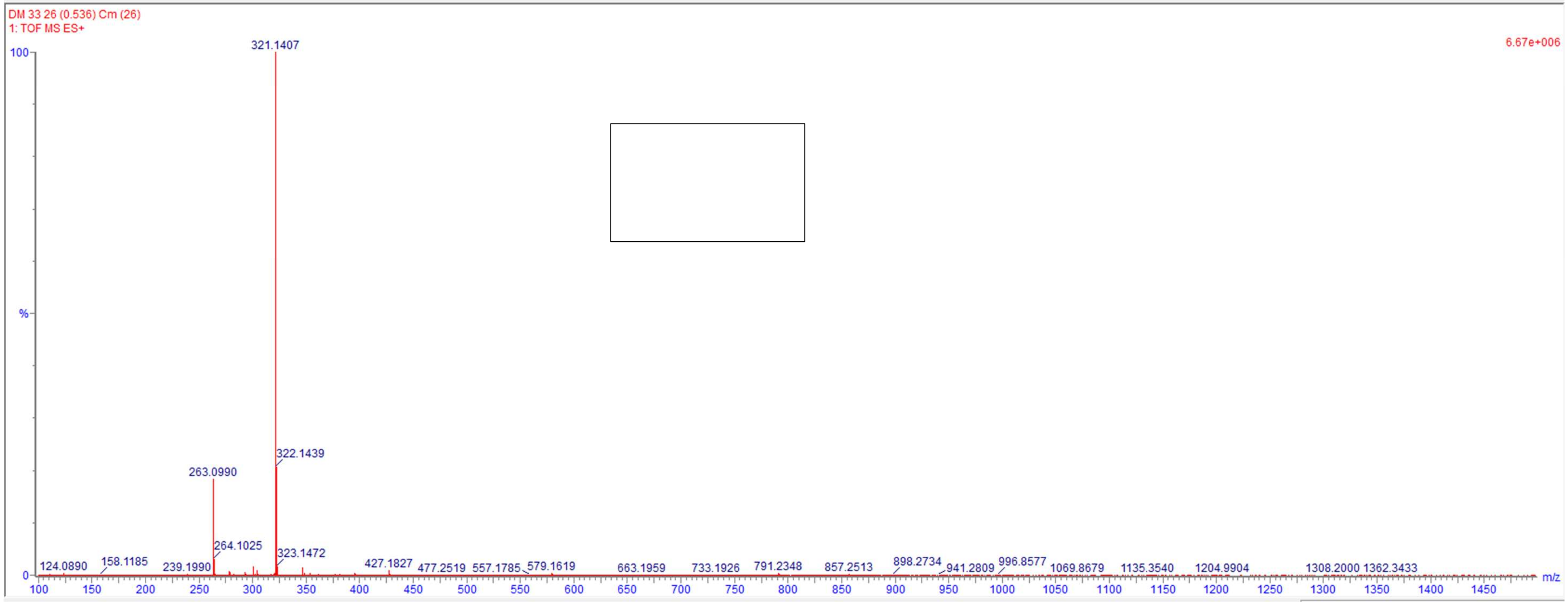

MS spectrum of 1-hydroxypropyltriphenylphosphonium triflate (**1c**).

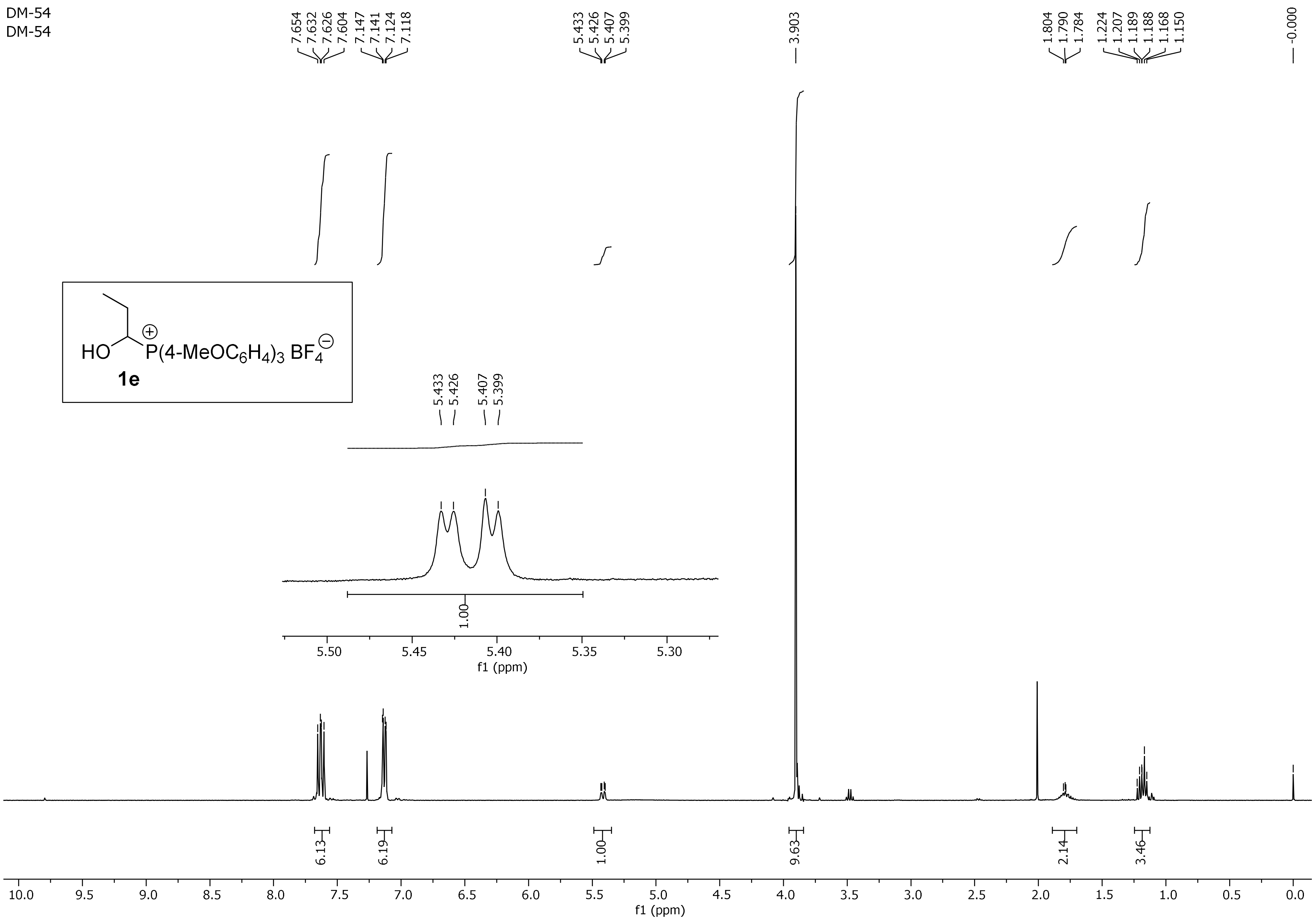

^1H NMR spectrum of 1-hydroxypropyltris(4-methoxyphenyl)phosphonium tetrafluoroborate (**1e**); 400 MHz/$CDCl_3$/TMS; δ (ppm).

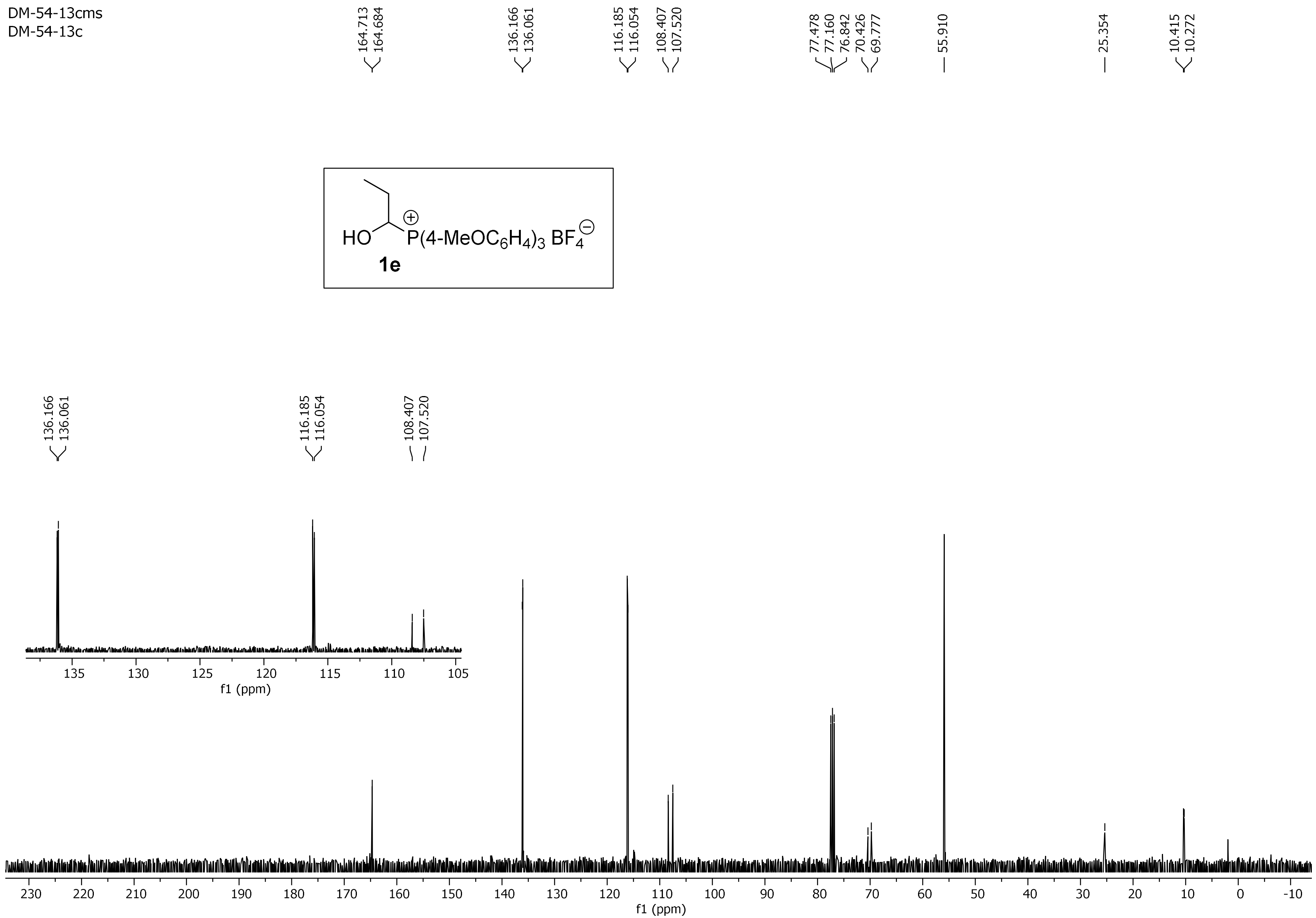

^{13}C NMR spectrum of 1-hydroxypropyltris(4-methoxyphenyl)phosphonium tetrafluoroborate (**1e**); 100 MHz/$CDCl_3$/TMS; δ (ppm).

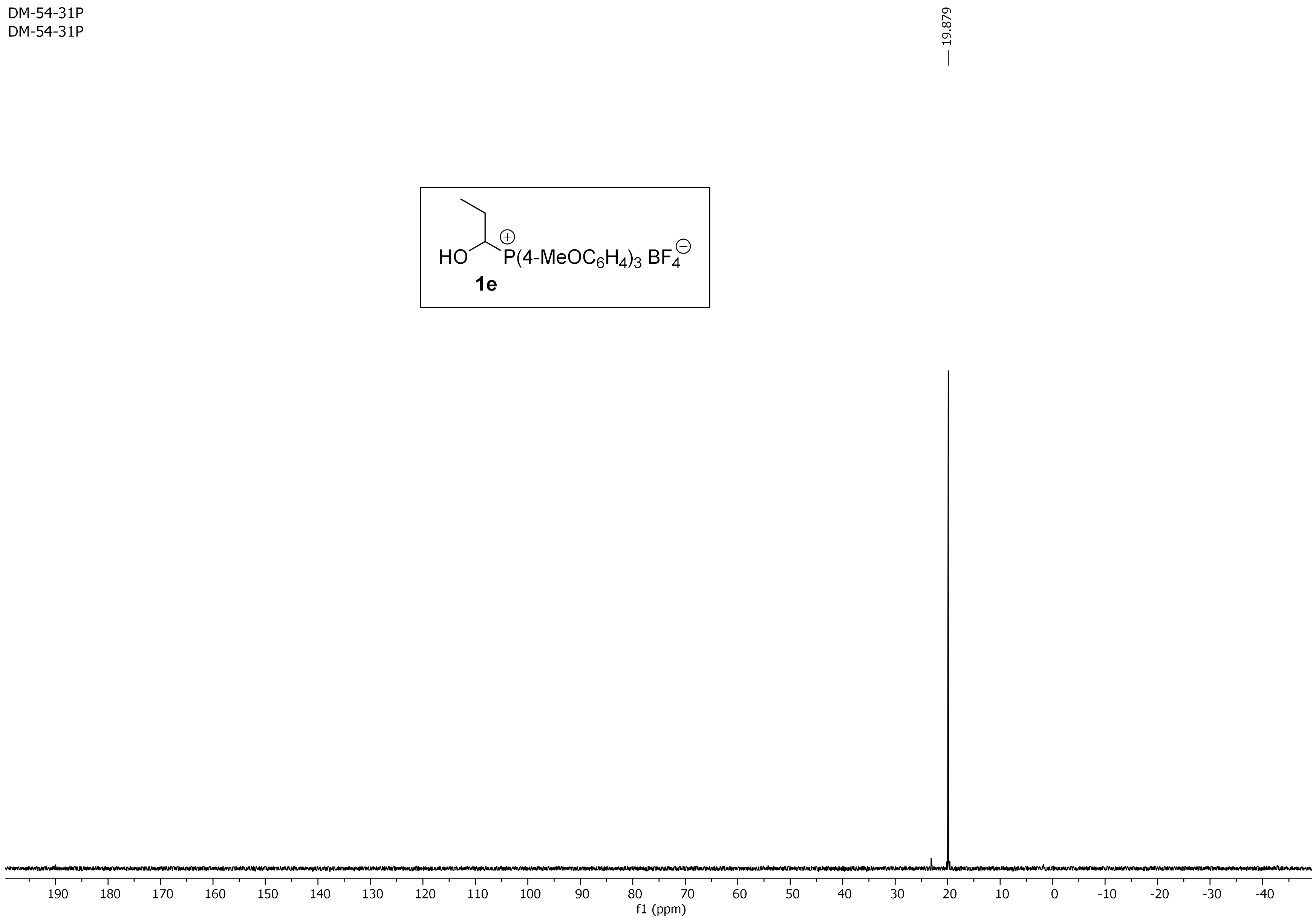

^{31}P NMR spectrum of 1-hydroxypropyltris(4-methoxyphenyl)phosphonium tetrafluoroborate (**1e**); 161.9 MHz/$CDCl_3$; δ (ppm).

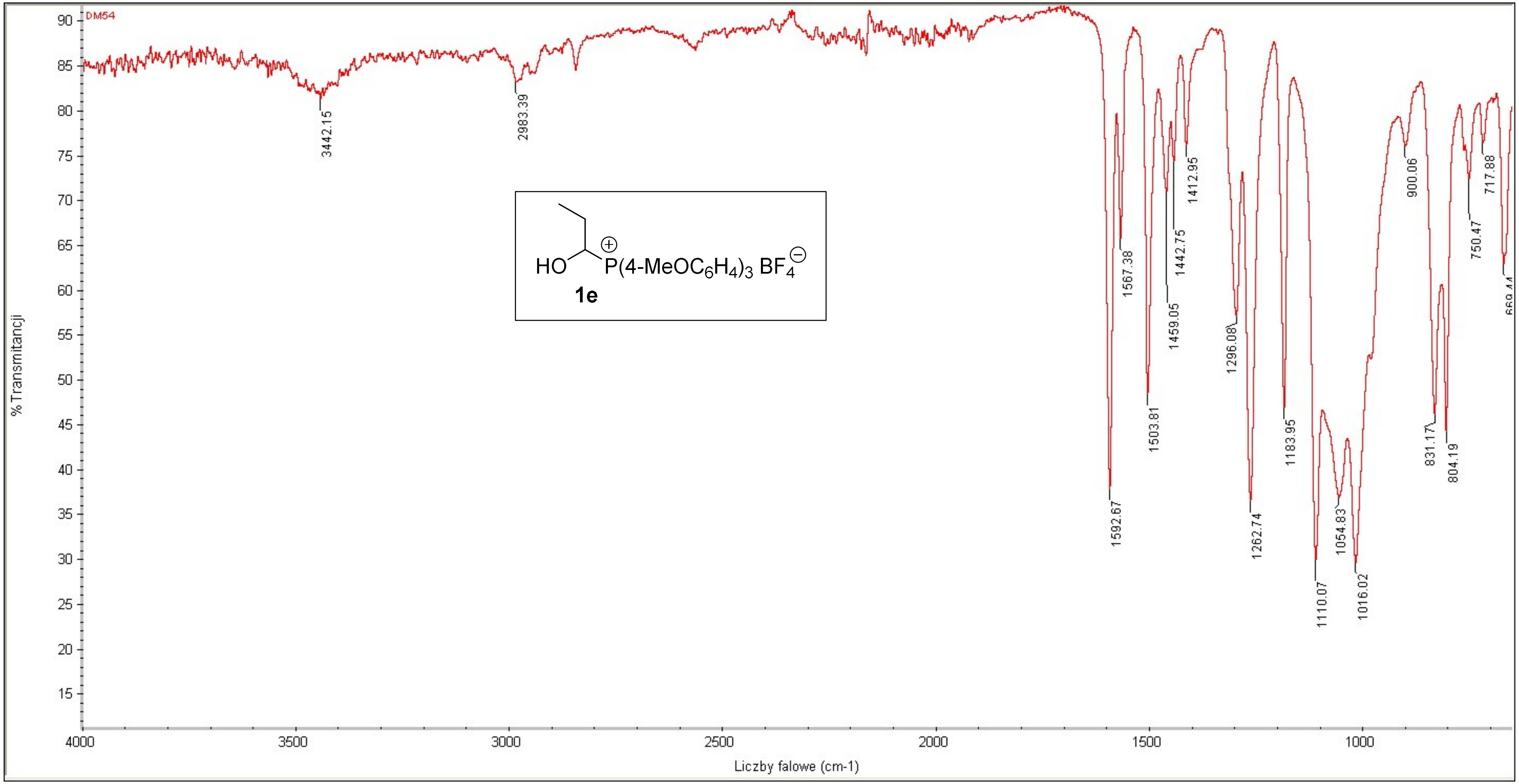

IR spectrum of 1-hydroxypropyltris(4-methoxyphenyl)phosphonium tetrafluoroborate (**1e**); ATR (cm^{-1}).

Tolerance = 40.0 mDa / DBE: min = -10.0, max = 100.0
Element prediction: Off
Number of isotope peaks used for i-FIT = 2
Monoisotopic Mass, Even Electron Ions
6 formula(e) evaluated with 1 results within limits (up to 3 closest results for each mass)
Elements Used:

Mass	RA	Calc. Mass	mDa	PPM	DBE	Formula	i-FIT	i-FIT Norm	Fit Conf %	C	H	O	P
411.1721	100.00	411.1725	-0.4	-1.0	11.5	C24 H28 O4 P	472.0	n/a	n/a	24	28	4	1

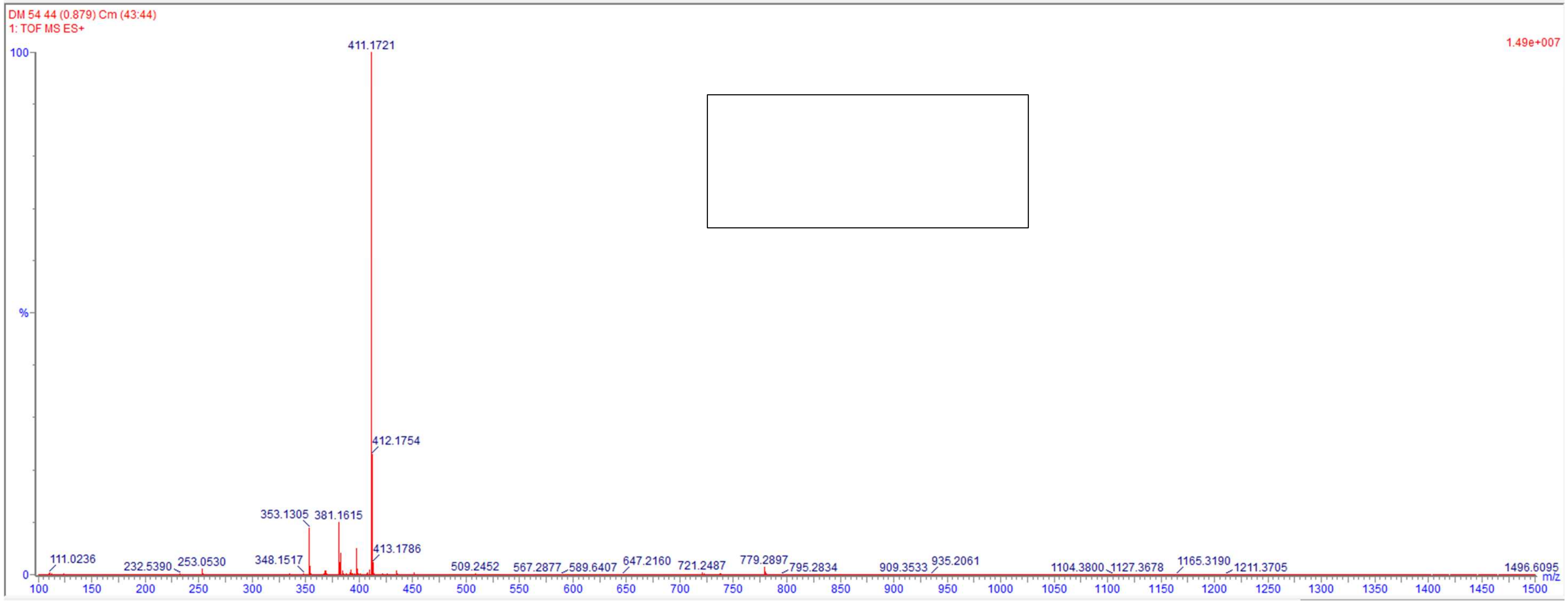

MS spectrum of 1-hydroxypropyltris(4-methoxyphenyl)phosphonium tetrafluoroborate (**1e**).

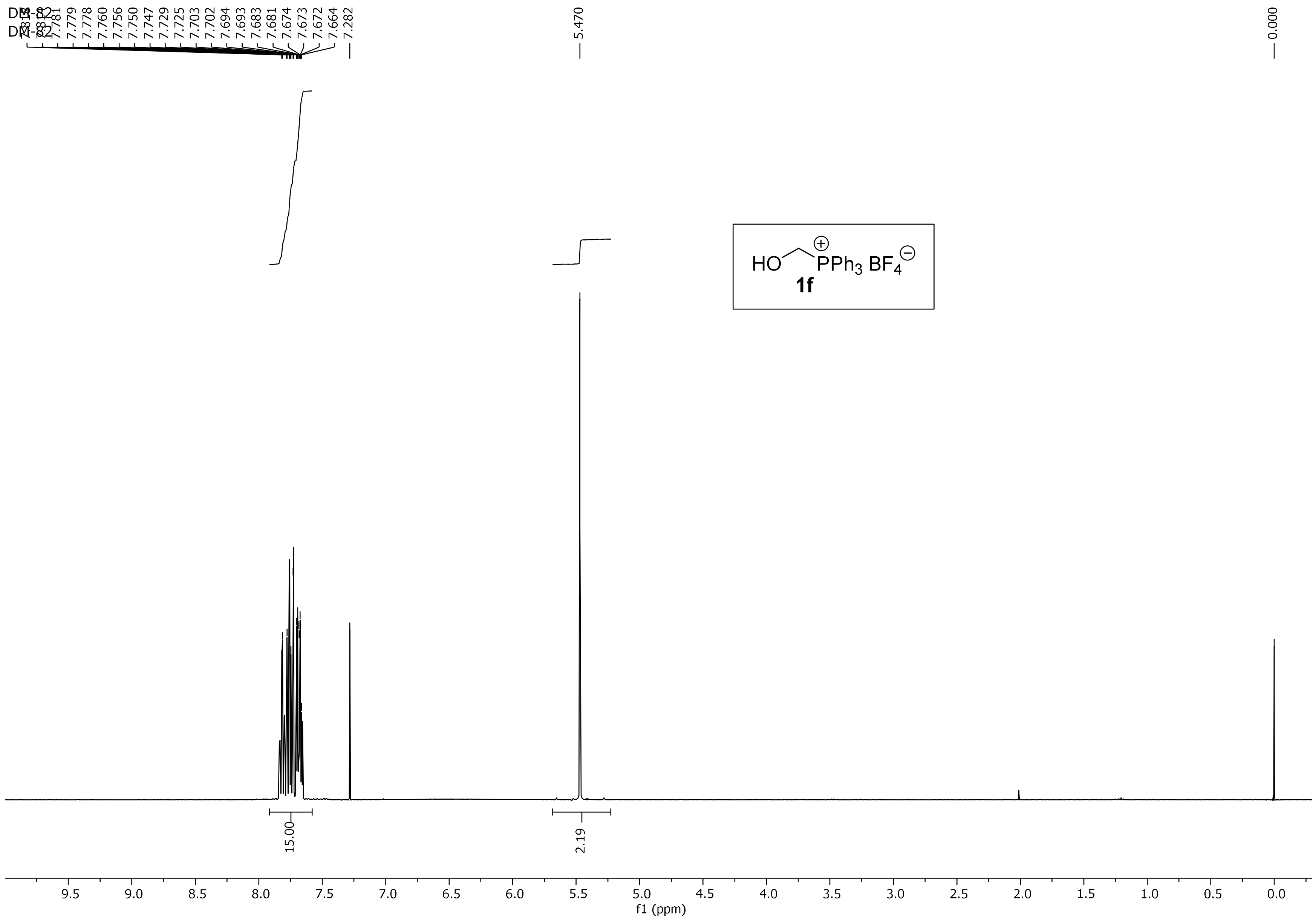

^{1}H NMR spectrum of hydroxymethyltriphenylphosphonium tetrafluoroborate (**1f**); 400 MHz/CDCl$_3$/TMS; δ (ppm).

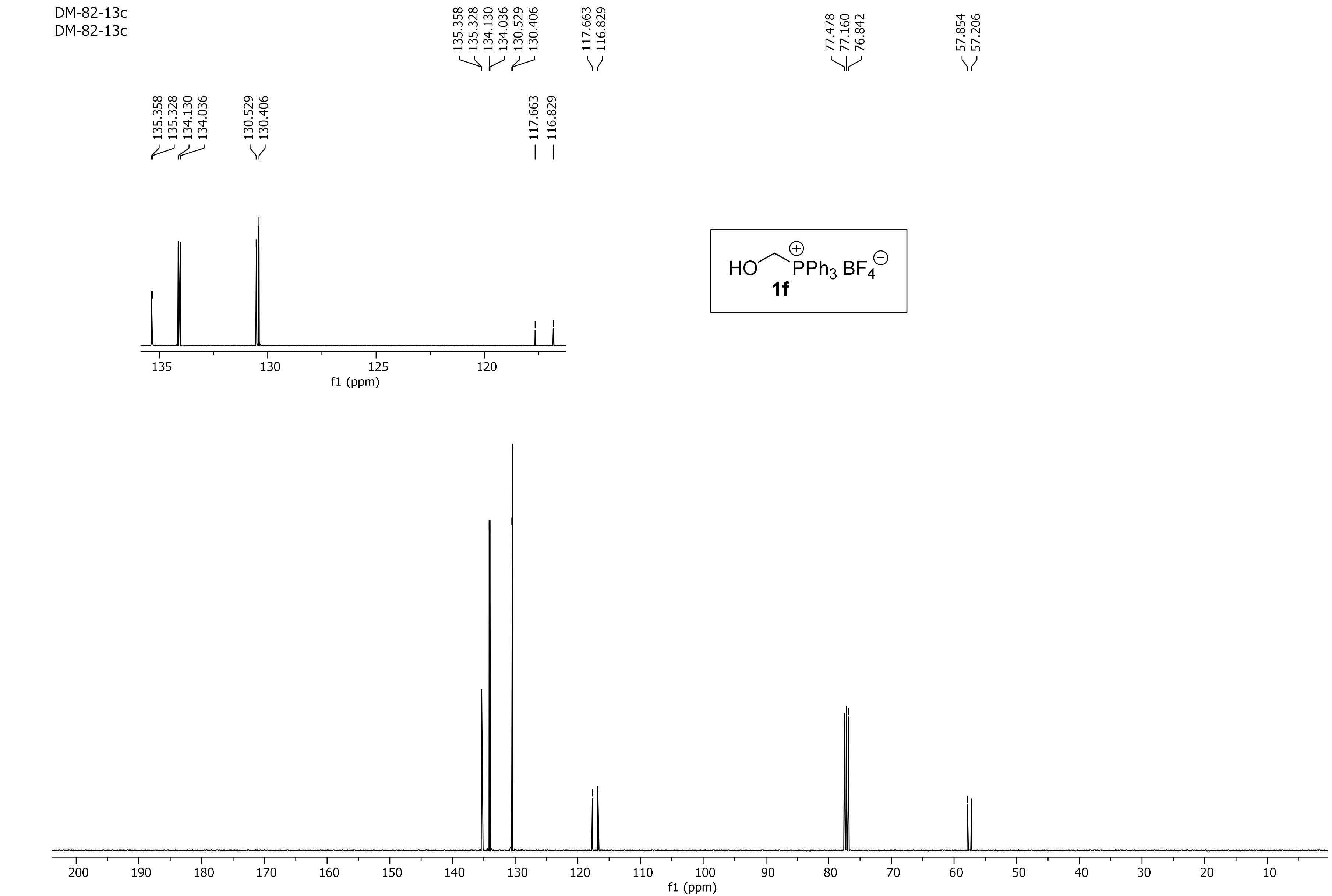

^{13}C NMR spectrum of hydroxymethyltriphenylphosphonium tetrafluoroborate (**1f**); 100 MHz/$CDCl_3$/TMS; δ (ppm).

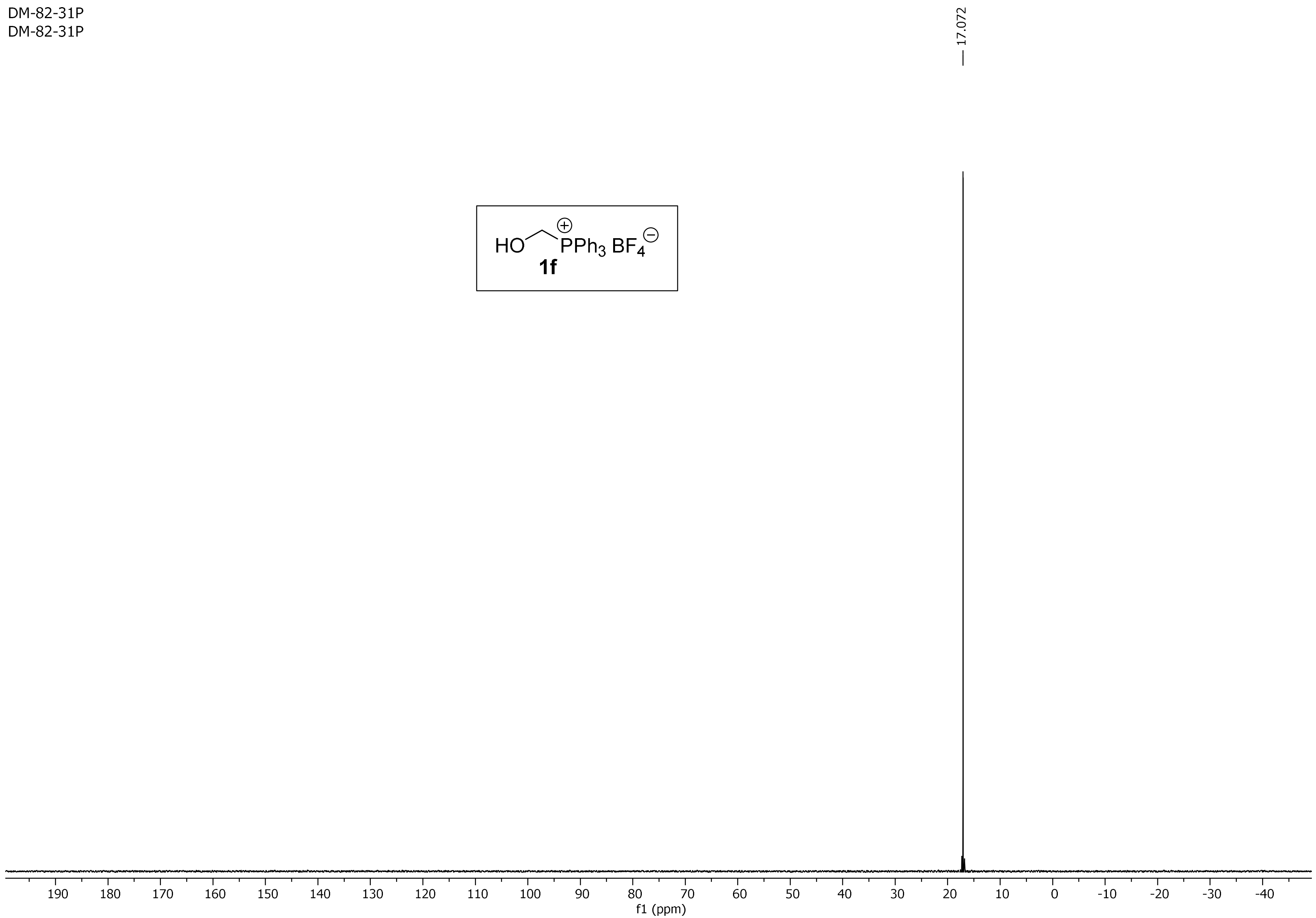

^{31}P NMR spectrum of hydroxymethyltriphenylphosphonium tetrafluoroborate (**1f**); 161.9 MHz/$CDCl_3$; δ (ppm).

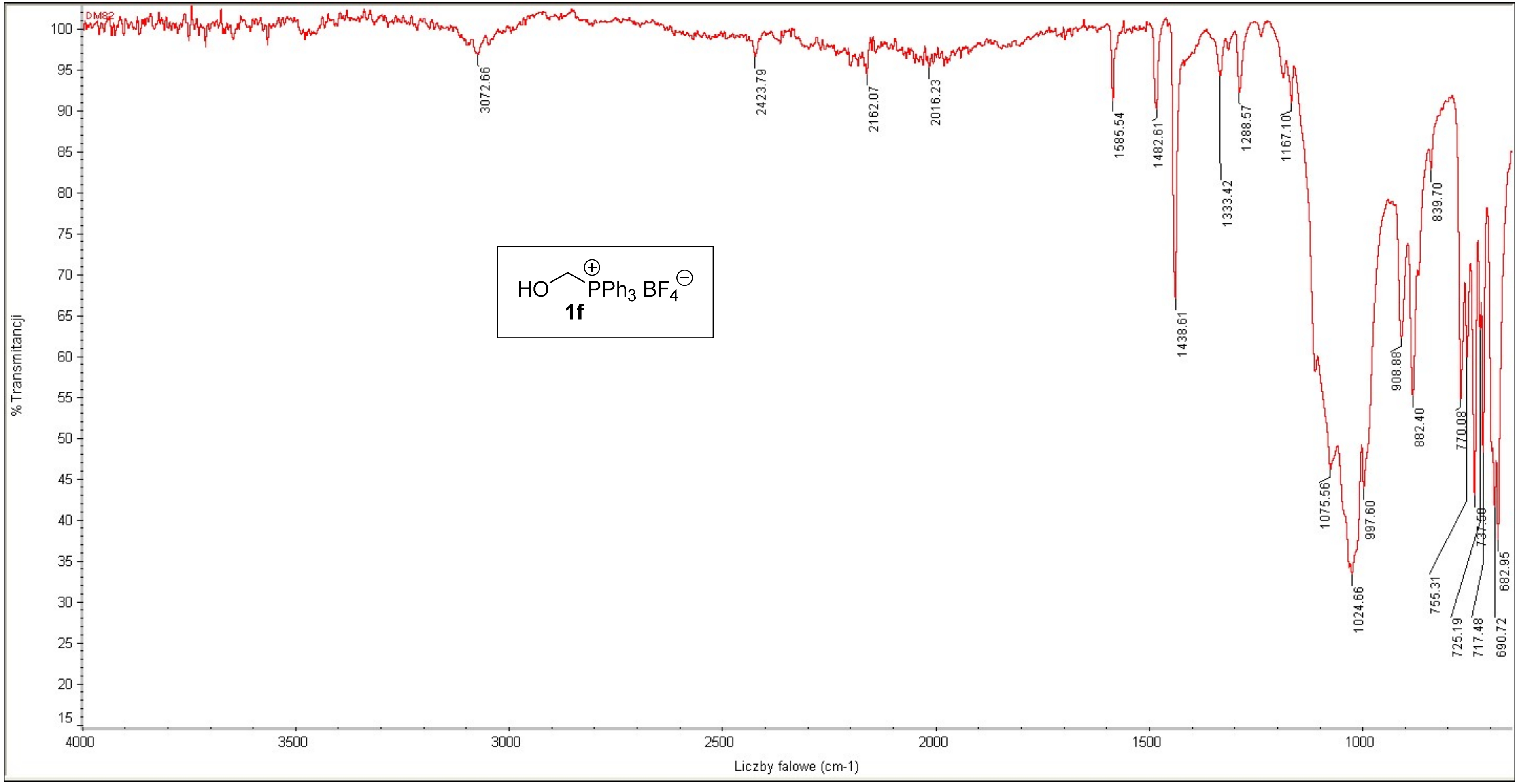

IR spectrum of hydroxymethyltriphenylphosphonium tetrafluoroborate (**1f**); ATR (cm^{-1}).

Tolerance = 40.0 mDa / DBE: min = -10.0, max = 100.0
Element prediction: Off
Number of isotope peaks used for i-FIT = 2
Monoisotopic Mass, Even Electron Ions
5 formula(e) evaluated with 1 results within limits (up to 3 closest results for each mass)
Elements Used:

Mass	RA	Calc. Mass	mDa	PPM	DBE	Formula	i-FIT	i-FIT Norm	Fit Conf %	C	H	O	P
293.1085	100.00	293.1095	-1.0	-3.4	11.5	C19 H18 O P	554.0	n/a	n/a	19	18	1	1

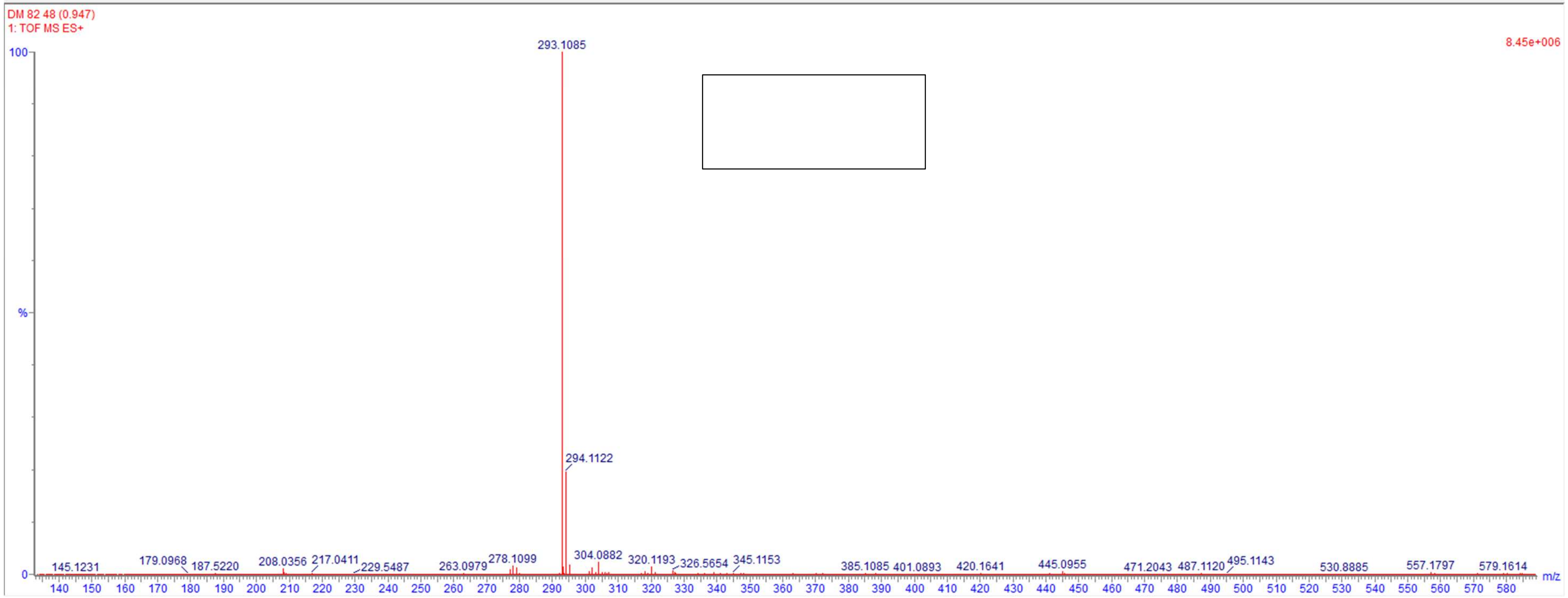

MS spectrum of hydroxymethyltriphenylphosphonium tetrafluoroborate (**1f**).

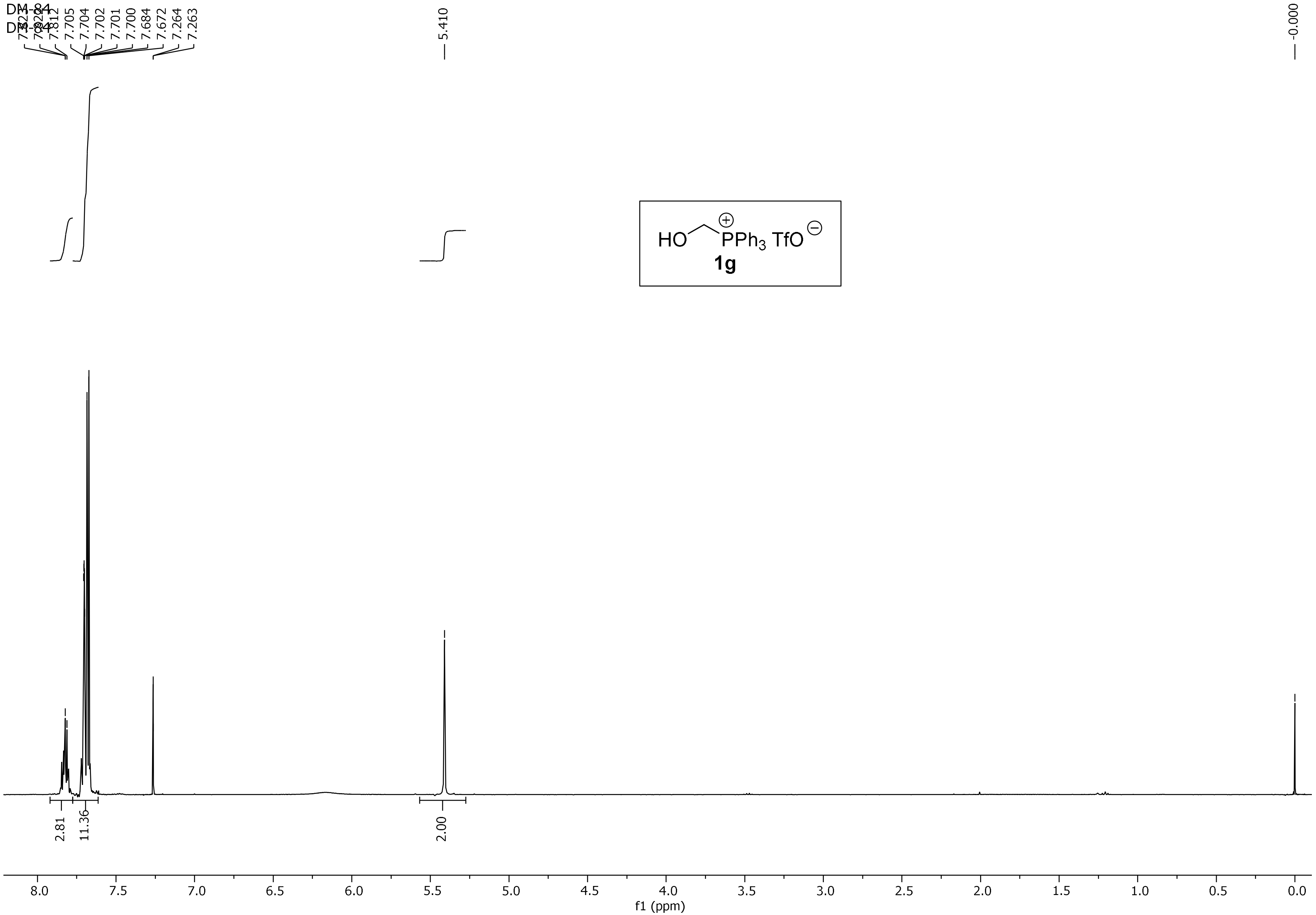

^1H NMR spectrum of hydroxymethyltriphenylphosphonium triflate (**1g**); 400 MHz/$CDCl_3$/TMS; δ (ppm).

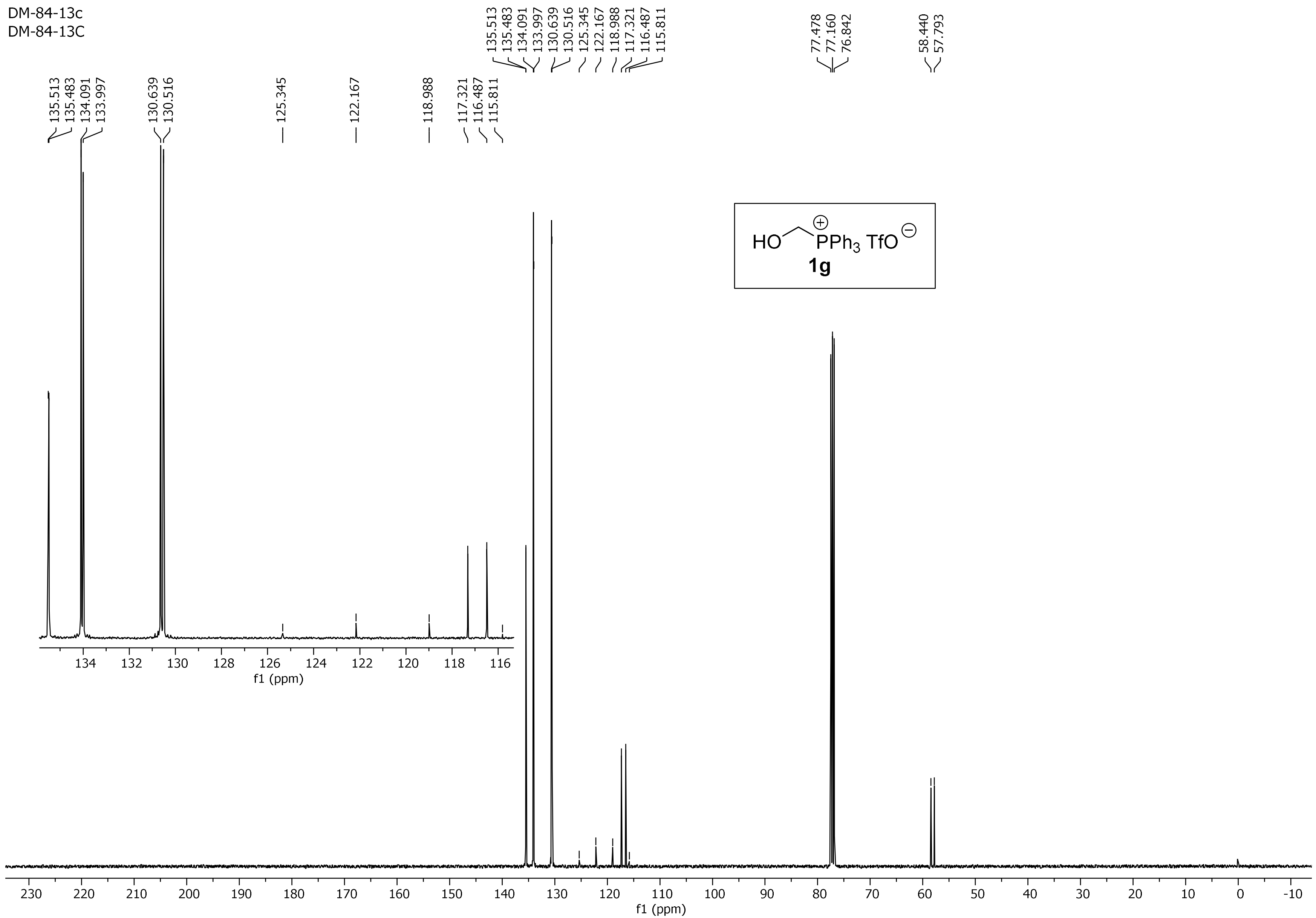

^{13}C NMR spectrum of hydroxymethyltriphenylphosphonium triflate (**1g**); 100 MHz/$CDCl_3$/TMS; δ (ppm).

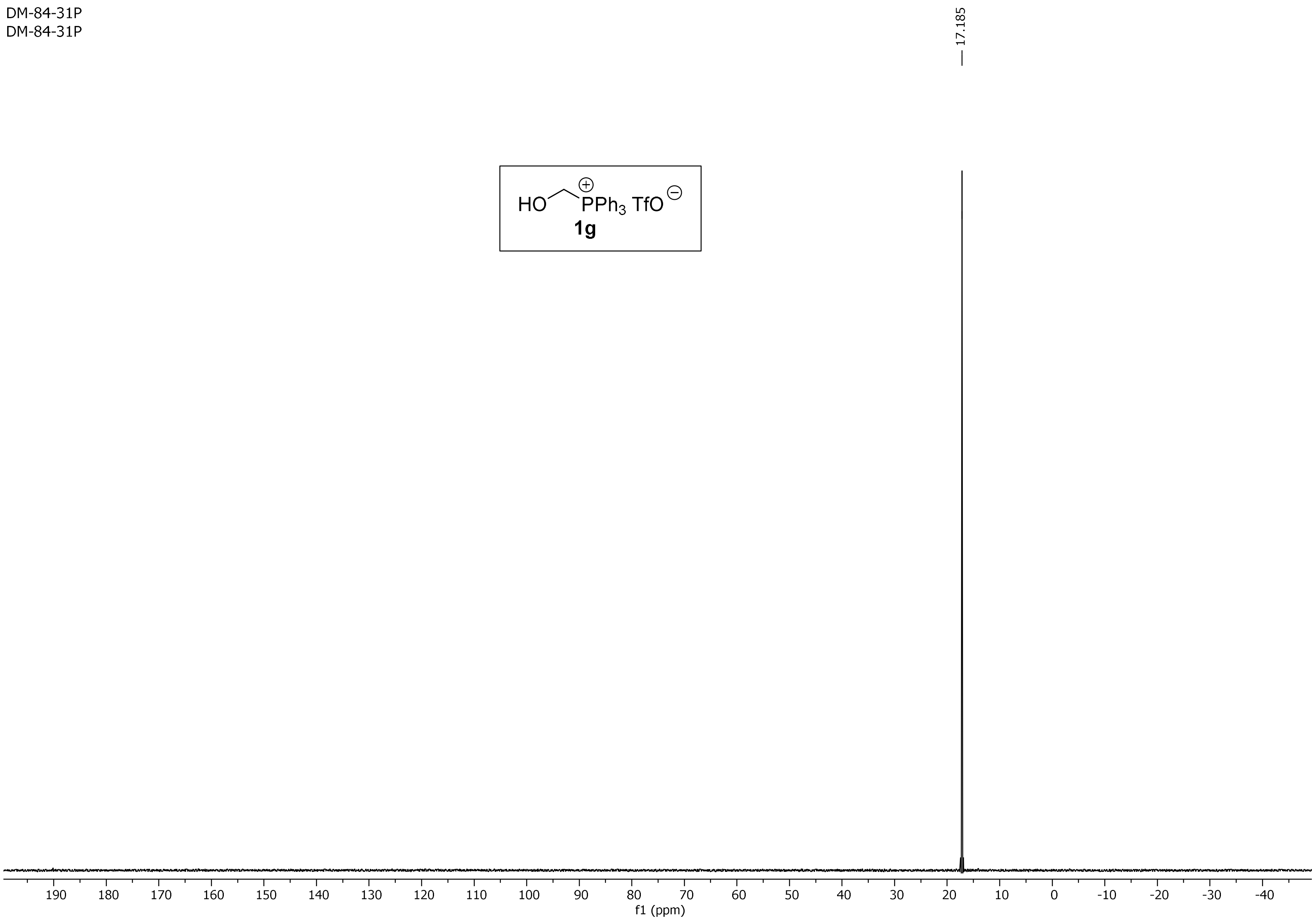

^{31}P NMR spectrum of hydroxymethyltriphenylphosphonium triflate (**1g**); 161.9 MHz/$CDCl_3$; δ (ppm).

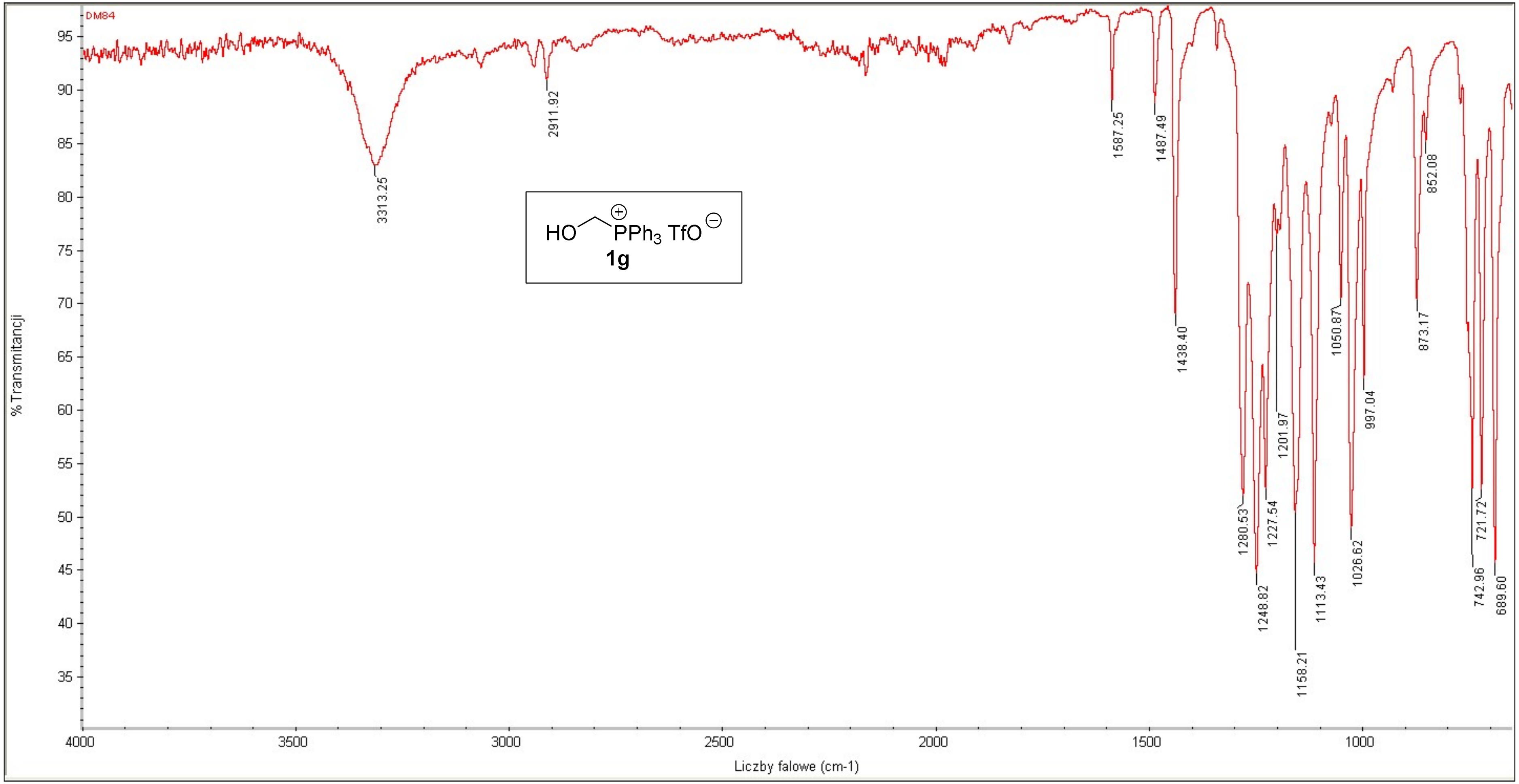

IR spectrum of hydroxymethyltriphenylphosphonium triflate (**1g**); ATR (cm^{-1}).

Tolerance = 40.0 mDa / DBE: min = -10.0, max = 100.0
Element prediction: Off
Number of isotope peaks used for i-FIT = 2
Monoisotopic Mass, Even Electron Ions
5 formula(e) evaluated with 1 results within limits (up to 3 closest results for each mass)
Elements Used:

Mass	RA	Calc. Mass	mDa	PPM	DBE	Formula	i-FIT	i-FIT Norm	Fit Conf %	C	H	O	P
293.1096	100.00	293.1095	0.1	0.3	11.5	C19 H18 O P	460.6	n/a	n/a	19	18	1	1

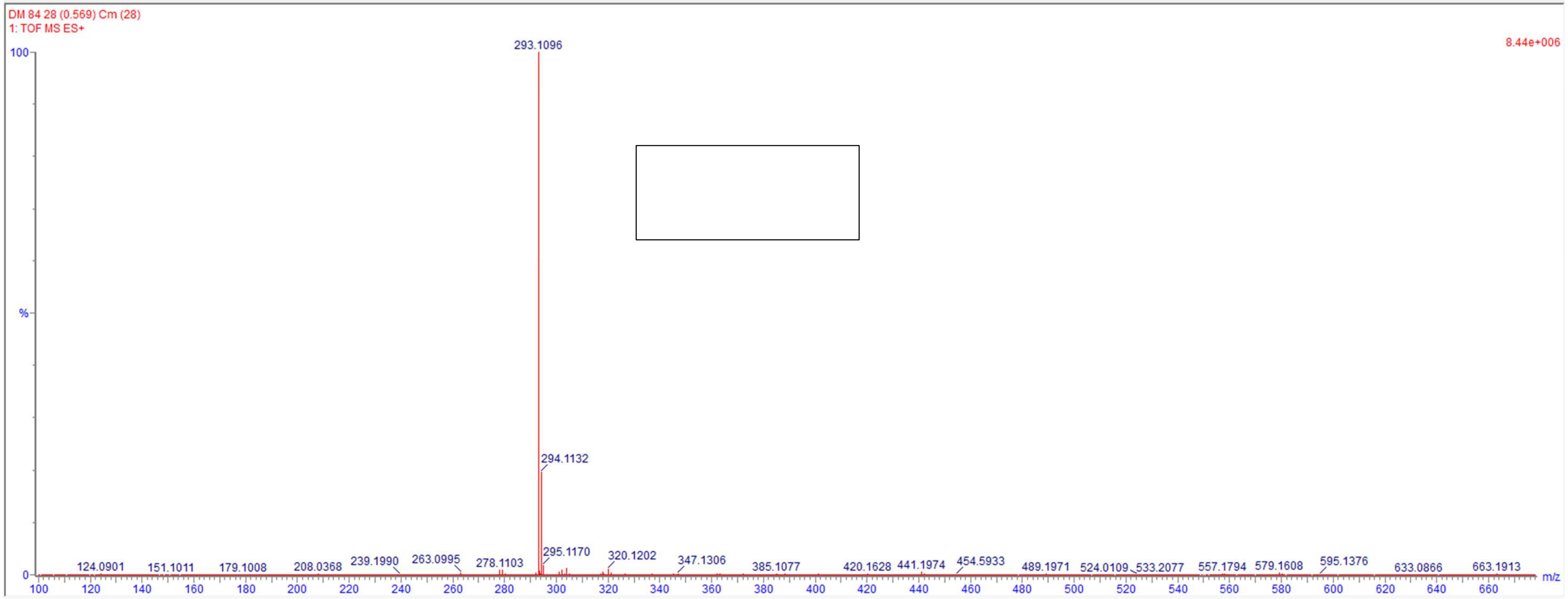

MS spectrum of hydroxymethyltriphenylphosphonium triflate (**1g**).

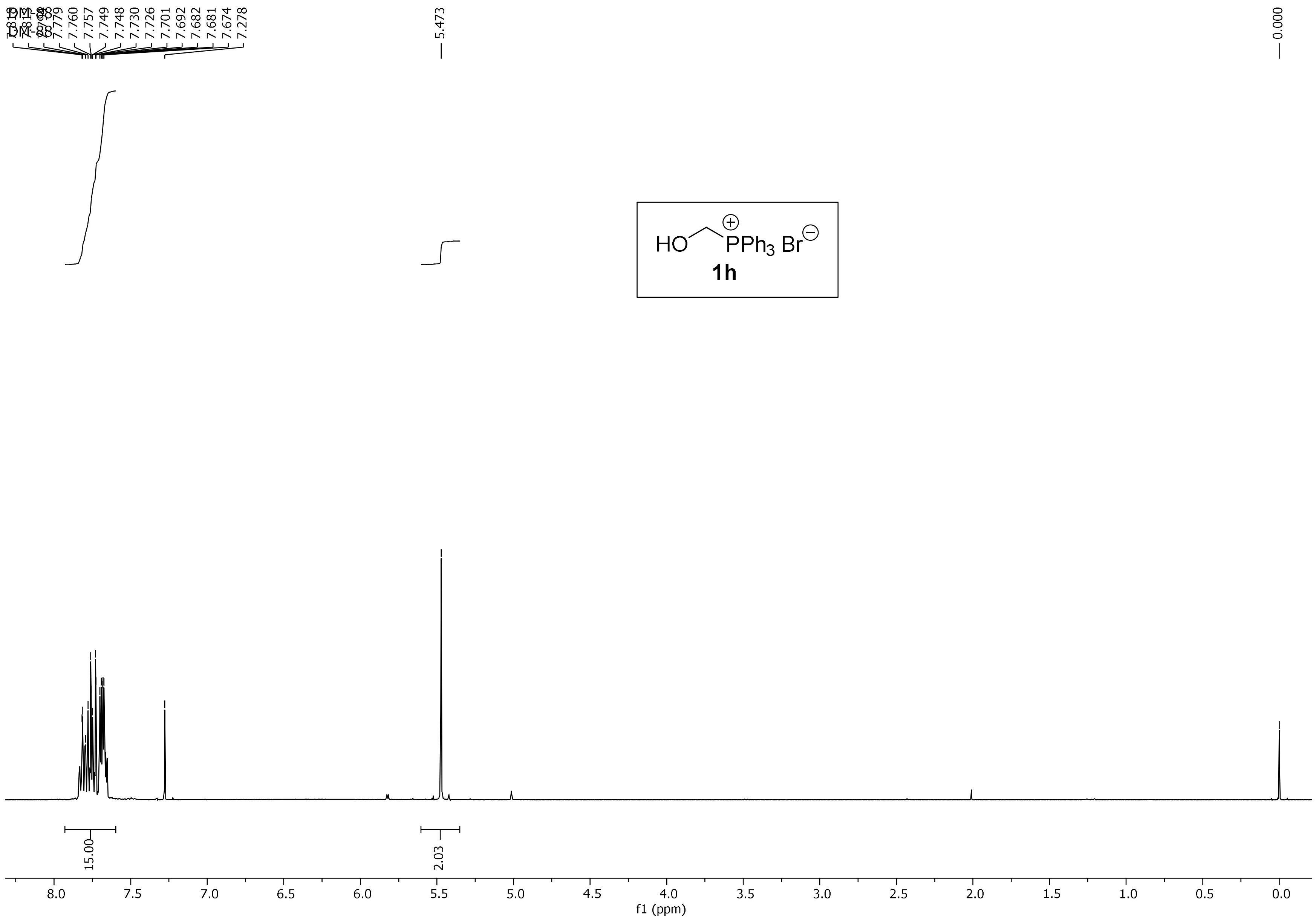

¹H NMR spectrum of hydroxymethyltriphenylphosphonium bromide (**1h**); 400 MHz/$CDCl_3$/TMS; δ (ppm).

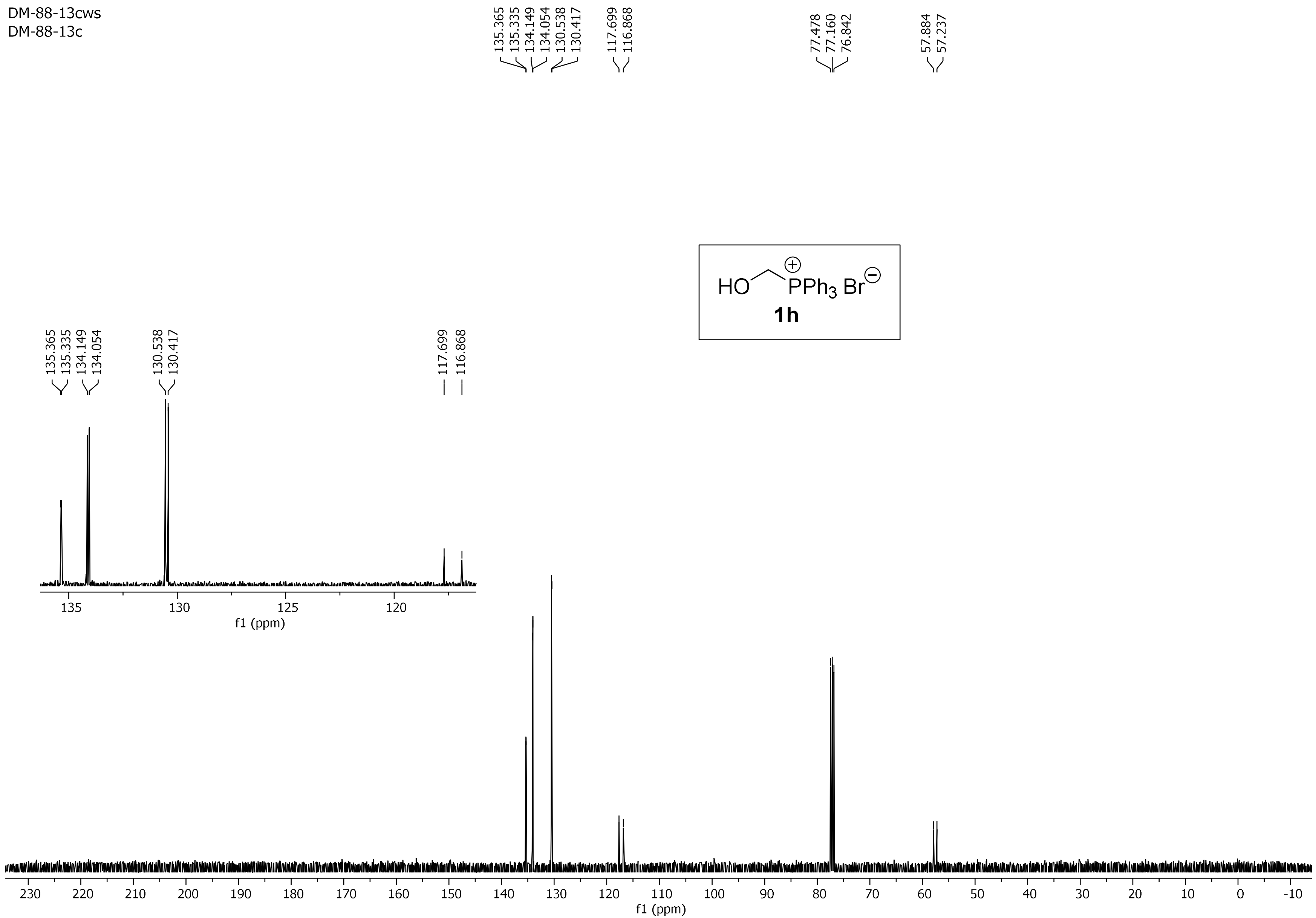

^{13}C NMR spectrum of hydroxymethyltriphenylphosphonium bromide (**1h**); 100 MHz/$CDCl_3$/TMS; δ (ppm).

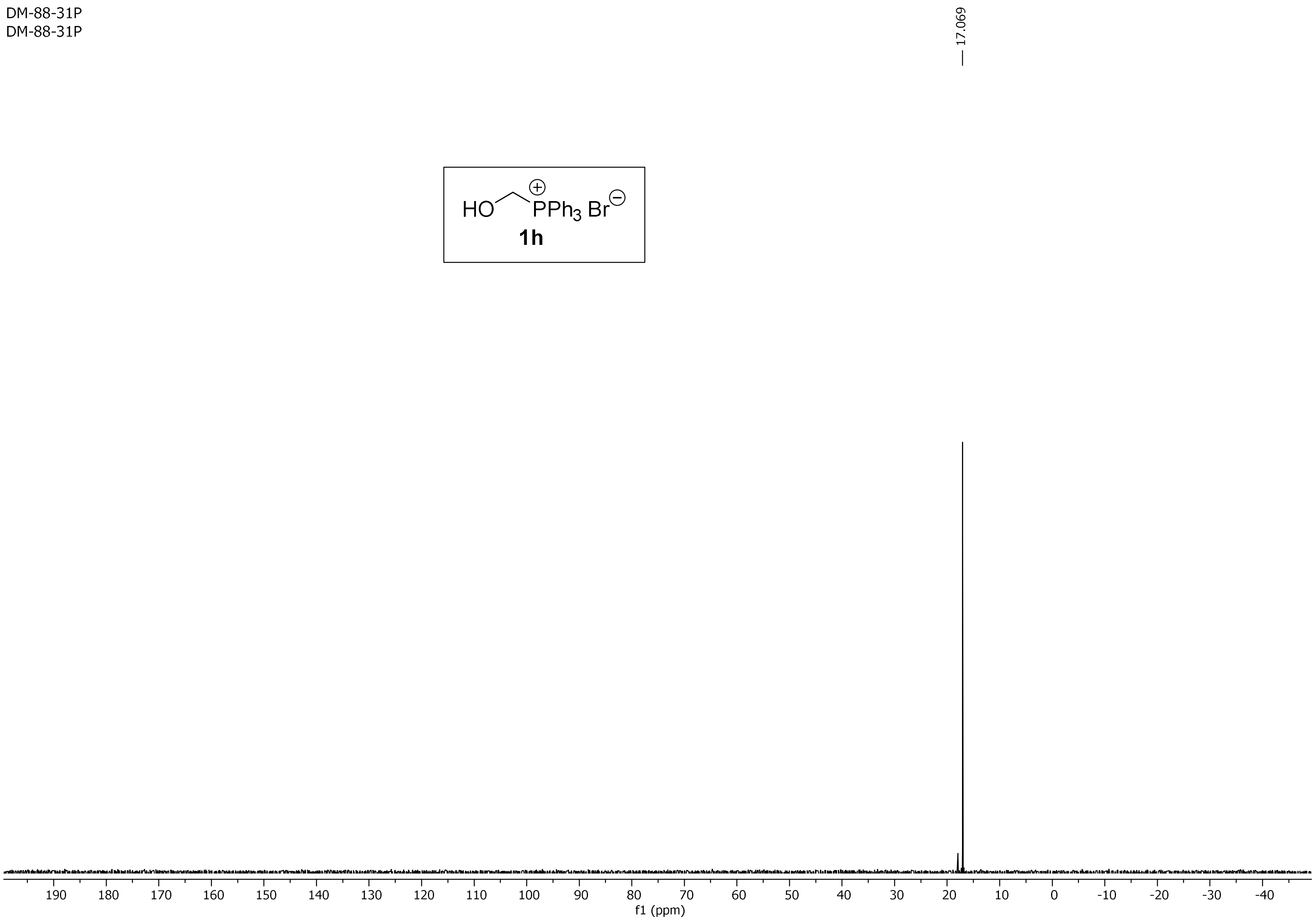

^{31}P NMR spectrum of hydroxymethyltriphenylphosphonium bromide (**1h**); 161.9 MHz/$CDCl_3$; δ (ppm).

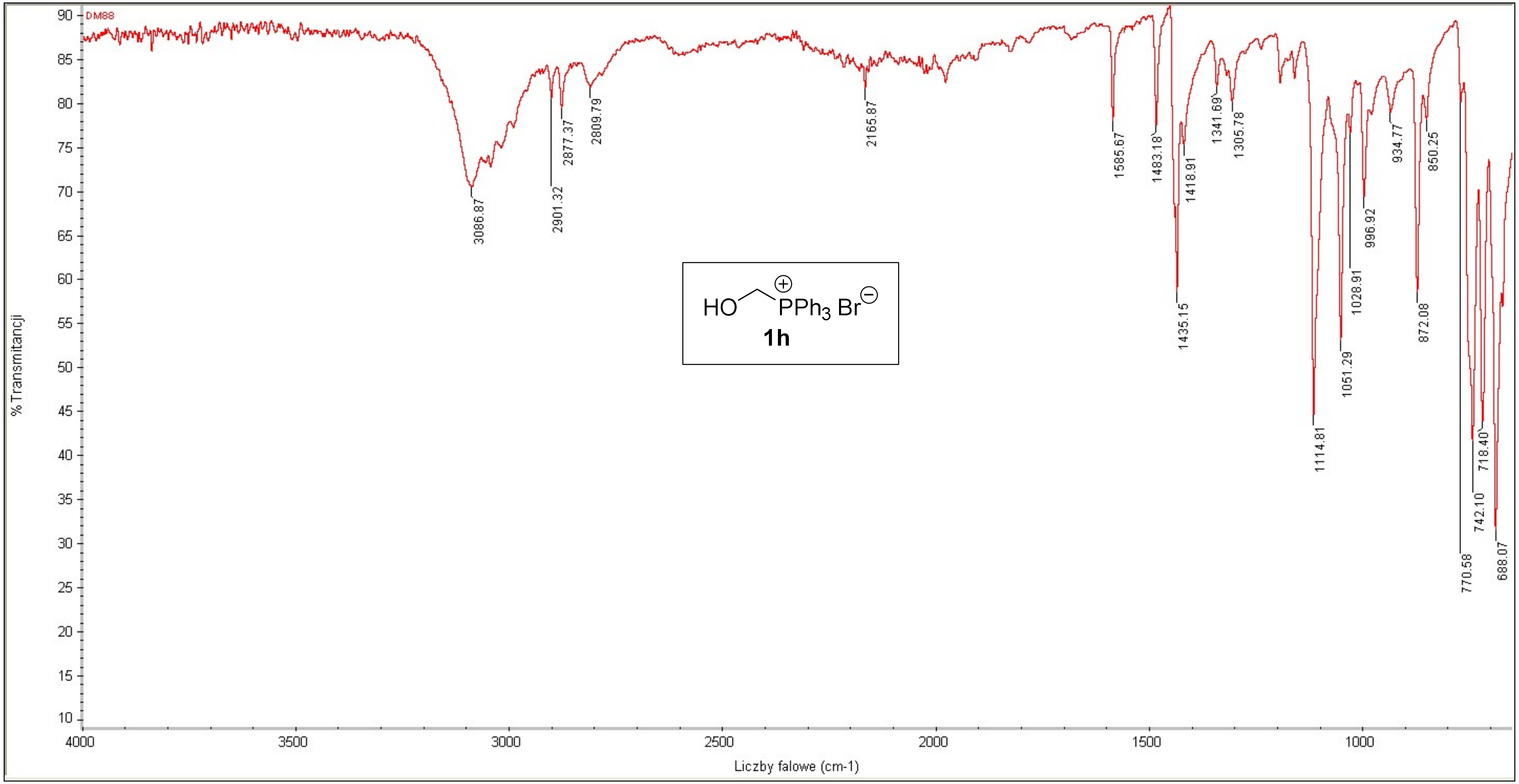

IR spectrum of hydroxymethyltriphenylphosphonium bromide (**1h**); ATR (cm^{-1}).

Tolerance = 40.0 mDa / DBE: min = -10.0, max = 100.0
Element prediction: Off
Number of isotope peaks used for i-FIT = 2
Monoisotopic Mass, Even Electron Ions
5 formula(e) evaluated with 1 results within limits (up to 3 closest results for each mass)
Elements Used:

Mass	RA	Calc. Mass	mDa	PPM	DBE	Formula	i-FIT	i-FIT Norm	Fit Conf %	C	H	O	P
293.1096	100.00	293.1095	0.1	0.3	11.5	C19 H18 O P	502.9	n/a	n/a	19	18	1	1

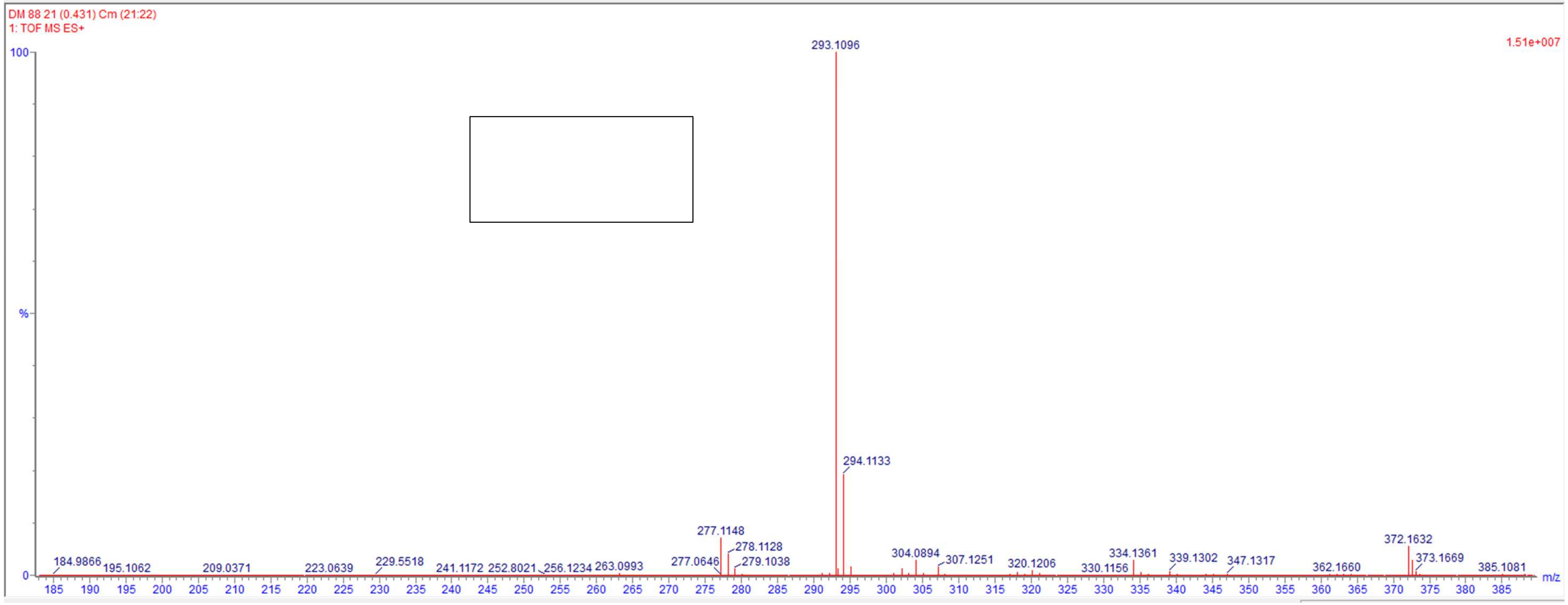

MS spectrum of hydroxymethyltriphenylphosphonium bromide (**1h**).

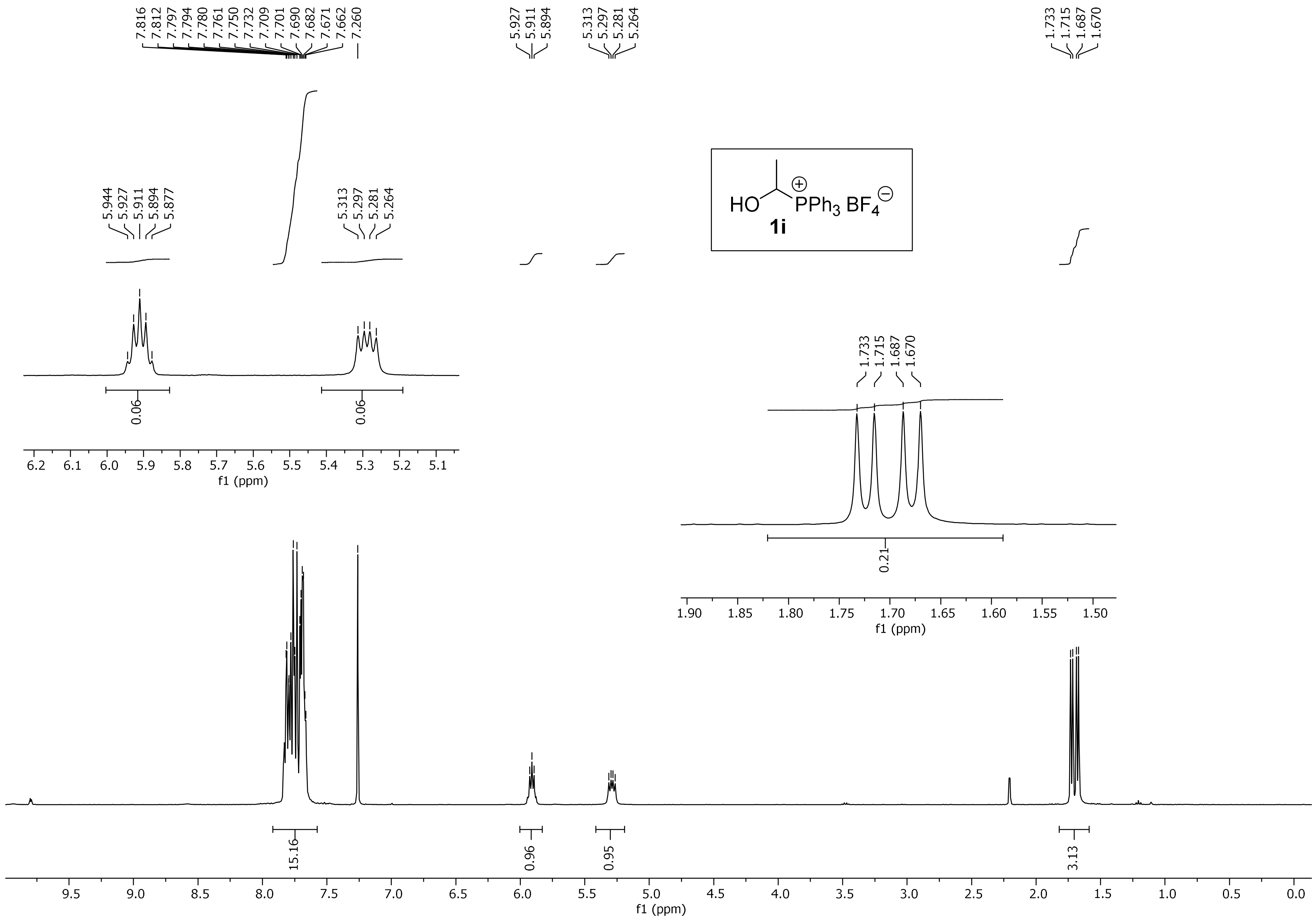

^{1}H NMR spectrum of 1-hydroxyethyltriphenylphosphonium tetrafluoroborate (**1i**); 400 MHz/$CDCl_3$/TMS; δ (ppm).

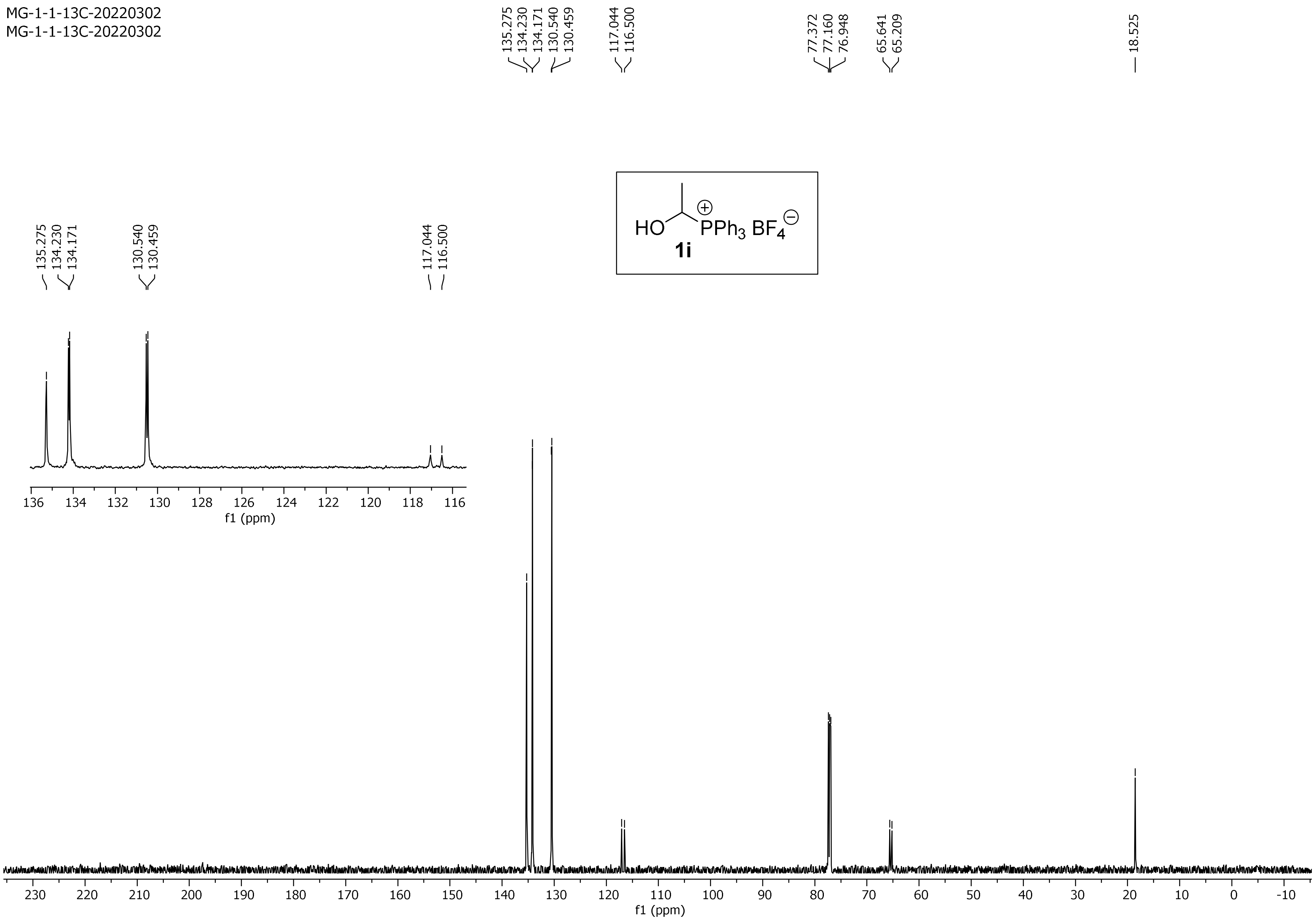

^{13}C NMR spectrum of 1-hydroxyethyltriphenylphosphonium tetrafluoroborate (**1i**); 150 MHz/$CDCl_3$/TMS; *δ* (ppm).

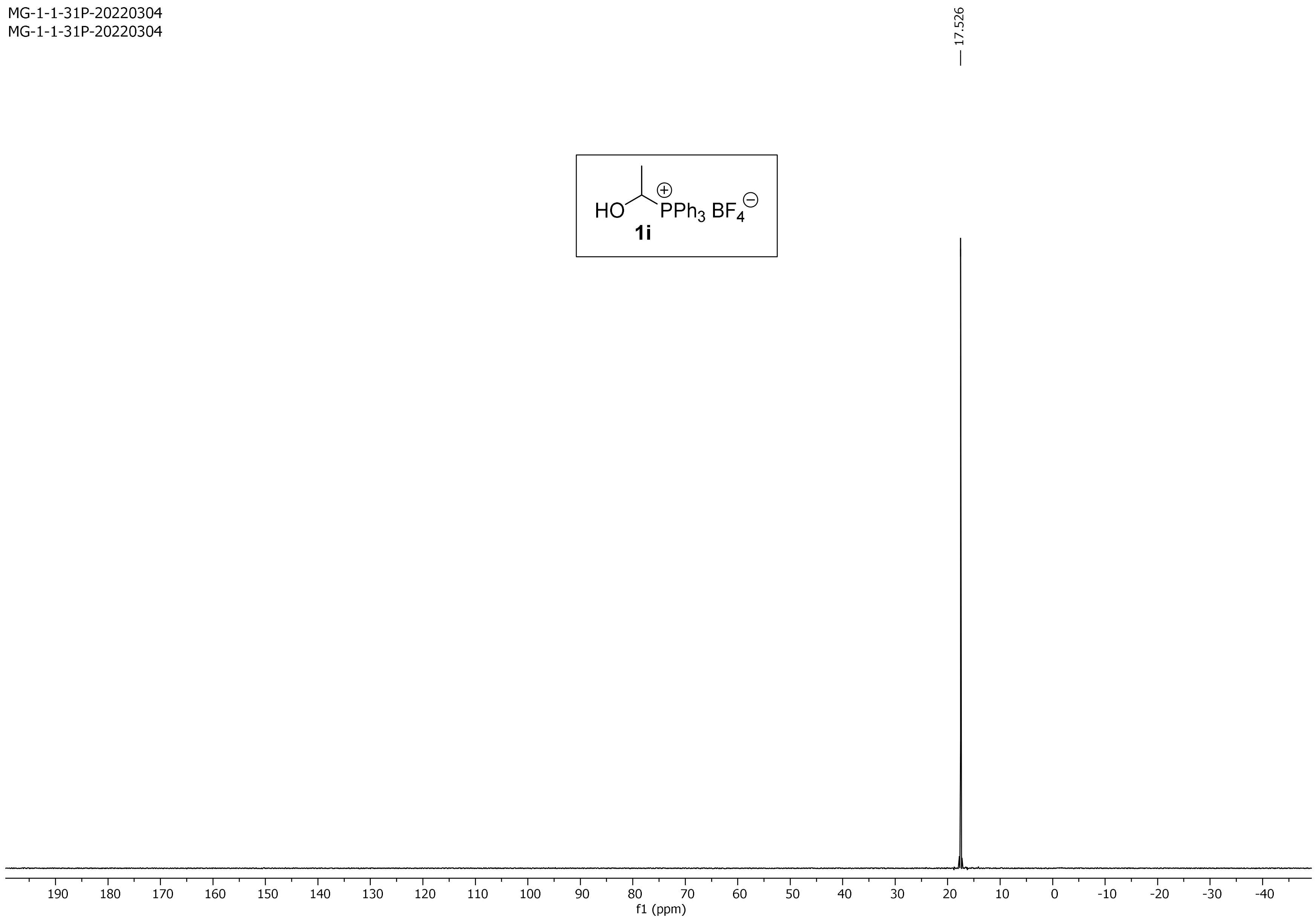

^{31}P NMR spectrum of 1-hydroxyethyltriphenylphosphonium tetrafluoroborate (**1i**); 161.9 MHz/$CDCl_3$; δ (ppm).

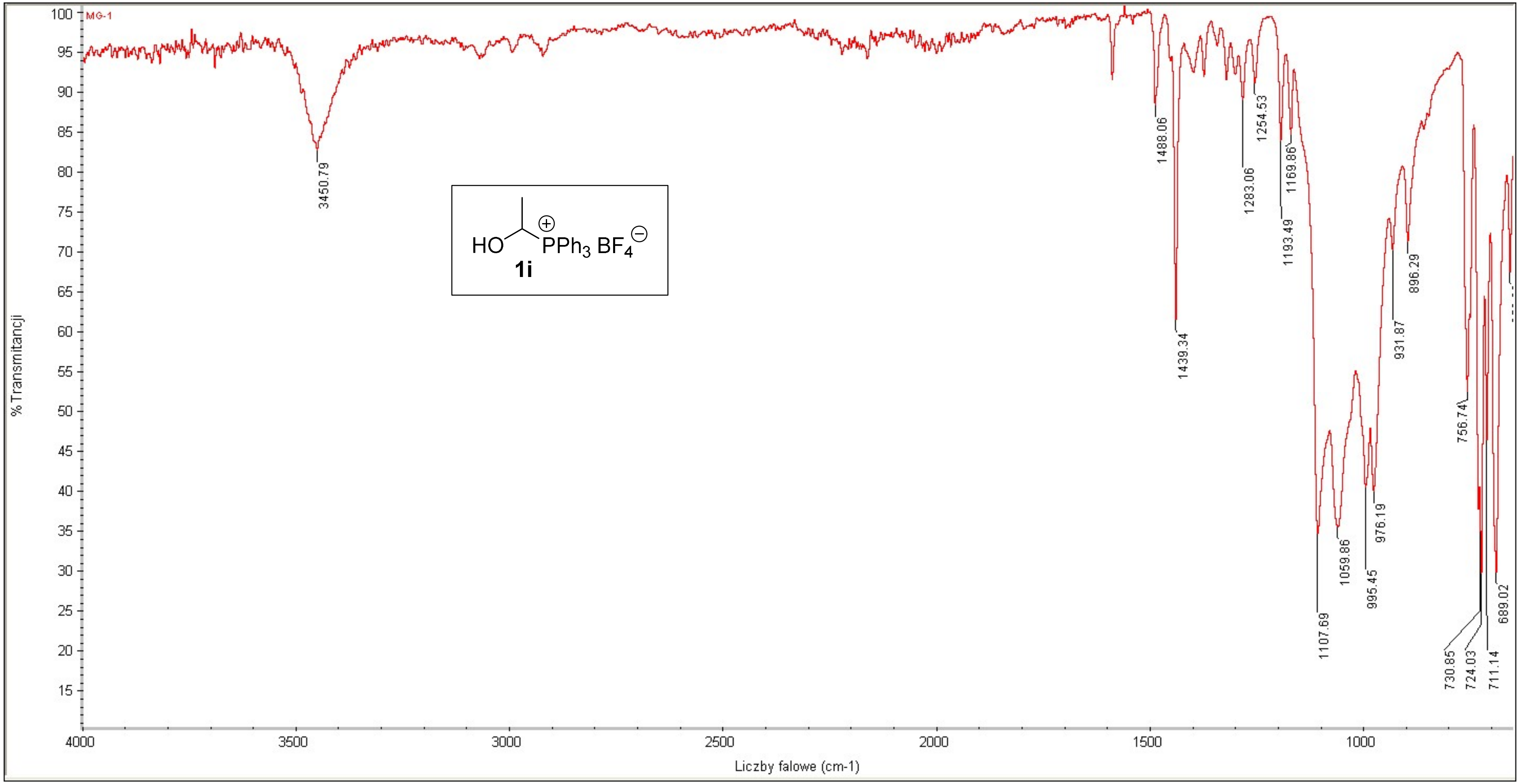

IR spectrum of 1-hydroxyethyltriphenylphosphonium tetrafluoroborate (**1i**); ATR (cm^{-1}).

Tolerance = 40.0 mDa / DBE: min = -10.0, max = 100.0
Element prediction: Off
Number of isotope peaks used for i-FIT = 2
Monoisotopic Mass, Even Electron Ions
5 formula(e) evaluated with 1 results within limits (all results (up to 1000) for each mass)
Elements Used:

Mass	RA	Calc. Mass	mDa	PPM	DBE	Formula	i-FIT	i-FIT Norm	Fit Conf %	C	H	O	P
307.1251	100.00	307.1252	-0.1	-0.3	11.5	C20 H20 O P	589.5	n/a	n/a	20	20	1	1

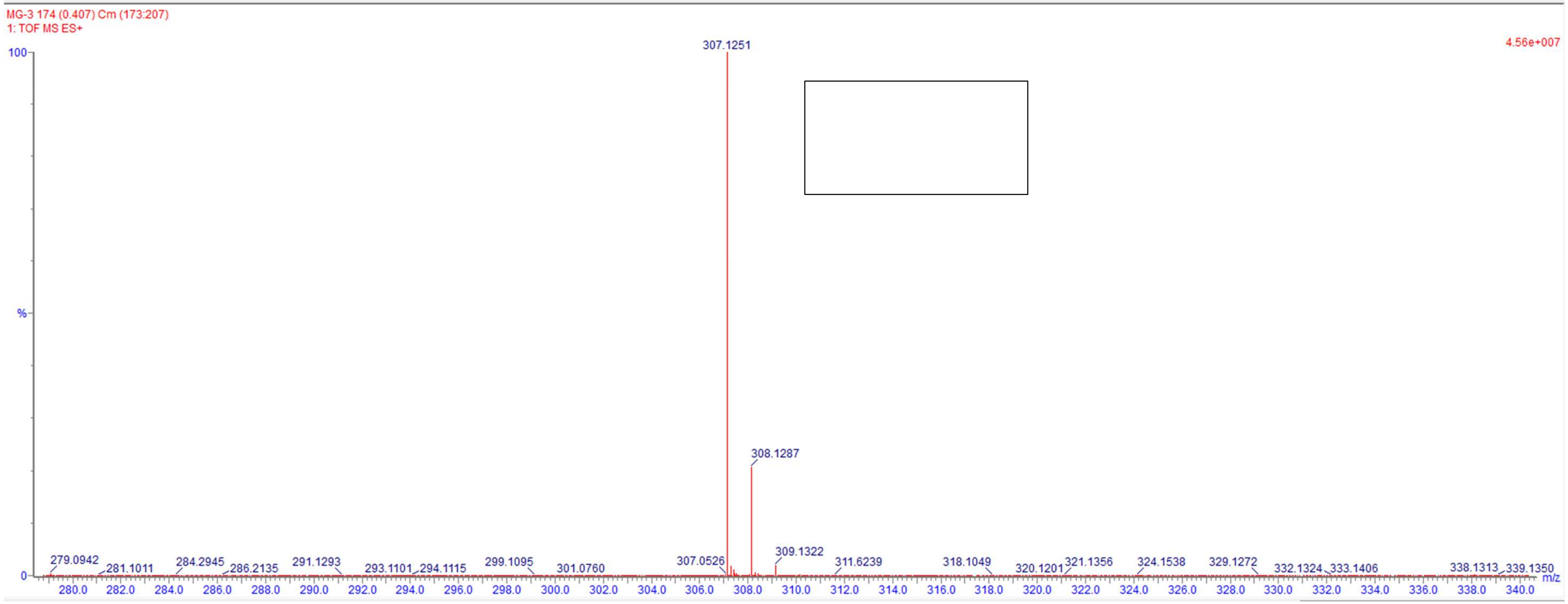

MS spectrum of 1-hydroxyethyltriphenylphosphonium tetrafluoroborate (**1i**).

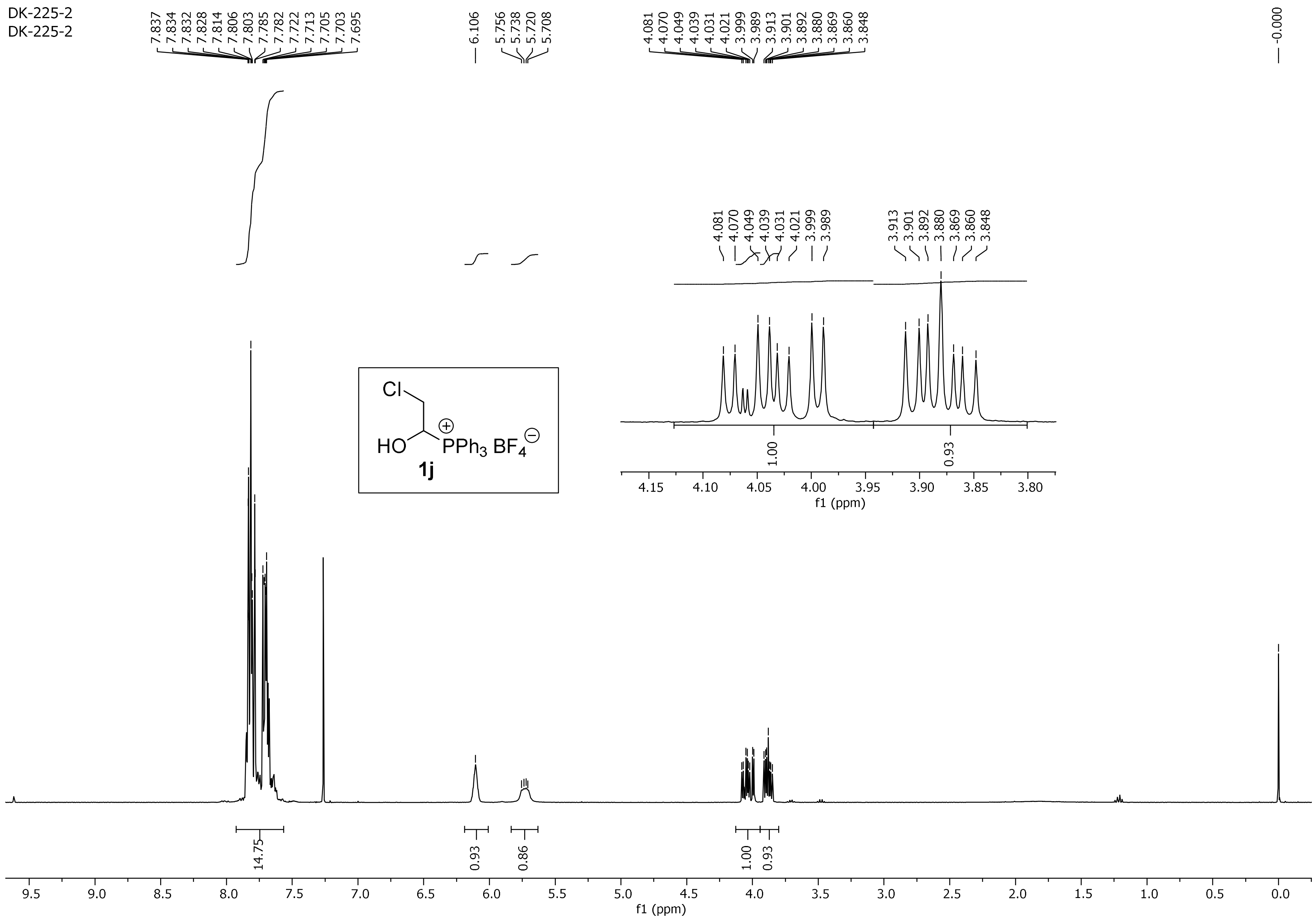

^{1}H NMR spectrum of 2-chloro-1-hydroxyethyltriphenylphosphonium tetrafluoroborate (**1j**); 400 MHz/$CDCl_3$/TMS; δ (ppm).

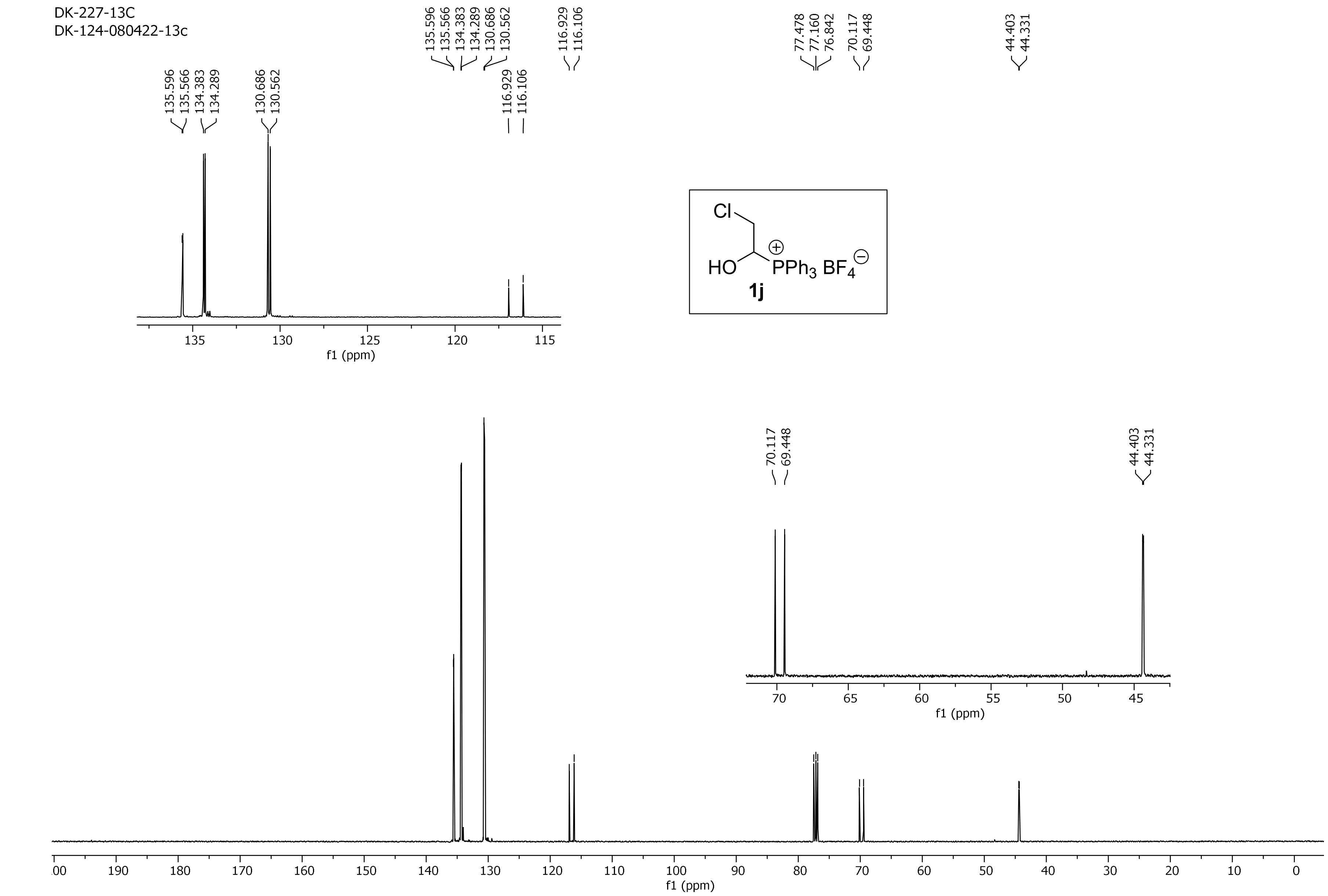

^{13}C NMR spectrum of 2-chloro-1-hydroxyethyltriphenylphosphonium tetrafluoroborate (**1j**); 100 MHz/$CDCl_3$/TMS; δ (ppm).

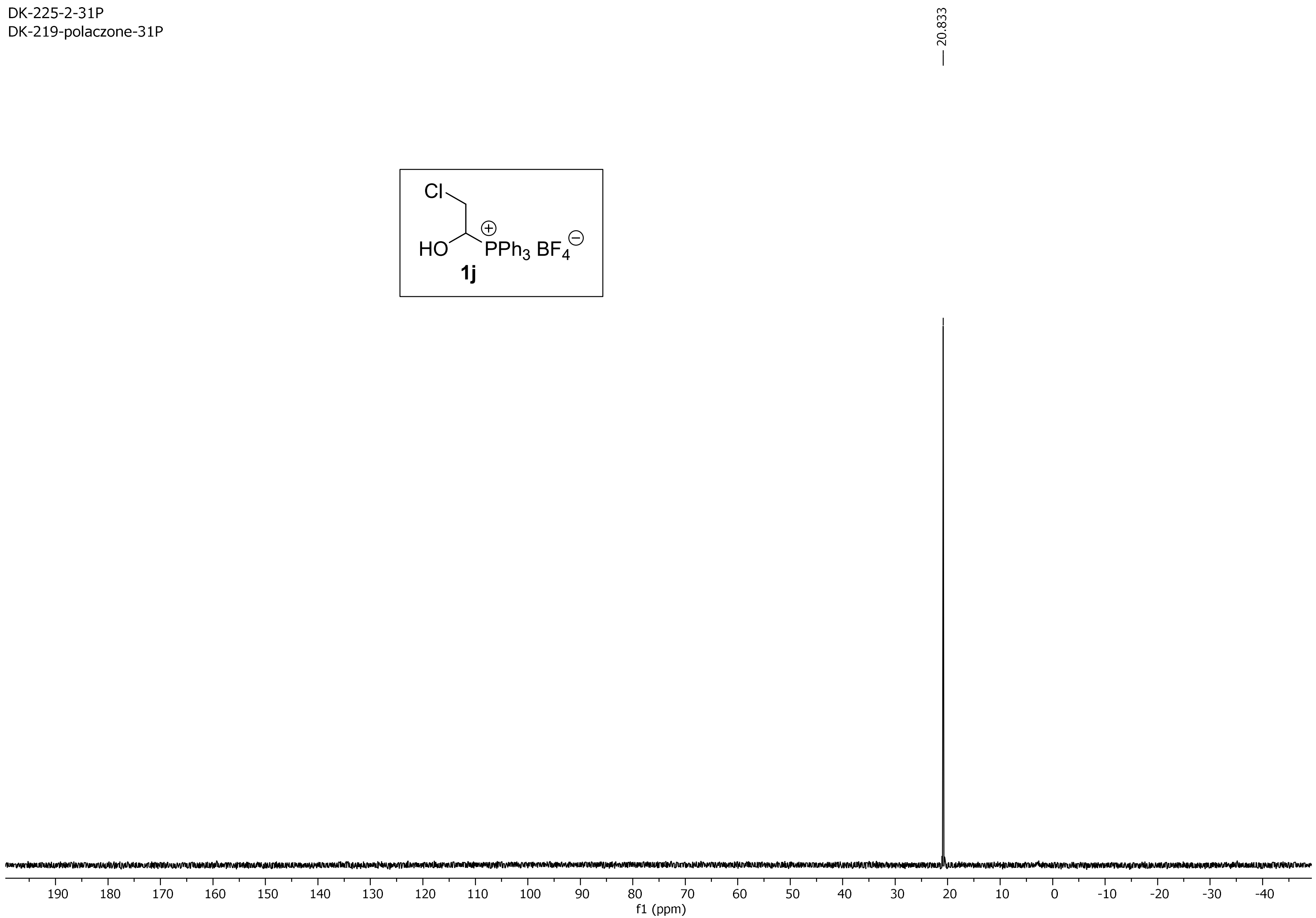

^{31}P NMR spectrum of 2-chloro-1-hydroxyethyltriphenylphosphonium tetrafluoroborate (**1j**); 161.9 MHz/$CDCl_3$; δ (ppm).

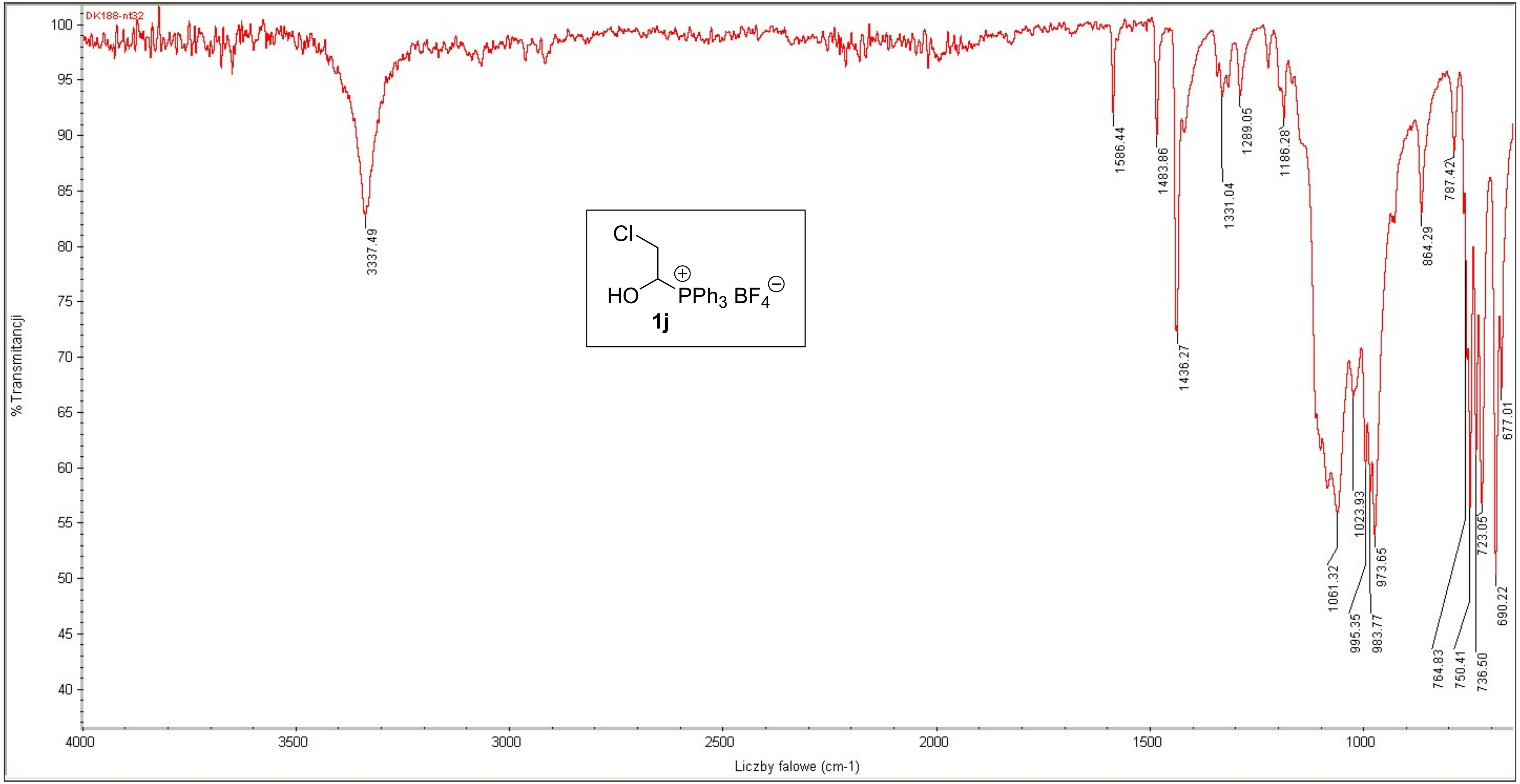

IR spectrum of 2-chloro-1-hydroxyethyltriphenylphosphonium tetrafluoroborate (**1j**); ATR (cm^{-1}).

Tolerance = 40.0 mDa / DBE: min = -10.0, max = 100.0
Element prediction: Off
Number of isotope peaks used for i-FIT = 2
Monoisotopic Mass, Even Electron Ions
5 formula(e) evaluated with 1 results within limits (all results (up to 1000) for each mass)
Elements Used:

Mass	RA	Calc. Mass	mDa	PPM	DBE	Formula	i-FIT	i-FIT Norm	Fit Conf %	C	H	O	P	Cl
341.0861	100.00	341.0862	-0.1	-0.3	11.5	C20 H19 O P Cl	401.9	n/a	n/a	20	19	1	1	1

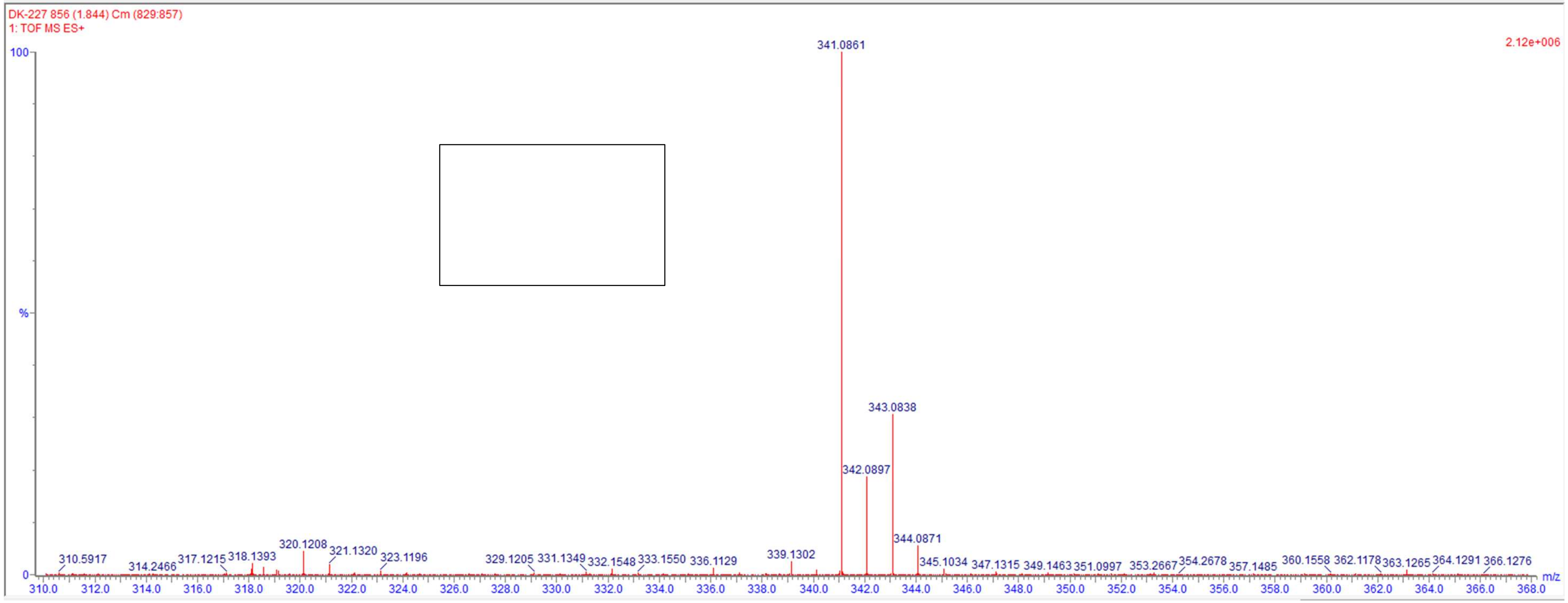

MS spectrum of 2-chloro-1-hydroxyethyltriphenylphosphonium tetrafluoroborate (**1j**).

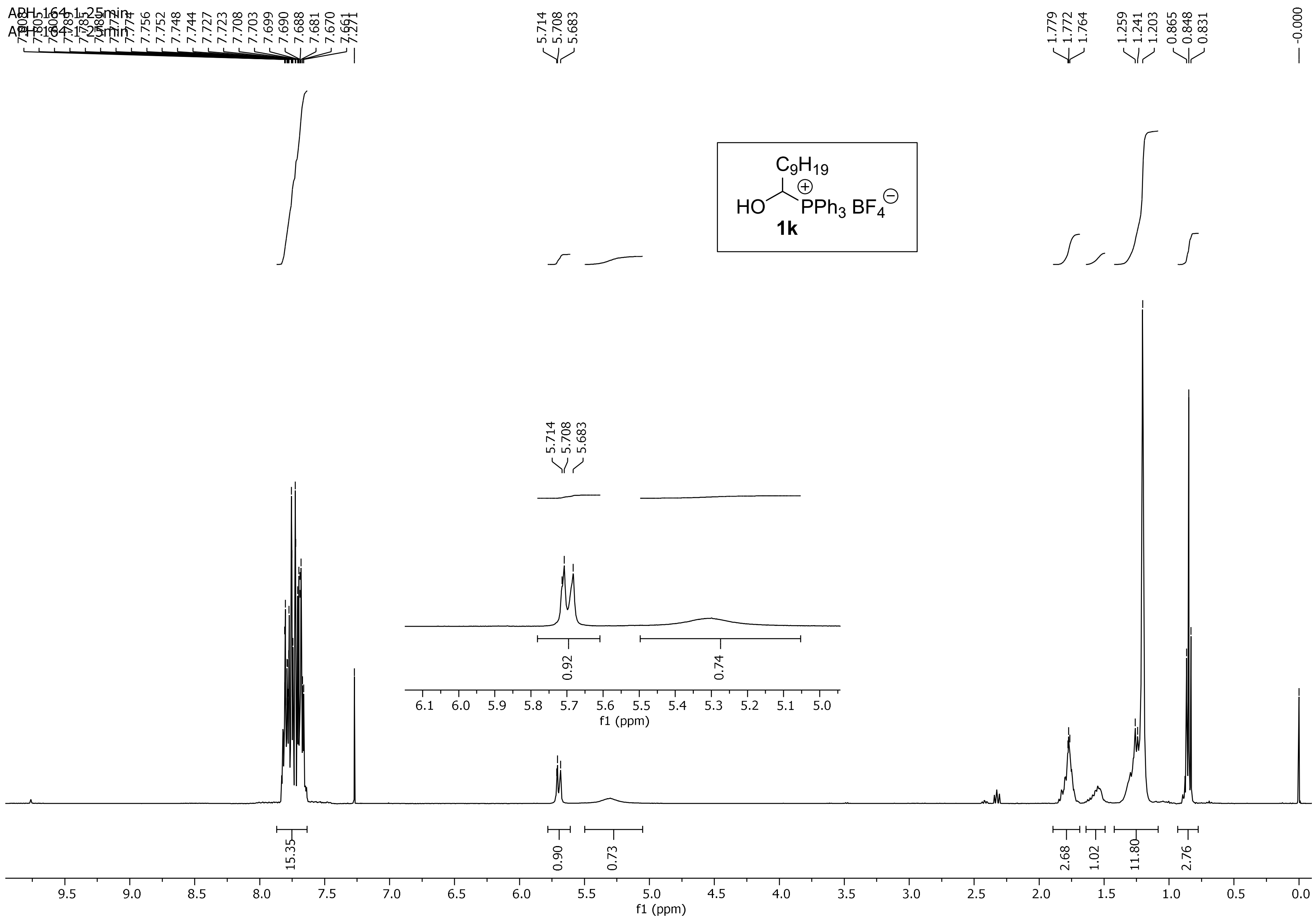

^{1}H NMR spectrum of 1-hydroxydecyltriphenylphosphonium tetrafluoroborate (**1k**); 400 MHz/$CDCl_3$/TMS; δ (ppm).

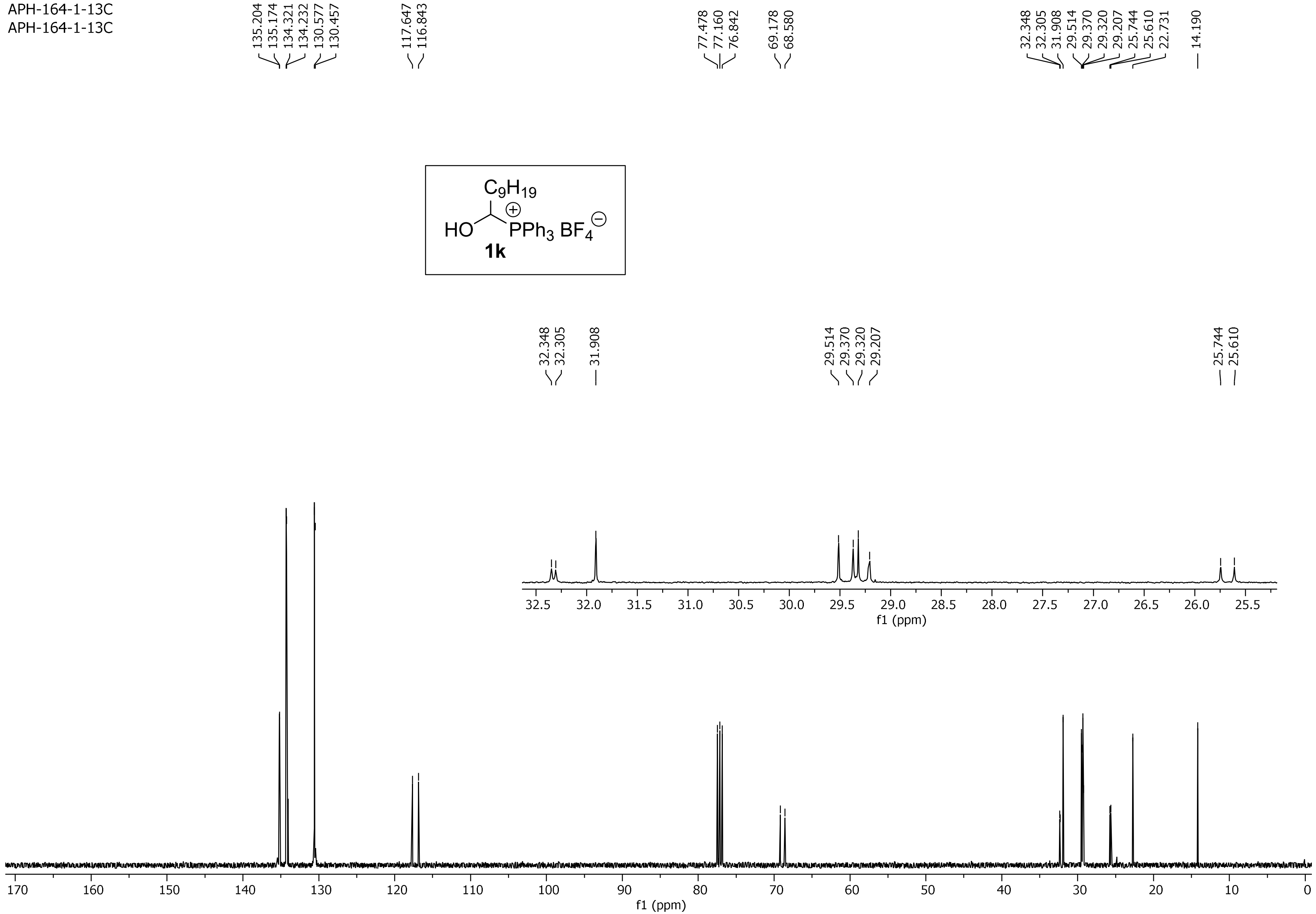

^{13}C NMR spectrum of 1-hydroxydecyltriphenylphosphonium tetrafluoroborate (**1k**); 100 MHz/CDCl$_3$/TMS; δ (ppm).

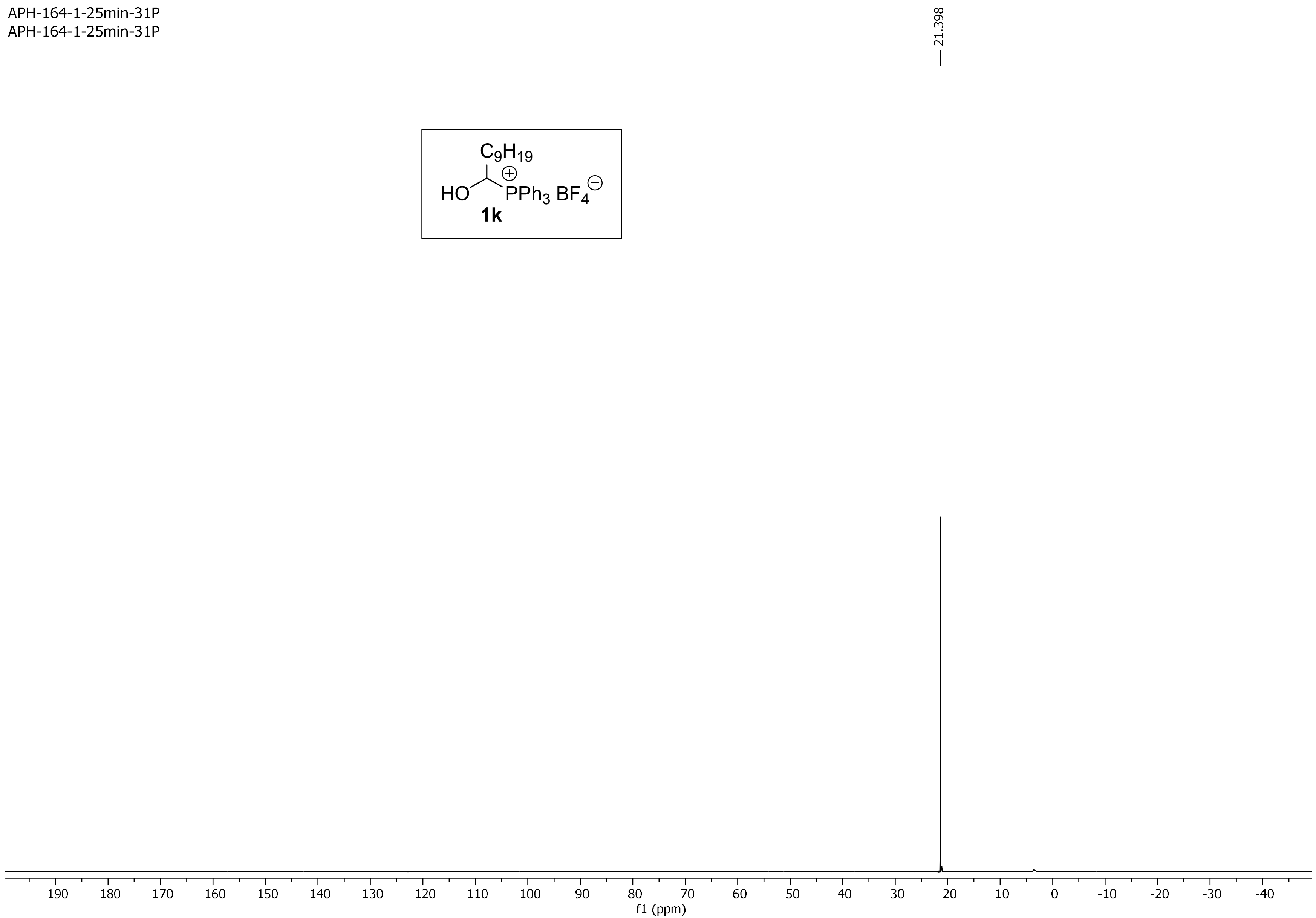

^{31}P NMR spectrum of 1-hydroxydecyltriphenylphosphonium tetrafluoroborate (**1k**); 161.9 MHz/$CDCl_3$; δ (ppm).

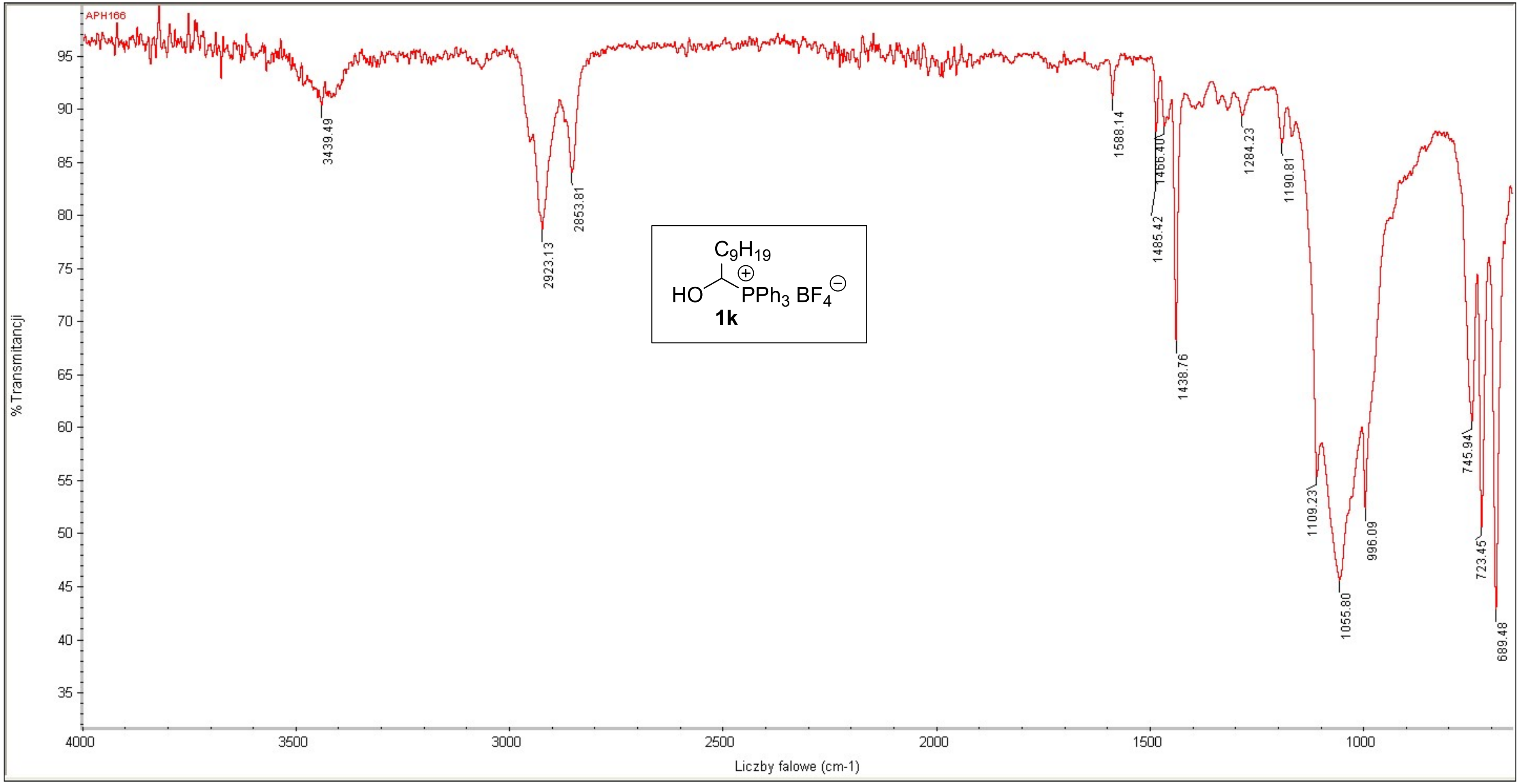

IR spectrum of 1-hydroxydecyltriphenylphosphonium tetrafluoroborate (**1k**); ATR (cm^{-1}).

Tolerance = 40.0 mDa / DBE: min = -10.0, max = 100.0
Element prediction: Off
Number of isotope peaks used for i-FIT = 2
Monoisotopic Mass, Even Electron Ions
7 formula(e) evaluated with 1 results within limits (all results (up to 1000) for each mass)
Elements Used:

Mass	RA	Calc. Mass	mDa	PPM	DBE	Formula	i-FIT	i-FIT Norm	Fit Conf %	C	H	O	P
419.2499	100.00	419.2504	-0.5	-1.2	11.5	C28 H36 O P	628.6	n/a	n/a	28	36	1	1

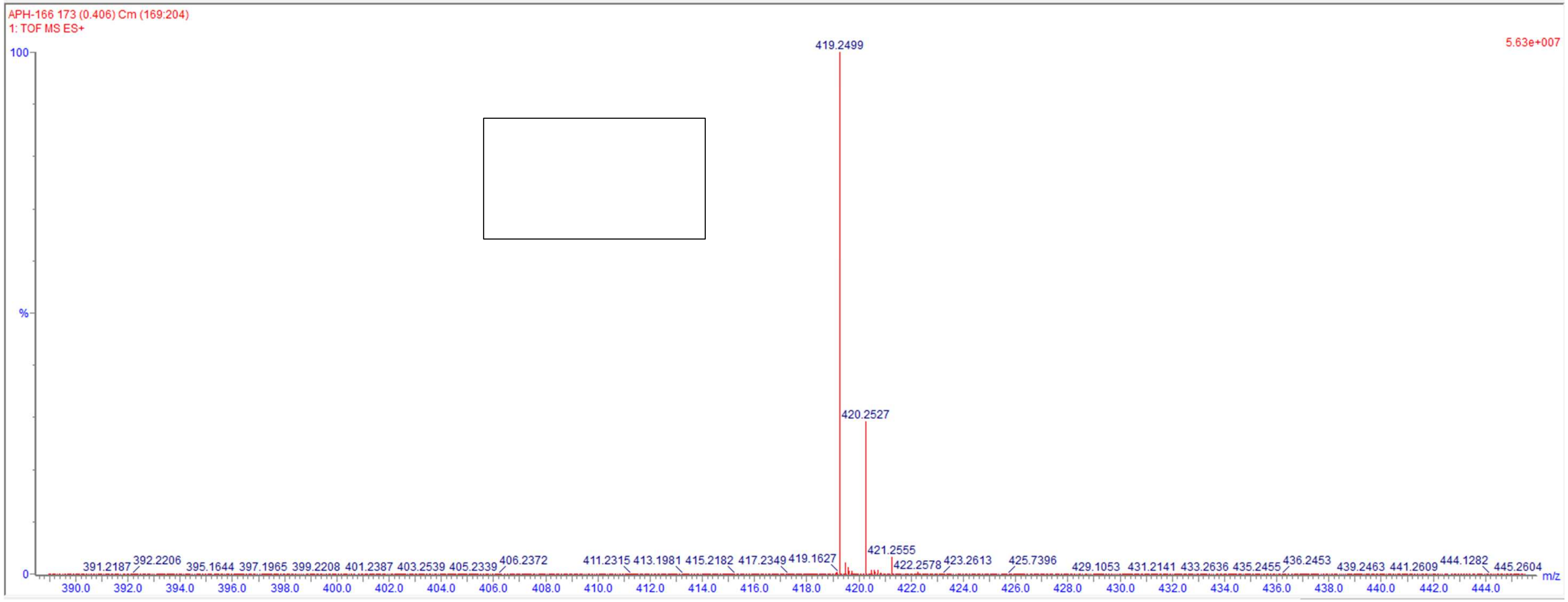

MS spectrum of 1-hydroxydecyltriphenylphosphonium tetrafluoroborate (**1k**).

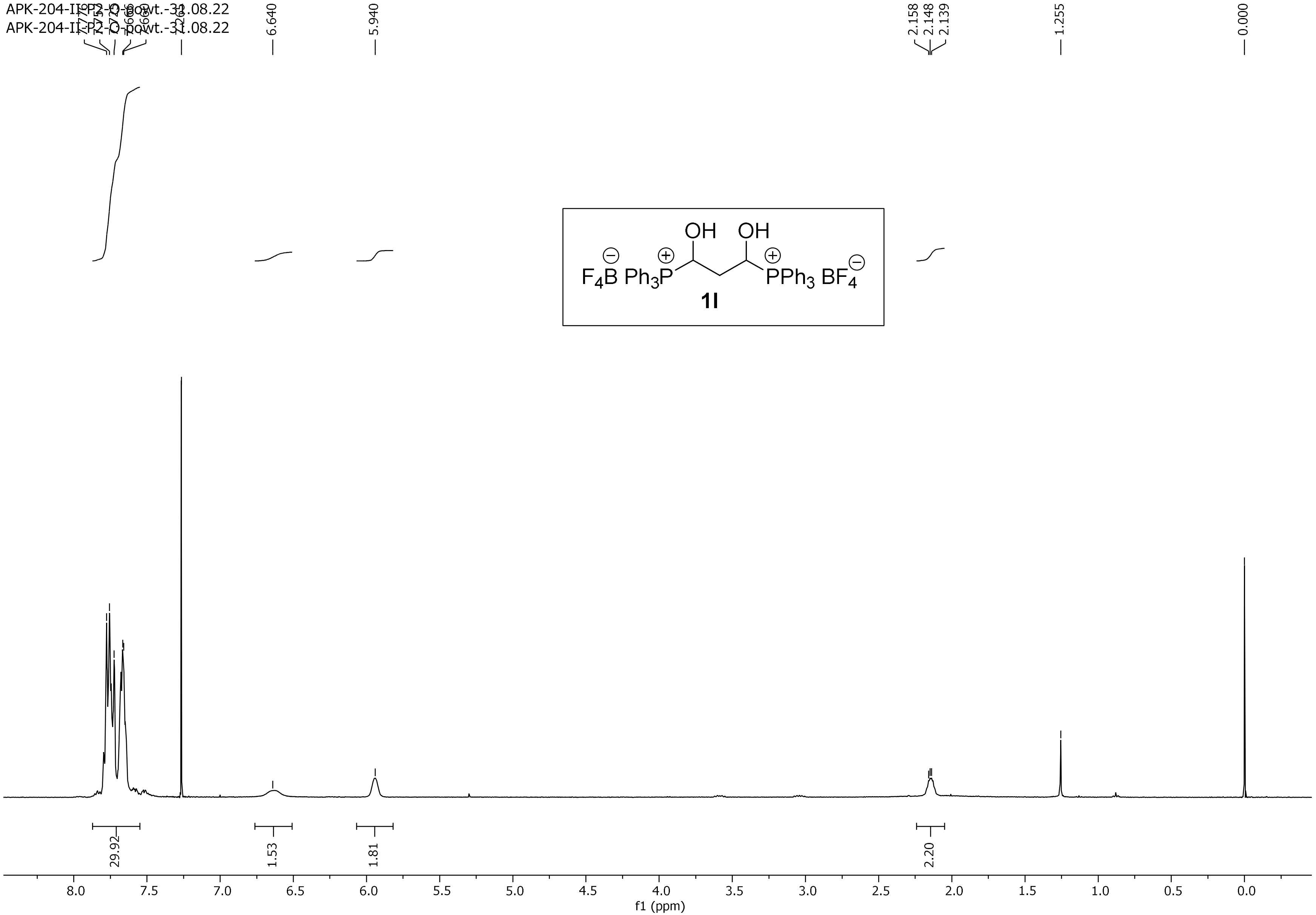

^{1}H NMR spectrum of 1,3-dihydroxypropane-1,3-bis(triphenylphosphonium) bis(tetrafluoroborate) (**1l**); 400 MHz/$CDCl_3$/TMS; δ (ppm).

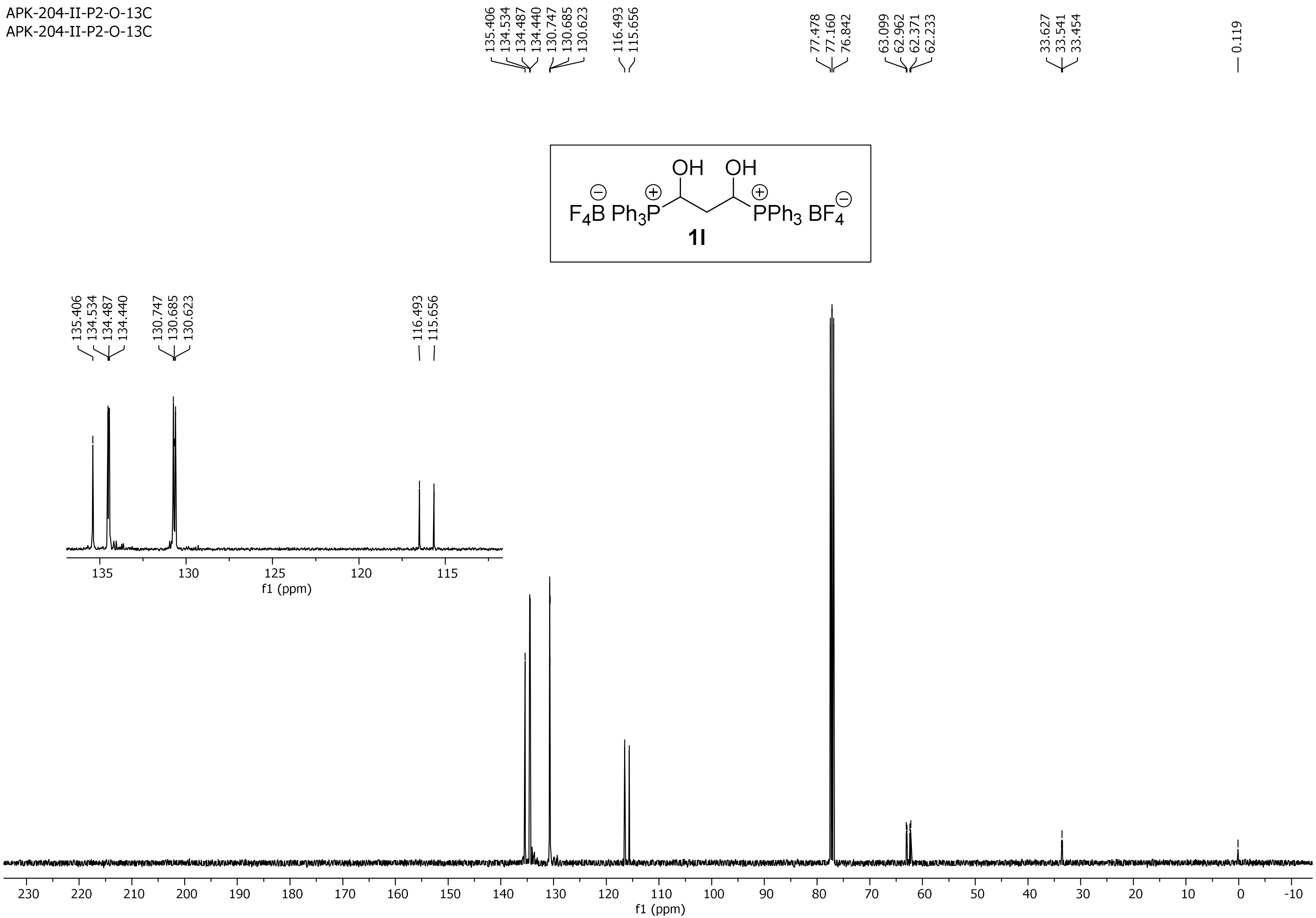

^{13}C NMR spectrum of 1,3-dihydroxypropane-1,3-bis(triphenylphosphonium) bis(tetrafluoroborate) (**1l**); 100 MHz/$CDCl_3$/TMS; δ (ppm).

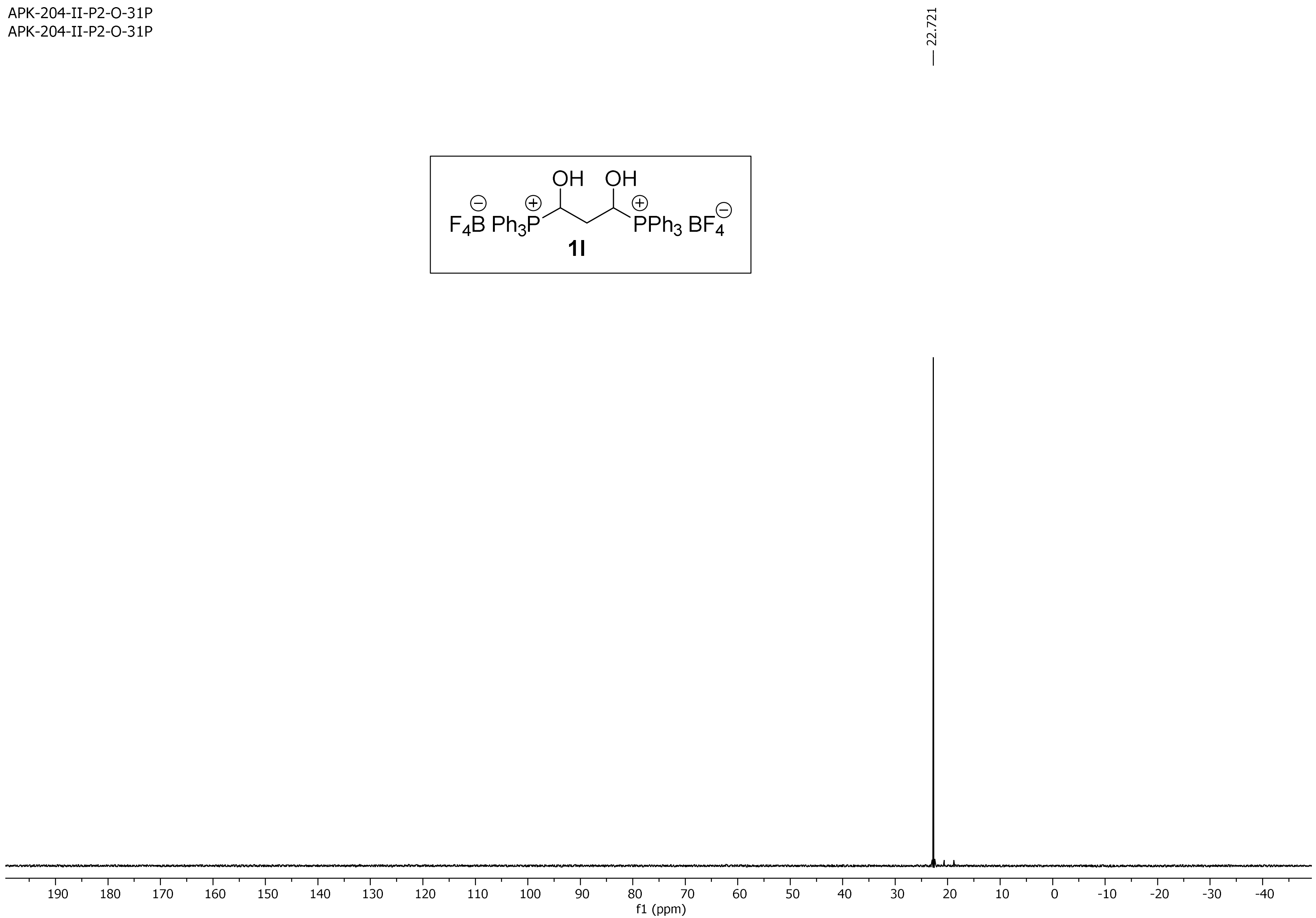

^{31}P NMR spectrum of 1,3-dihydroxypropane-1,3-bis(triphenylphosphonium) bis(tetrafluoroborate) (**1l**); 161.9 MHz/$CDCl_3$; δ (ppm).

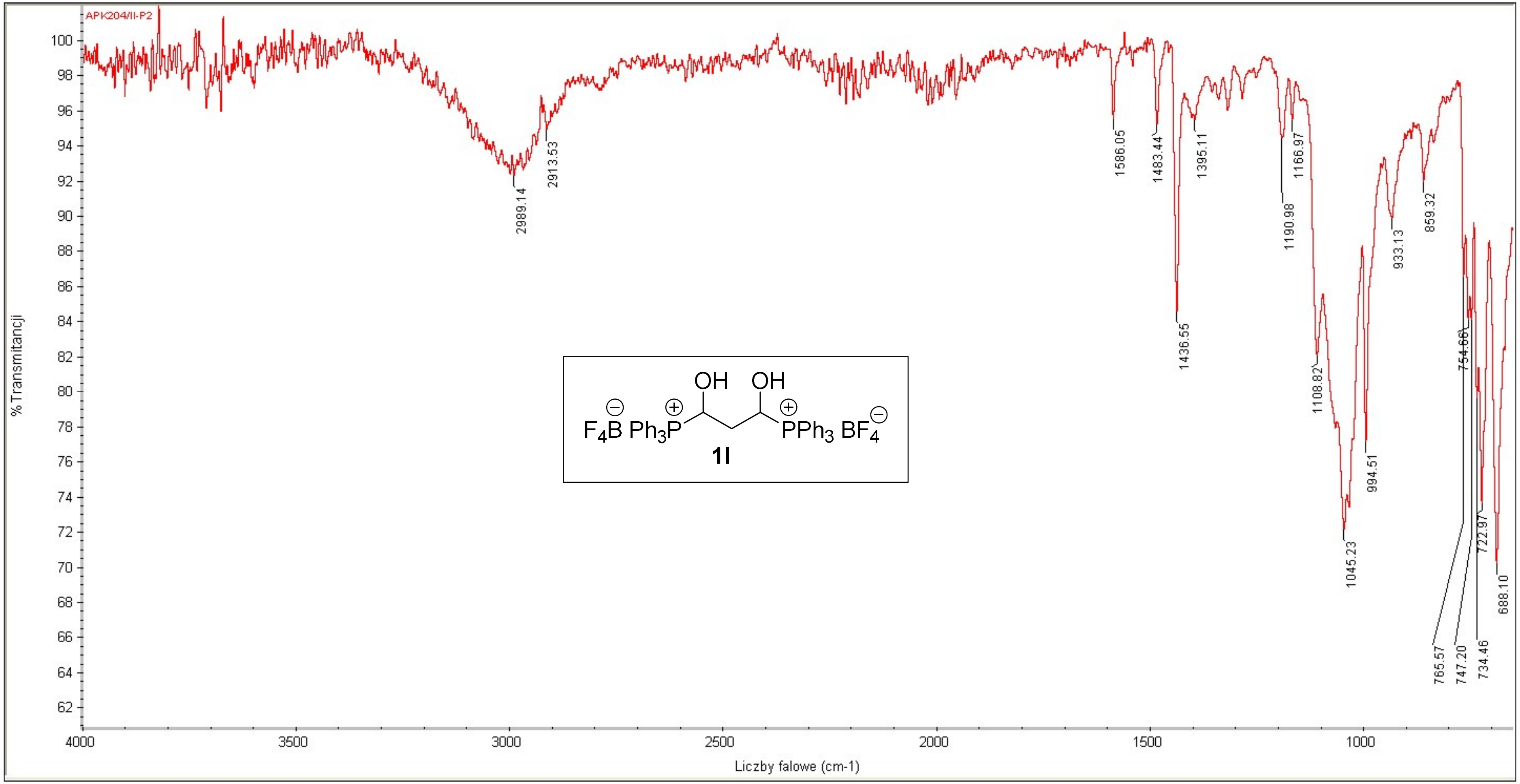

IR spectrum of 1,3-dihydroxypropane-1,3-bis(triphenylphosphonium) bis(tetrafluoroborate) (**1l**); ATR (cm^{-1}).

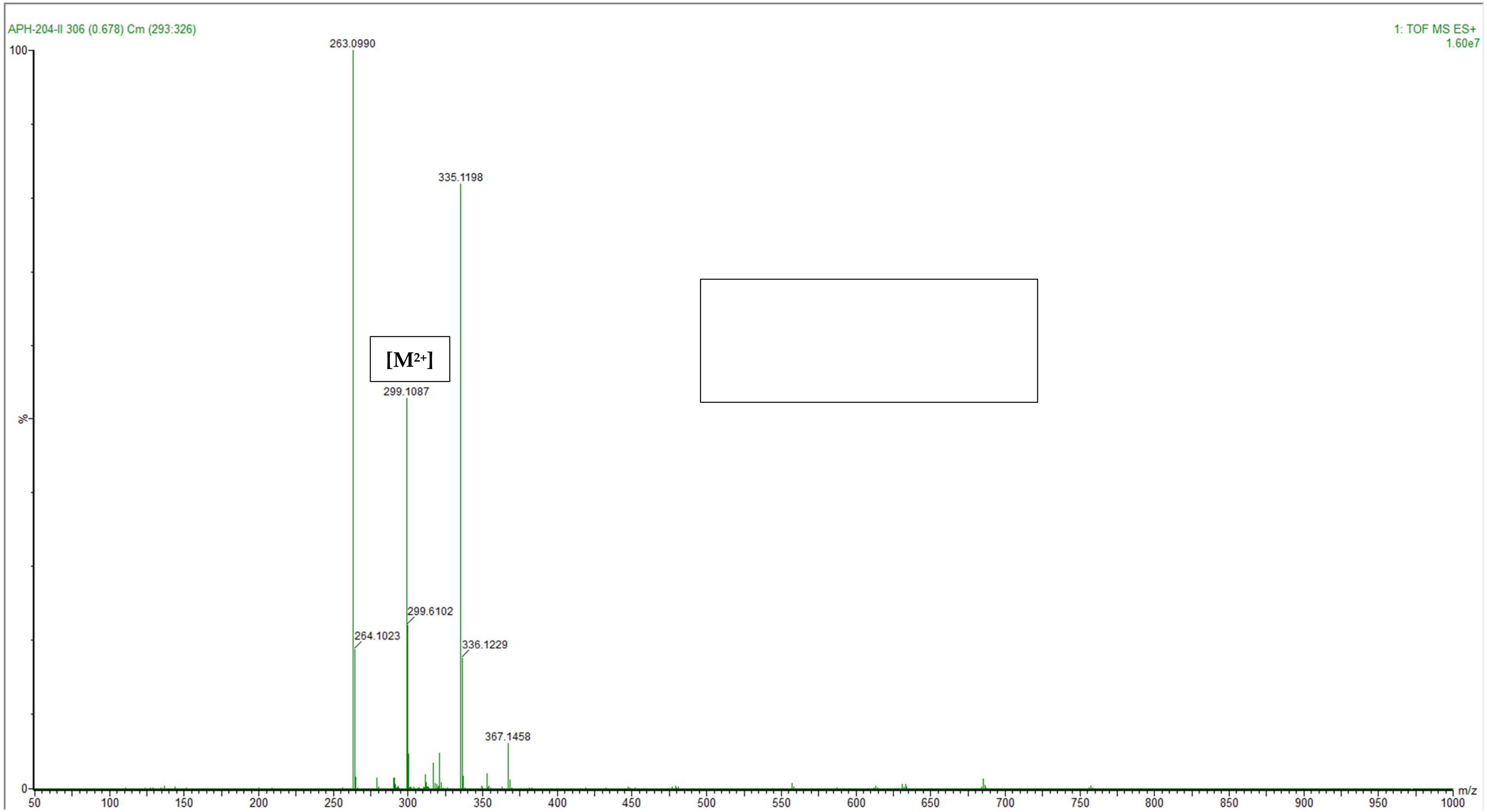

MS spectrum of 1,3-dihydroxypropane-1,3-bis(triphenylphosphonium) bis(tetrafluoroborate) (**1l**).

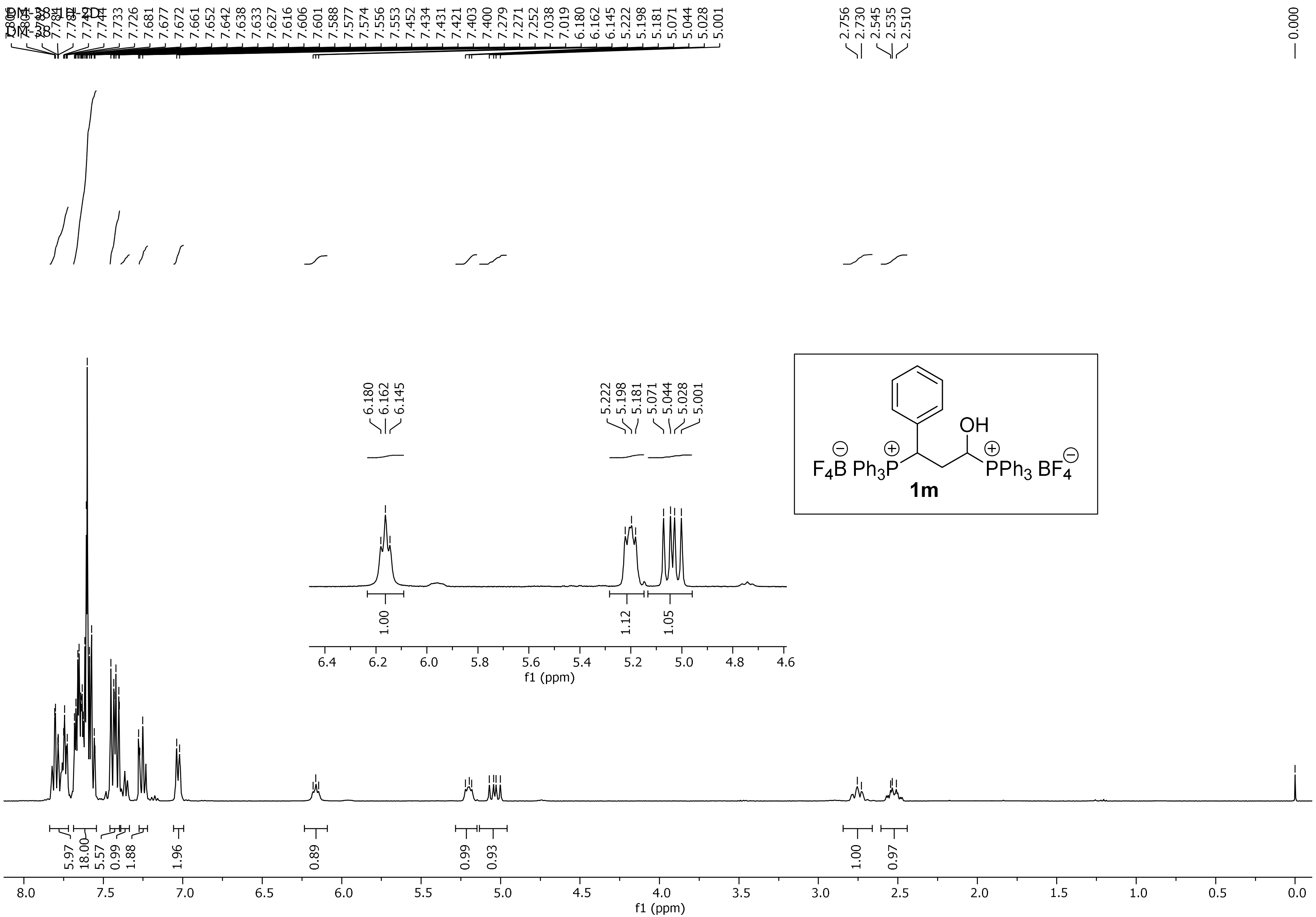

^{1}H NMR spectrum of 1-hydroxy-3-phenylpropane-1,3-bis(triphenylphosphonium) bis(tetrafluoroborate) (**1m**); 400 MHz/$CDCl_3$/TMS; δ (ppm).

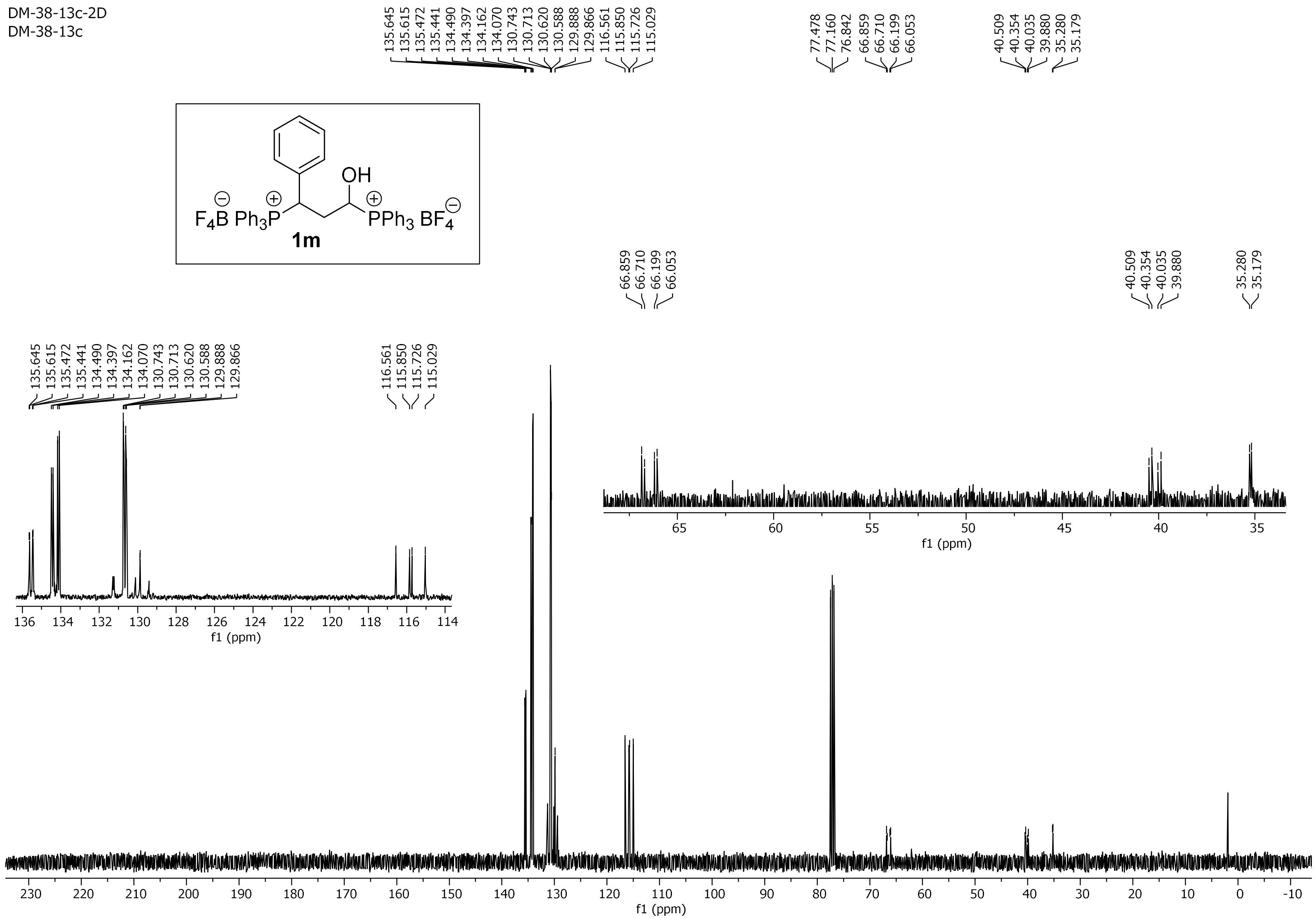

^{13}C NMR spectrum of 1-hydroxy-3-phenylpropane-1,3-bis(triphenylphosphonium) bis(tetrafluoroborate) (**1m**); 100 MHz/$CDCl_3$/TMS; δ (ppm).

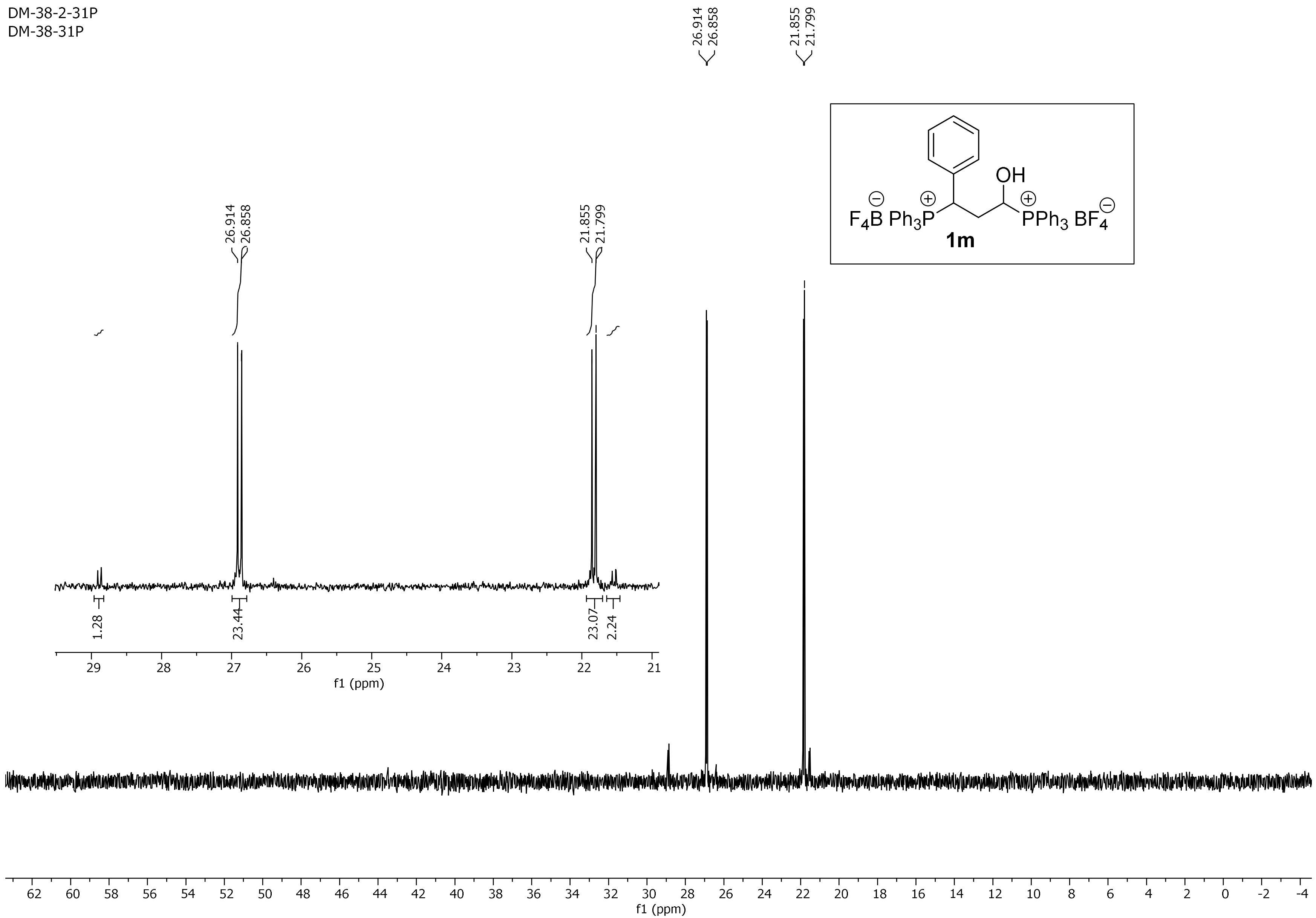

^{31}P NMR spectrum of 1-hydroxy-3-phenylpropane-1,3-bis(triphenylphosphonium) bis(tetrafluoroborate) (**1m**); 161.9 MHz/$CDCl_3$; δ (ppm).

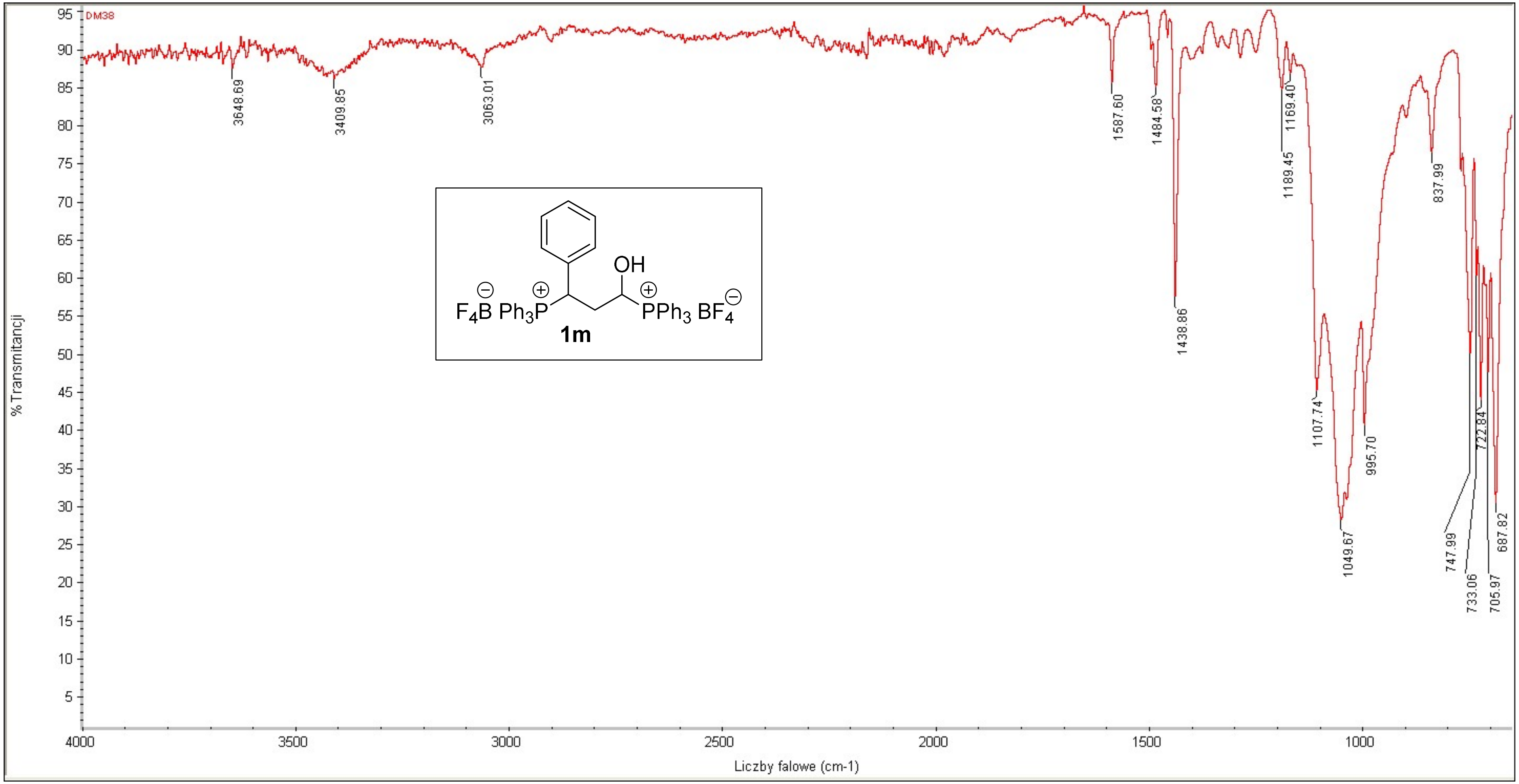

IR spectrum of 1-hydroxy-3-phenylpropane-1,3-bis(triphenylphosphonium) bis(tetrafluoroborate) (**1m**); ATR (cm^{-1}).

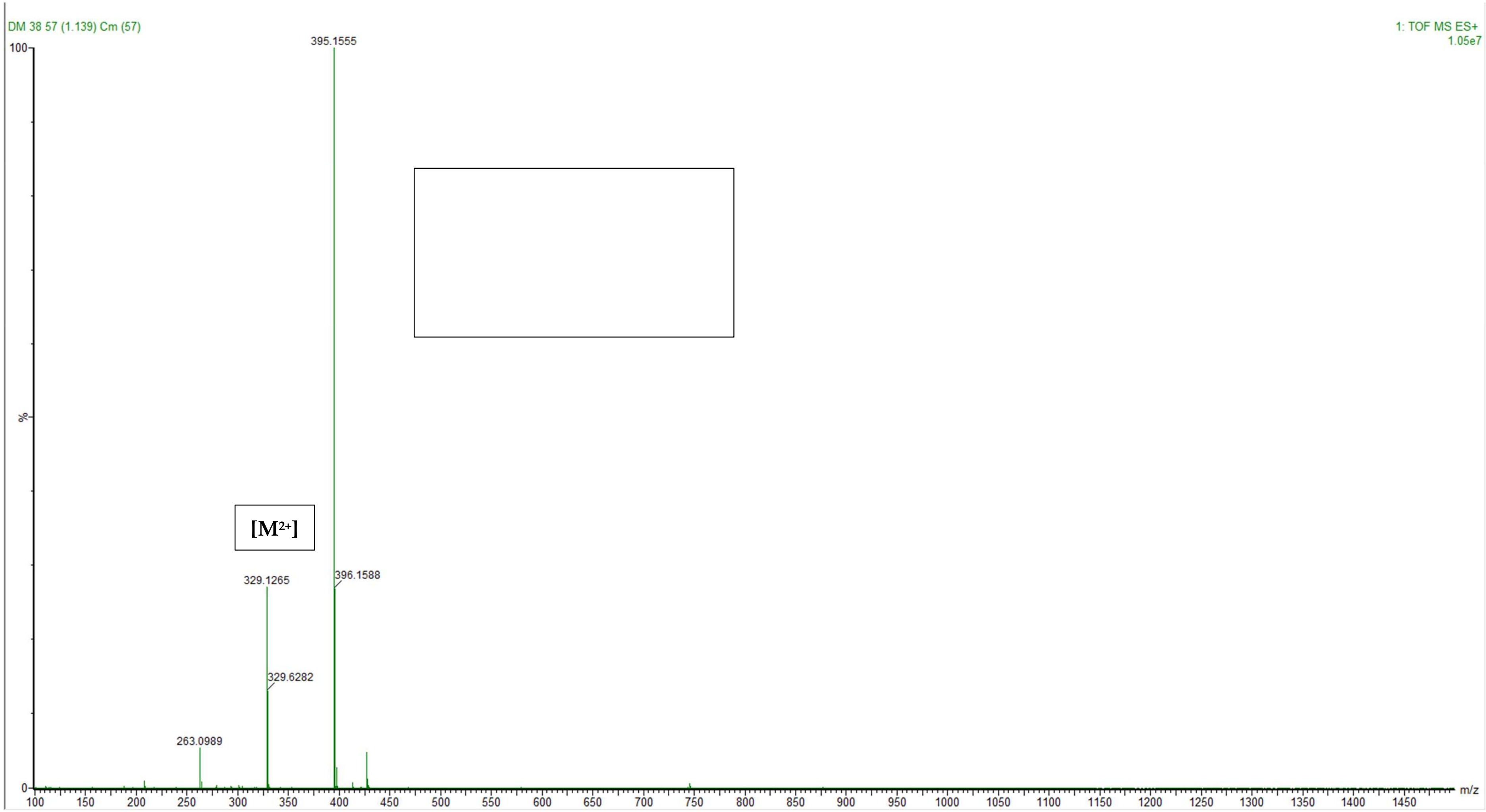

MS spectrum of 1-hydroxy-3-phenylpropane-1,3-bis(triphenylphosphonium) bis(tetrafluoroborate) (**1m**).

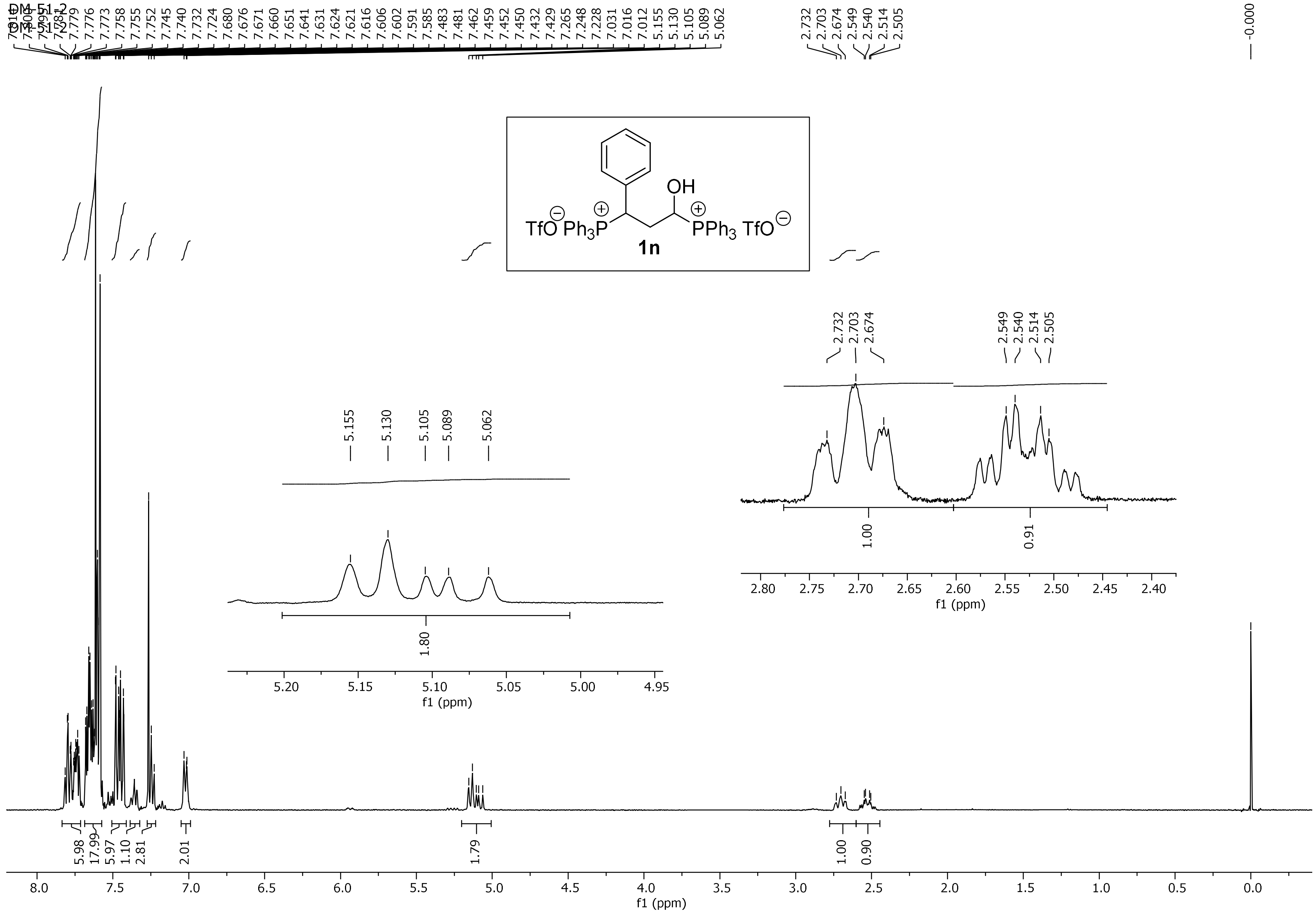

^{1}H NMR spectrum of 1-hydroxy-3-phenylpropane-1,3-bis(triphenylphosphonium) bis(triflate) (**1n**); 400 MHz/$CDCl_3$/TMS; δ (ppm).

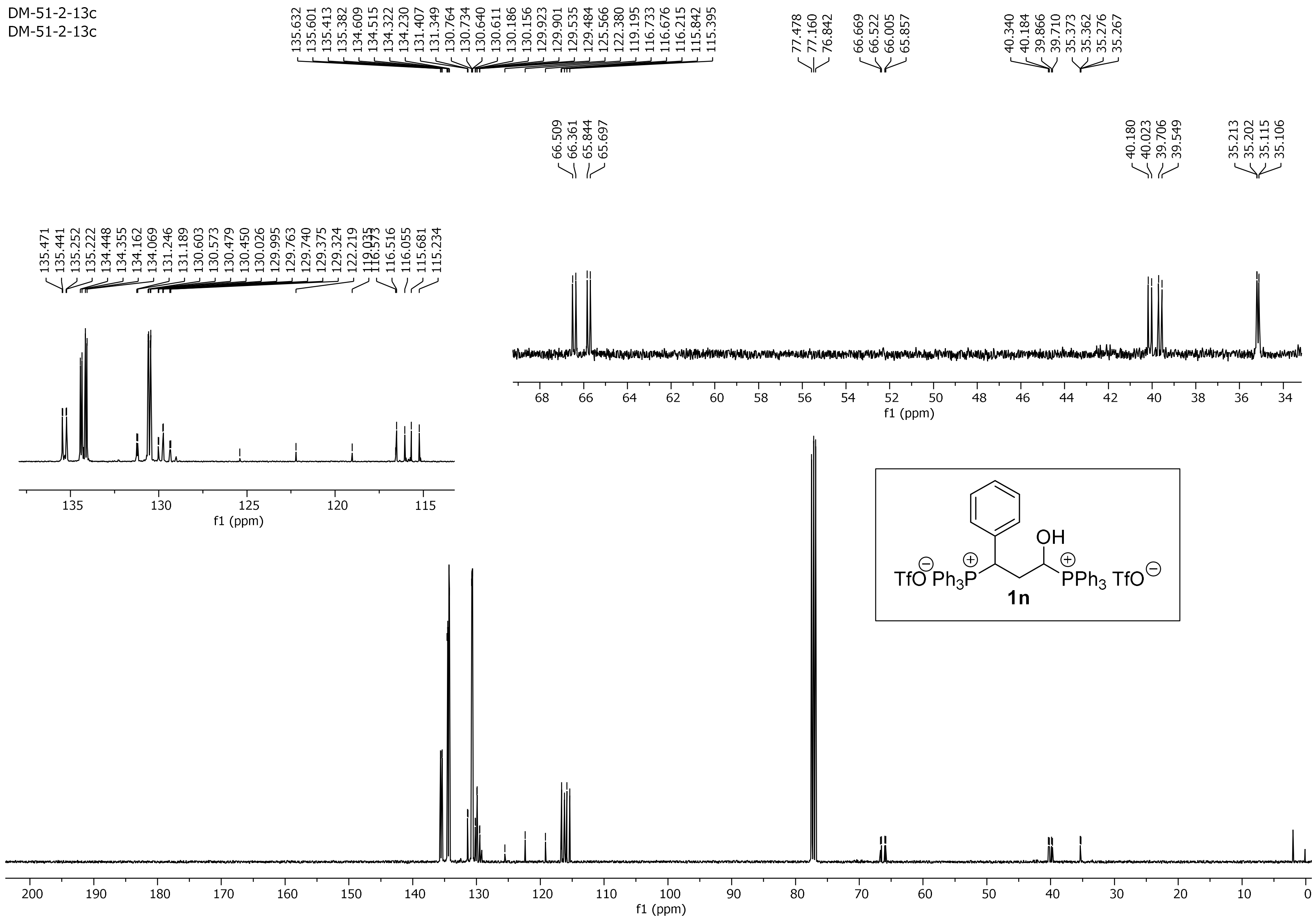

^{13}C NMR spectrum of 1-hydroxy-3-phenylpropane-1,3-bis(triphenylphosphonium) bis(triflate) (**1n**); 100 MHz/CDCl$_3$/TMS; δ (ppm).

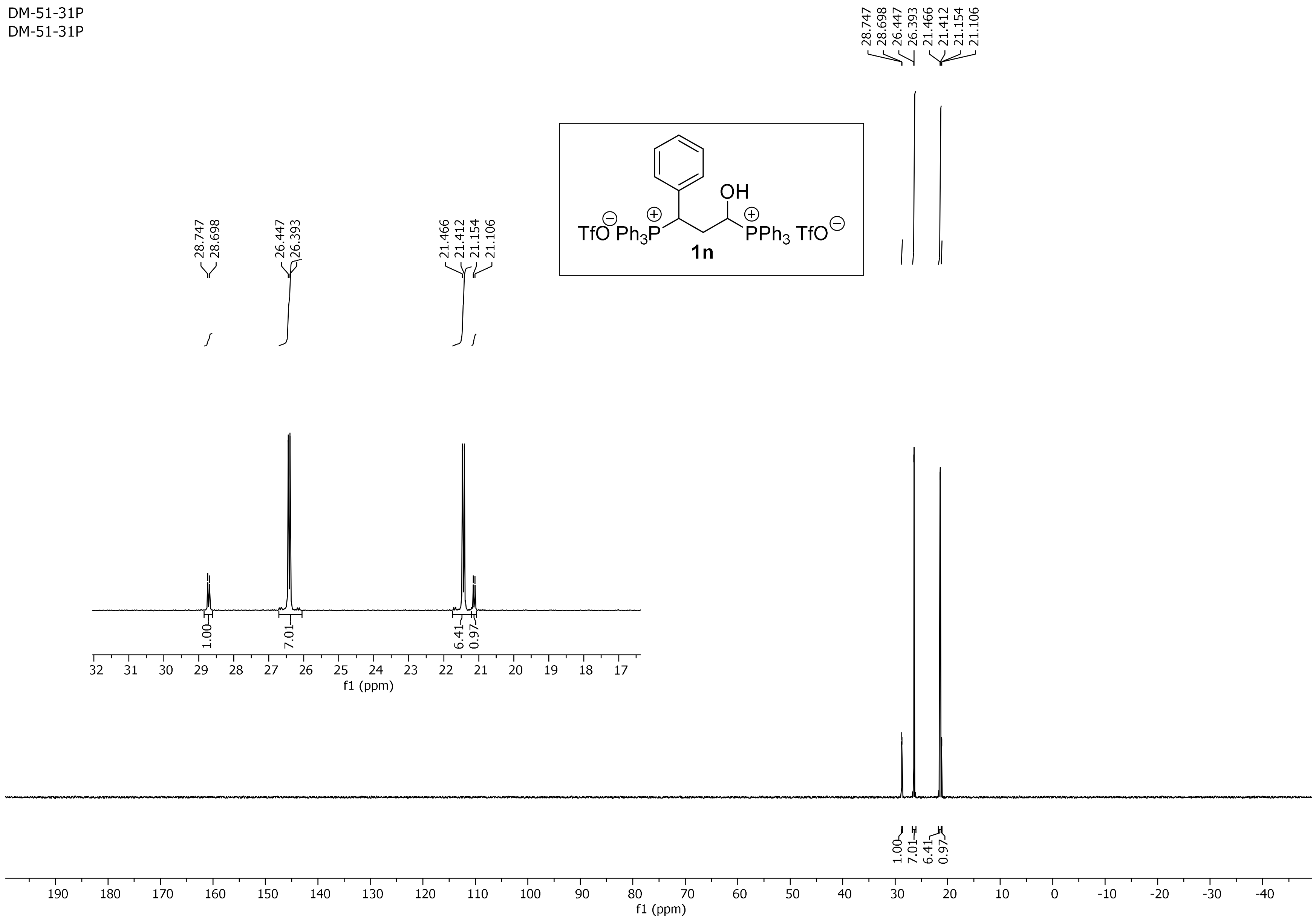

^{31}P NMR spectrum of 1-hydroxy-3-phenylpropane-1,3-bis(triphenylphosphonium) bis(triflate) (**1n**); 161.9 MHz/$CDCl_3$; δ (ppm).

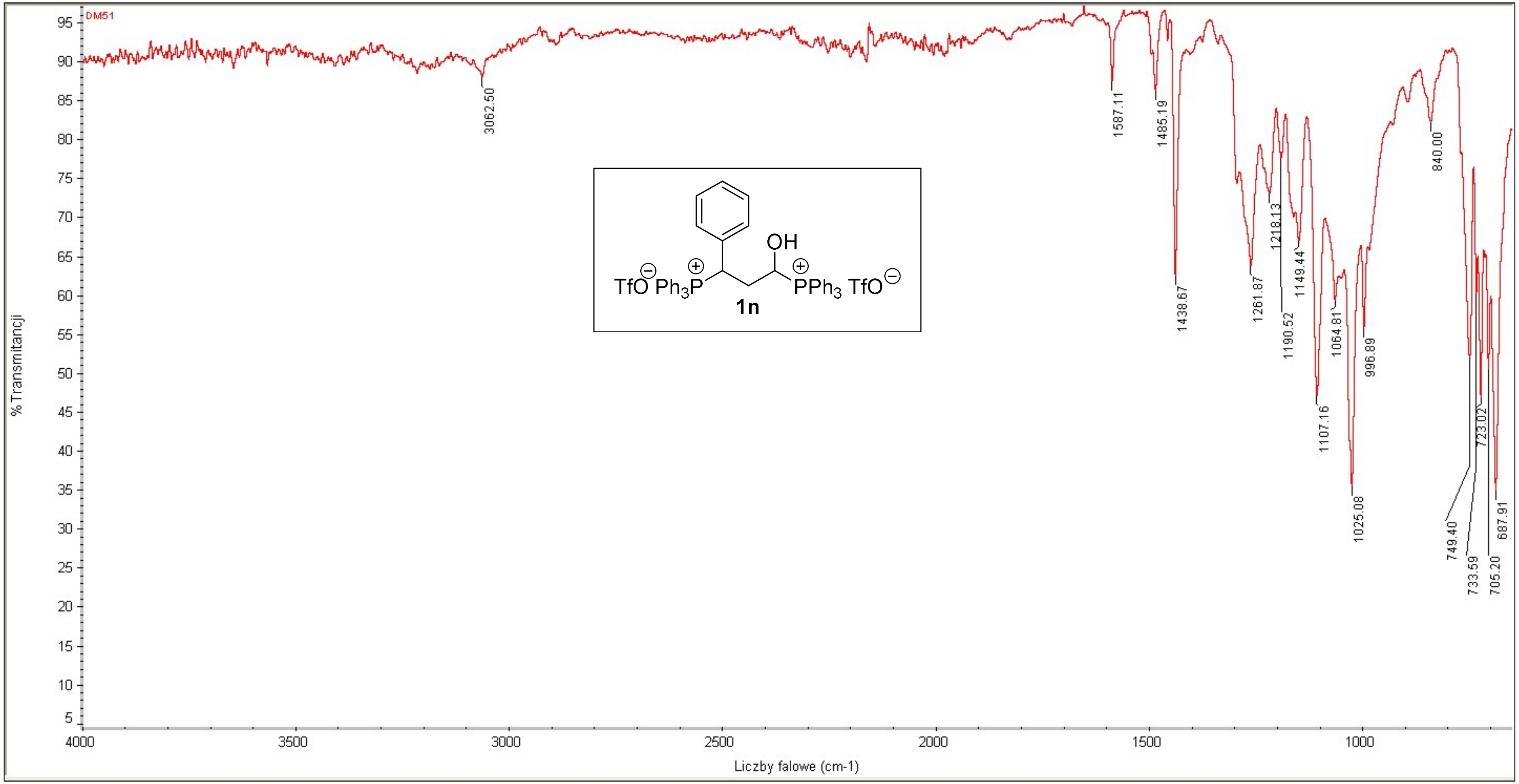

IR spectrum of 1-hydroxy-3-phenylpropane-1,3-bis(triphenylphosphonium) bis(triflate) (**1n**); ATR (cm^{-1}).

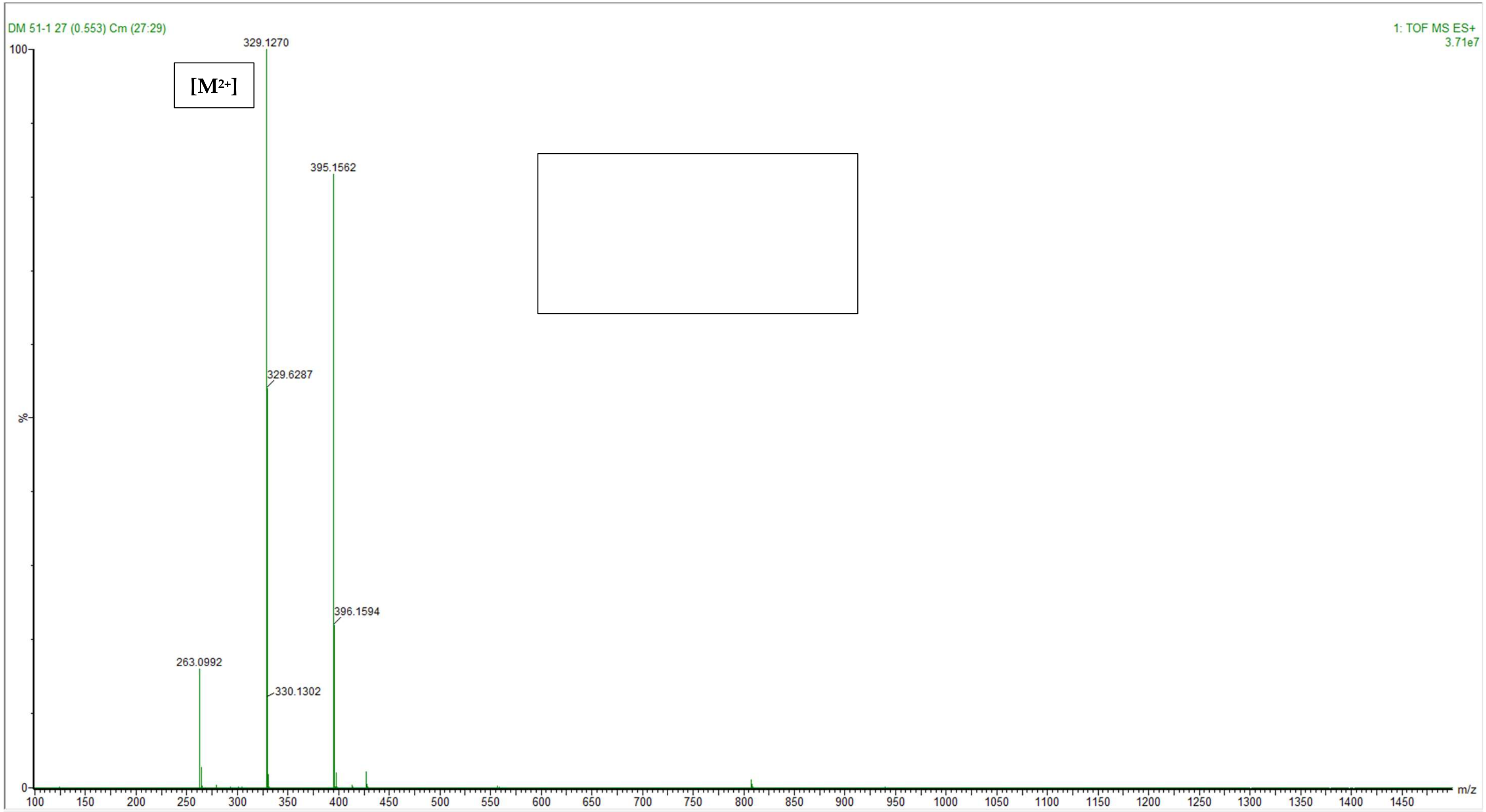

MS spectrum of 1-hydroxy-3-phenylpropane-1,3-bis(triphenylphosphonium) bis(triflate) (**1n**).

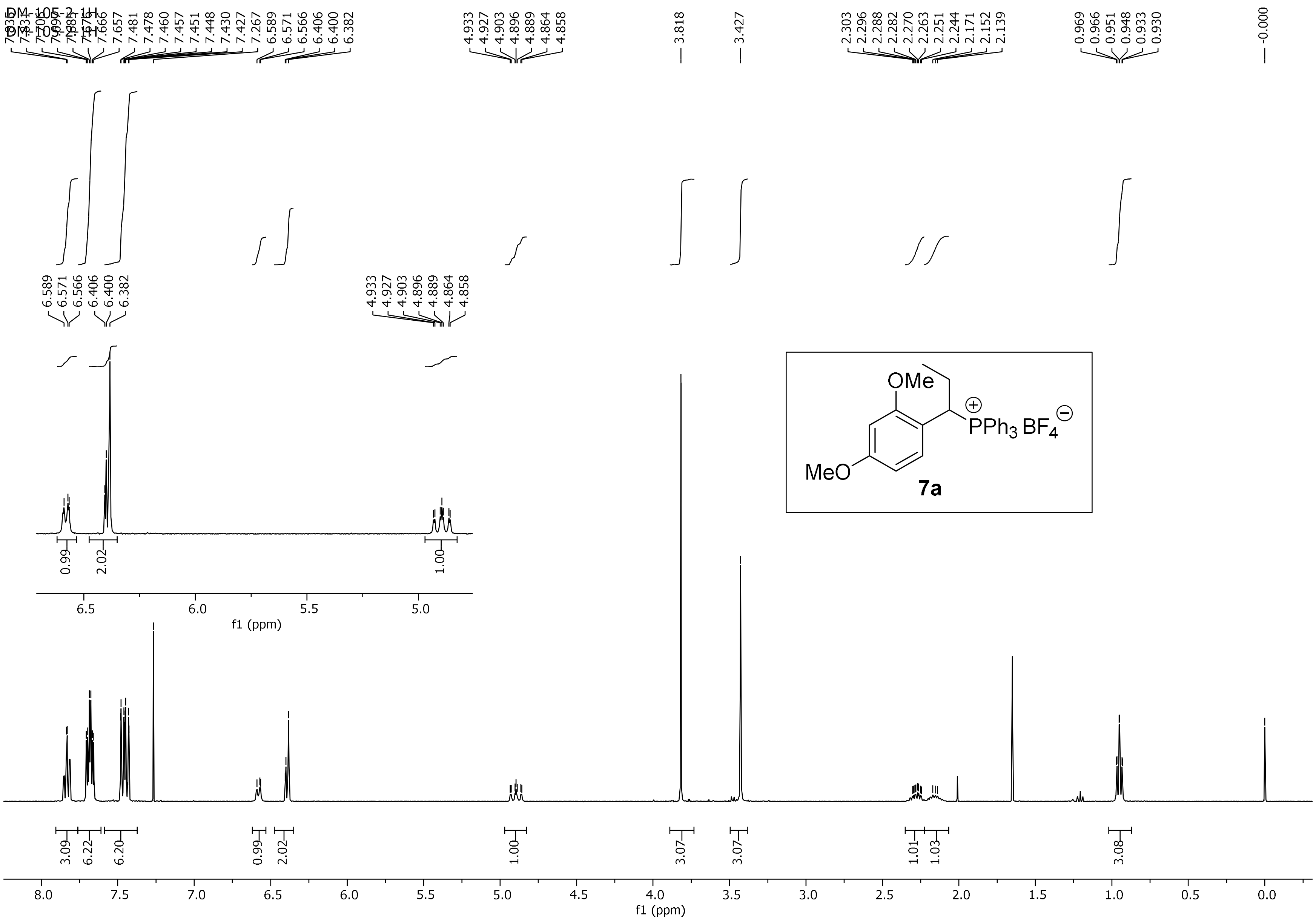

^{1}H NMR spectrum of 1-(2,4-dimethoxyphenyl)propyltriphenylphosphonium tetrafluoroborate (**7a**); 400 MHz/$CDCl_3$/TMS; δ (ppm).

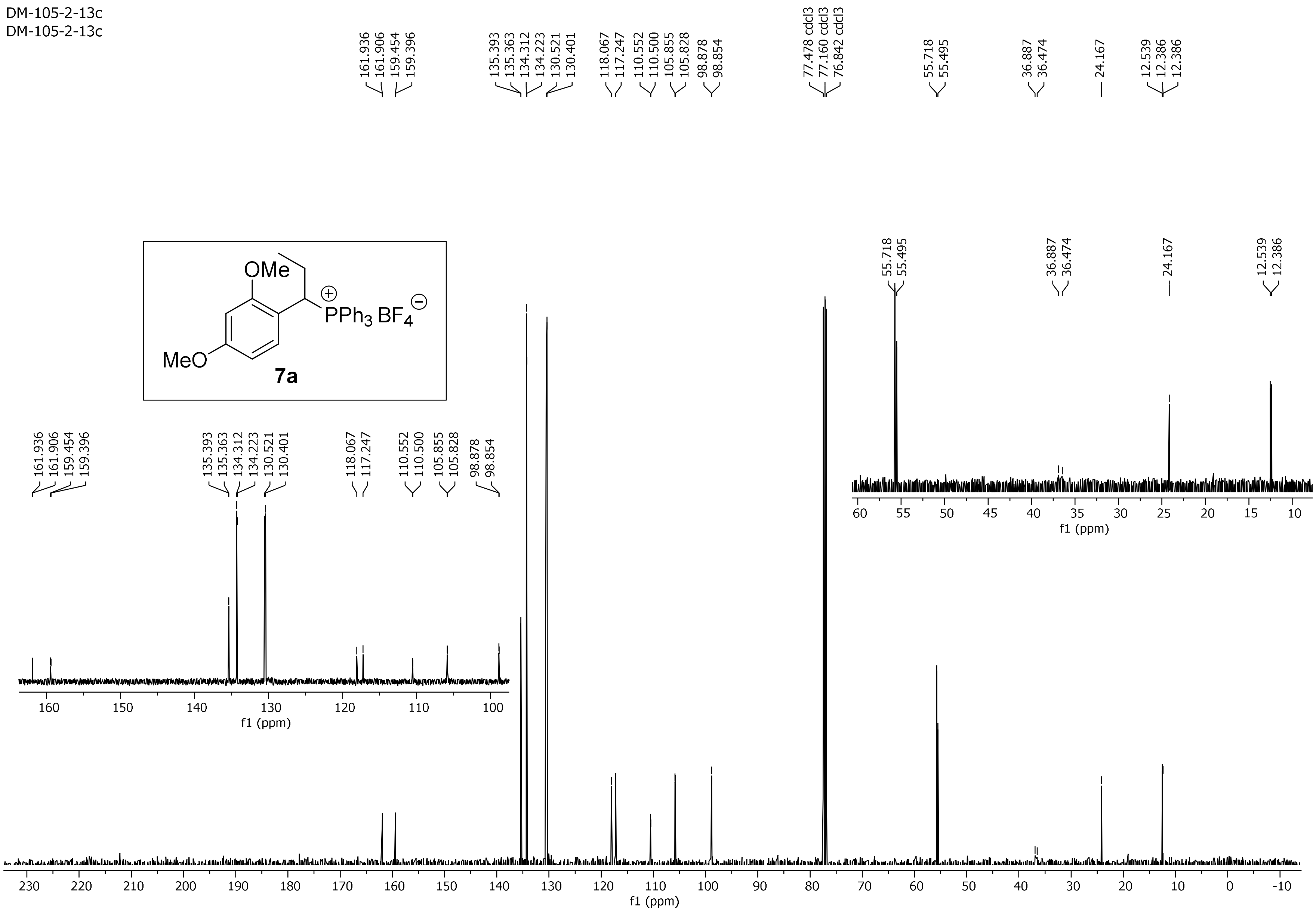

^{13}C NMR spectrum of 1-(2,4-dimethoxyphenyl)propyltriphenylphosphonium tetrafluoroborate (**7a**); 100 MHz/$CDCl_3$/TMS; δ (ppm).

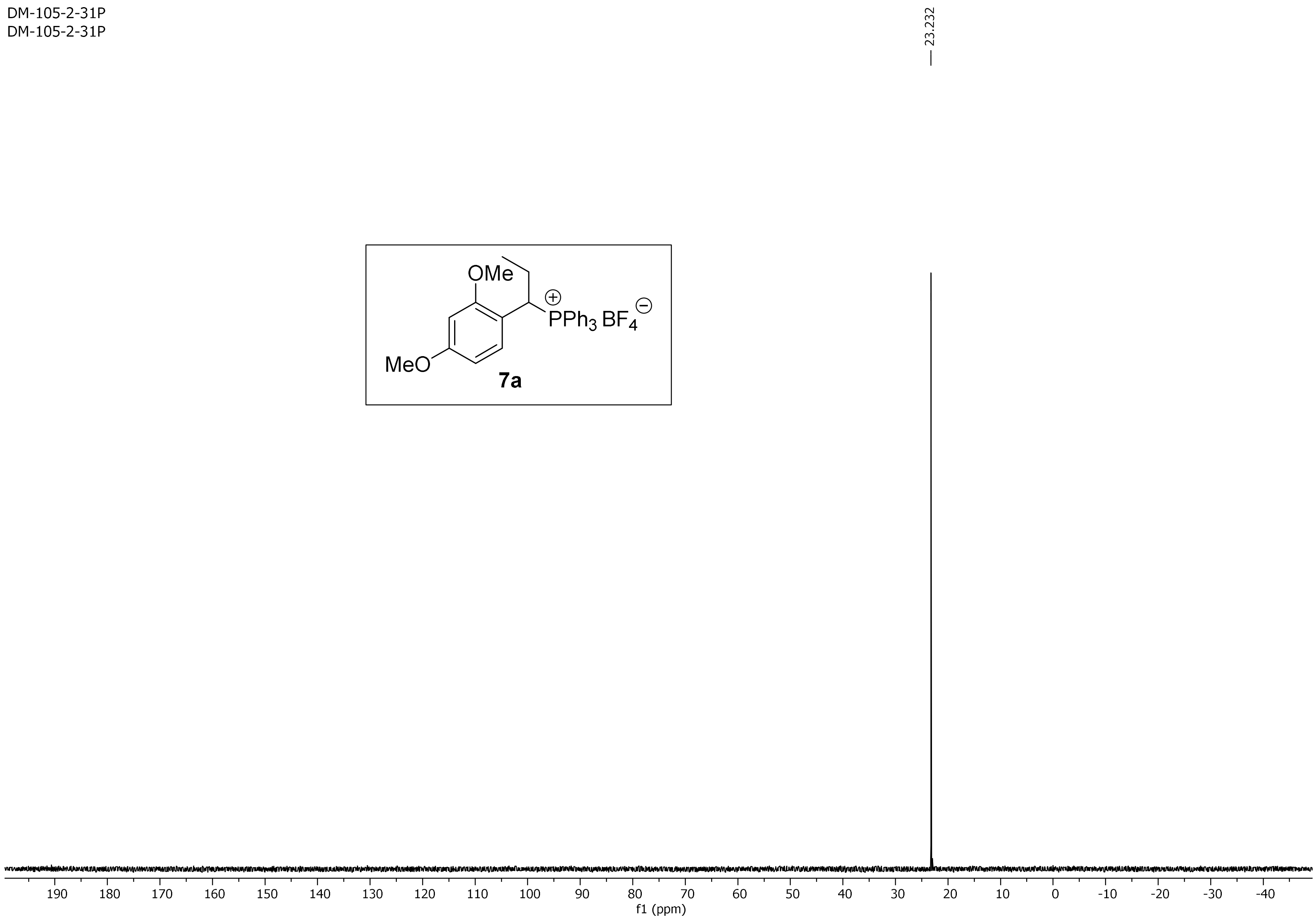

^{31}P NMR spectrum of 1-(2,4-dimethoxyphenyl)propyltriphenylphosphonium tetrafluoroborate (**7a**); 161.9 MHz/$CDCl_3$; δ (ppm).

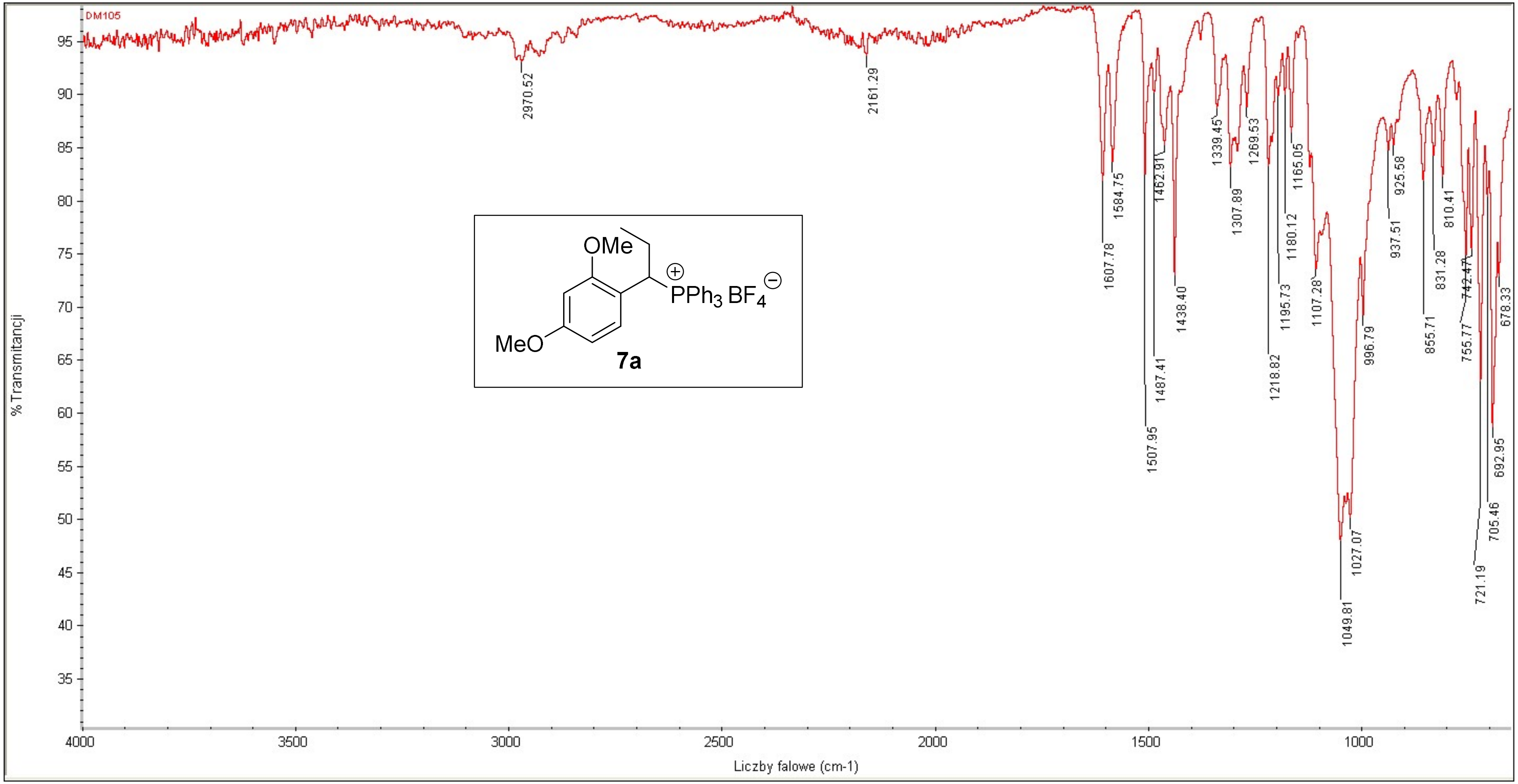

IR spectrum of 1-(2,4-dimethoxyphenyl)propyltriphenylphosphonium tetrafluoroborate (**7a**); ATR (cm^{-1}).

Tolerance = 40.0 mDa / DBE: min = -10.0, max = 100.0
Element prediction: Off
Number of isotope peaks used for i-FIT = 2
Monoisotopic Mass, Even Electron Ions
7 formula(e) evaluated with 1 results within limits (up to 3 closest results for each mass)
Elements Used:

Mass	RA	Calc. Mass	mDa	PPM	DBE	Formula	i-FIT	i-FIT Norm	Fit Conf %	C	H	O	P
441.1982	100.00	441.1983	-0.1	-0.2	15.5	C29 H30 O2 P	514.2	n/a	n/a	29	30	2	1

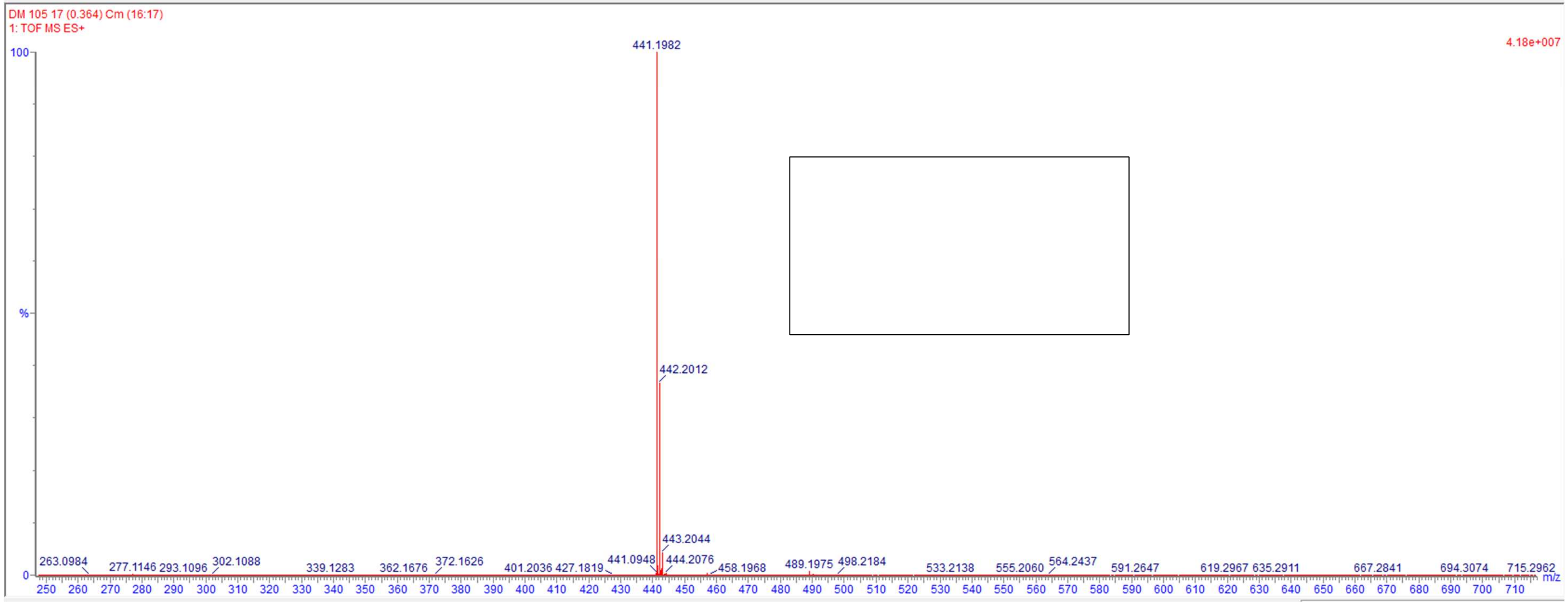

MS spectrum of 1-(2,4-dimethoxyphenyl)propyltriphenylphosphonium tetrafluoroborate (**7a**).

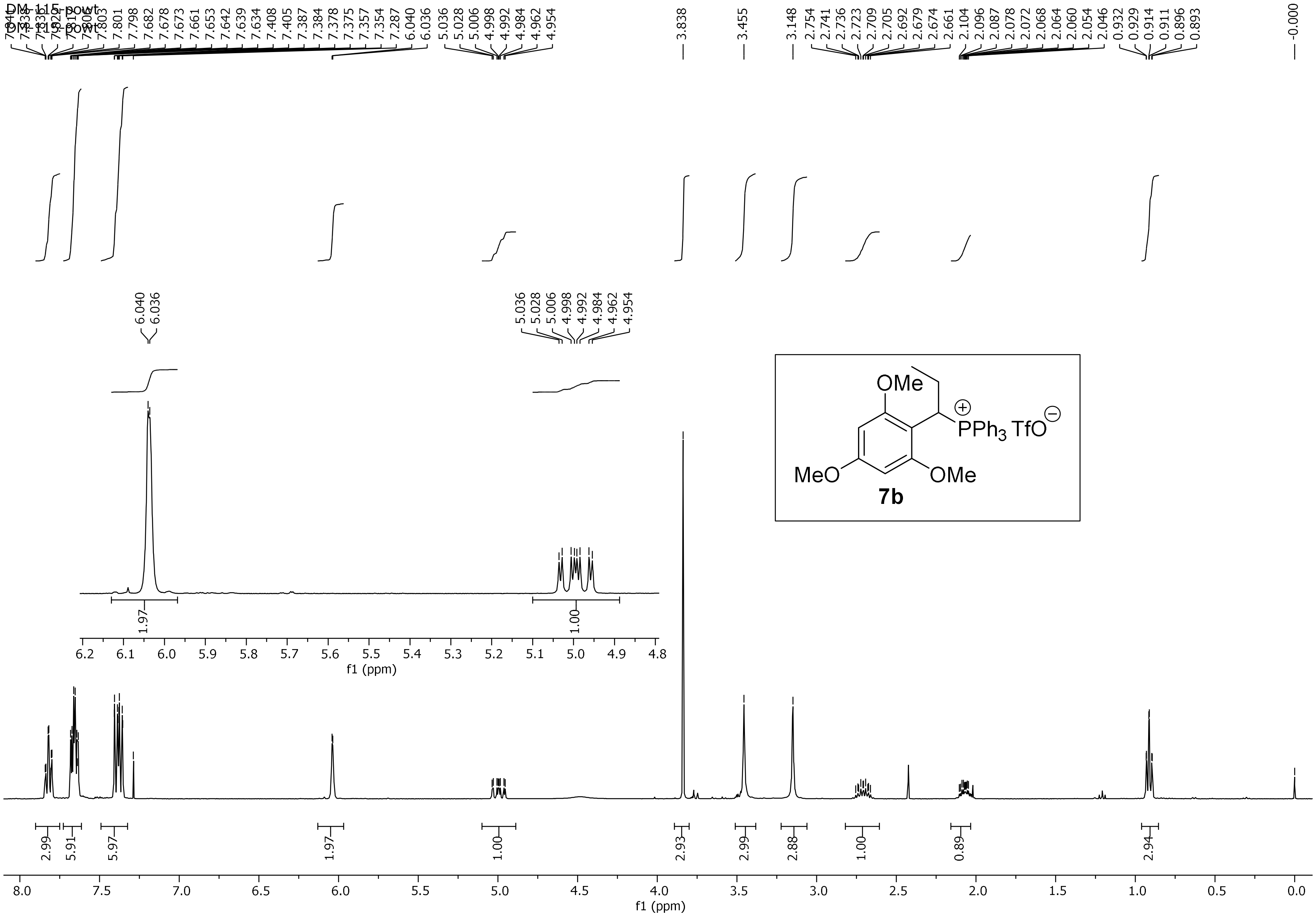

7b

^{1}H NMR spectrum of 1-(2,4,6-trimethoxyphenyl)propyltriphenylphosphonium triflate (**7b**); 400 MHz/$CDCl_3$/TMS; δ (ppm).

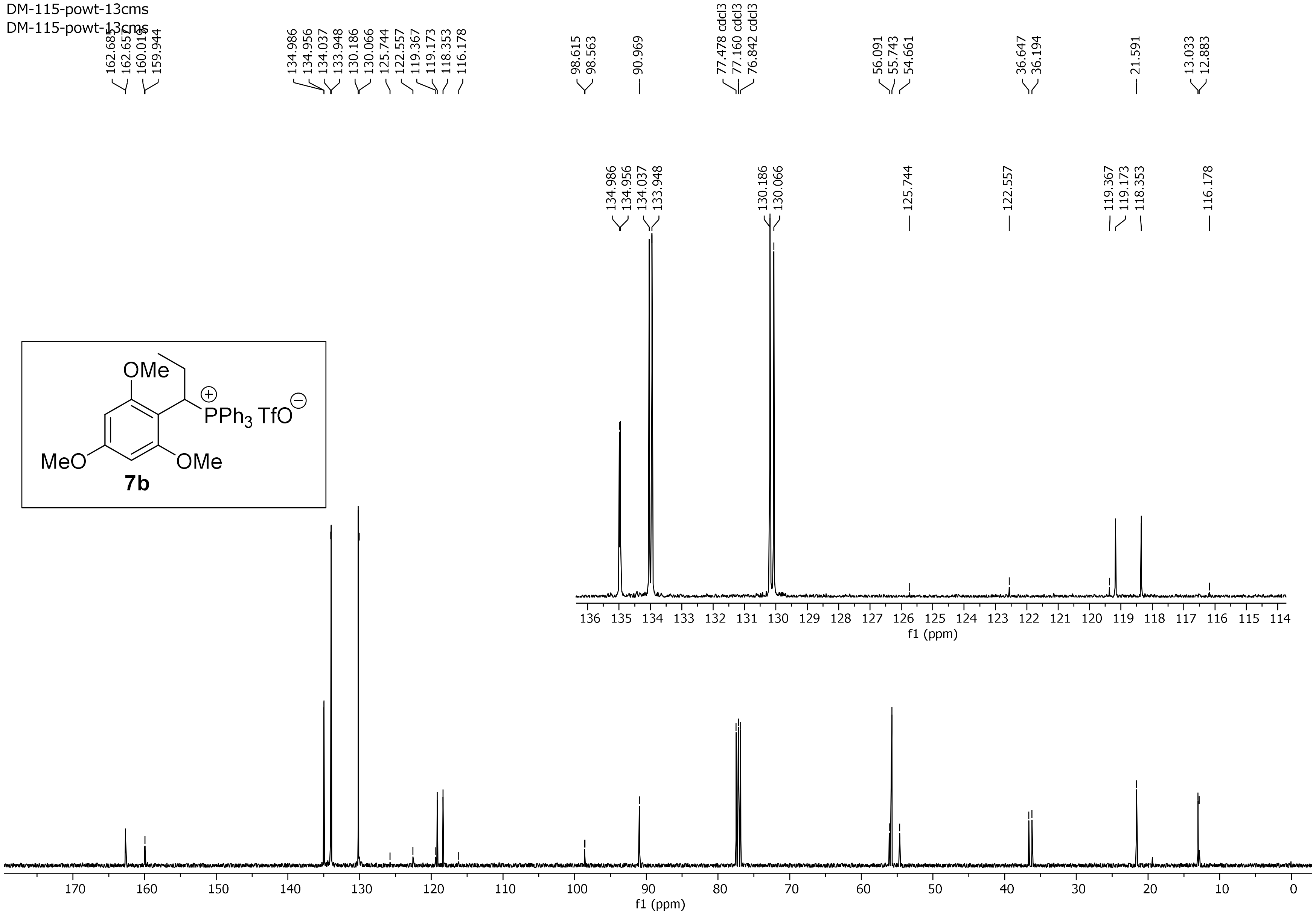

^{13}C NMR spectrum of 1-(2,4,6-trimethoxyphenyl)propyltriphenylphosphonium triflate (**7b**); 100 MHz/$CDCl_3$/TMS; δ (ppm).

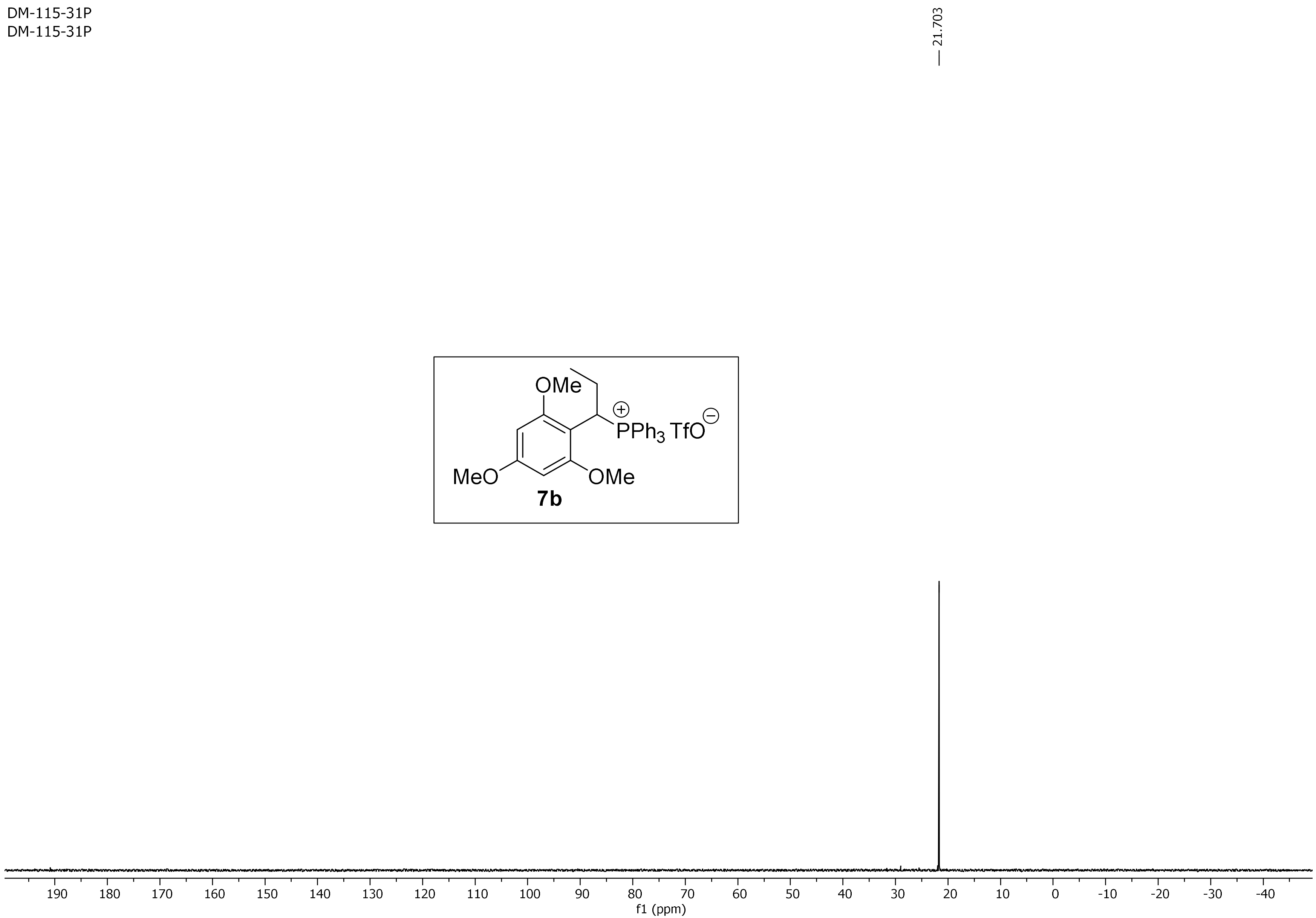

^{31}P NMR spectrum of 1-(2,4,6-trimethoxyphenyl)propyltriphenylphosphonium triflate (**7b**); 161.9 MHz/$CDCl_3$; δ (ppm).

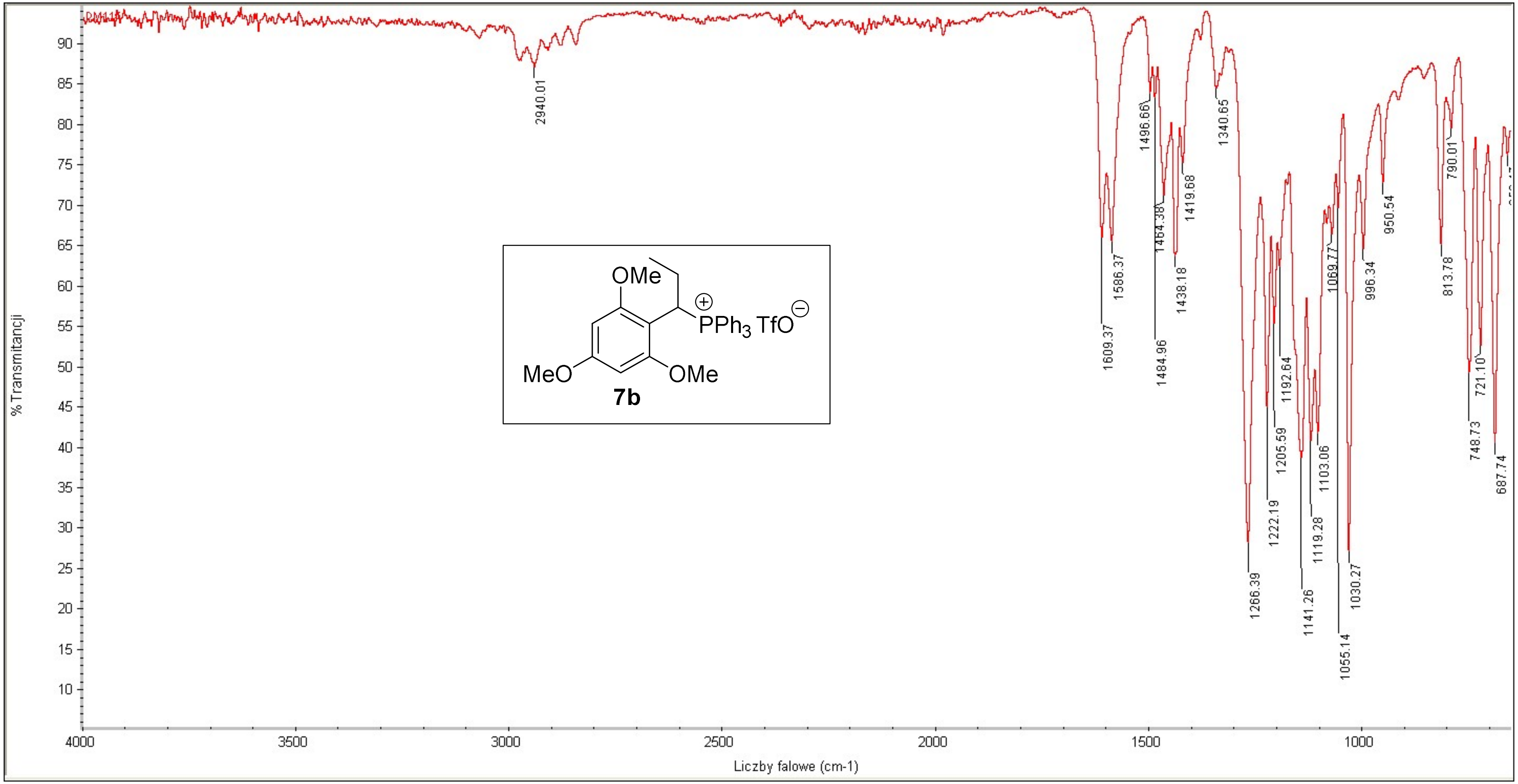

IR spectrum of 1-(2,4,6-trimethoxyphenyl)propyltriphenylphosphonium triflate (**7b**); ATR (cm^{-1}).

Tolerance = 40.0 mDa / DBE: min = -10.0, max = 100.0
Element prediction: Off
Number of isotope peaks used for i-FIT = 2
Monoisotopic Mass, Even Electron Ions
7 formula(e) evaluated with 1 results within limits (up to 3 closest results for each mass)
Elements Used:

Mass	RA	Calc. Mass	mDa	PPM	DBE	Formula	i-FIT	i-FIT Norm	Fit Conf %	C	H	O	P
471.2093	100.00	471.2089	0.4	0.8	15.5	C30 H32 O3 P	555.9	n/a	n/a	30	32	3	1

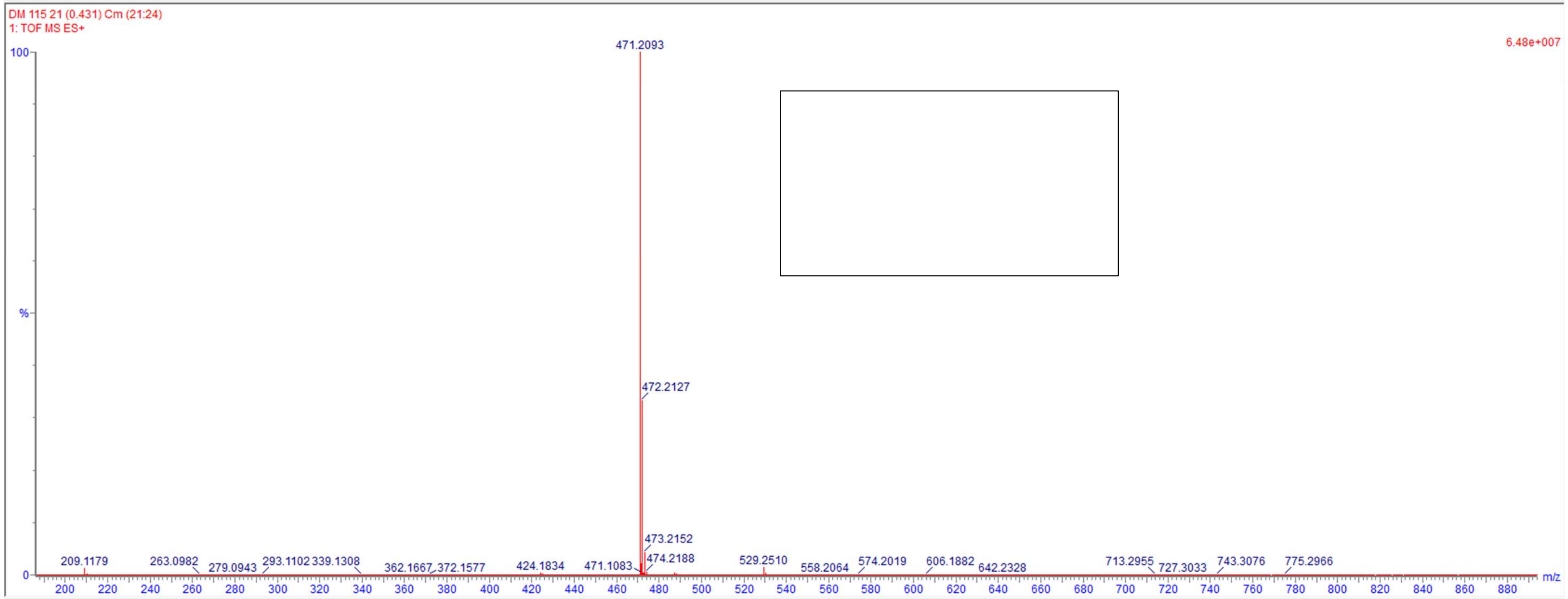

MS spectrum of 1-(2,4,6-trimethoxyphenyl)propyltriphenylphosphonium triflate (**7b**).

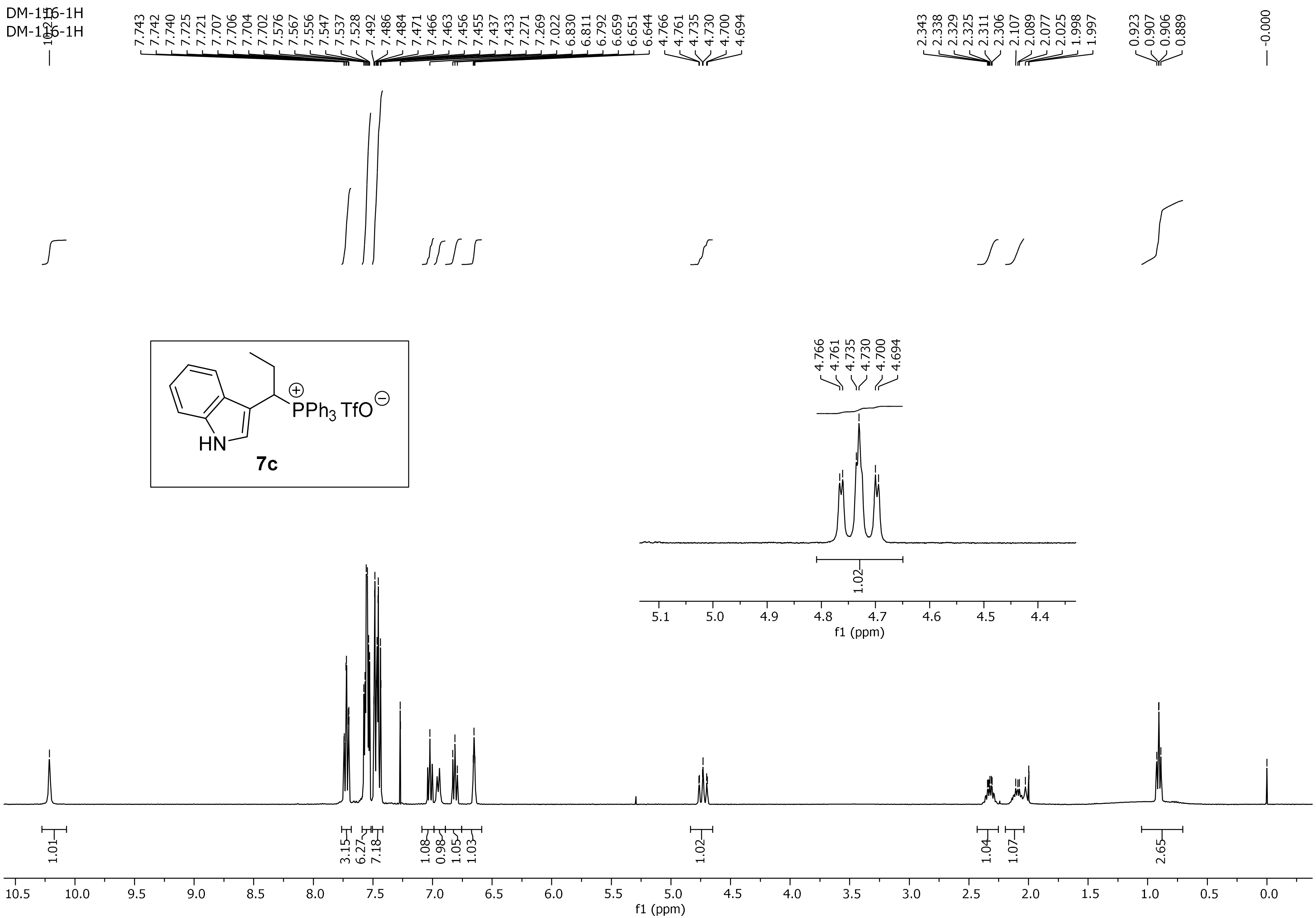

^{1}H NMR spectrum of 1-(*H*-indol-3-yl)propyltriphenylphosphonium triflate (**7c**); 400 MHz/$CDCl_3$/TMS; δ (ppm).

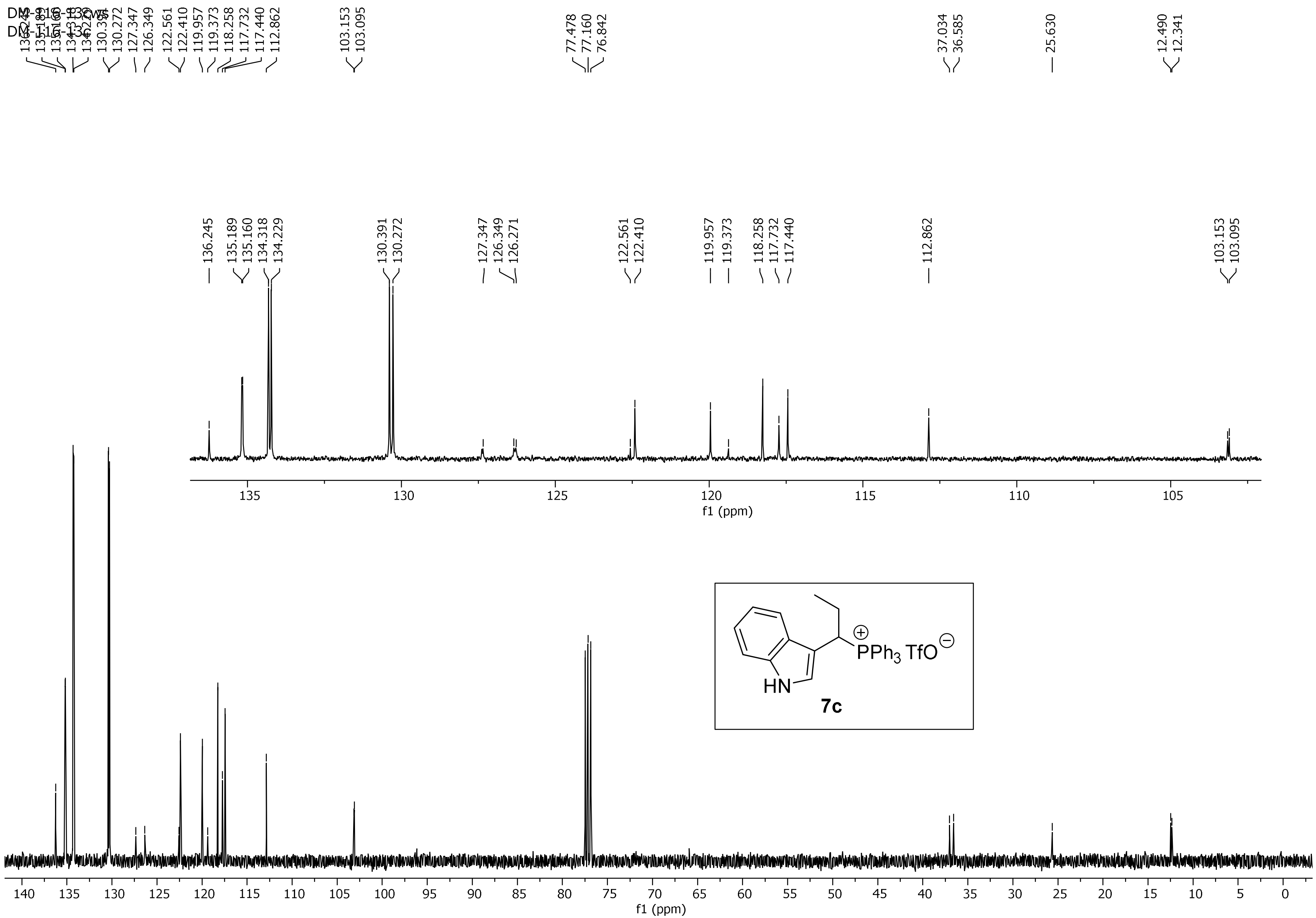

^{13}C NMR spectrum of 1-(*H*-indol-3-yl)propyltriphenylphosphonium triflate (**7c**); 100 MHz/$CDCl_3$/TMS; *δ* (ppm).

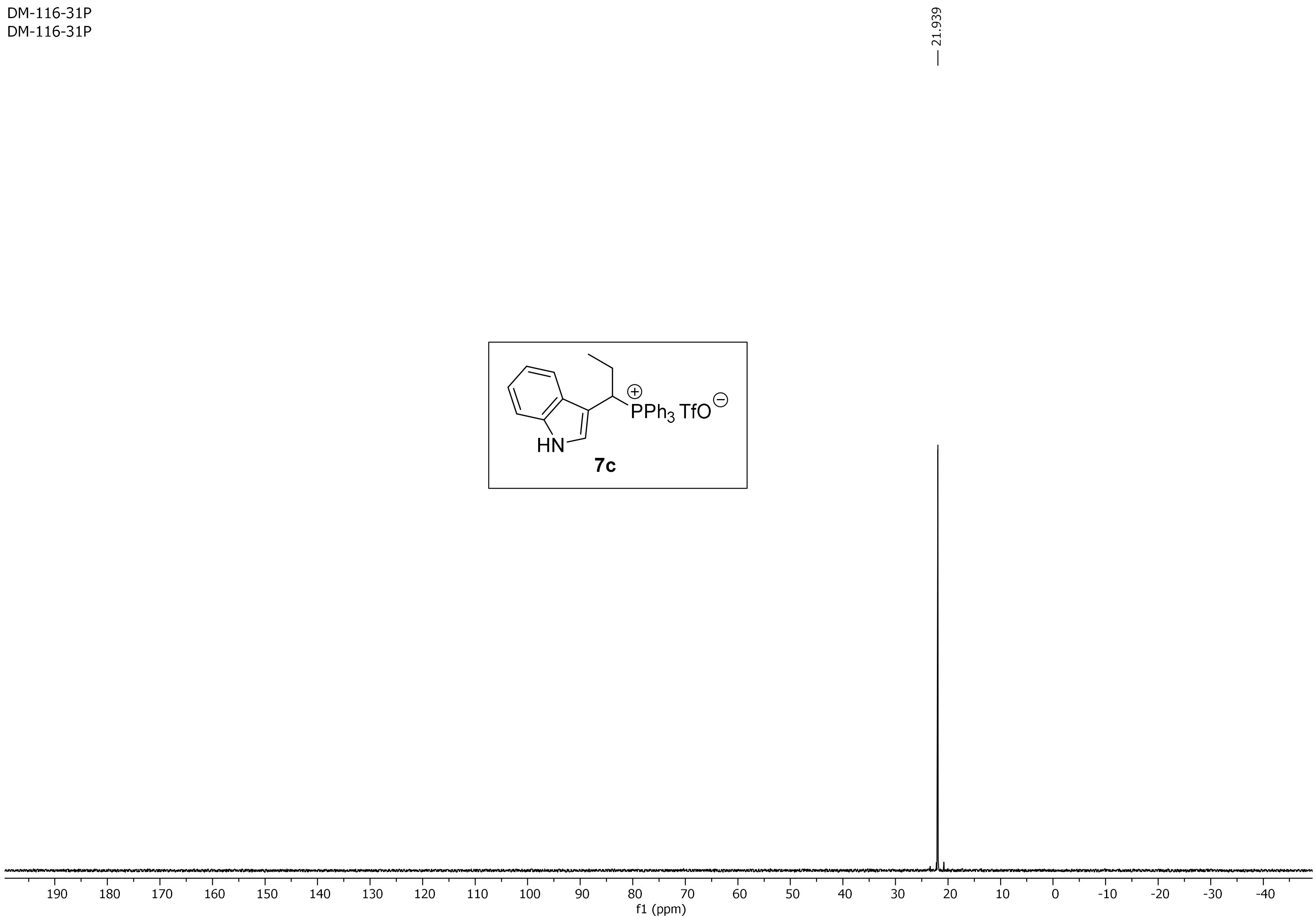

^{31}P NMR spectrum of 1-(*H*-indol-3-yl)propyltriphenylphosphonium triflate (**7c**); 161.9 MHz/$CDCl_3$; δ (ppm).

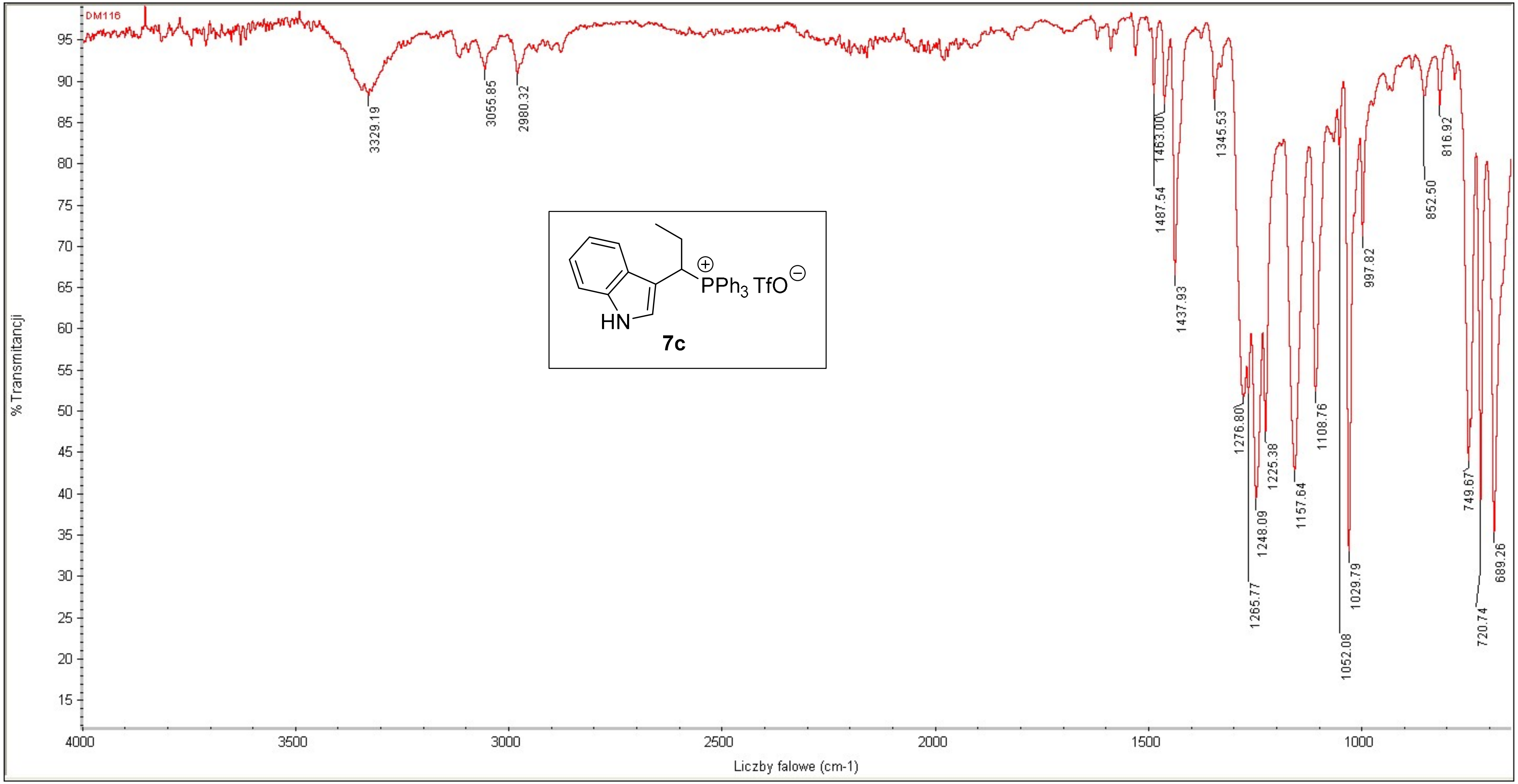

IR spectrum of 1-(*H*-indol-3-yl)propyltriphenylphosphonium triflate (**7c**); ATR (cm^{-1}).

Tolerance = 40.0 mDa / DBE: min = -10.0, max = 100.0
Element prediction: Off
Number of isotope peaks used for i-FIT = 2
Monoisotopic Mass, Even Electron Ions
7 formula(e) evaluated with 1 results within limits (up to 3 closest results for each mass)
Elements Used:

Mass	RA	Calc. Mass	mDa	PPM	DBE	Formula	i-FIT	i-FIT Norm	Fit Conf %	C	H	N	P
420.1882	100.00	420.1881	0.1	0.2	17.5	C29 H27 N P	520.7	n/a	n/a	29	27	1	1

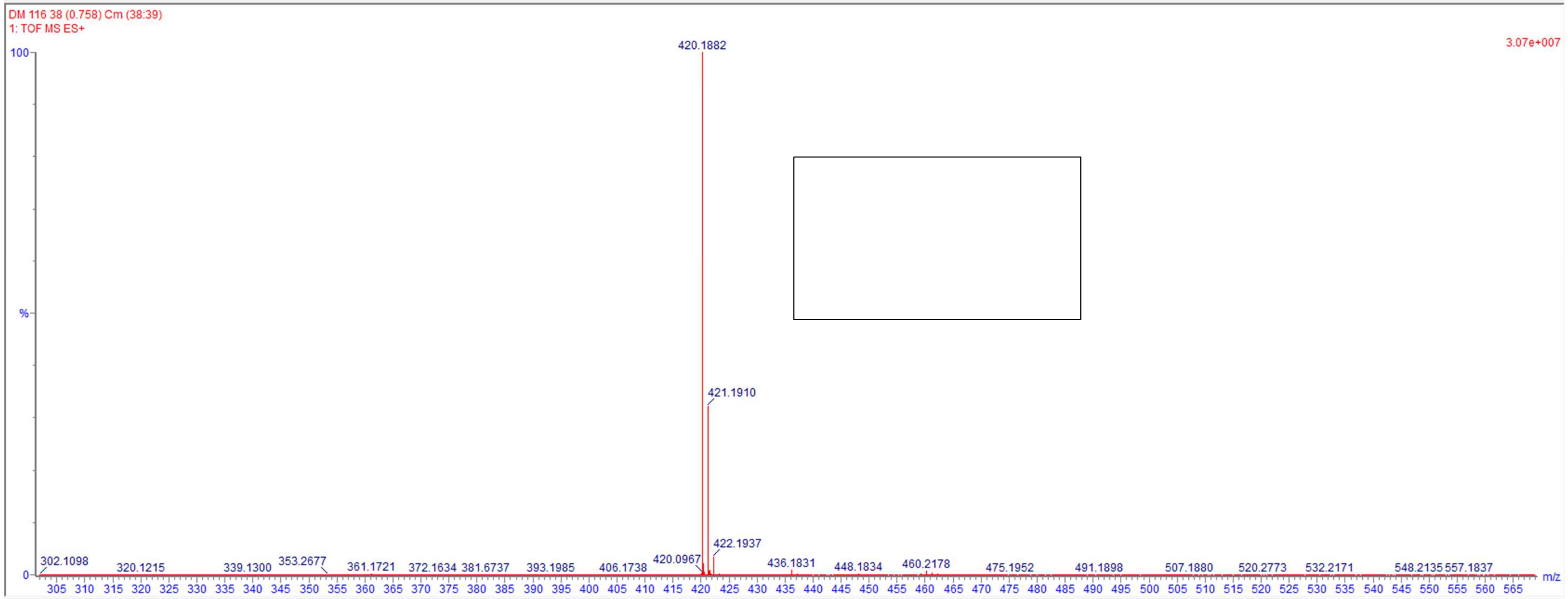

MS spectrum of 1-(*H*-indol-3-yl)propyltriphenylphosphonium triflate (**7c**).

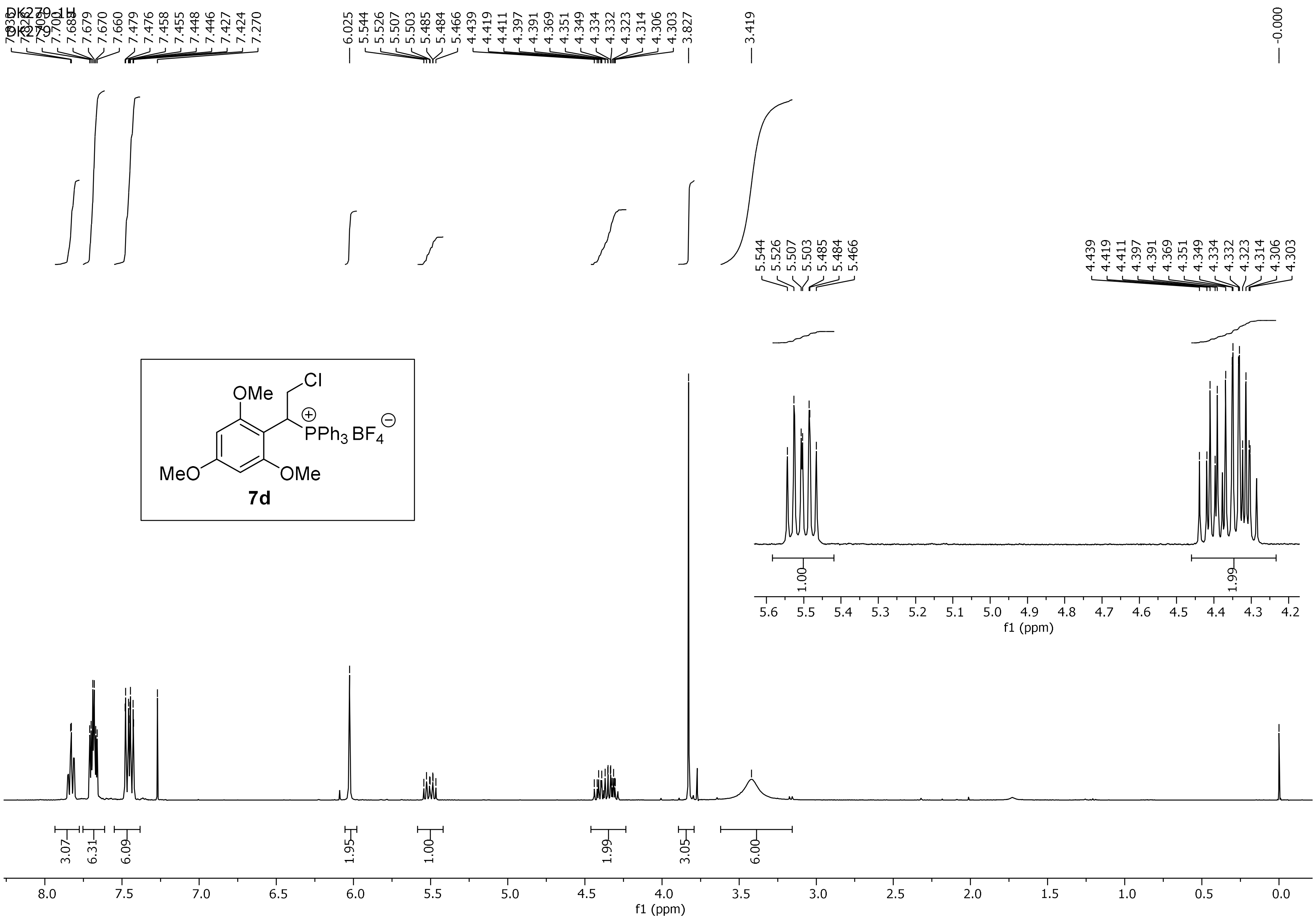

^{1}H NMR spectrum of 2-chloro-1-(2,4,6-trimethoxyphenyl)ethyltriphenylphosphonium tetrafluoroborate (**7d**); 400 MHz/$CDCl_3$/TMS; δ (ppm).

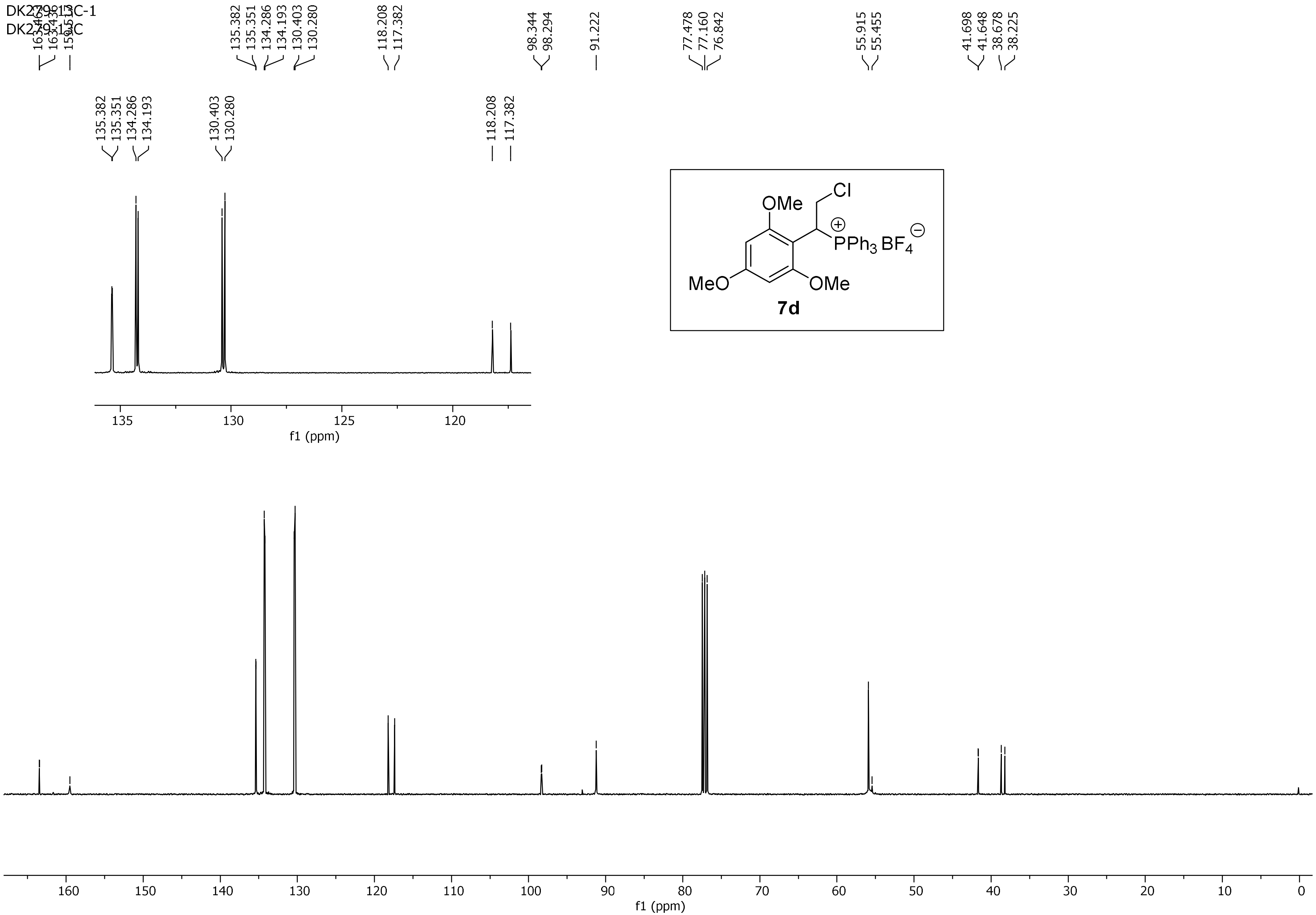

^{13}C NMR spectrum of 2-chloro-1-(2,4,6-trimethoxyphenyl)ethyltriphenylphosphonium tetrafluoroborate (**7d**); 100 MHz/$CDCl_3$/TMS; δ (ppm).

DK279-31P-nt128
STANDARD PHOSPHORUS PARAMETERS

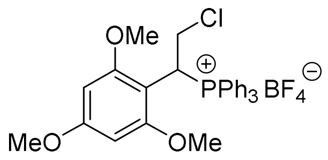

7d

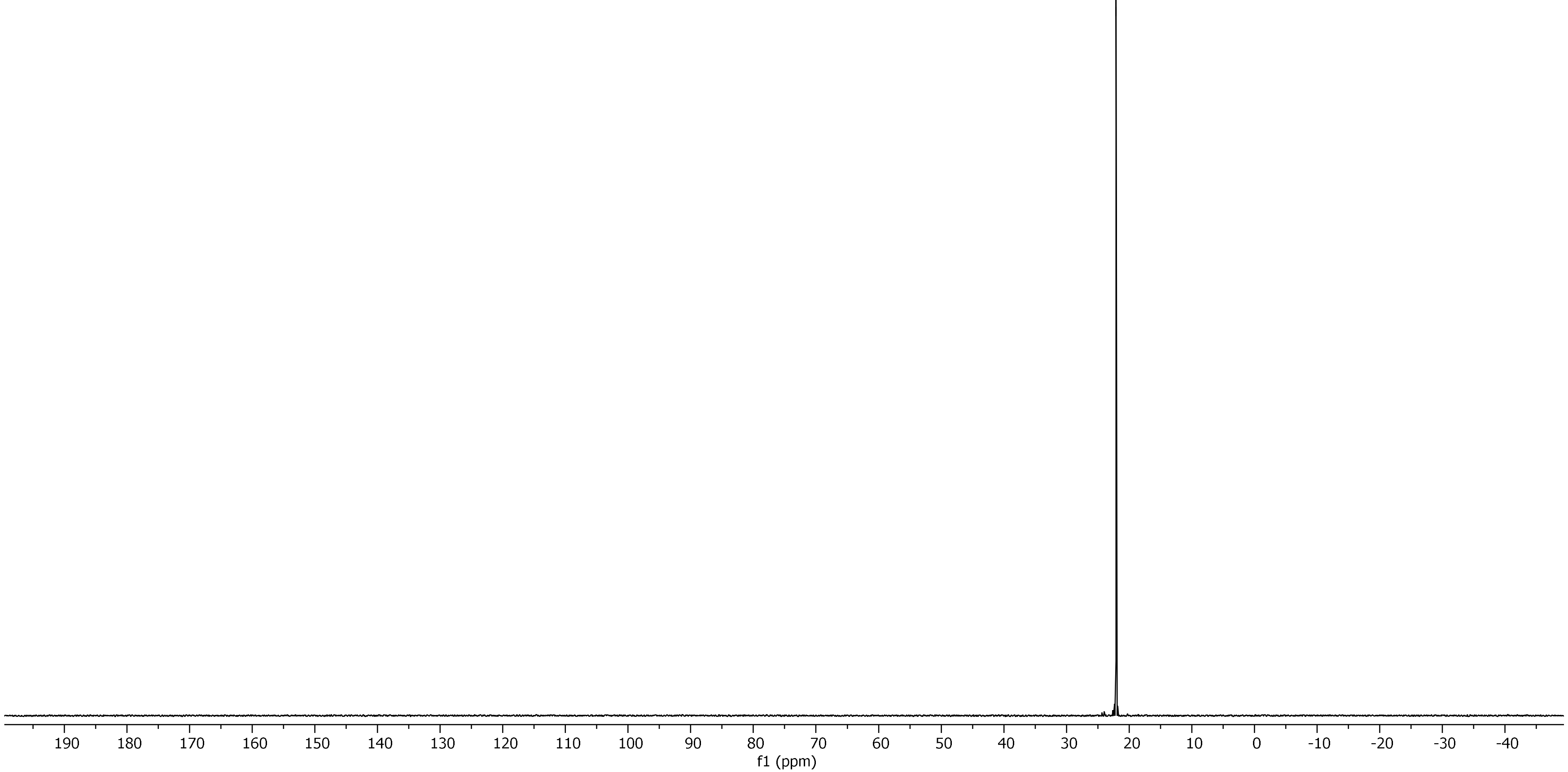

^{31}P NMR spectrum of 2-chloro-1-(2,4,6-trimethoxyphenyl)ethyltriphenylphosphonium tetrafluoroborate (**7d**); 161.9 MHz/$CDCl_3$; δ (ppm).

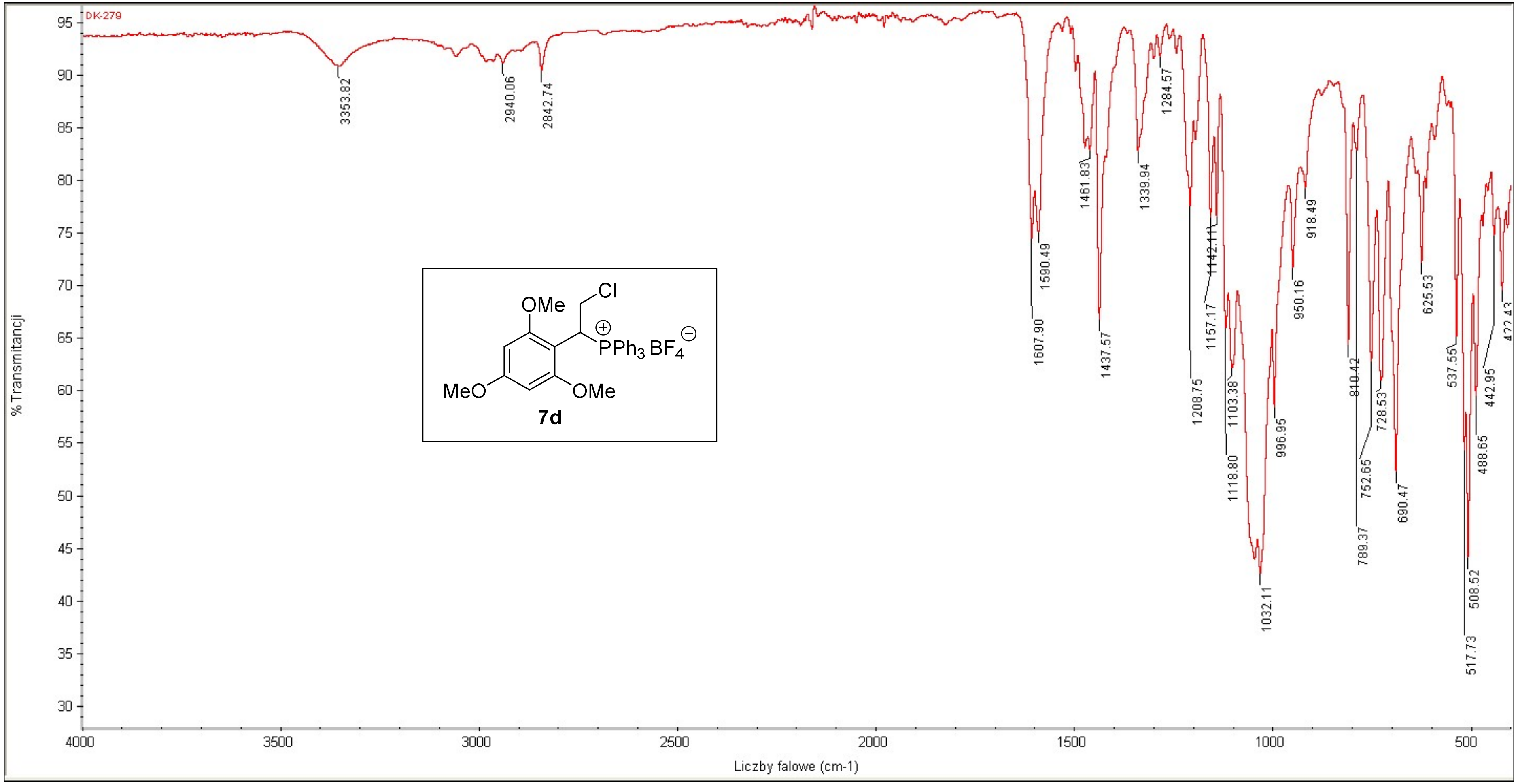

IR spectrum of 2-chloro-1-(2,4,6-trimethoxyphenyl)ethyltriphenylphosphonium tetrafluoroborate (**7d**); ATR (cm^{-1}).

Tolerance = 40.0 mDa / DBE: min = -10.0, max = 100.0
Element prediction: Off
Number of isotope peaks used for i-FIT = 2
Monoisotopic Mass, Even Electron Ions
7 formula(e) evaluated with 1 results within limits (up to 3 closest results for each mass)
Elements Used:

Mass	RA	Calc. Mass	mDa	PPM	DBE	Formula	i-FIT	i-FIT Norm	Fit Conf %	C	H	O	P	Cl
491.1546	100.00	491.1543	0.3	0.6	15.5	C29 H29 O3 P Cl	502.3	n/a	n/a	29	29	3	1	1

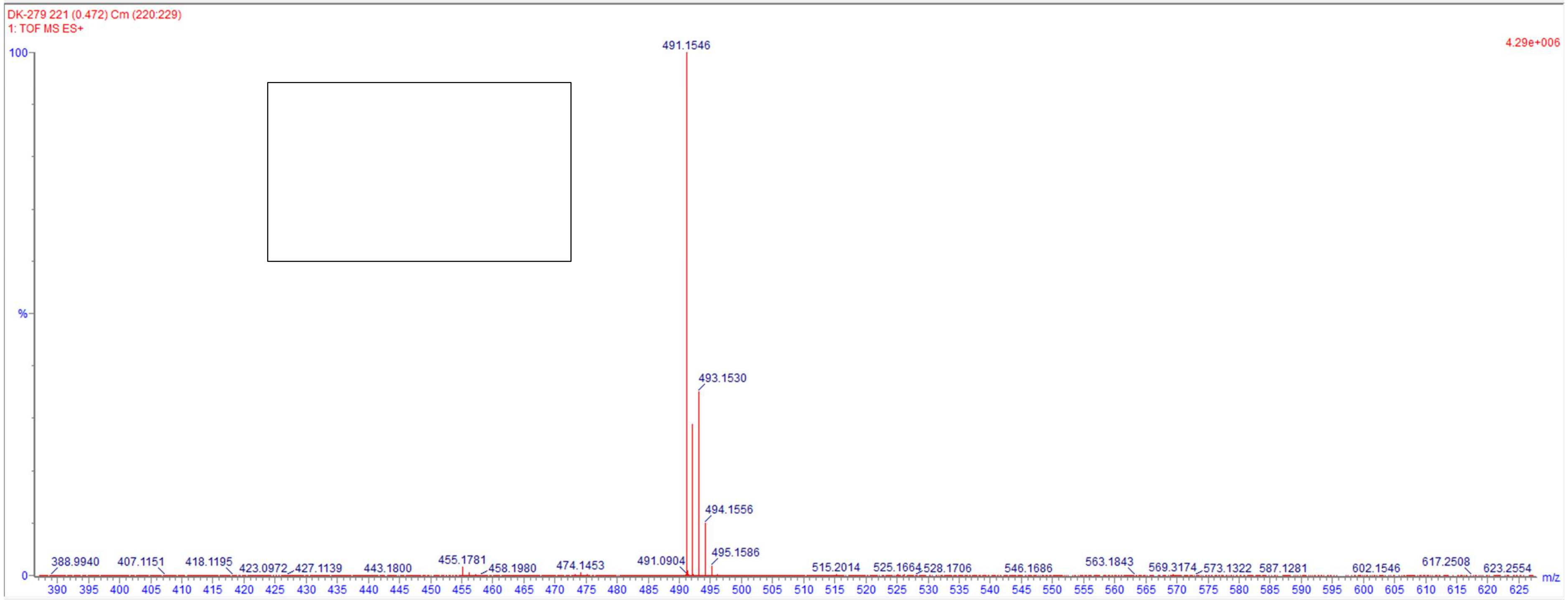

MS spectrum of 2-chloro-1-(2,4,6-trimethoxyphenyl)ethyltriphenylphosphonium tetrafluoroborate (**7d**).

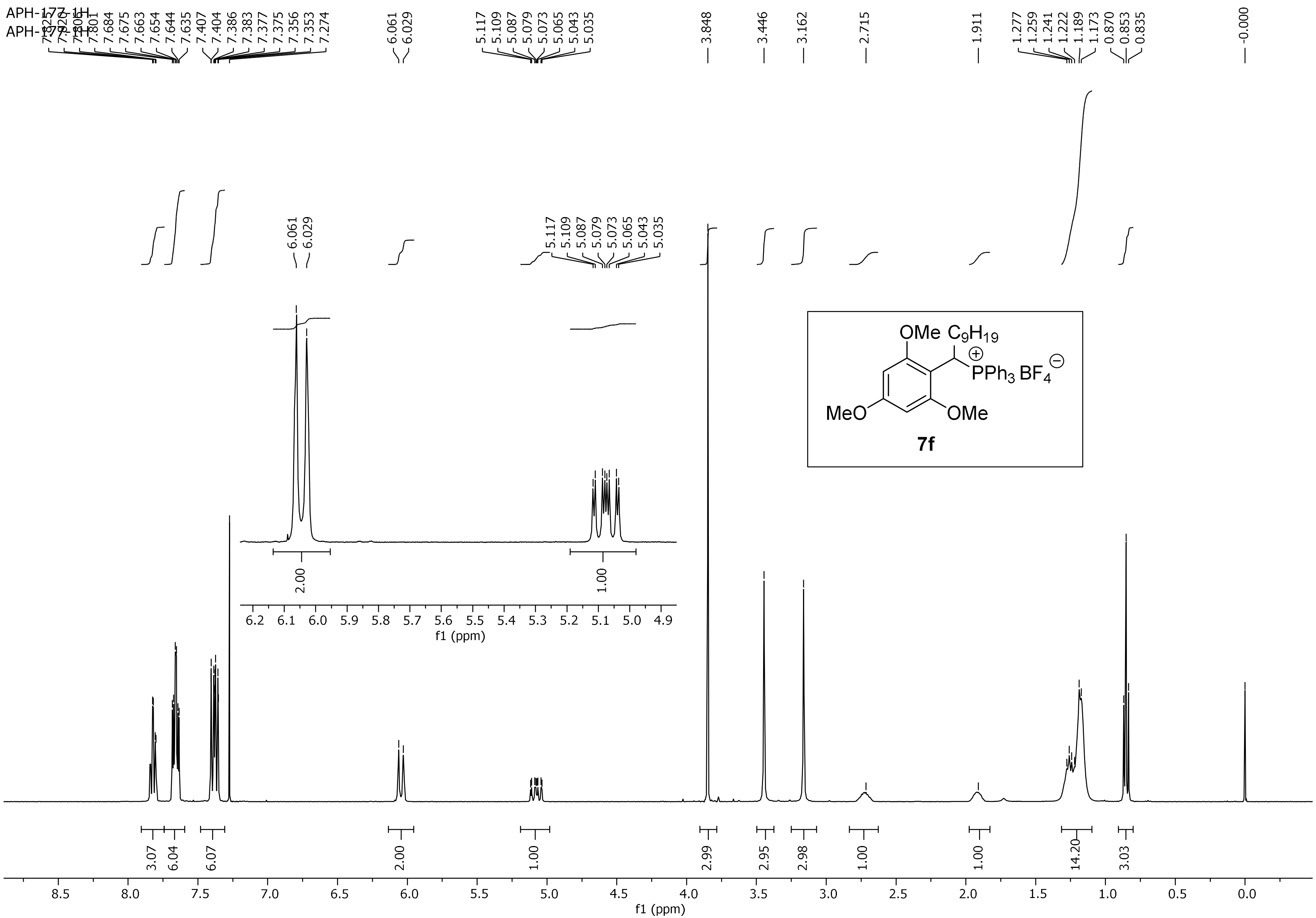

^1H NMR spectrum of 1-(2,4,6-trimethoxyphenyl)decyltriphenylphosphonium tetrafluoroborate (**7f**); 400 MHz/$CDCl_3$/TMS; δ (ppm).

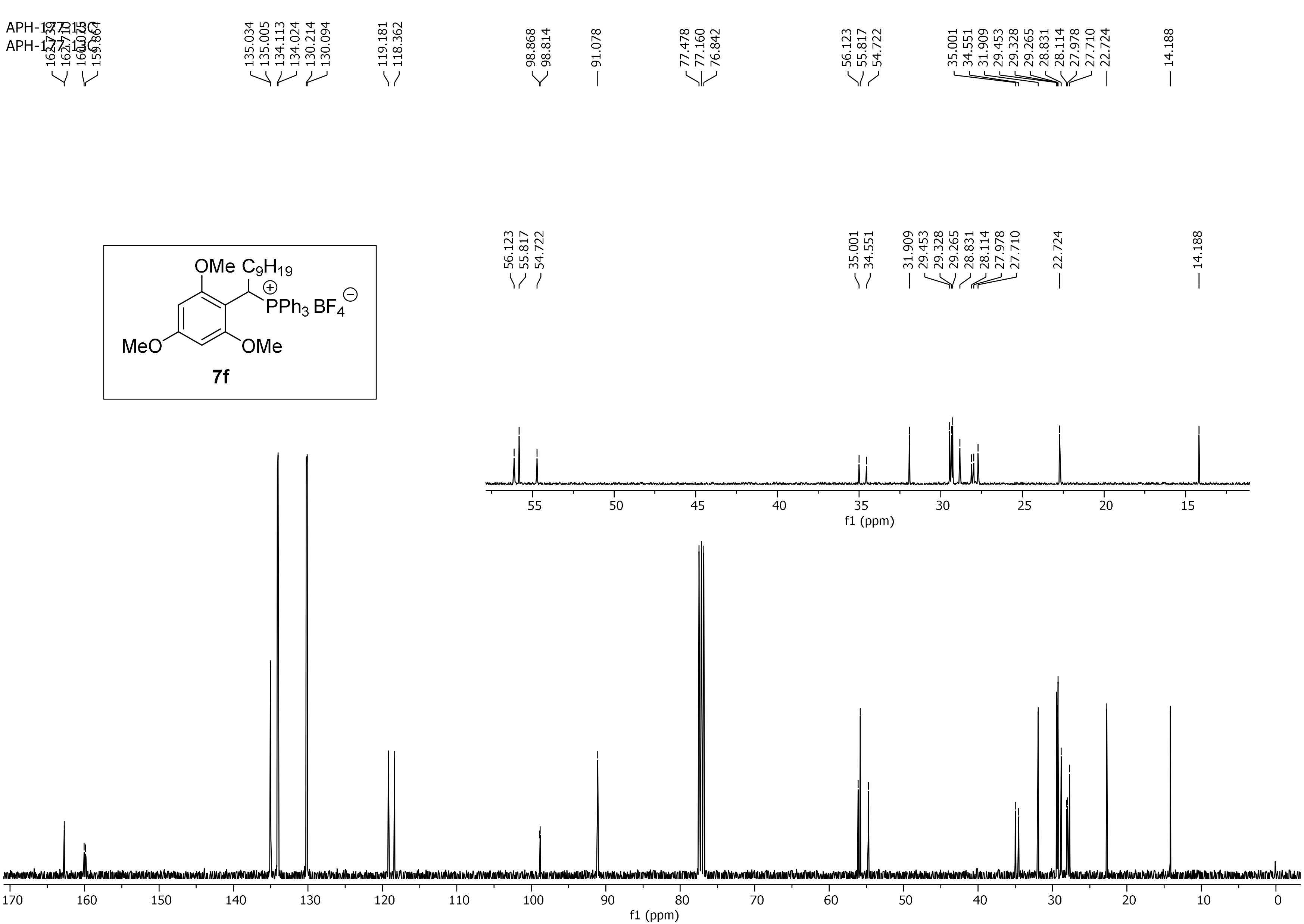

^{13}C NMR spectrum of 1-(2,4,6-trimethoxyphenyl)decyltriphenylphosphonium tetrafluoroborate (**7f**); 100 MHz/CDCl$_3$/TMS; δ (ppm).

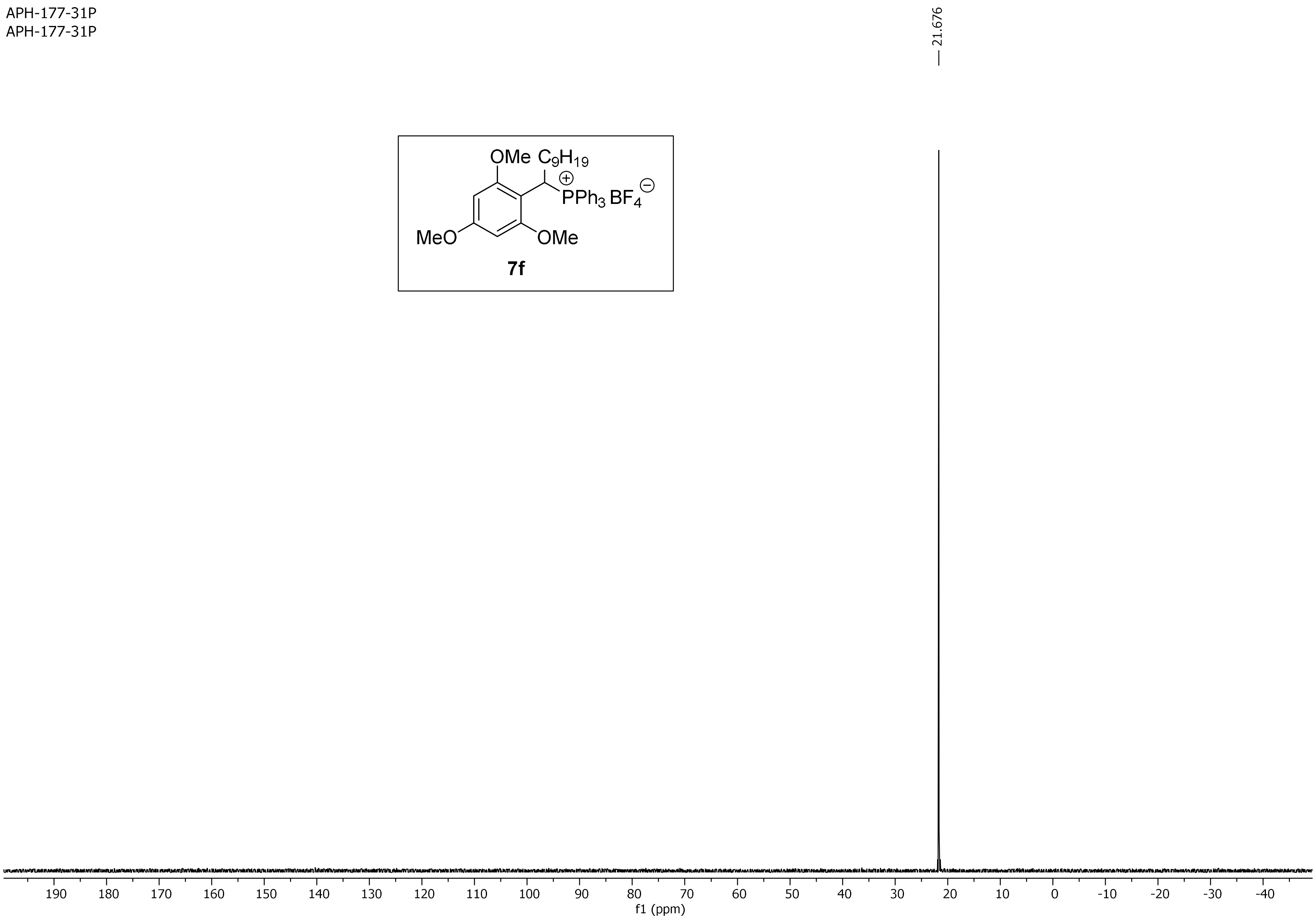

^{31}P NMR spectrum of 1-(2,4,6-trimethoxyphenyl)decyltriphenylphosphonium tetrafluoroborate (**7f**); 161.9 MHz/$CDCl_3$; δ (ppm).

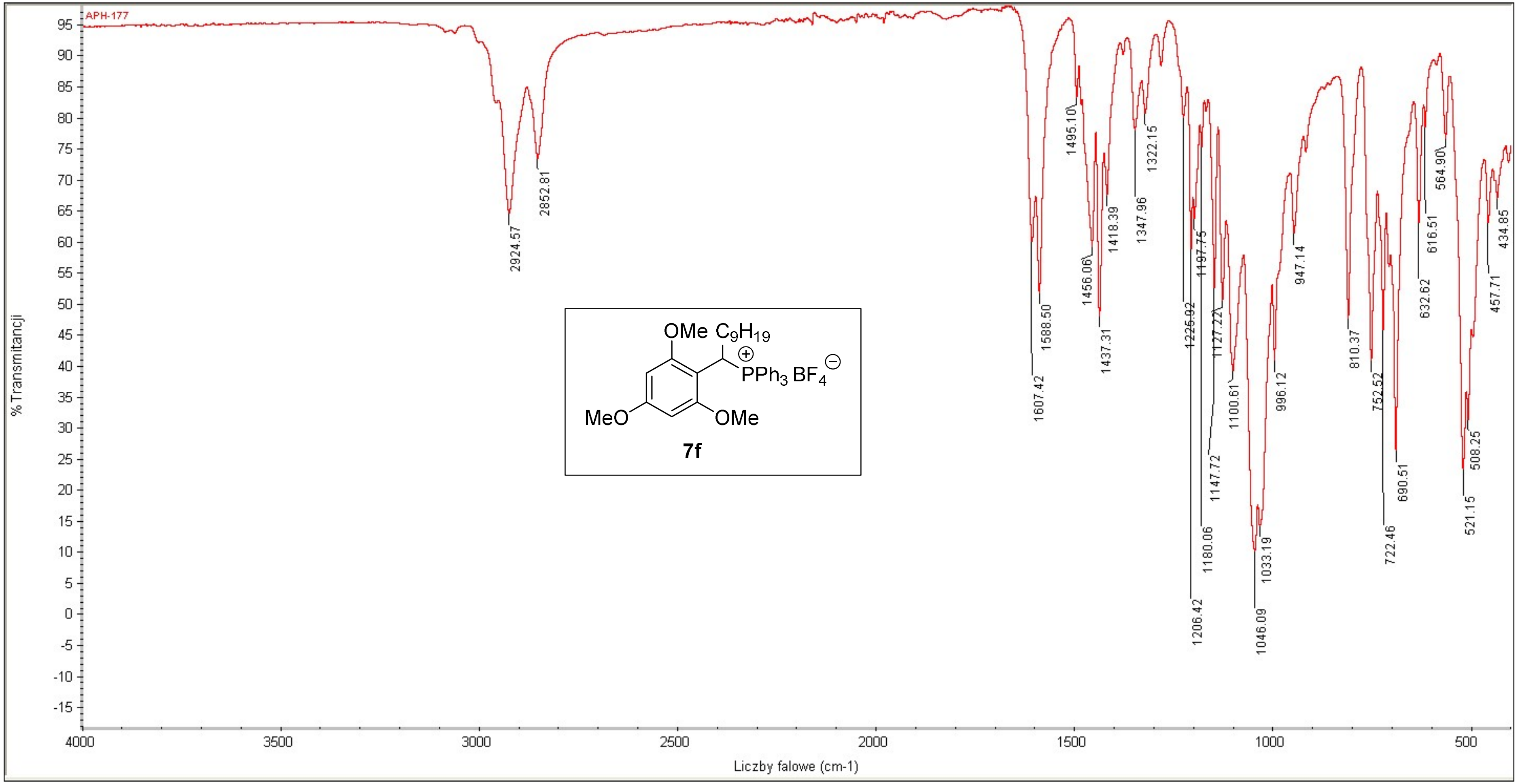

IR spectrum of 1-(2,4,6-trimethoxyphenyl)decyltriphenylphosphonium tetrafluoroborate (**7f**); ATR (cm^{-1}).

Tolerance = 40.0 mDa / DBE: min = -10.0, max = 100.0
Element prediction: Off
Number of isotope peaks used for i-FIT = 2
Monoisotopic Mass, Even Electron Ions
8 formula(e) evaluated with 1 results within limits (up to 3 closest results for each mass)
Elements Used:

Mass	RA	Calc. Mass	mDa	PPM	DBE	Formula	i-FIT	i-FIT Norm	Fit Conf %	C	H	O	P
569.3185	100.00	569.3185	0.0	0.0	15.5	C37 H46 O3 P	488.8	n/a	n/a	37	46	3	1

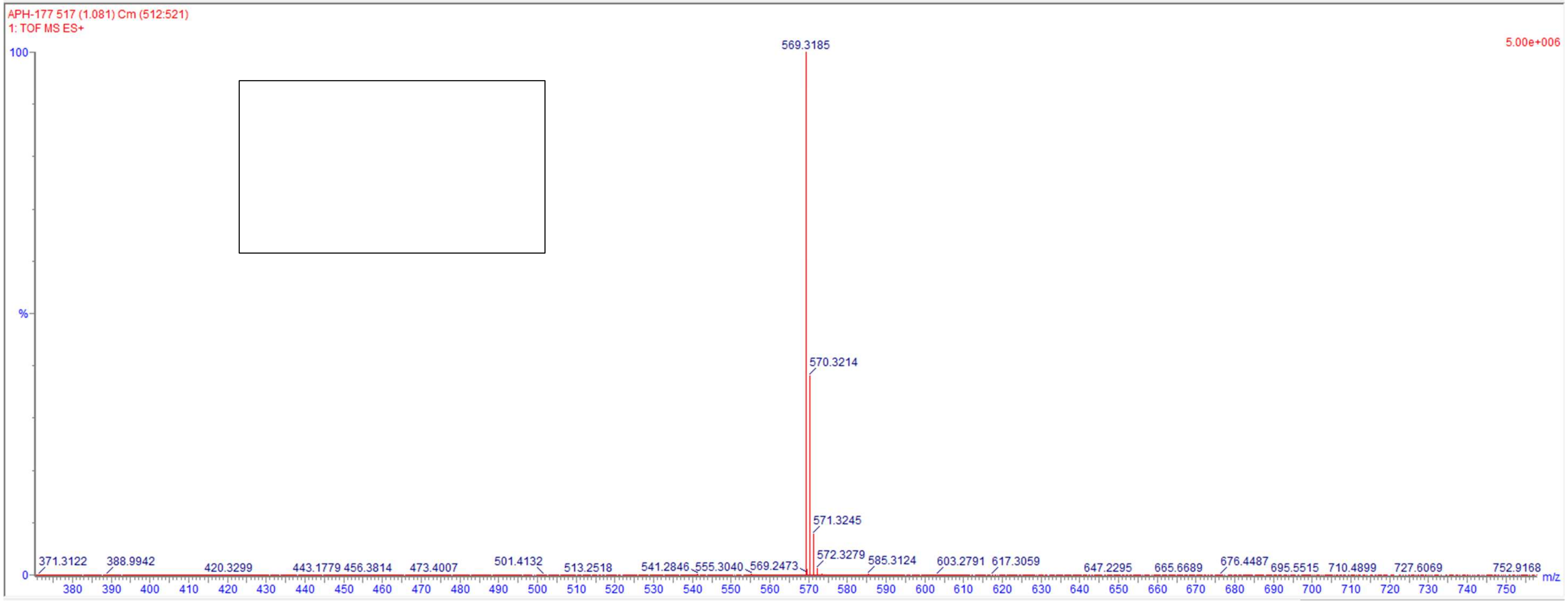

MS spectrum of 1-(2,4,6-trimethoxyphenyl)decyltriphenylphosphonium tetrafluoroborate (**7f**).

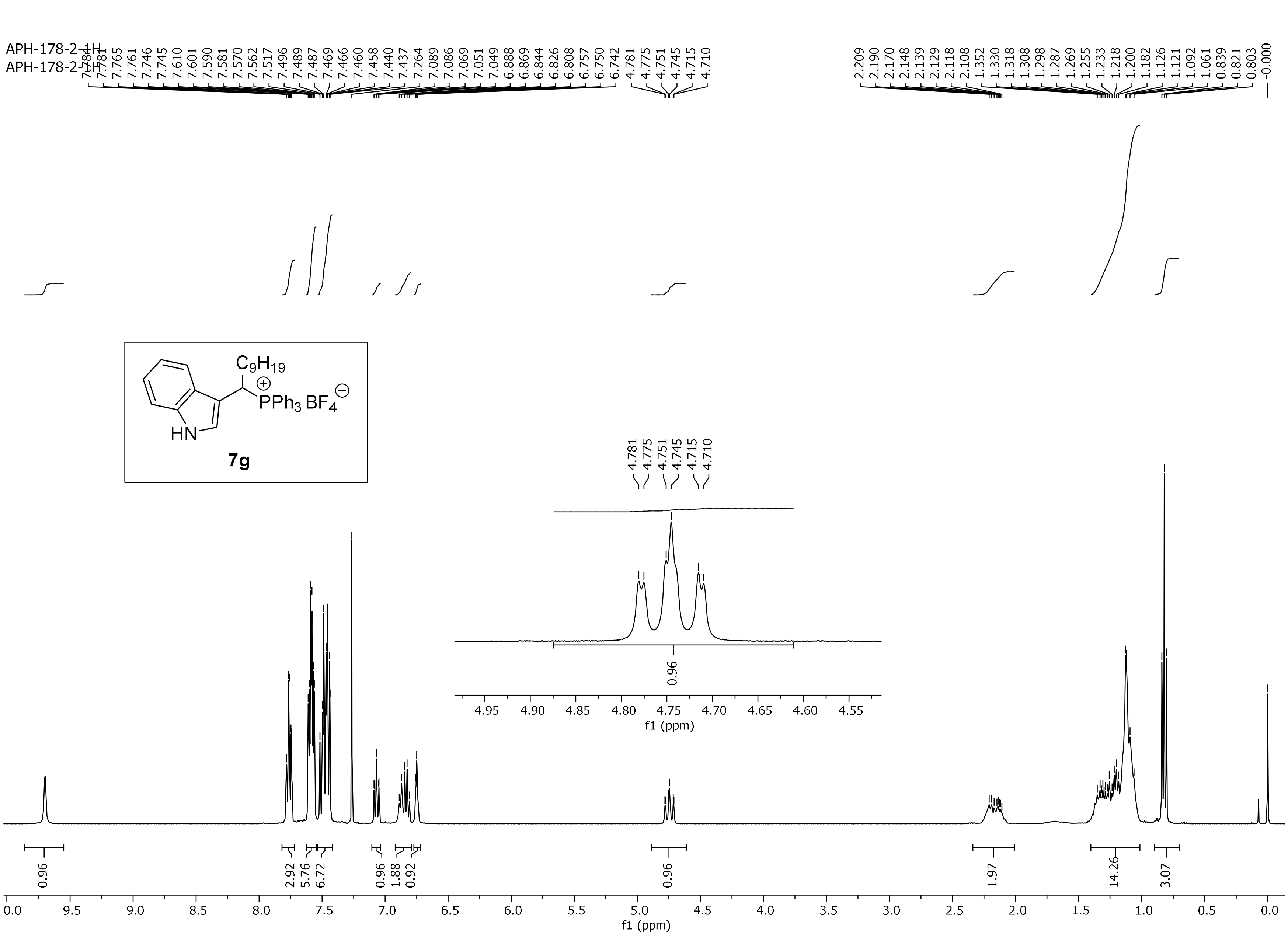

^{1}H NMR spectrum of 1-(*H*-indol-3-yl)decyltriphenylphosphonium tetrafluoroborate (**7g**); 400 MHz/$CDCl_3$/TMS; *δ* (ppm).

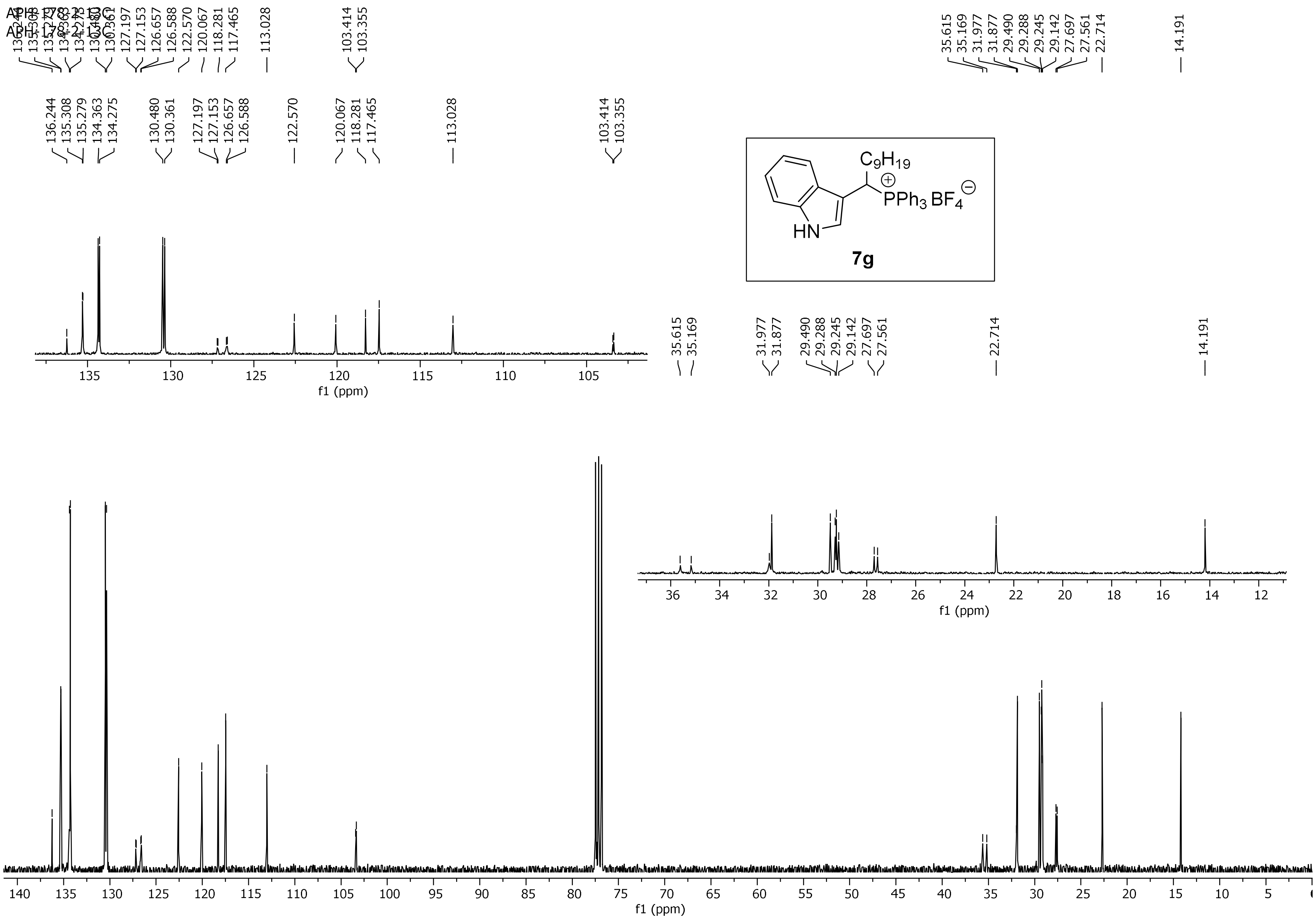

^{13}C NMR spectrum of 1-(*H*-indol-3-yl)decyltriphenylphosphonium tetrafluoroborate (**7g**); 100 MHz/$CDCl_3$/TMS; δ (ppm).

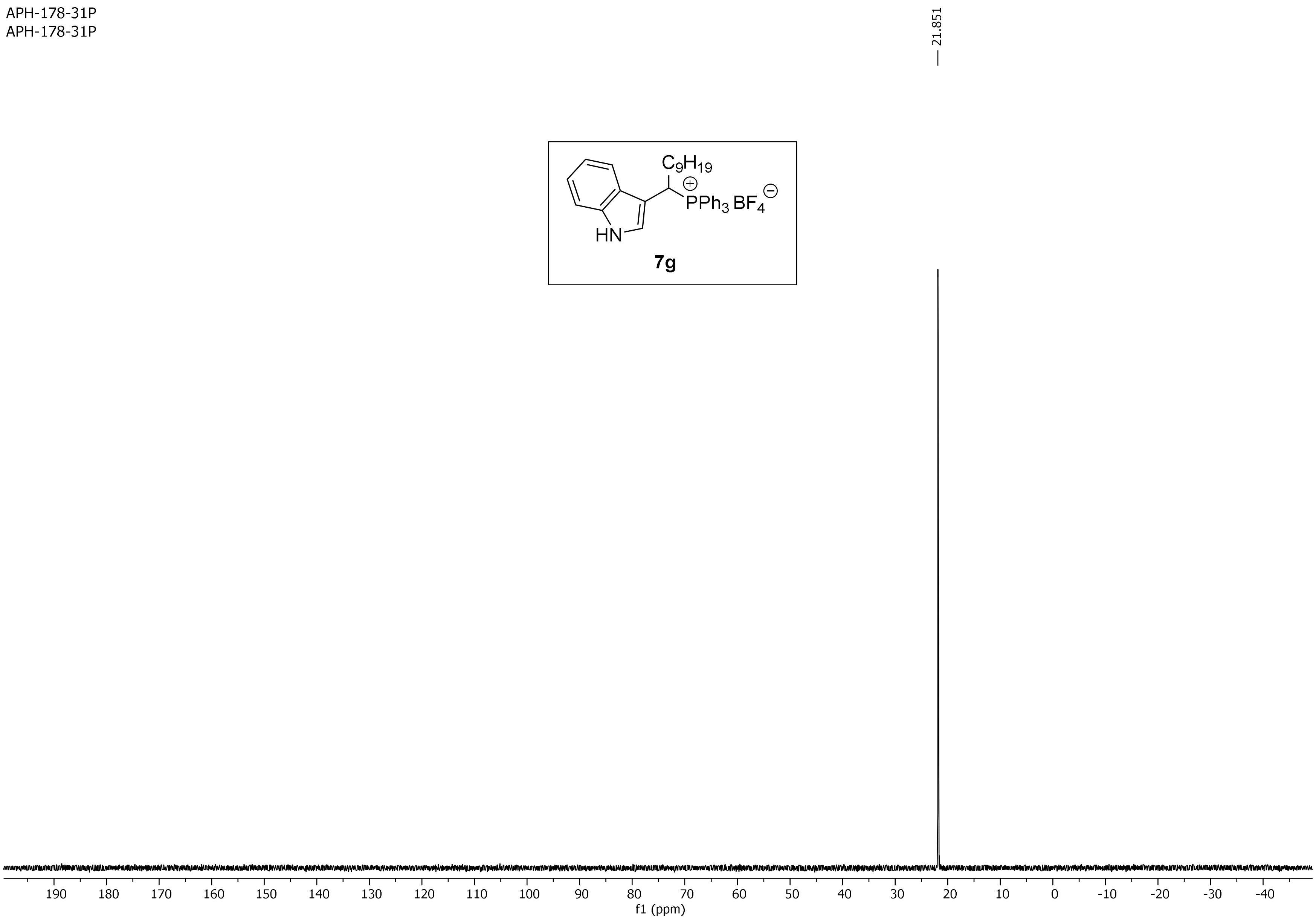

^{31}P NMR spectrum of 1-(*H*-indol-3-yl)decyltriphenylphosphonium tetrafluoroborate (**7g**); 161.9 MHz/$CDCl_3$; δ (ppm).

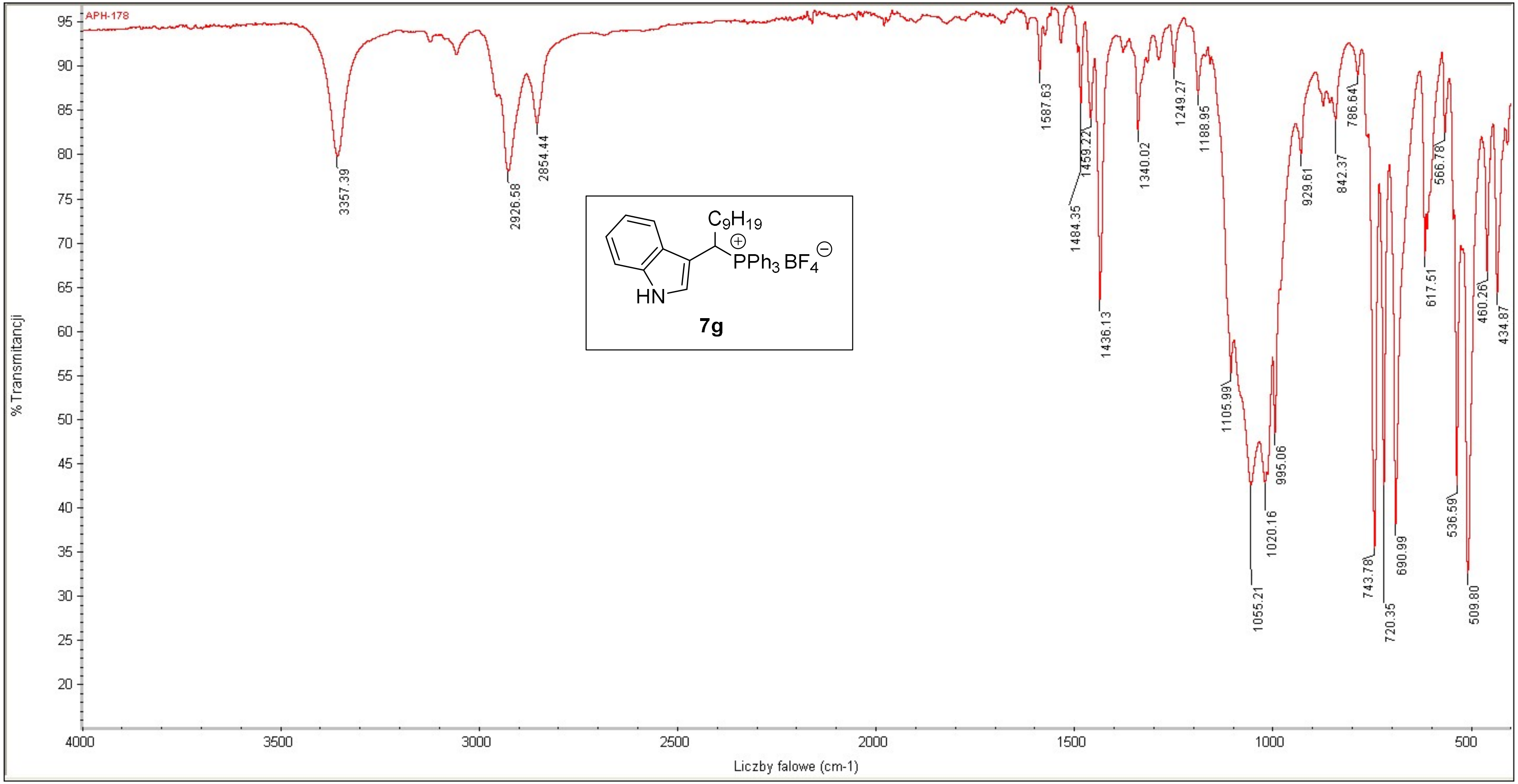

IR spectrum of 1-(*H*-indol-3-yl)decyltriphenylphosphonium tetrafluoroborate (**7g**); ATR (cm-1).

Tolerance = 40.0 mDa / DBE: min = -10.0, max = 100.0
Element prediction: Off
Number of isotope peaks used for i-FIT = 2
Monoisotopic Mass, Even Electron Ions
8 formula(e) evaluated with 1 results within limits (up to 3 closest results for each mass)
Elements Used:

Mass	RA	Calc. Mass	mDa	PPM	DBE	Formula	i-FIT	i-FIT Norm	Fit Conf %	C	H	N	P
518.2982	100.00	518.2977	0.5	1.0	17.5	C36 H41 N P	545.9	n/a	n/a	36	41	1	1

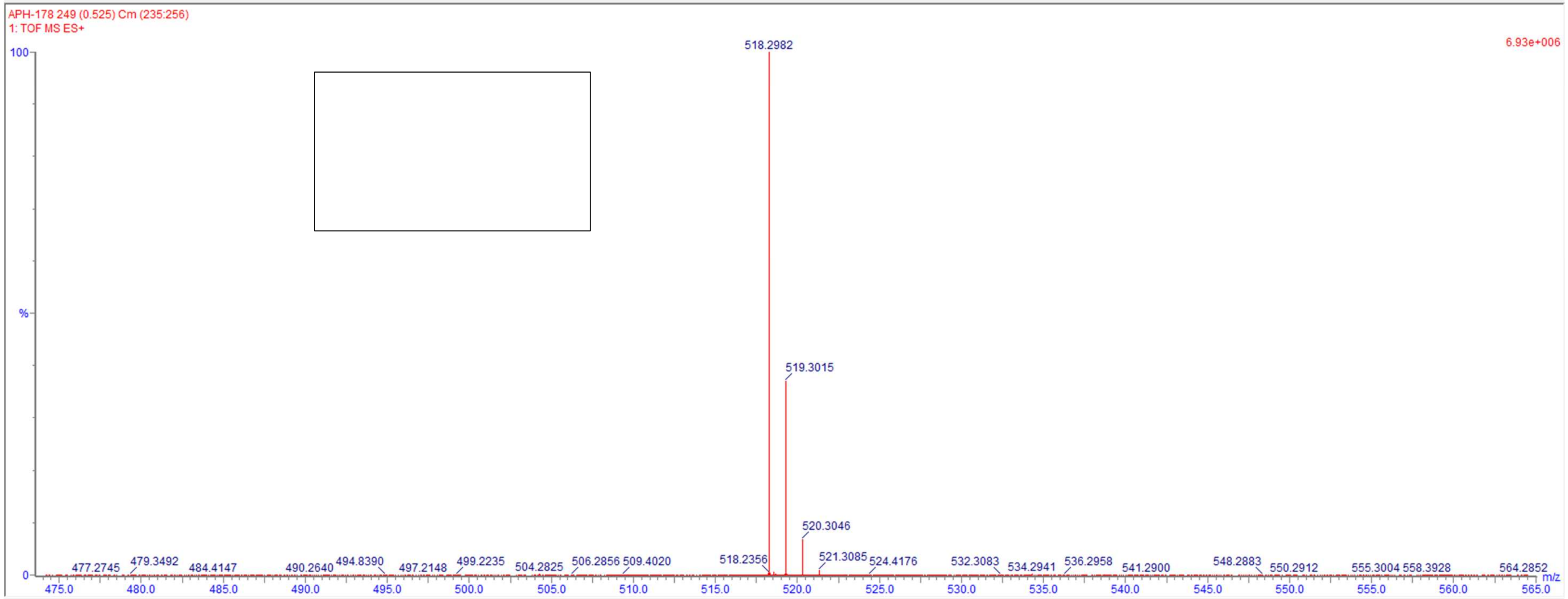

MS spectrum of 1-(*H*-indol-3-yl)decyltriphenylphosphonium tetrafluoroborate (**7g**).

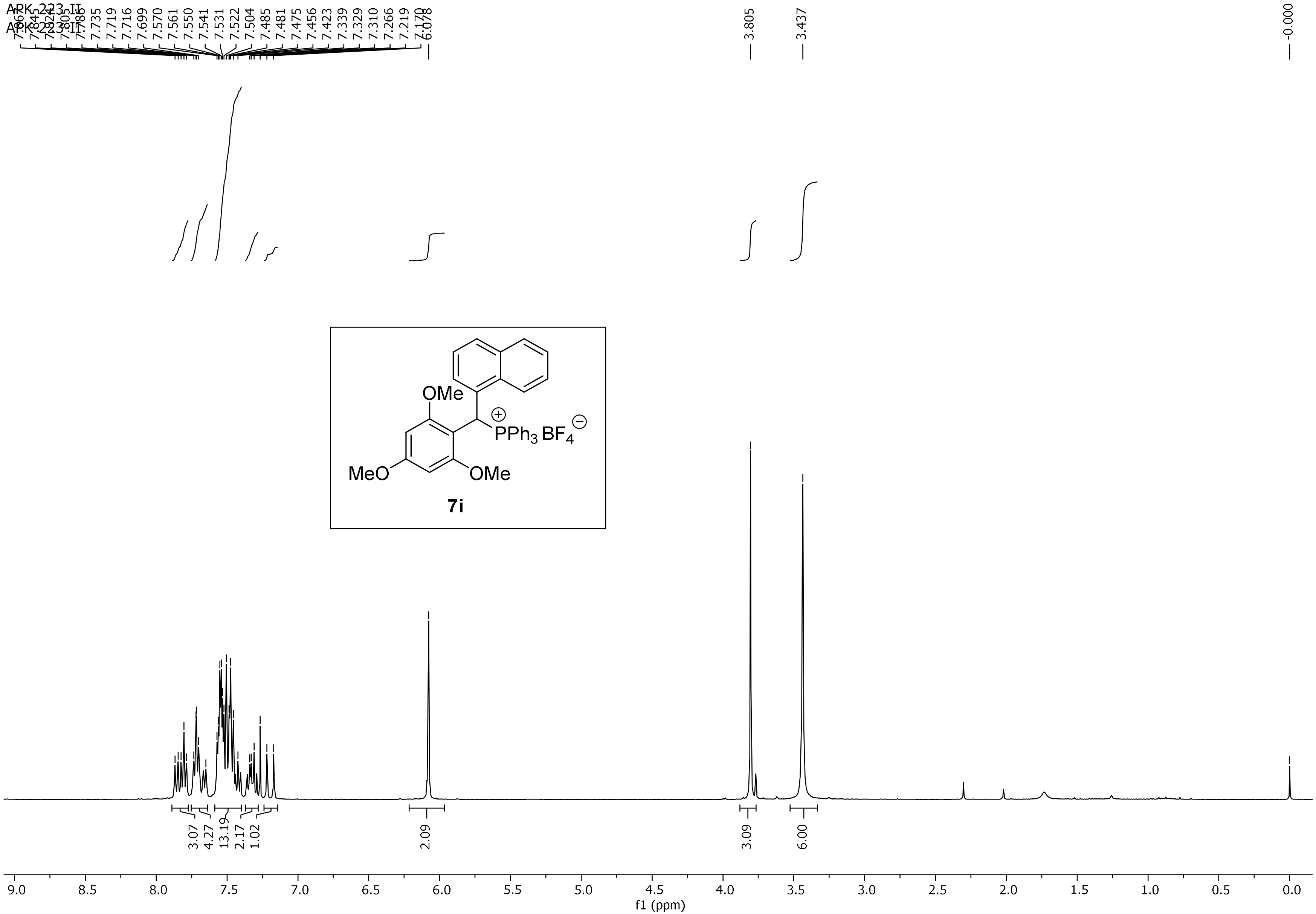

^{1}H NMR spectrum of 1-(naphthalen-1-yl)-1-(2,4,6-trimethoxyphenyl)methyltriphenylphosphonium tetrafluoroborate (**7i**); 400 MHz/$CDCl_3$/TMS; δ (ppm).

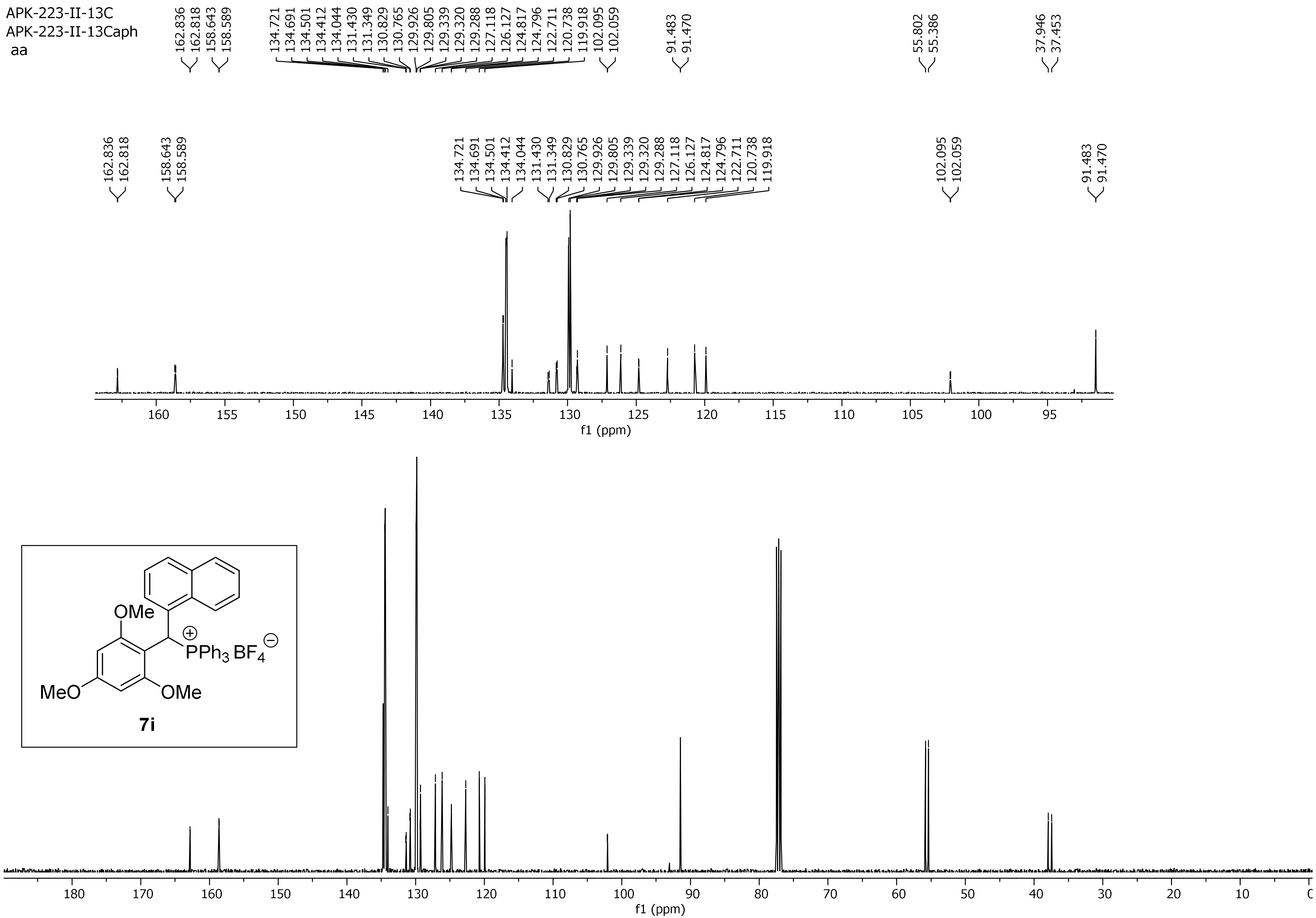

^{13}C NMR spectrum of 1-(naphthalen-1-yl)-1-(2,4,6-trimethoxyphenyl)methyltriphenylphosphonium tetrafluoroborate (**7i**); 100 MHz/$CDCl_3$/TMS; δ (ppm).

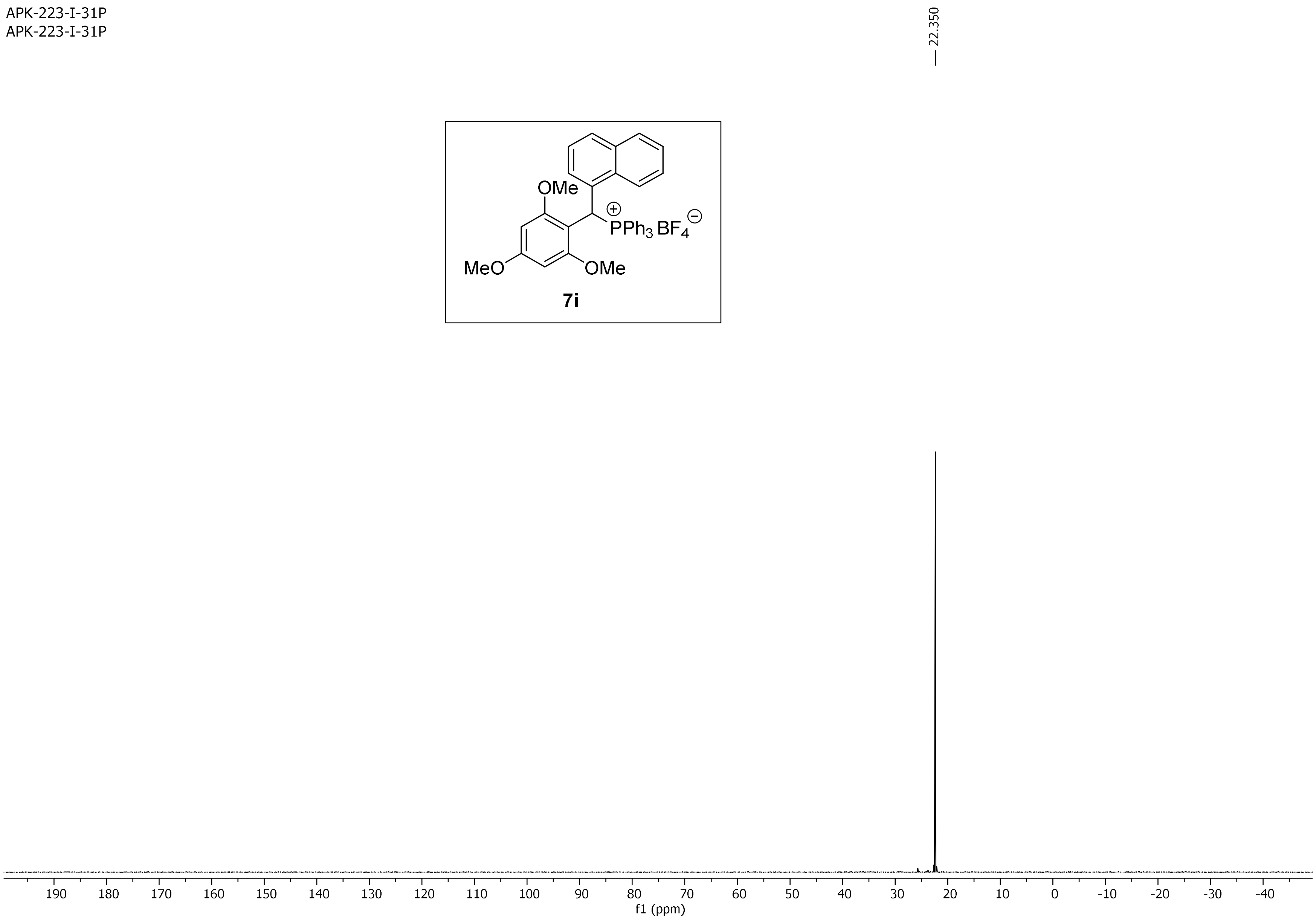

^{31}P NMR spectrum of 1-(naphthalen-1-yl)-1-(2,4,6-trimethoxyphenyl)methyltriphenylphosphonium tetrafluoroborate (**7i**); 161.9 MHz/$CDCl_3$; δ (ppm).

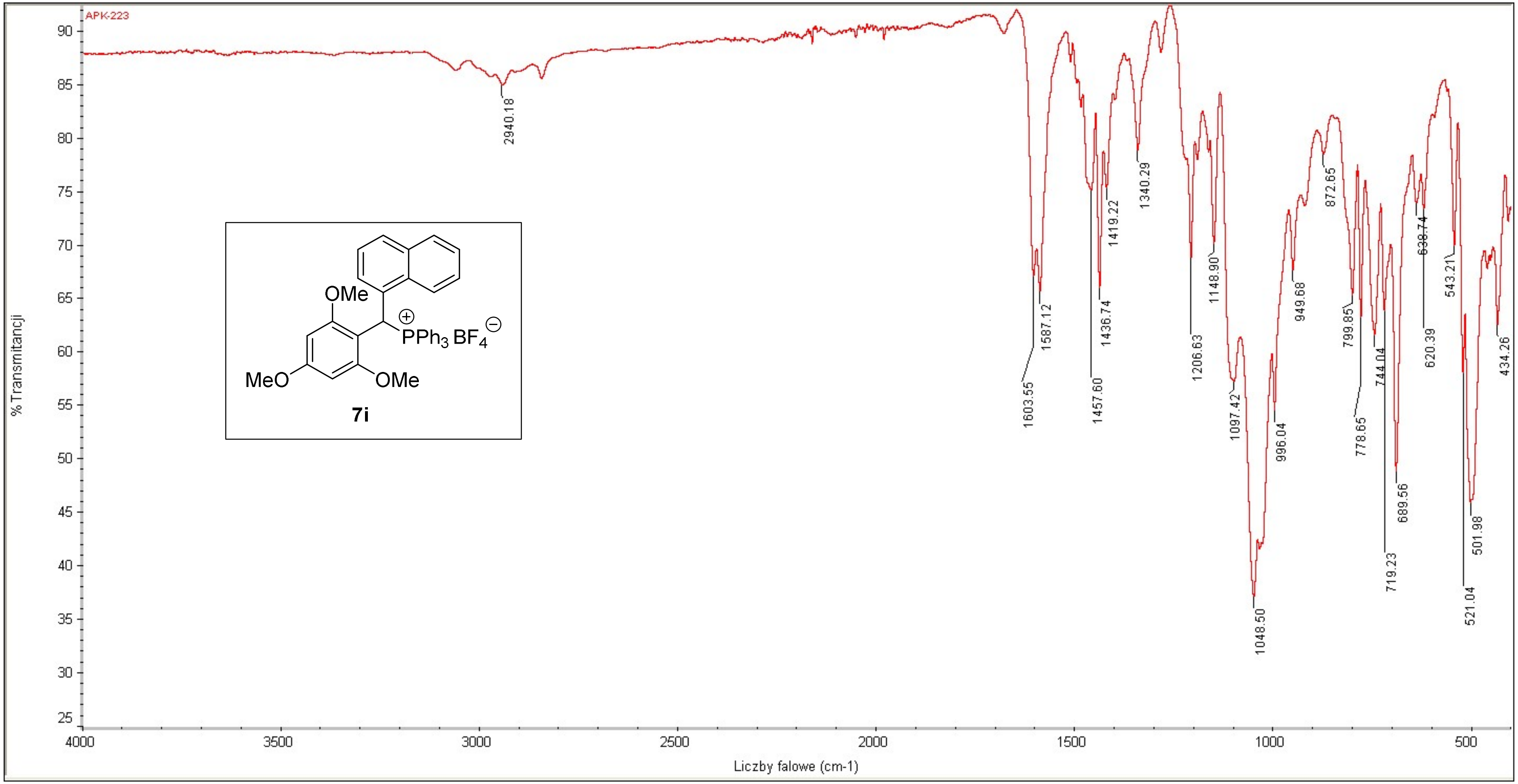

IR spectrum of 1-(naphthalen-1-yl)-1-(2,4,6-trimethoxyphenyl)methyltriphenylphosphonium tetrafluoroborate (**7i**); ATR (cm^{-1}).

Tolerance = 40.0 mDa / DBE: min = -10.0, max = 100.0
Element prediction: Off
Number of isotope peaks used for i-FIT = 2
Monoisotopic Mass, Even Electron Ions
8 formula(e) evaluated with 1 results within limits (up to 3 closest results for each mass)
Elements Used:

Mass	RA	Calc. Mass	mDa	PPM	DBE	Formula	i-FIT	i-FIT Norm	Fit Conf %	C	H	O	P
569.2252	100.00	569.2246	0.6	1.1	22.5	C38 H34 O3 P	499.9	n/a	n/a	38	34	3	1

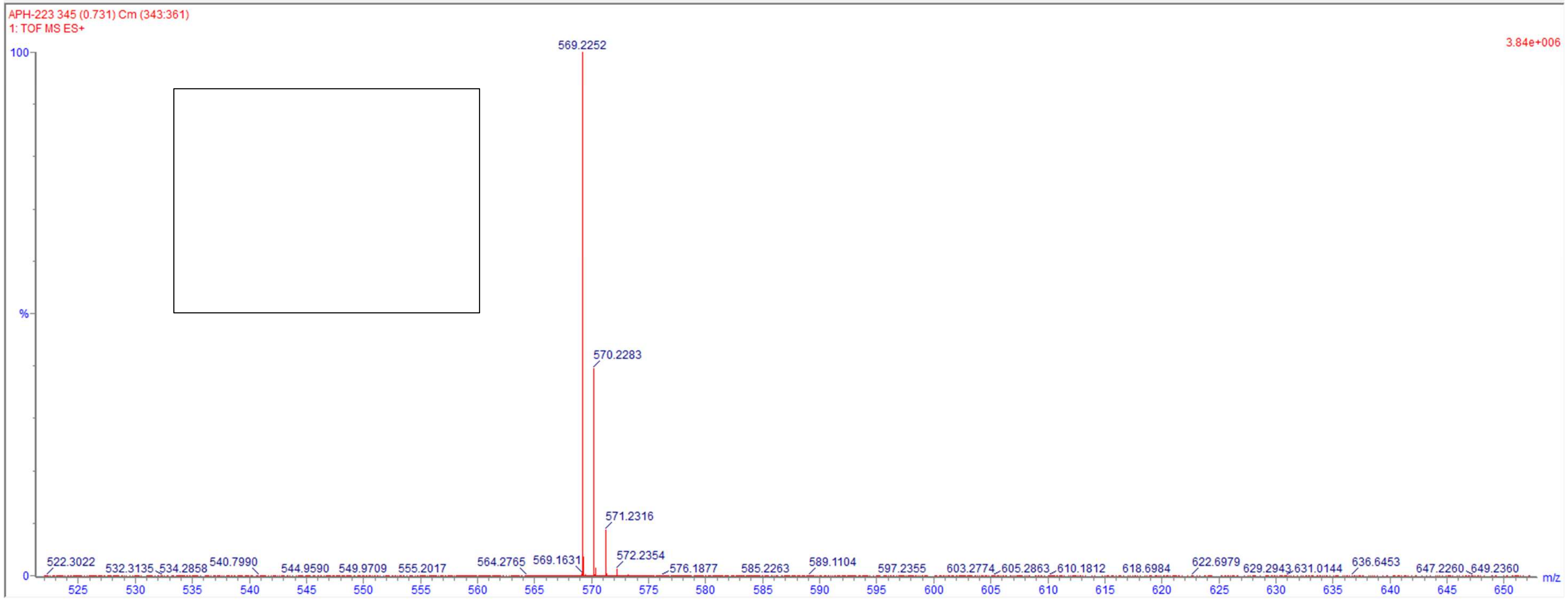

MS spectrum of 1-(naphthalen-1-yl)-1-(2,4,6-trimethoxyphenyl)methyltriphenylphosphonium tetrafluoroborate (**7i**).

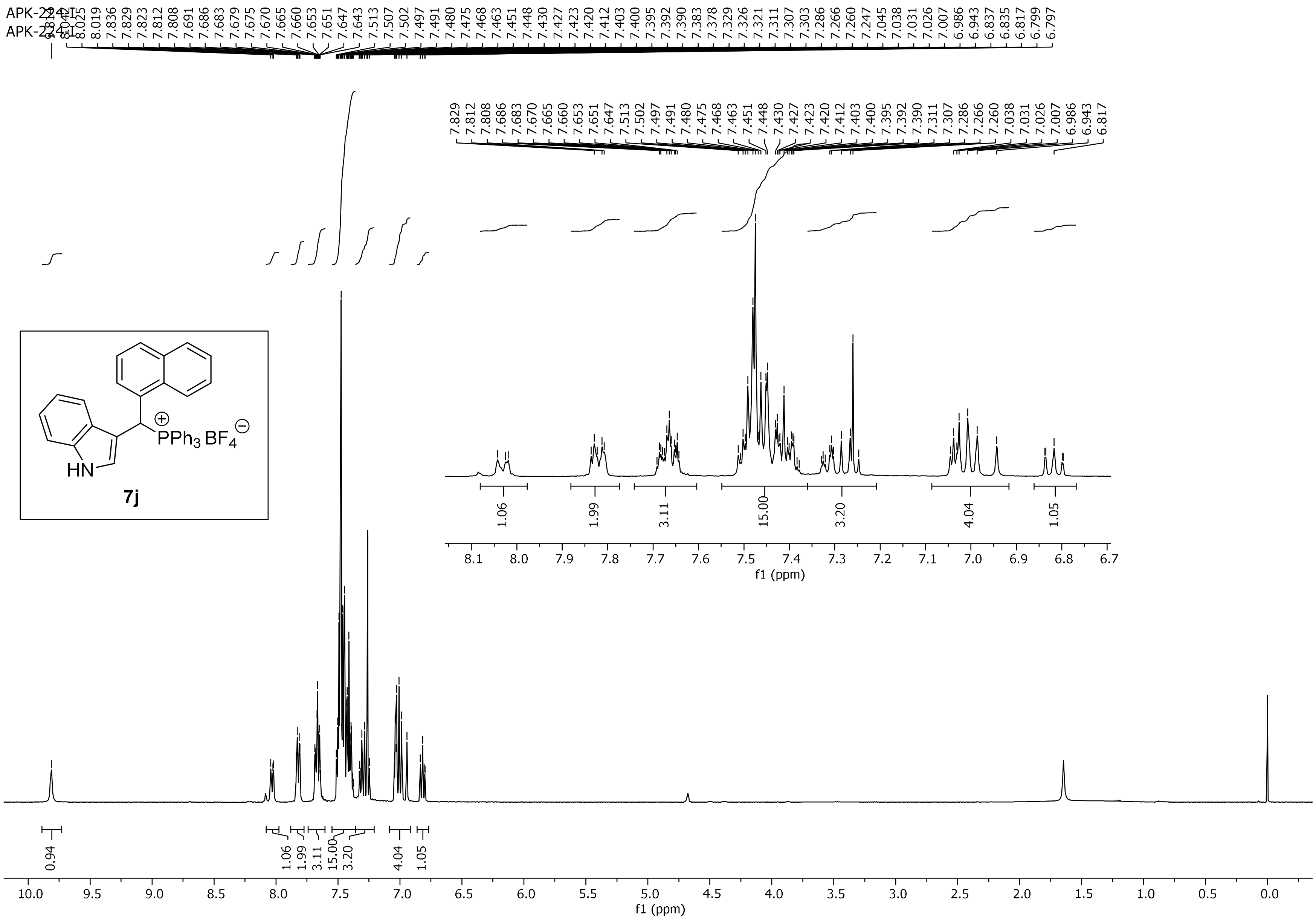

^{1}H NMR spectrum of 1-(*H*-indol-3-yl)-1-(naphthalen-1-yl)methyltriphenylphosphonium tetrafluoroborate (**7j**); 400 MHz/$CDCl_3$/TMS; δ (ppm).

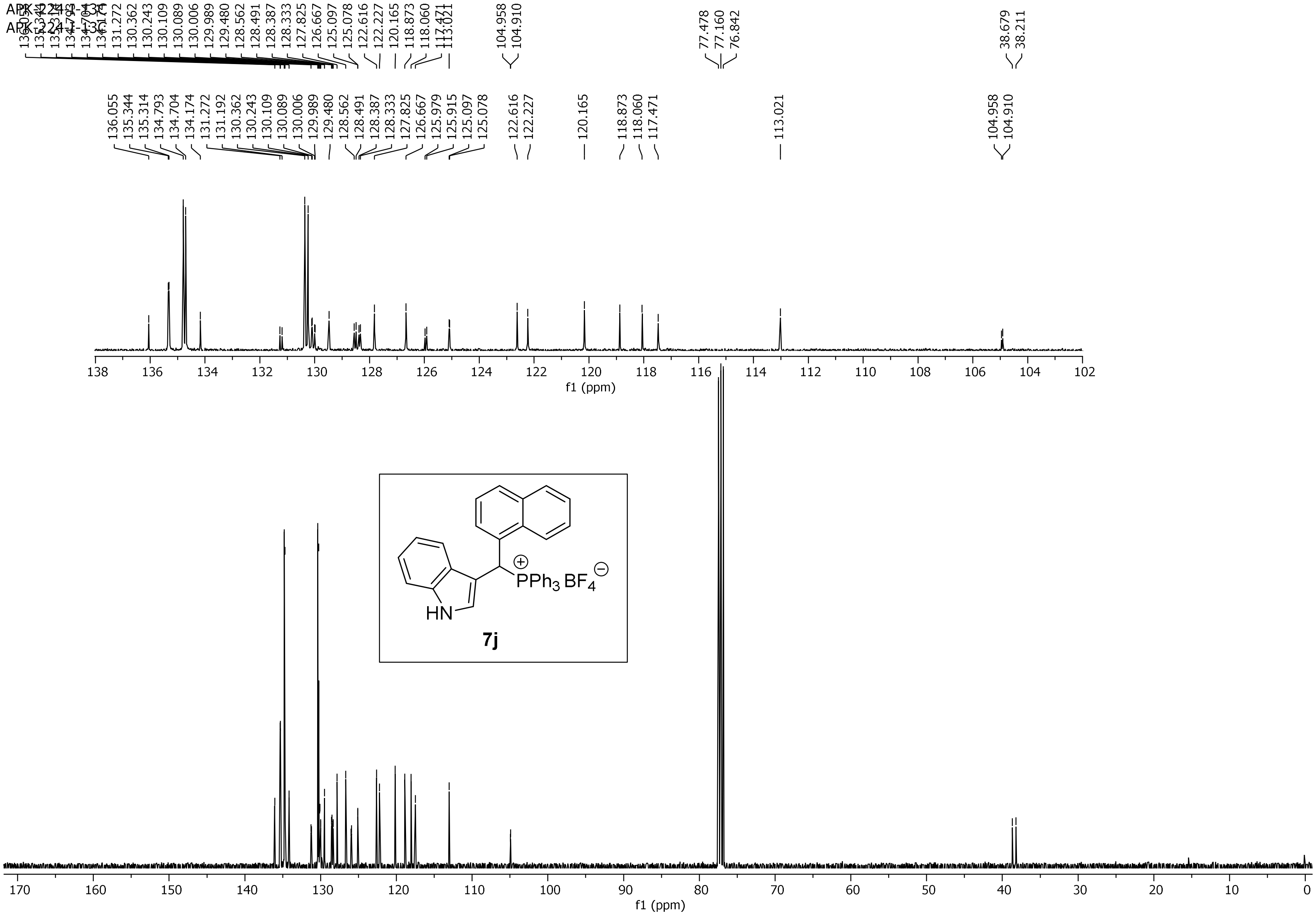

^{13}C NMR spectrum of 1-(*H*-indol-3-yl)-1-(naphthalen-1-yl)methyltriphenylphosphonium tetrafluoroborate (**7j**); 100 MHz/$CDCl_3$/TMS; δ (ppm).

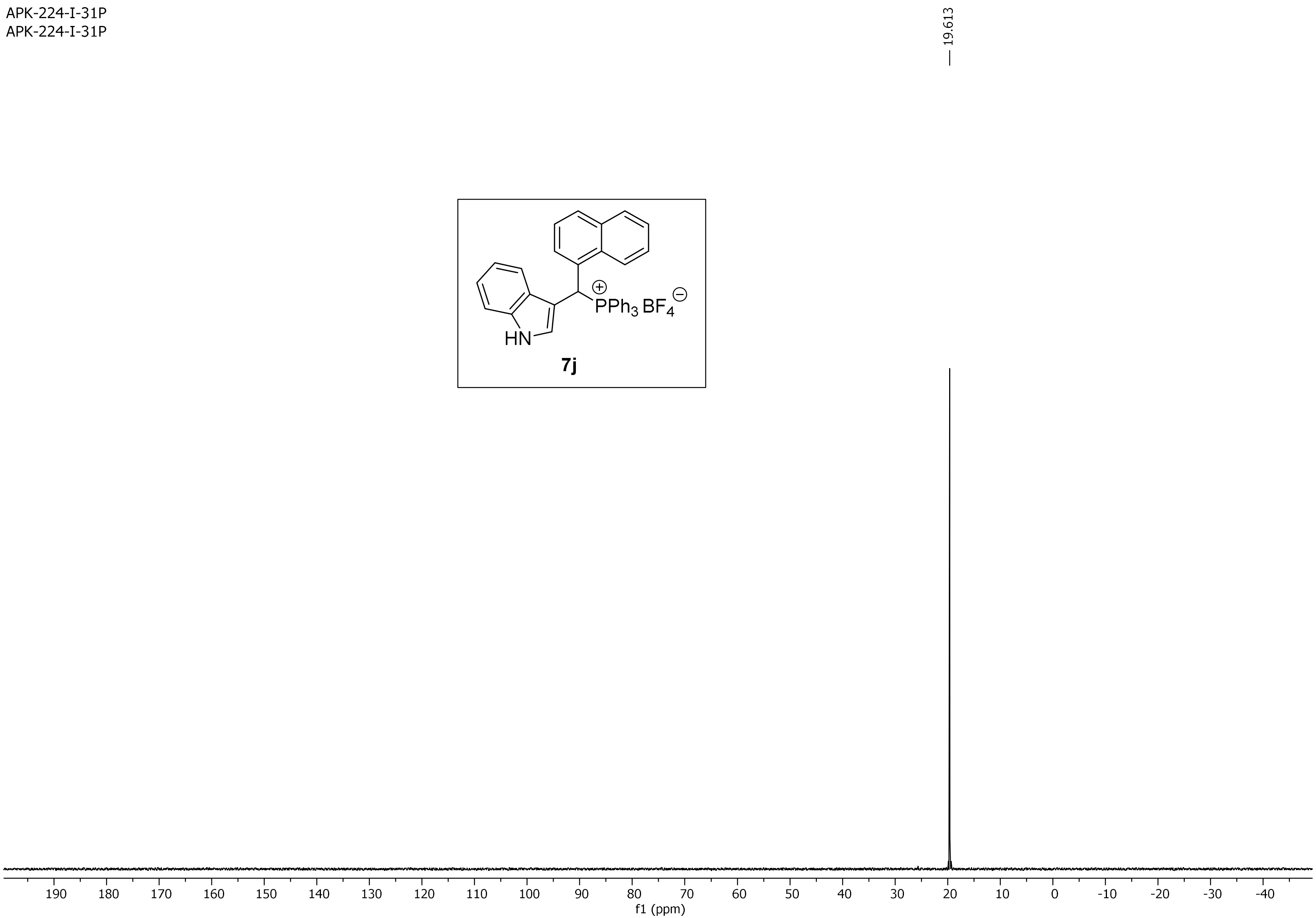

^{31}P NMR spectrum of 1-(*H*-indol-3-yl)-1-(naphthalen-1-yl)methyltriphenylphosphonium tetrafluoroborate (**7j**); 161.9 MHz/$CDCl_3$; δ (ppm).

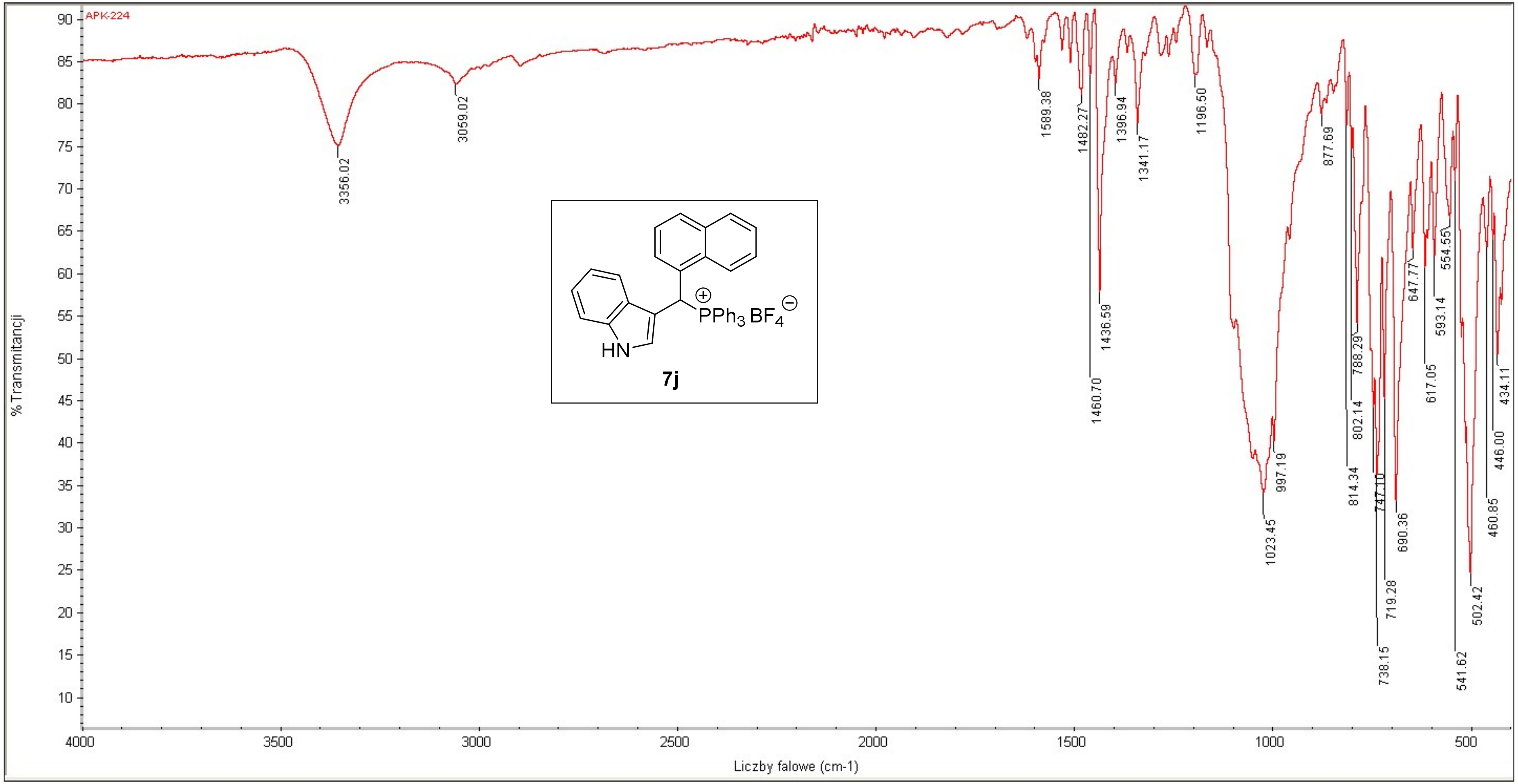

IR spectrum of 1-(*H*-indol-3-yl)-1-(naphthalen-1-yl)methyltriphenylphosphonium tetrafluoroborate (**7j**); ATR (cm^{-1}).

Tolerance = 40.0 mDa / DBE: min = -10.0, max = 100.0
Element prediction: Off
Number of isotope peaks used for i-FIT = 2
Monoisotopic Mass, Even Electron Ions
8 formula(e) evaluated with 1 results within limits (up to 3 closest results for each mass)
Elements Used:

Mass	RA	Calc. Mass	mDa	PPM	DBE	Formula	i-FIT	i-FIT Norm	Fit Conf %	C	H	N	P
518.2036	100.00	518.2038	-0.2	-0.4	24.5	C37 H29 N P	368.1	n/a	n/a	37	29	1	1

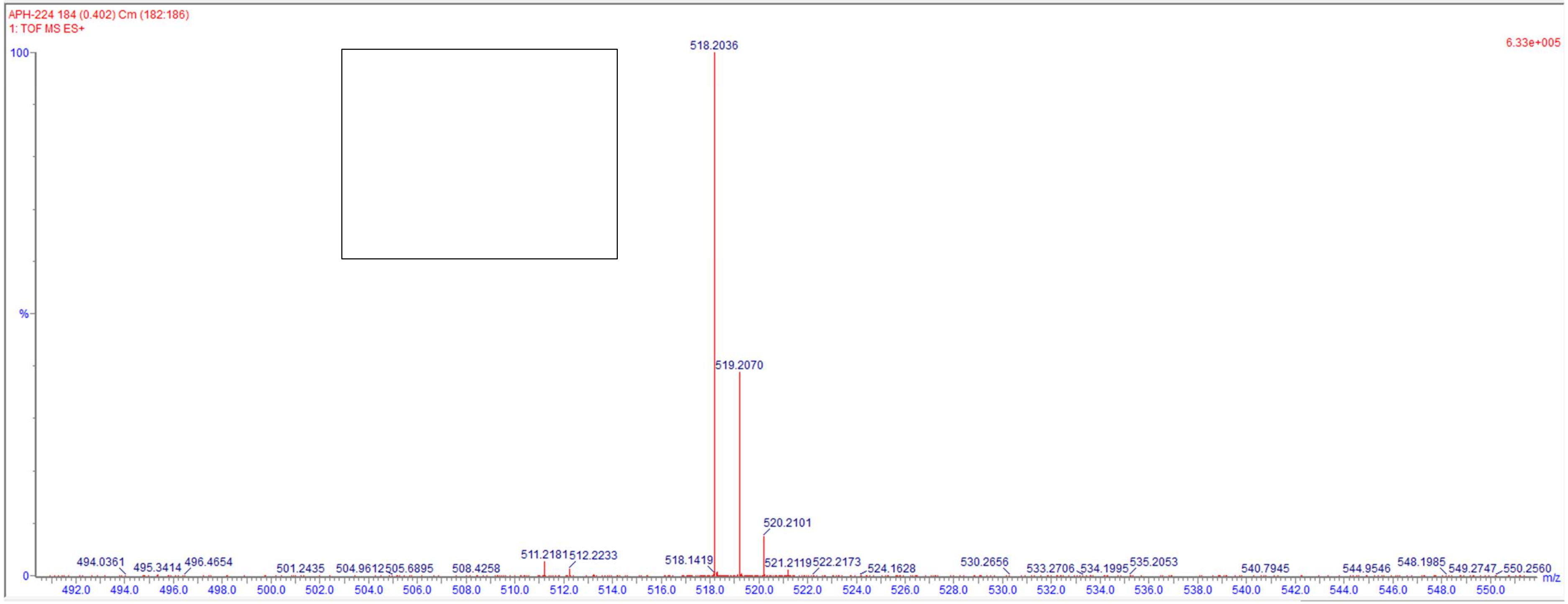

MS spectrum of 1-(*H*-indol-3-yl)-1-(naphthalen-1-yl)methyltriphenylphosphonium tetrafluoroborate (**7j**).

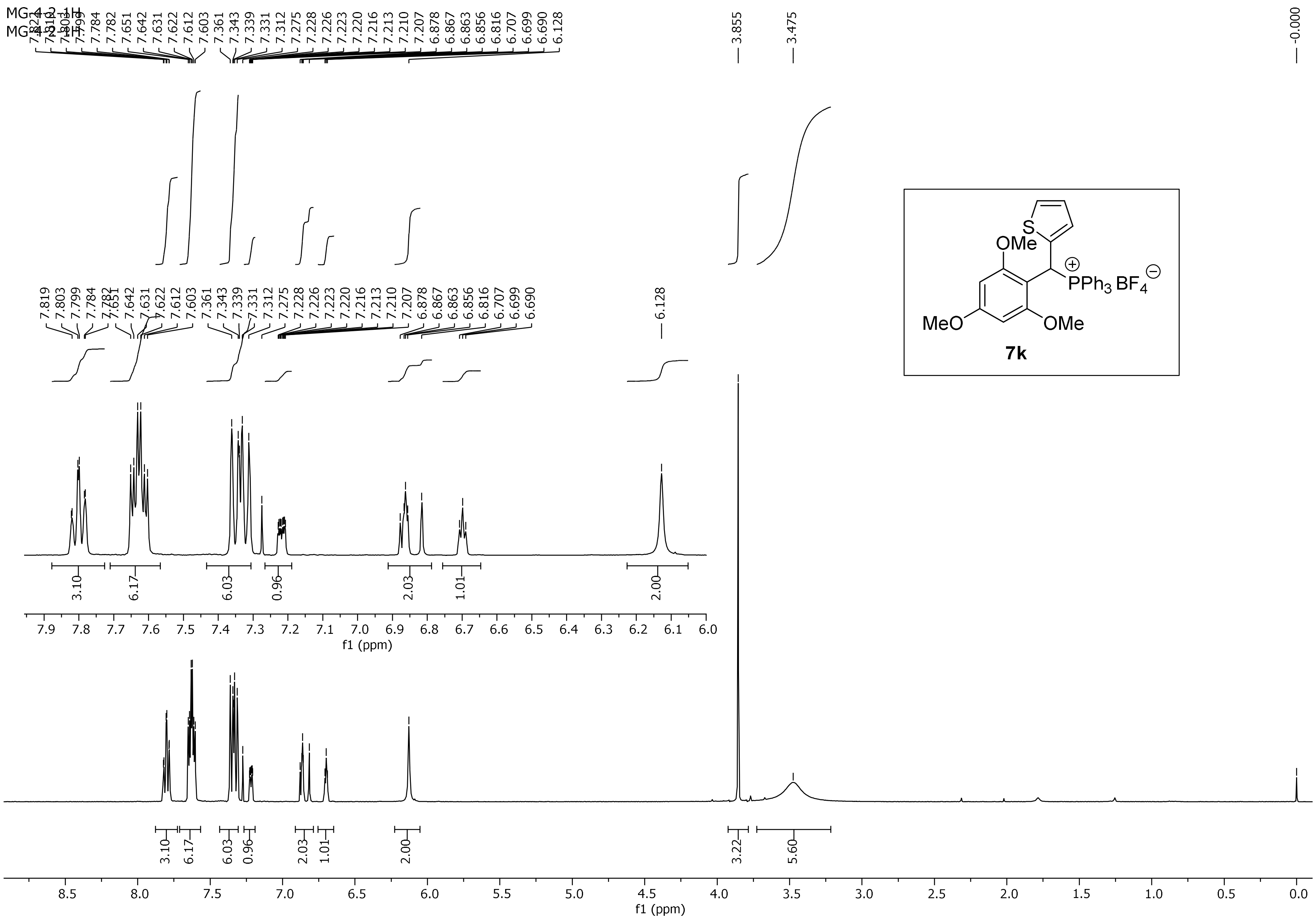

^{1}H NMR spectrum of 1-(tiophen-2-yl)-1-(2,4,6-trimethoxyphenyl)methyltriphenylphosphonium tetrafluoroborate (**7k**); 400 MHz/$CDCl_3$/TMS; δ (ppm).

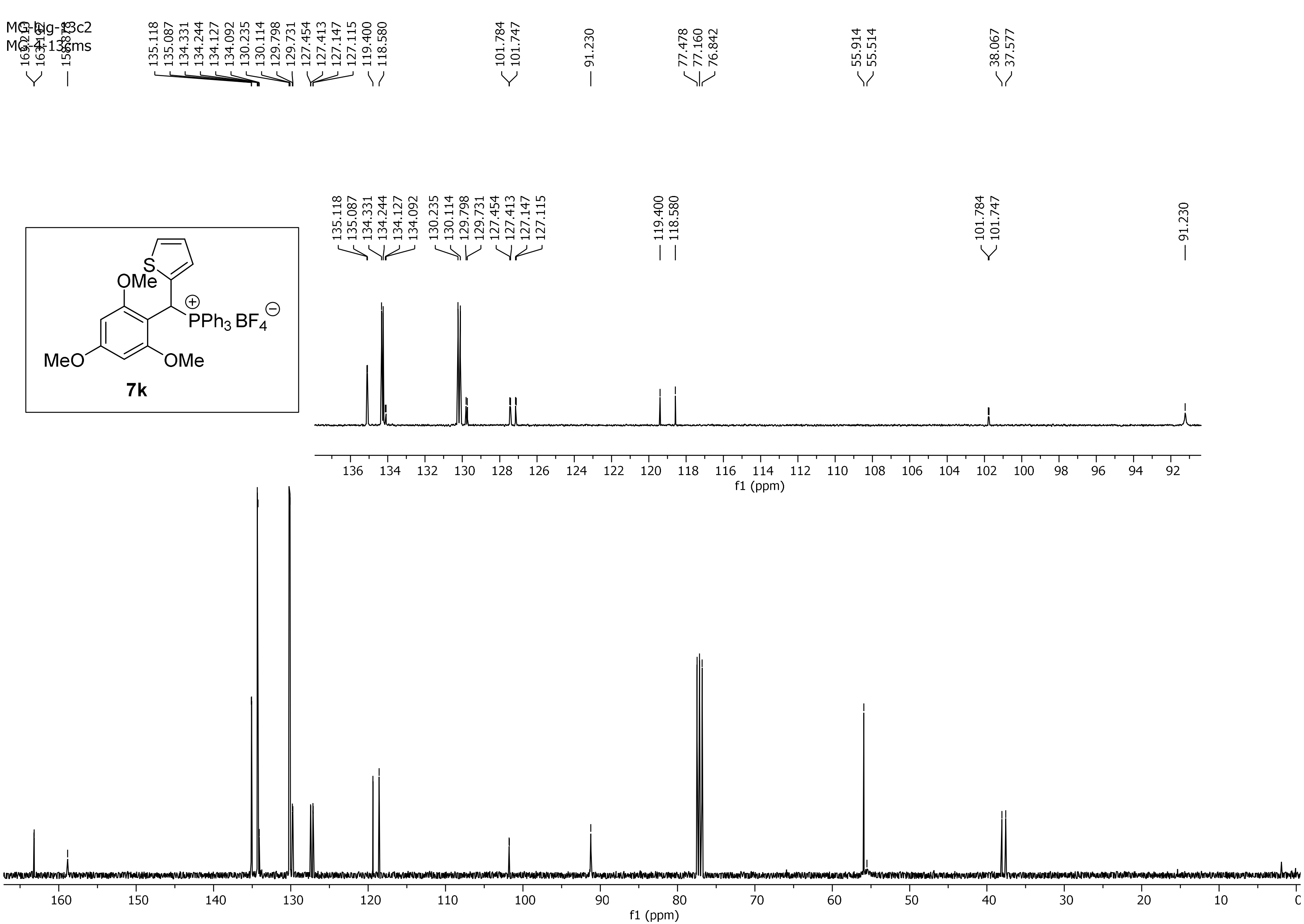

^{13}C NMR spectrum of 1-(tiophen-2-yl)-1-(2,4,6-trimethoxyphenyl)methyltriphenylphosphonium tetrafluoroborate (**7k**); 100 MHz/$CDCl_3$/TMS; δ (ppm).

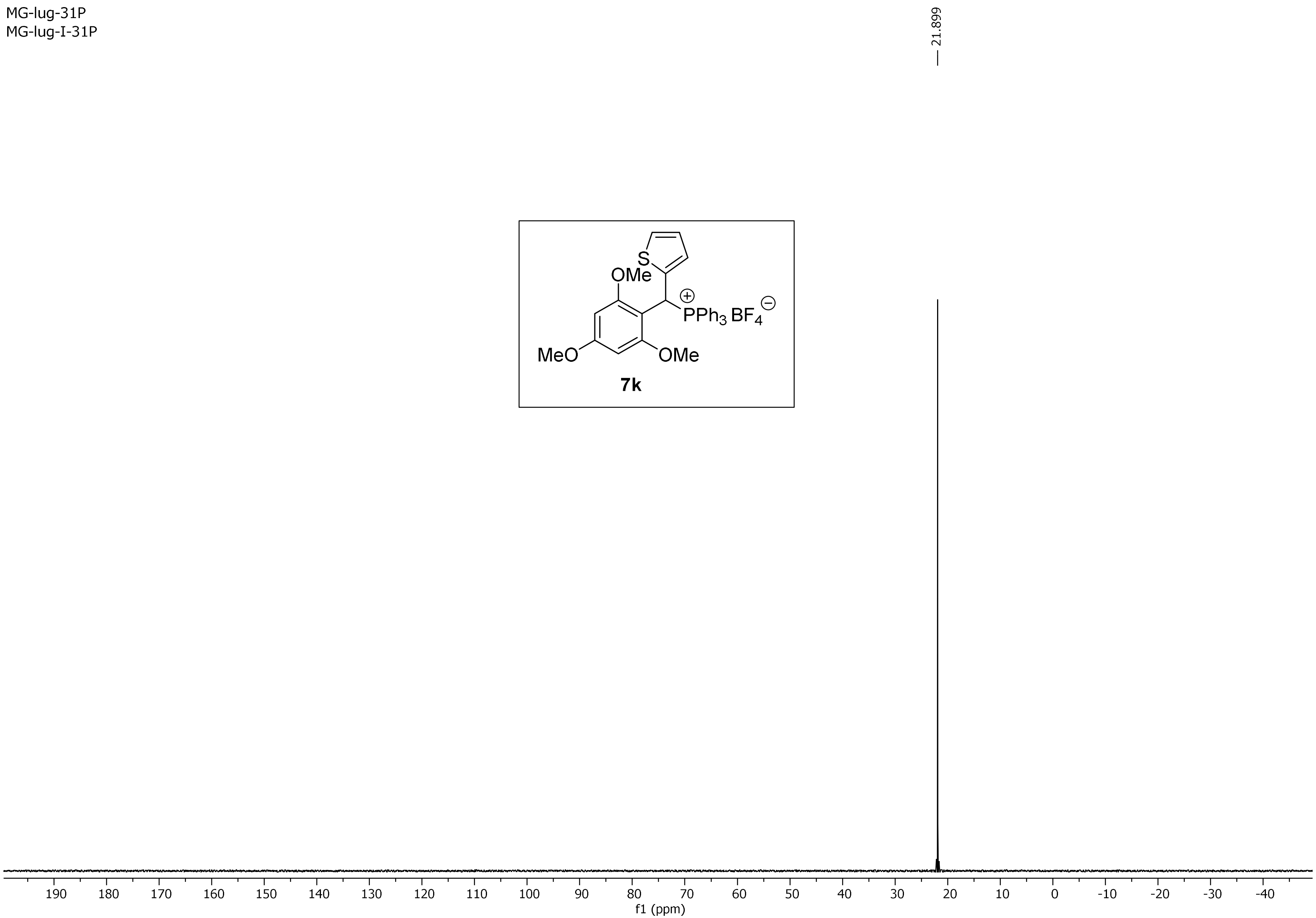

^{31}P NMR spectrum of 1-(tiophen-2-yl)-1-(2,4,6-trimethoxyphenyl)methyltriphenylphosphonium tetrafluoroborate (**7k**); 161.9 MHz/$CDCl_3$; δ (ppm).

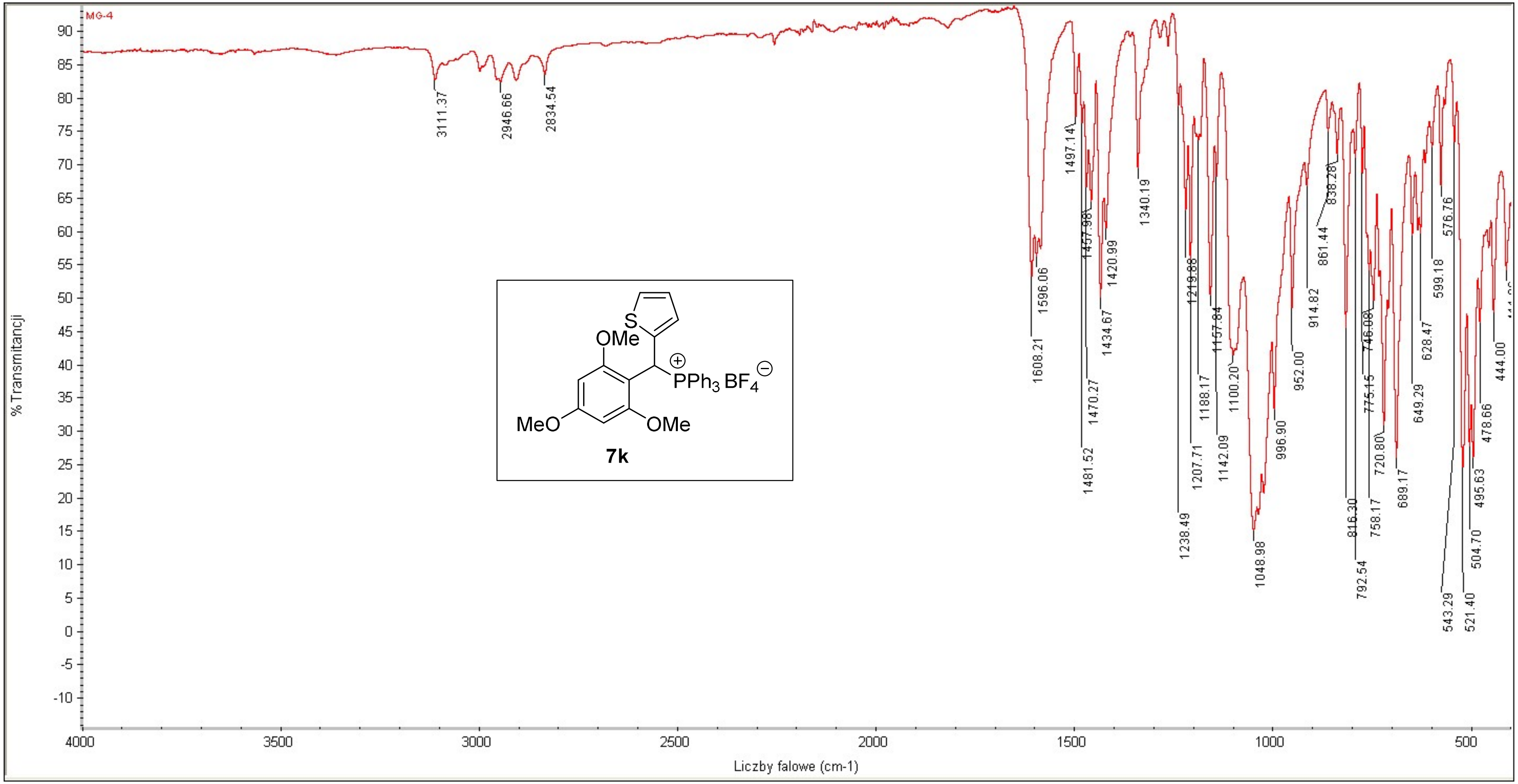

IR spectrum of 1-(tiophen-2-yl)-1-(2,4,6-trimethoxyphenyl)methyltriphenylphosphonium tetrafluoroborate (**7k**); ATR (cm^{-1}).

Tolerance = 40.0 mDa / DBE: min = -10.0, max = 100.0
Element prediction: Off
Number of isotope peaks used for i-FIT = 2
Monoisotopic Mass, Even Electron Ions
7 formula(e) evaluated with 1 results within limits (up to 3 closest results for each mass)
Elements Used:

Mass	RA	Calc. Mass	mDa	PPM	DBE	Formula	i-FIT	i-FIT Norm	Fit Conf %	C	H	O	P	S
525.1655	100.00	525.1653	0.2	0.4	18.5	C32 H30 O3 P S	278.2	n/a	n/a	32	30	3	1	1

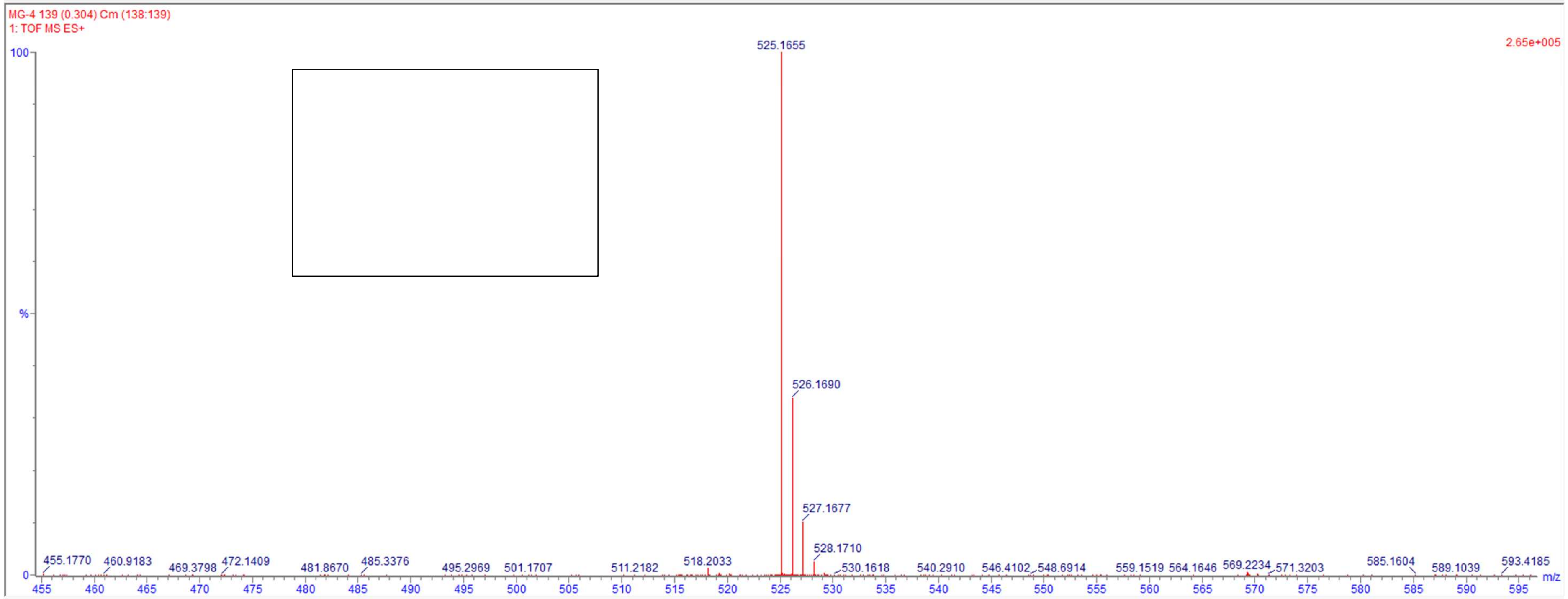

MS spectrum of 1-(tiophen-2-yl)-1-(2,4,6-trimethoxyphenyl)methyltriphenylphosphonium tetrafluoroborate (**7k**).

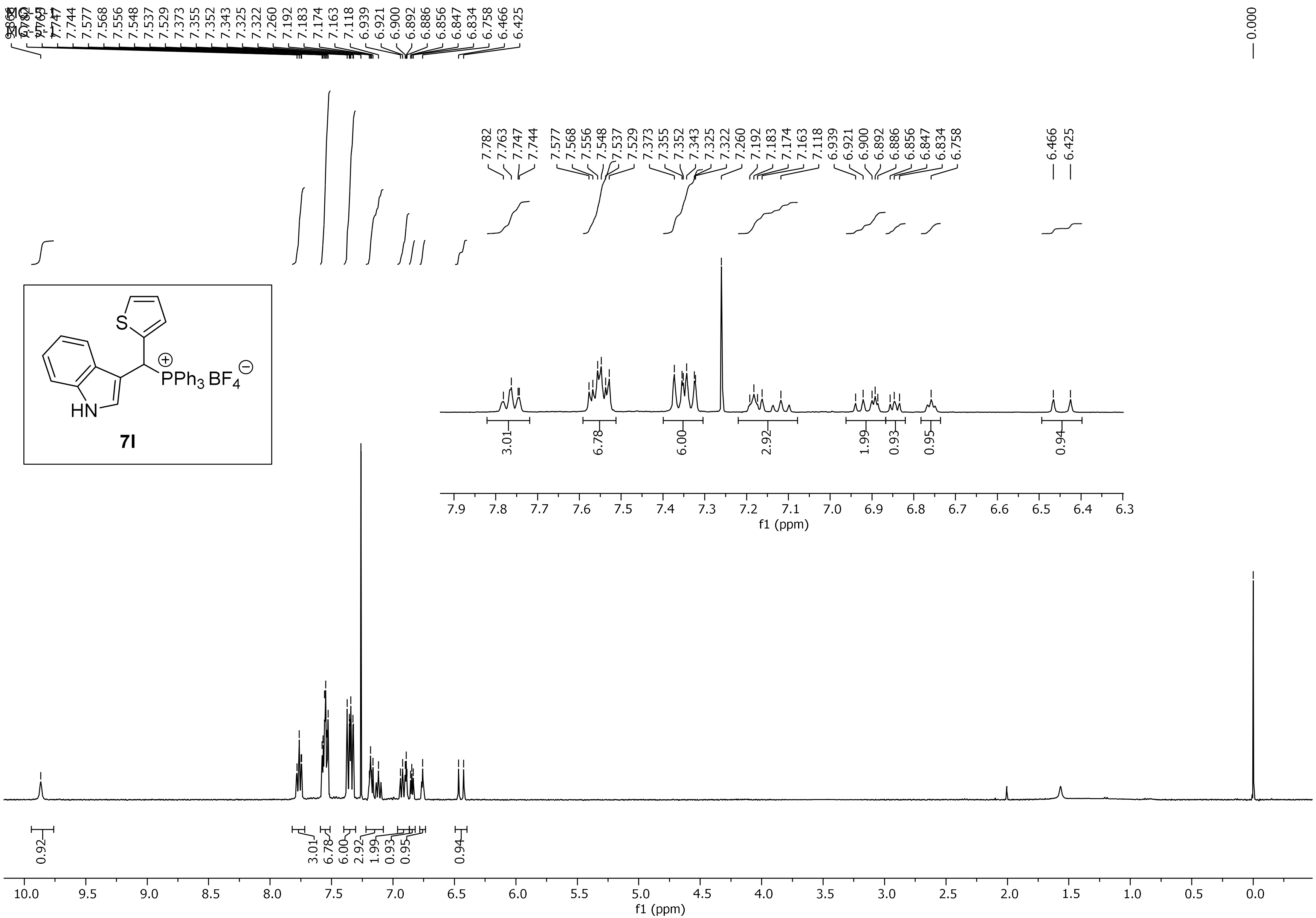

^{1}H NMR spectrum of 1-(*H*-indol-3-yl)-1-(tiophen-2-yl)methyltriphenylphosphonium tetrafluoroborate (**7l**); 400 MHz/$CDCl_3$/TMS; *δ* (ppm).

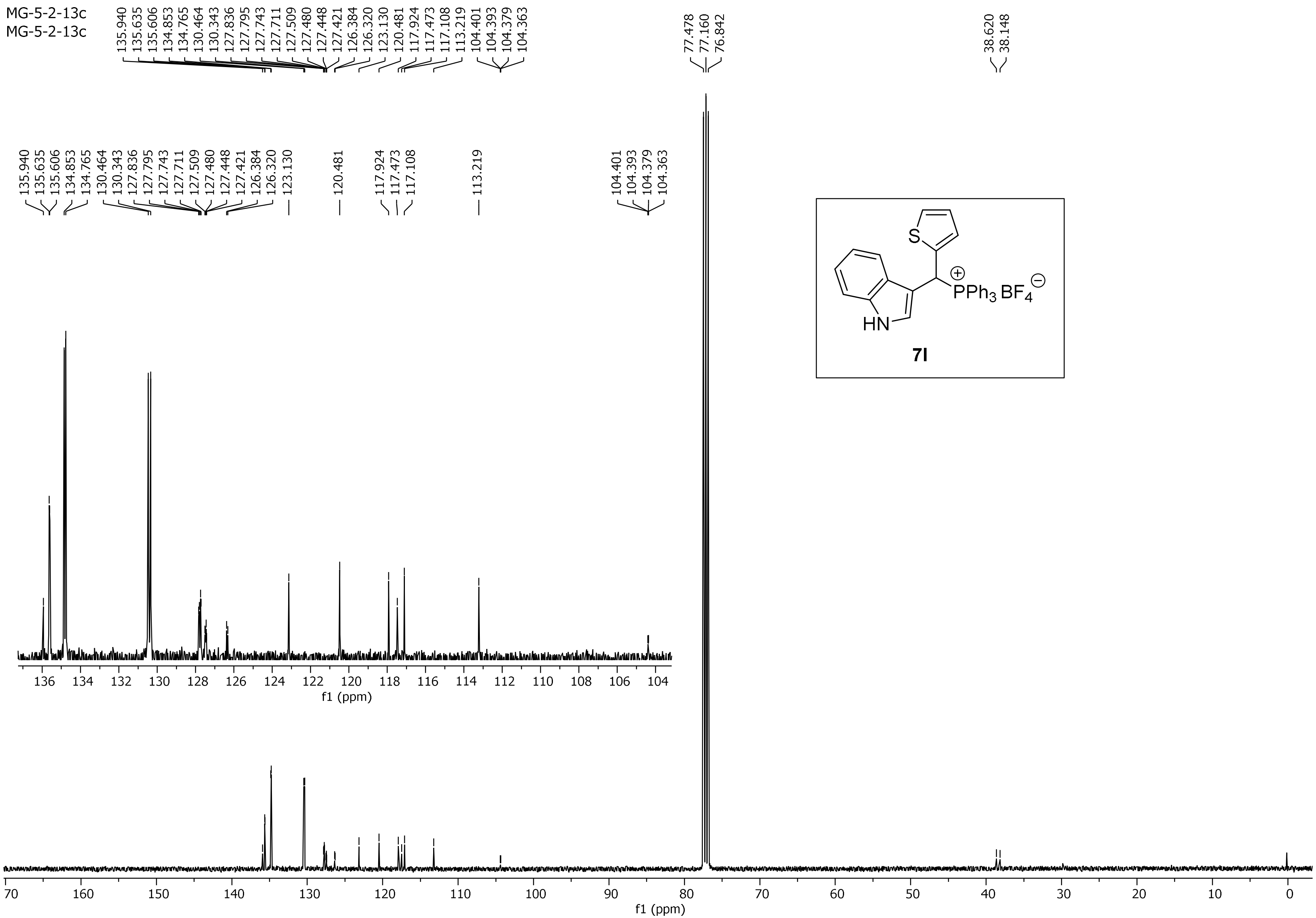

^{13}C NMR spectrum of 1-(*H*-indol-3-yl)-1-(tiophen-2-yl)methyltriphenylphosphonium tetrafluoroborate (**7l**); 100 MHz/$CDCl_3$/TMS; δ (ppm).

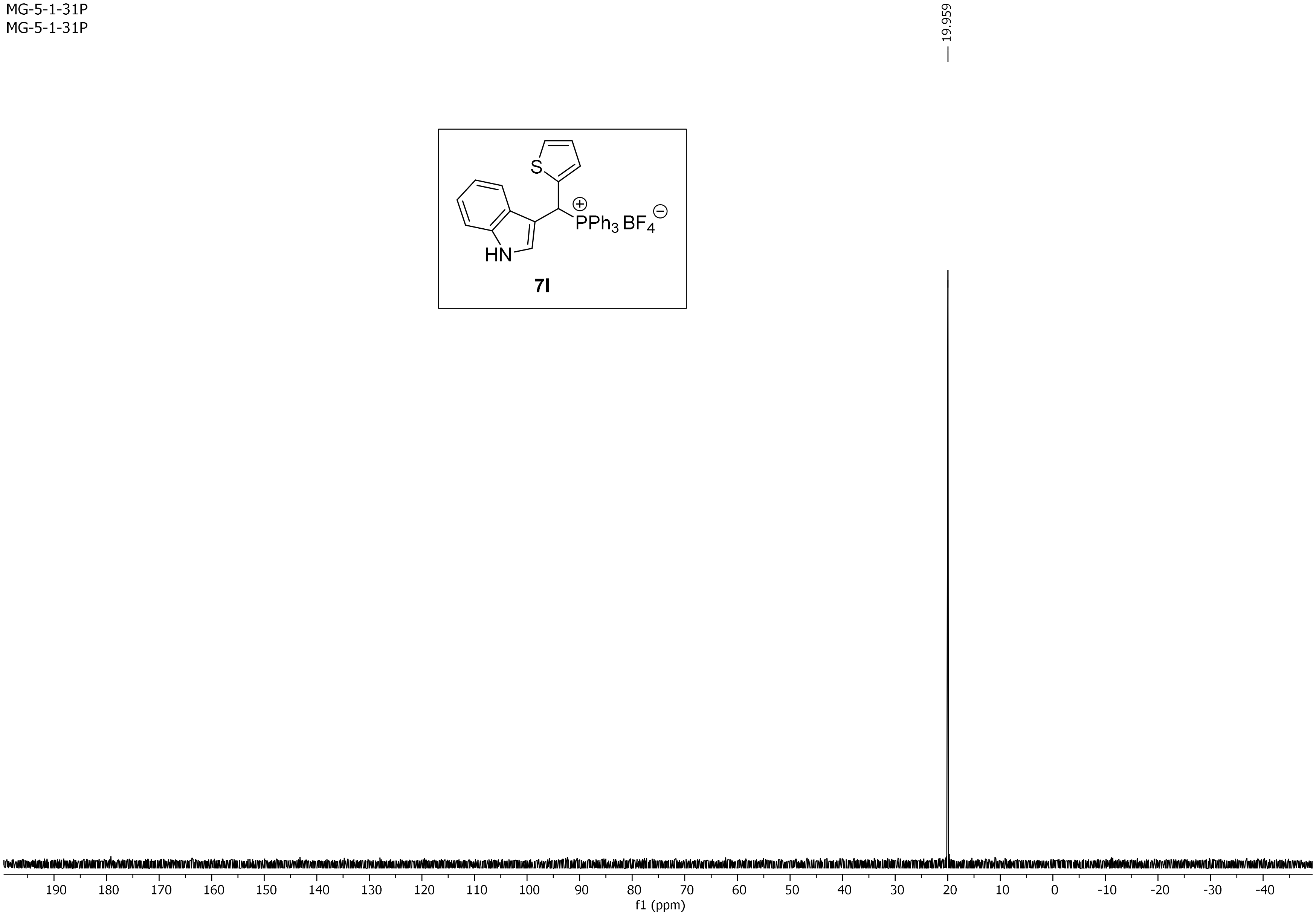

^{31}P NMR spectrum of 1-(*H*-indol-3-yl)-1-(tiophen-2-yl)methyltriphenylphosphonium tetrafluoroborate (**7l**); 161.9 MHz/$CDCl_3$; δ (ppm).

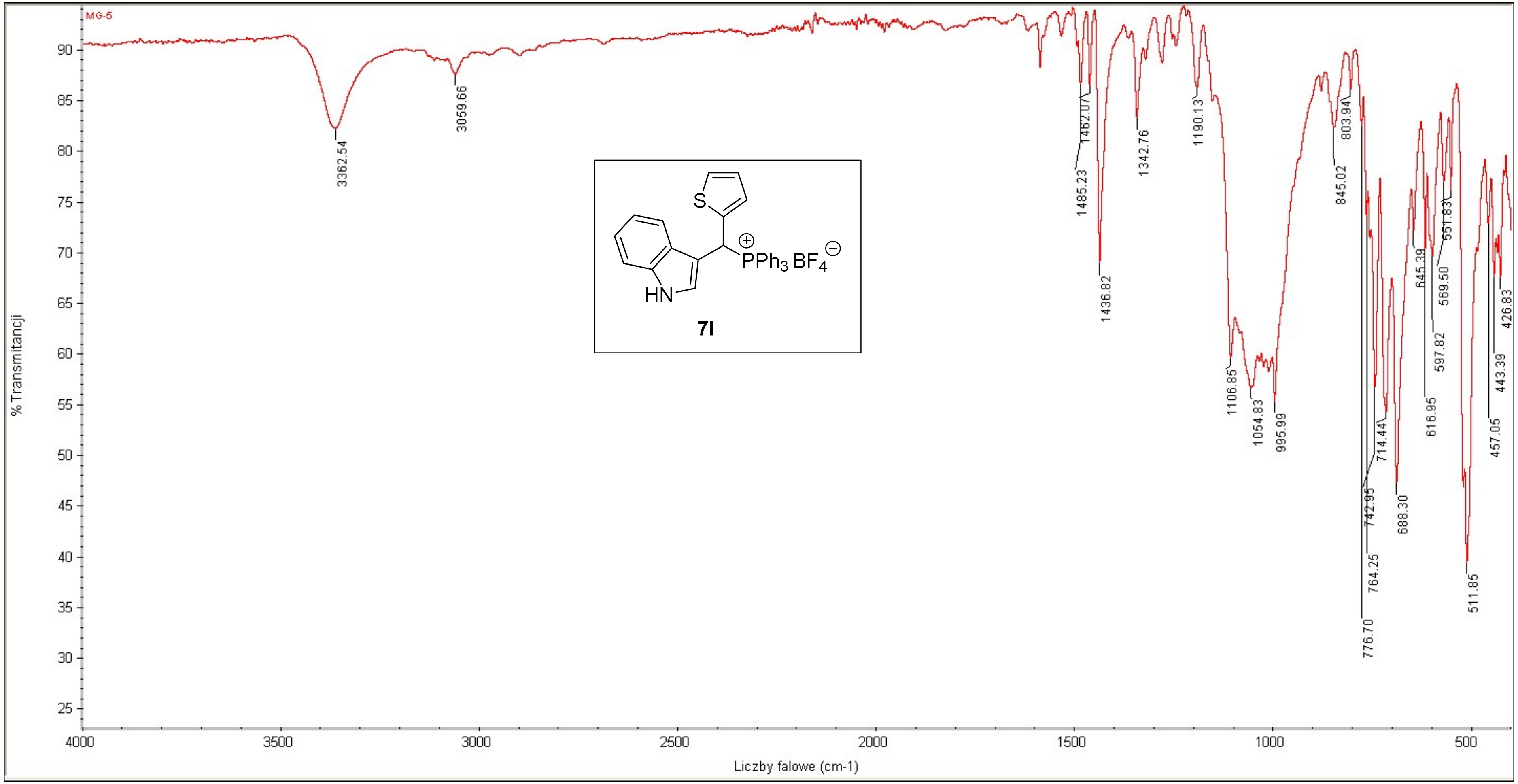

IR spectrum of 1-(*H*-indol-3-yl)-1-(tiophen-2-yl)methyltriphenylphosphonium tetrafluoroborate (**7l**); ATR (cm^{-1}).

Tolerance = 40.0 mDa / DBE: min = -10.0, max = 100.0
Element prediction: Off
Number of isotope peaks used for i-FIT = 2
Monoisotopic Mass, Even Electron Ions
7 formula(e) evaluated with 1 results within limits (up to 3 closest results for each mass)
Elements Used:

Mass	RA	Calc. Mass	mDa	PPM	DBE	Formula	i-FIT	i-FIT Norm	Fit Conf %	C	H	N	P	S
474.1437	100.00	474.1445	-0.8	-1.7	20.5	C31 H25 N P S	344.4	n/a	n/a	31	25	1	1	1

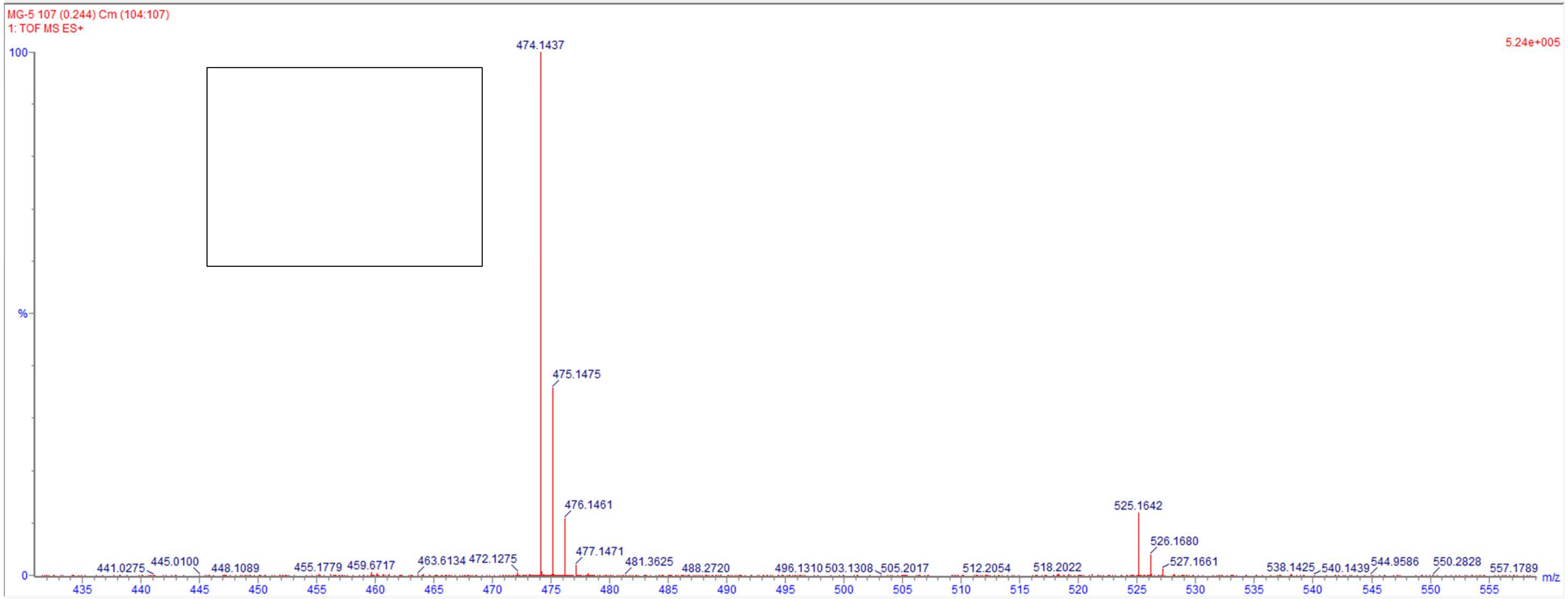

MS spectrum of 1-(*H*-indol-3-yl)-1-(tiophen-2-yl)methyltriphenylphosphonium tetrafluoroborate (**7l**).

Table S1. Characteristic signals (^{1}H and ^{31}P{^{1}H} NMR) for the identification of compounds **1o-x**.

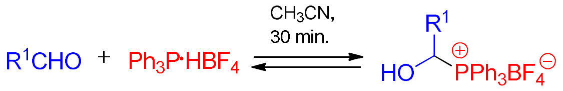

2 6 1

No.	Phosphonium salts 1	Solvent	^{1}H NMR, ppm $C_{\alpha}H$ and/or OH	^{31}P{^{1}H} NMR, ppm
**1o**	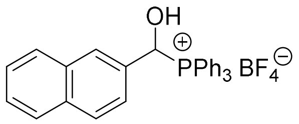	CD3CN	5.72 (dd, *J* = 16.7, 4.4 Hz, 1H)	20.5
**1p**	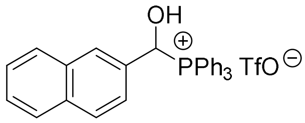	CD3CN	6.09 (br s)	20.4
**1q**	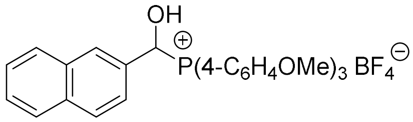	CD3CN	5.54 (br d, *J* = 14.5 Hz, 1H)	19.3
**1r**	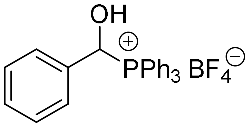	CD3CN	6.62 (dd, *J* = 4.8, 4.0 Hz, 1H), 5.59 (dd, *J* = 17.3, 5.8 Hz, 1H)	21.2/20.9[a]
**1s**	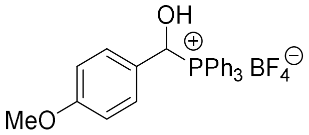	CD3CN	6.55 (br s, 1H), 5.49 (dd, *J* = 17.7, 5.3 Hz, 1H)	20.4
**1t**	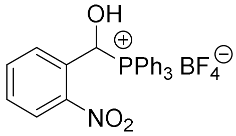	CD3CN	5.86 (dd, *J* = 13.1, 6.3 Hz, 1H)	21.8
**1u**	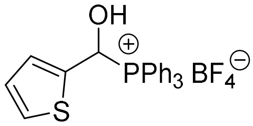	CD3CN	5.82 (dd, *J* = 17.1, 5.9 Hz, 1H)	21.0
**1v**	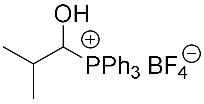	CD3CN	5.49 (ddd, *J* = 7.7, 3.7, 1.9 Hz, 1H), 4.96 (dd, *J* = 9.7, 7.9 Hz, 1H)	19.3/19.1[a]
**1w**	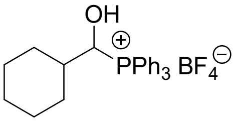	CDCl3	5.63 (br s, 1H), 5.25 (br t, *J* = 9 Hz, 1H)	18.8
**1x**	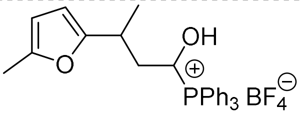	CDCl3	6.27 (d, *J* = 3.6 Hz, 1H), 5.54-5.49 (m, 1H); 5.91 (d, *J* = 3.0 Hz, 1H), 5.37-5.35 (m, 1H)[b]	16.7; 15.4[b]

[a] Spectra recorded also in CDCl3; [b] A mixture of two diastereomers is formed in the ratio of 1:1.9.

Table S2. Comparison of yields in one-pot and step-by step methodology for the reactions of 1-hydroxyalkylphosphonium salts **1** with the amide-type substrates.

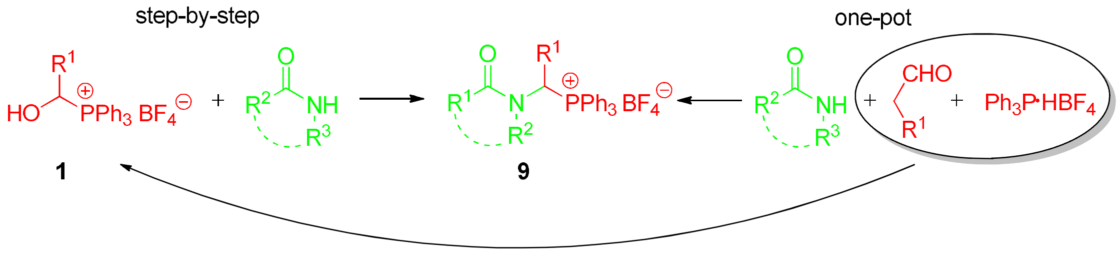

No.	1-(*N*-acylamino)alkylphosphonium salts 9	step-by-step *this work, %*	one-pot *previous work [24], %*
**9a**	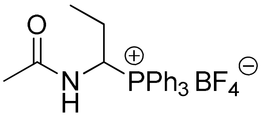	86	86
**9b**	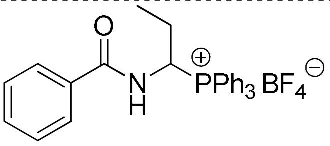	86	55
**9c**	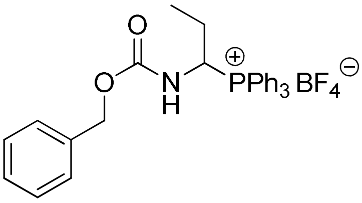	75	79
**9d**	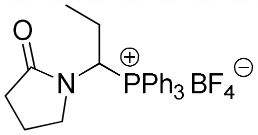	77[a]	98[a]
**9e**	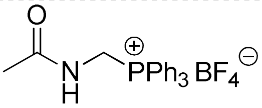	91	91

[a] Reactions were conducted in different temepratures (50°C: step-by-step vs. 100°C: one-step)
